# Structure-Based Design of a Potent and Selective YTHDC1 Ligand

**DOI:** 10.1021/acs.jmedchem.4c00599

**Published:** 2024-05-24

**Authors:** František Zálešák, Francesco Nai, Marcin Herok, Elena Bochenkova, Rajiv K. Bedi, Yaozong Li, Francesco Errani, Amedeo Caflisch

**Affiliations:** Department of Biochemistry, University of Zurich, Winterthurerstrasse 190, CH-8057 Zurich, Switzerland

## Abstract

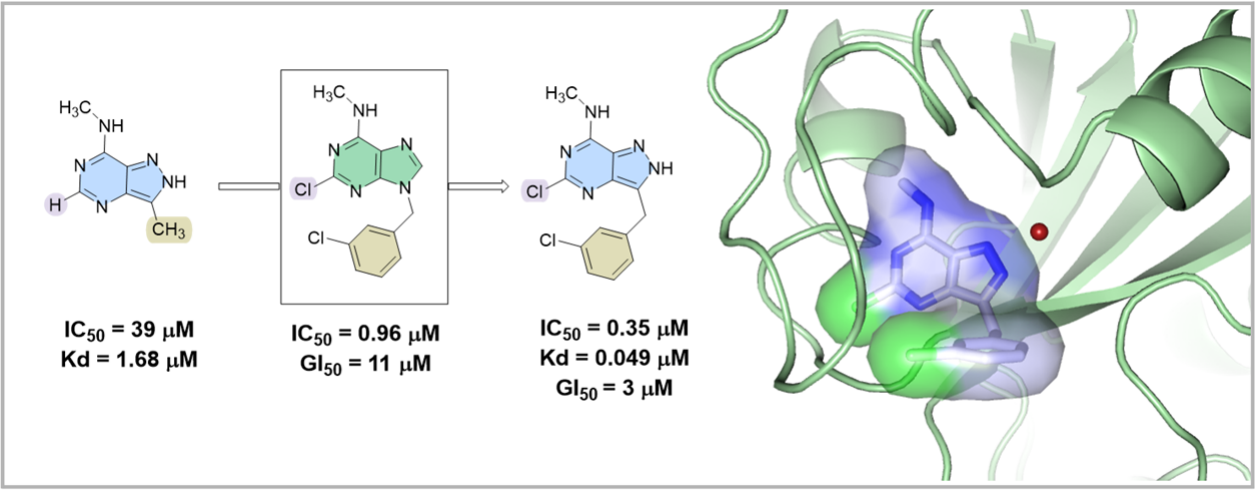

N^6^-Adenosine methylation (m^6^A) is a prevalent post-transcriptional modification of mRNA, with YTHDC1 being the reader protein responsible for recognizing this modification in the cell nucleus. Here, we present a protein structure-based medicinal chemistry campaign that resulted in the YTHDC1 inhibitor **40**, which shows an equilibrium dissociation constant (*K*_d_) of 49 nM. The crystal structure of the complex (1.6 Å resolution) validated the design. Compound **40** is selective against the cytoplasmic m^6^A-RNA readers YTHDF1–3 and YTHDC2 and shows antiproliferative activity against the acute myeloid leukemia (AML) cell lines THP-1, MOLM-13, and NOMO-1. For the series of compounds that culminated into ligand **40**, the good correlation between the affinity in the biochemical assay and antiproliferative activity in the THP-1 cell line provides evidence of YTHDC1 target engagement in the cell. The binding to YTHDC1 in the cell is further supported by the cellular thermal shift assay. Thus, ligand **40** is a tool compound for studying the role of YTHDC1 in AML.

## Introduction

Post-transcriptional changes of eukaryotic mRNA play a pivotal role in cellular processes.^1^ Before leaving the nucleus, mRNA undergoes a series of chemical alterations, such as methylation, acetylation, and splicing.^2,3^ These modifications directly affect mRNA stability, processing, and translation efficiency.^4,5^ Recent investigation of post-transcriptional events have given rise to a dynamic research field known as epitranscriptomics.^3^ Among the diverse mRNA modifications in the human transcriptome, the most prevalent and extensively studied is the N^6^ methylation of adenosine (m^6^A).^6^ This reversible process is facilitated by proteins referred to as “writers”, such as METTL3/14,^7,8^ while the demethylation is achieved by “erasers” like FTO or ALKBH5.^9,10^ Our research group has made recent contributions to this field through the development of small-molecule inhibitors targeting METTL3/14, demonstrating the modulation of interconnected cellular events.^11,12^

Recognition of the m^6^A modification is mediated by “readers” that subsequently impact downstream processes.^13^ The YTH family, comprising five proteins (YTHDC1, YTHDC2, and YTHDF1–3), is the best characterized family of m^6^A readers.^14^ Due to their crucial role in diverse biological processes, these proteins hold great promise as therapeutical targets.^15,16^ Predominantly localized in the nucleus, YTHDC1 is responsible for the regulation of pre-mRNA splicing and mRNA export from the nucleus.^17^ A growing number of reports associate the activity of YTHDC1 with various biological functions, including embryonic development, neuronal development, and others.^18^ Furthermore, its critical role has been identified in various types of cancer.^19,20^

Here, we focus on the function of YTHDC1 in acute myeloid leukemia (AML). The gene was identified as essential for AML in a genome-wide CRISPR knockout screening.^20^ In another study, Sheng et al. indicate an oncogenic role of YTHDC1 in the regulation of leukemogenesis by MCM4, a component of the MCM complex responsible for DNA replication.^21^ Chen et al. showed that m^6^A-dependent export of mRNA from the nucleus, mediated by YTHDC1, in combination with lncRNA MALAT1 promotes the expression of fusion genes such as PML-RARA, MLL-ENL, or MLL-AP9 characteristic for particular types of leukemia.^22^ These examples describe the distinct role of YTHDC1 in blood cancers, indicating the need for therapeutics targeting this specific m^6^A reader protein.

Our group recently reported fragments binding to YTHDC1 or YTHDF2, respectively.^23,24^ Other studies in the literature have reported inhibition using rather promiscuous binders providing limited selectivity toward proteins of the YTH family,^25−27^ except for a recent report which describes a selective YTHDC1 inhibitor identified by *in vitro* high-throughput screening followed by a structure-based optimization.^28^

In this study, we present a structure-based design campaign aimed at developing a potent and selective ligand of YTHDC1. Additionally, we provide biochemical evaluation based on the homogeneous time-resolved fluorescence assay (HTRF), isothermal titration calorimetry (ITC), thermal shift assay (TSA), and protein X-ray crystallography. We also report a direct connection between YTHDC1 inhibition and antiproliferative activity on acute myeloid leukemia cell lines (THP-1, MOLM-13, NOMO-1).

## Results and Discussion

**Figure 1:**
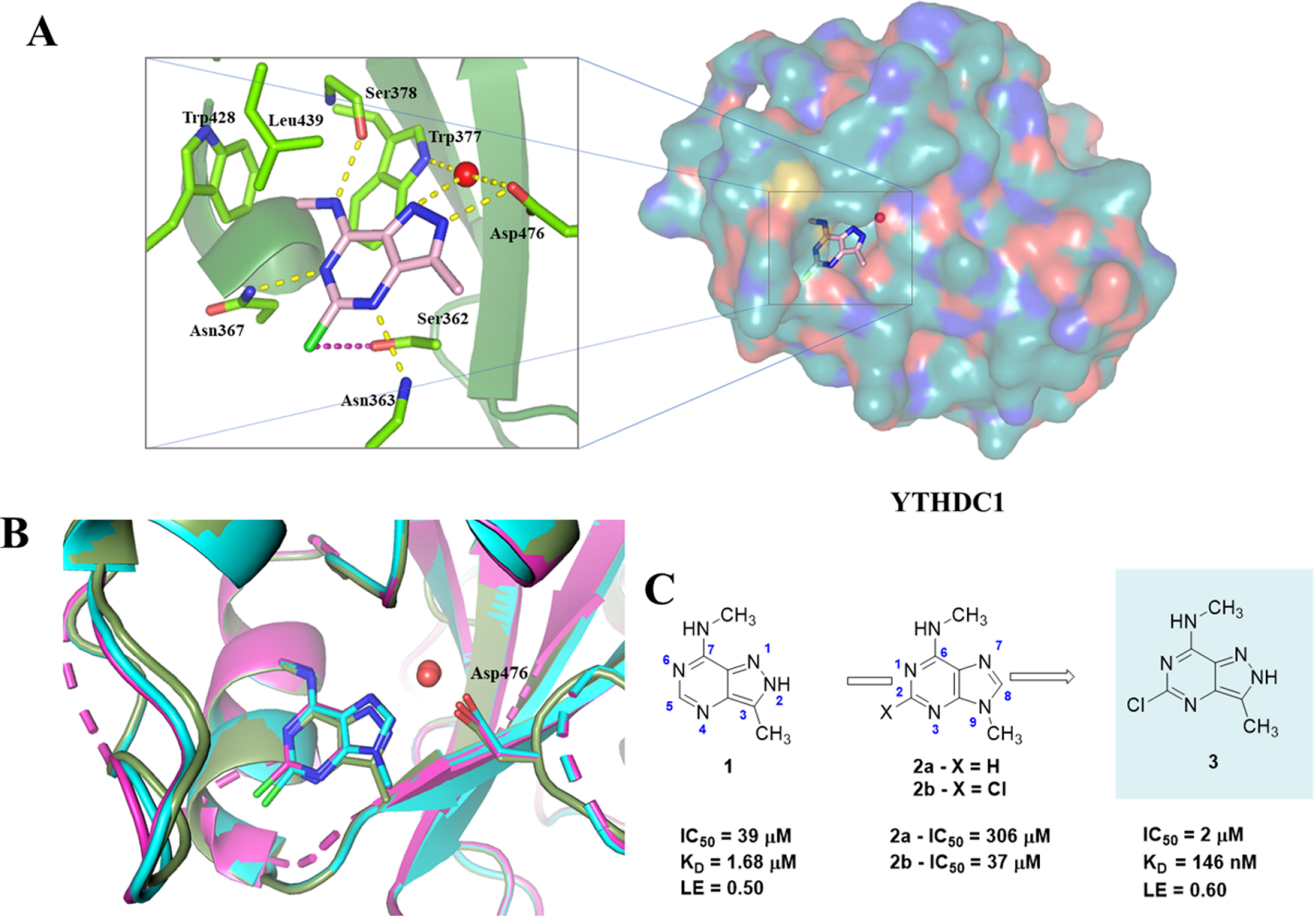
(A) Crystal structure of fragment **3** in the YTHDC1 binding site with relevant residues (light green). Hydrogen bonds are displayed in yellow; halogen-hydrogen bond donor interaction is displayed in magenta (C–Cl···H angle = 76°; Cl···O distance = 3.8 Å). (B) Structural overlap of fragments **1**–**3** in the binding site of YTHDC1 (compound **1—**green, PDB code 7P8F;^23^ compound **2b**—cyan, PDB code 8Q2Q; compound **3**—magenta, PDB code 8Q2R). (C) 2D structures of fragments **1**–**3**. Fragment **1** is the starting point of the optimization.^23^

The present medicinal chemistry campaign builds upon our initial *in silico* screening for YTHDC1.^23,29^ The structure-based medicinal chemistry optimization started here with the ligand-efficient fragment **1** (Figure 1C) which had been characterized by biochemical assays and crystallography.^23^ Structurally, this fragment contains a pyrazolo[4,3-*d*]pyrimidine core that mimics the natural ligand m^6^adenine (we use m^6^adenine for nucleotide, in contrast to m^6^A for nucleoside). The recognition of fragment **1** is achieved by the aromatic cage consisting of two tryptophan residues (Trp428, Trp377) and five hydrogen bonds between the pyrazolopyrimidine ring and the binding pocket (Asn367, Asp476, backbone N-H of Asn363, backbone C=O of Ser378, and structural water-bridging side chains of Trp377 and Asp476). Notably, unlike the natural ligand, the pyrazolopyrimidine **1** forms an additional hydrogen bond with the side chain of Asp476 (Figure 1, PDB: 7P8F). This interaction significantly enhances the YTHDC1 affinity of fragment **1** by nearly 10-fold compared to m^6^-adenine (IC_50_ = 39 vs 306 μM).^23^ Upon evaluating the binding pose of pyrazolopyrimidine **1** within the binding pocket, we identified two positions on the aromatic core that are conducive to ligand growth and optimization. The first position lies between N^6^ and N^4^ of the pyrimidine ring. The substituent in this position leads to a small pocket, suggesting that a suitable substituent could occupy the space and enhance binding. The second substituent leads from C^3^ toward a shallow, positively charged pocket that binds the negatively charged part of RNA in the natural ligand.

During the initial stages of the campaign, we identified that a chloride substituent located between N^6^ and N^4^ fits into the small pocket forming a halogen-hydrogen bond donor interaction^30^ with the side chain hydroxyl of Ser362 (C–Cl···H angle = 76°; Cl···O distance = 3.8 Å) (Figure 1A), which substantially improves the binding affinity. (Note the difference in the numbering of purine and pyrazolopyrimidine core as shown in Figure 1C). This improvement was demonstrated by comparing the potency of nonchlorinated **2a** and chlorinated m^6^adenine **2b** resulting in IC_50_ values of 306 and 37 μM, respectively. However, the adenine aromatic core cannot form a conventional hydrogen bond with Asp476, unlike pyrazolopyrimidine **1** moiety. Therefore, we decided to merge the two structural features and synthesize 5-chloropyrazolopyrimidine fragment **3**. This compound exhibited improved potency together with ligand efficiency (IC_50_ = 2 μM, LE = 0.60) and submicromolar equilibrium dissociation constant (*K*_D_ = 146 nM, Figure S6) measured by isothermal titration calorimetry (ITC). As mentioned above, an alternative position for ligand growth involved extending or replacing the methyl substituent at position 3 of 5-chloropyrazolopyrimidine **3**. Considering the synthetic challenges, we decided to pursue the optimization using 2-chloropurine **2b** instead of 5-chloropyrazolopyrimidine **3**. This approach offered the advantage of convenient derivatization at N^9^ of the purine moiety, as compared to C^3^ in the equivalent position of the pyrazolopyrimidine core. The primary rationale behind this choice was to focus on extending the purine scaffold and synthesizing only the most promising examples in combination with the 5-chloropyrazolopyrimidine **3**. This strategy was based on the observation of essentially identical binding poses among the fragments (see Figure 1B) and the hypothesis that interactions outside the aromatic cage would be preserved across different fragments.

### Ligand Growing—First Round of Optimization

**Figure 2:**
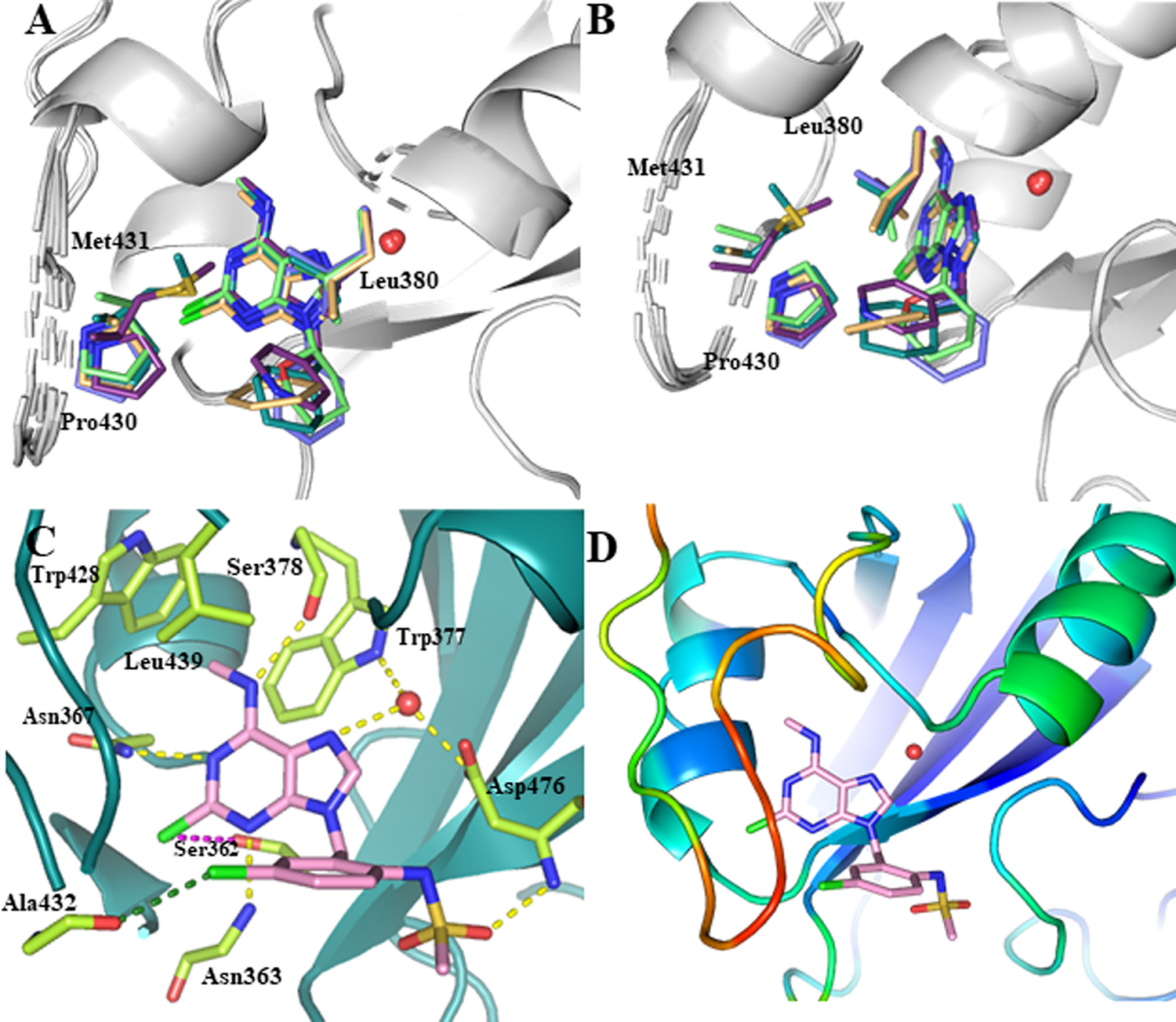
Interactions between ligands and YTHDC1 in crystal structures of the complexes. (A, B). Structural overlap of the complexes with compounds **4** (slate; PDB code 8Q2S), **5** (light orange; PDB code 8Q2U), **6** (magenta; PDB code 8Q2U), **7** (lime; PDB code 8Q2 V), and **8** (deep teal; PDB code 8Q2W). Compounds **5** and **6** have a methylene linker so that the phenyl and pyridine ring, respectively, form van der Waals interactions with the Pro430, Met431, and Leu380. (C) Binding pose of ligand **31** shows a halogen-hydrogen bond donor interaction (magenta, C–Cl···H angle = 76°, Cl···H distance = 3.6 Å), a halogen bond (forest green, C–Cl···O angle = 153°, Cl···O distance = 3.4 Å), and a hydrogen bond between an O atom of the sulfonamide and the backbone NH of Asp476 (also for ligand **13**). (D) The protein backbone is colored according to the crystallographic *B*-factor (disorder increasing from blue to red).

In the initial screening phase, we opted to replace/extend the methyl group at position 9 with various aliphatic and aromatic rings, including both −CH_2_– bridged and nonbridged structures (Table 1). Notably, the substituents with a methylene linker (**5**, **6**, **8**, **9**, and **11**) exhibited low micromolar potency (IC_50_ = 3, 6, 8, 11, and 9 μM, respectively) in comparison with compounds devoid of the methylene: **4** (IC_50_ > 100 μM) and **7** (IC_50_ = 47 μM). These results suggest the presence of van der Waals interaction between the rings and lipophilic residues (Leu380, Pro431, Met434). The flexibility provided by the methylene allows the aromatic ring to achieve better interactions within the binding site residues, which is not the case for compounds **4** and **7** (Figure 2A,B). However, there was one exemption among the tested molecules. Compound **10** does not feature a methylene bridge and has a carboxyl group in *meta* position which resulted in enhanced potency (IC_50_ = 11 μM) with respect to compounds **4** and **7**. This observation indicates that the absence of hydrophobic interactions is compensated with ion–ion or dipole–ion interactions. Such interactions are likely to be formed between negatively charged carboxyl group and positively charged residues within the binding pocket. The significant impact of the carboxylic group is evident when comparing the potency of compound **4**, featuring a nonsubstituted phenyl ring to its carboxylic acid derivative **10**.

**Table 1:**
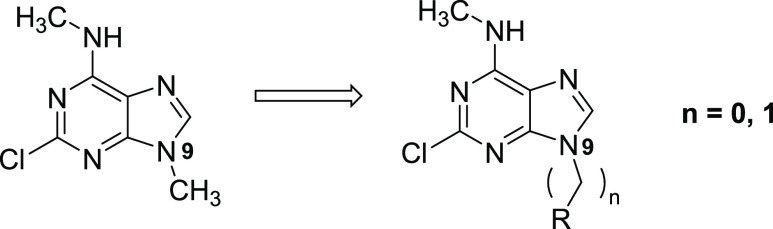

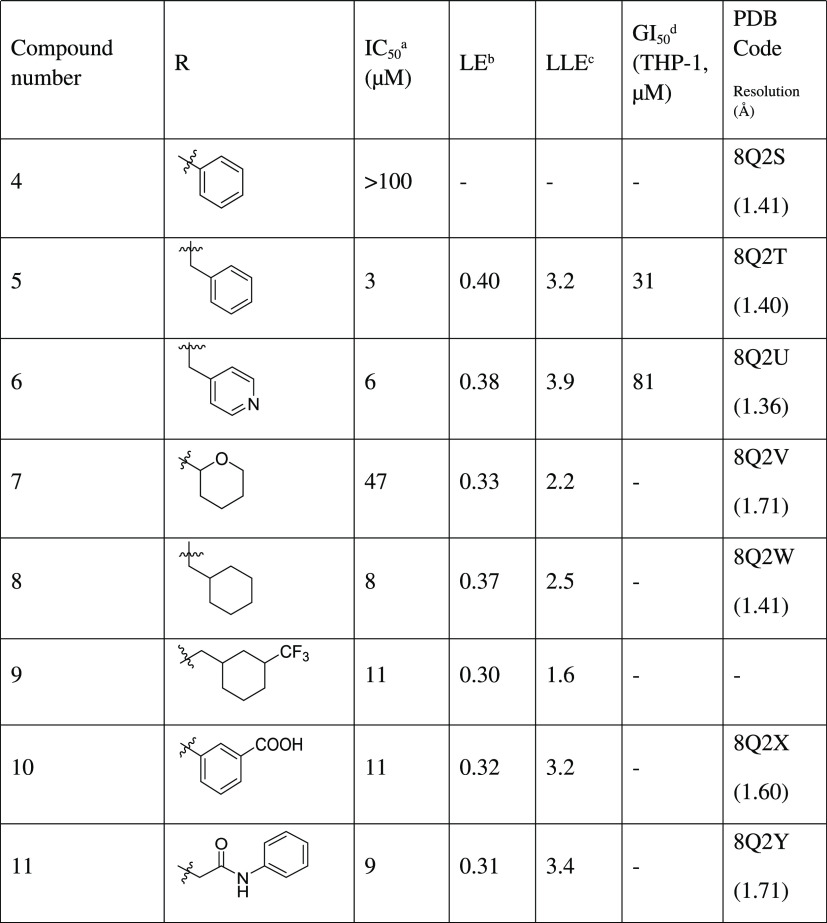
2-Chloropurine Derivatives with Different Substituents on N^9^

a Homogeneous time-resolved fluorescence (HTRF).

b Ligand efficiency (kcal·mol^–1^ heavy atom count^–1^).^31^

c Lipophilic ligand efficiency (pIC_50_ – *c* log *P*).^31^

d Growth inhibition 50 (GI_50_) values after 72 h treatment (THP-1).

### Second Round of Optimization—Benzyl Rings SAR Study

Due to its promising potency, the benzyl substituent was retained for further optimization. The easily accessible and commercially available nature of benzyl halides facilitated an efficient structure–activity relationship (SAR) study, enabling identifications of potent functionalization. The benzylic scaffolds we synthesized and tested could be divided into subgroups based on the position and number of substituents (Table 2). In the following, *ortho, meta*, and *para* positions refer to the substitution relative to the methylene connecting the purine heterocycle.

**Table 2:**
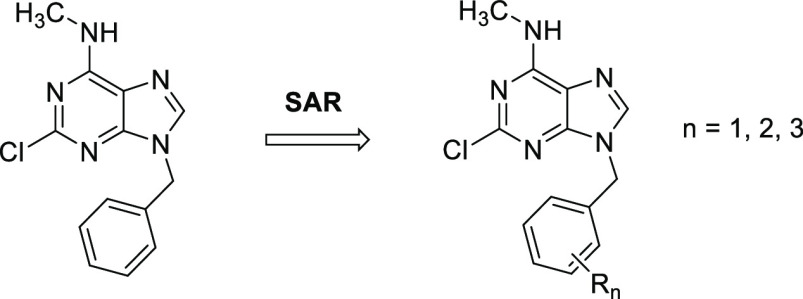

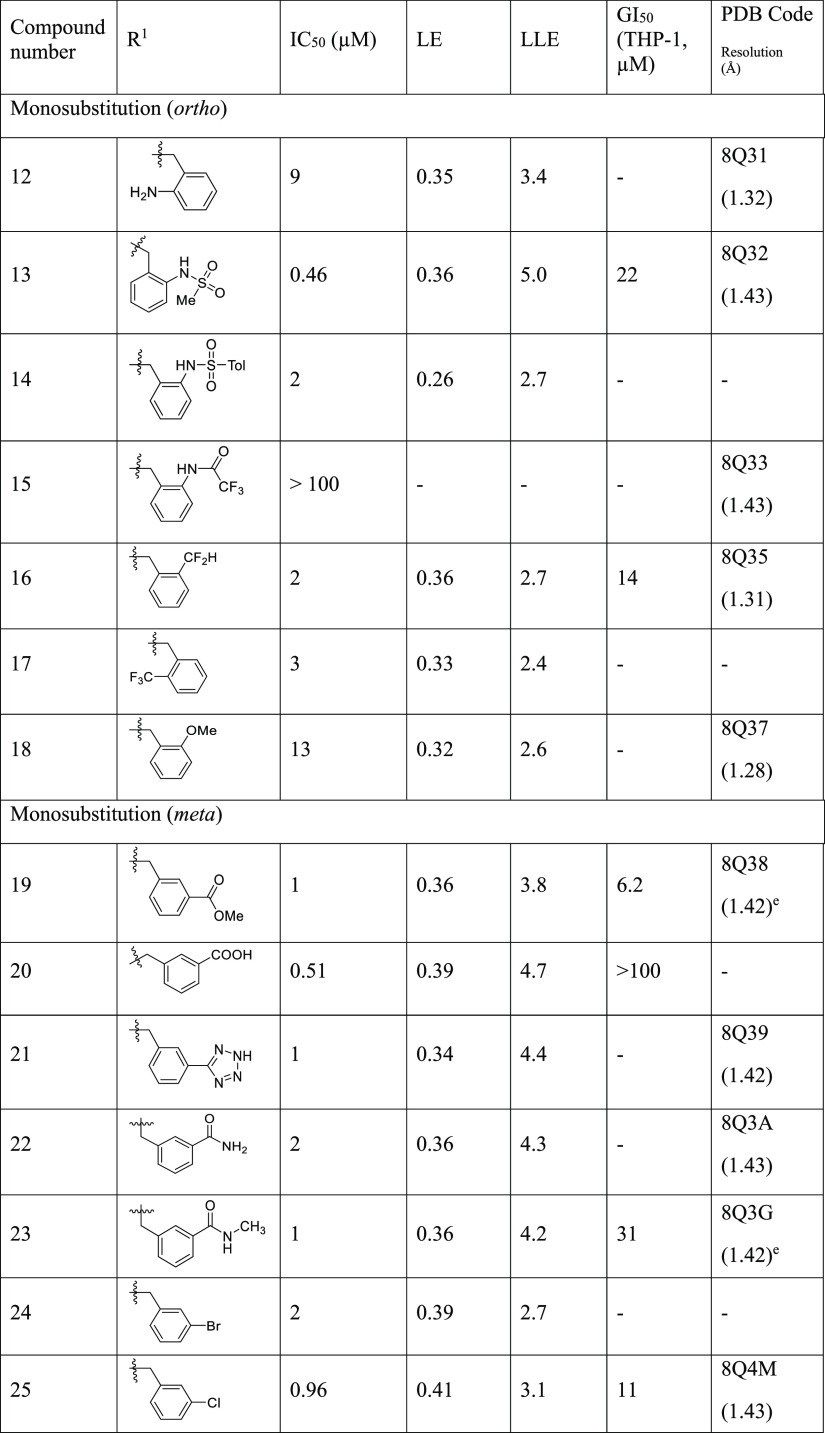

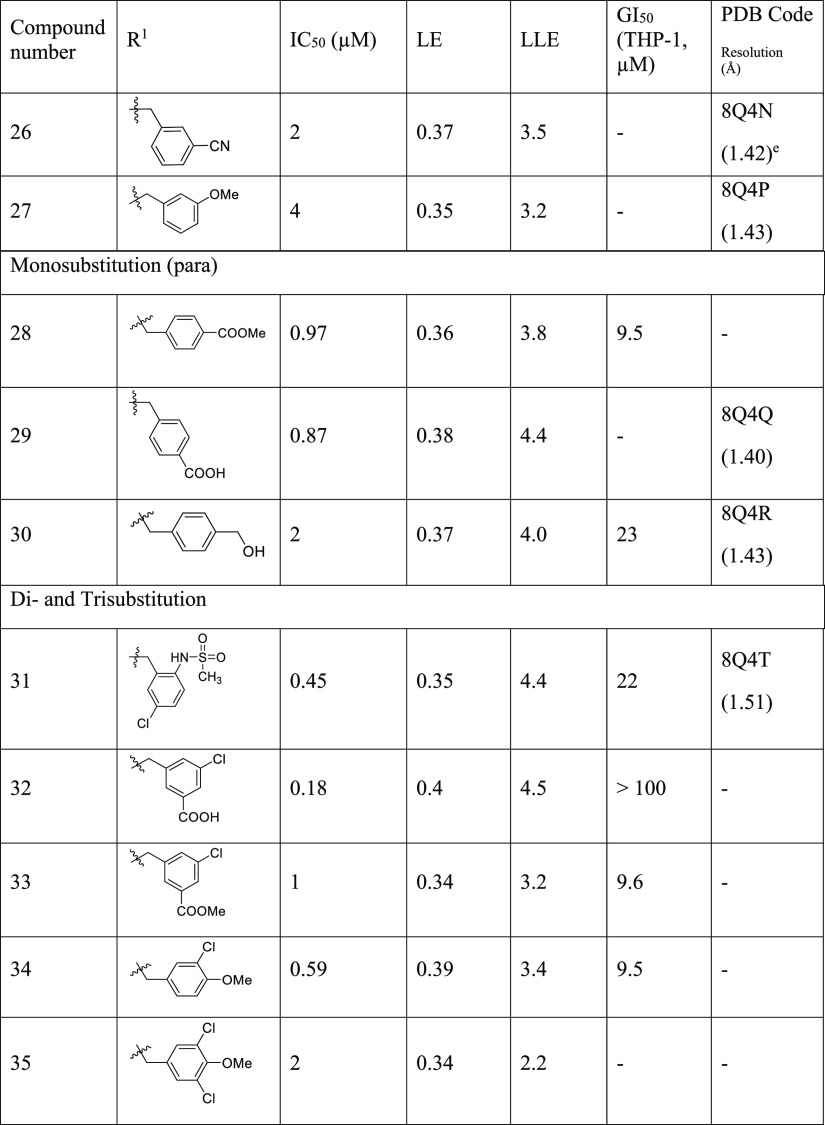
Structure–Activity Relationship: Benzyl Ring Substitutions^a^

a Same as Table 1.

e No density beyond the phenyl ring.

Among the *ortho-*substituted derivatives, compound **12**, containing free amine moiety, and its sulfonamide derivatives (**13, 14**) exhibited an IC_50_ value of 9, 0.46, and 2 μM, respectively. The potency of compound **13** could be potentially attributed to an additional hydrogen bond formed between the sulfonamide oxygen and the backbone N–H of Asp476 (Figure 2C). On the other hand, the presence of trifluoroacetic amide **15** and methoxy derivative **18** provided the IC_50_ value above 100 and 13 μM, respectively. This suggested that the carbonyl of **15** is unable to adopt the favorable geometry orientation for hydrogen bond formation observed between the sulfonamide oxygen of **13** and Asp476. Although perfluorinated alkyl substituents (**16**, **17**) exhibited lower potency in comparison with the most promising compound from this set (IC_50_ = 2 and 3 μM versus 0.46 μM for compound **14**), compound **16** showed antiproliferative activity against THP-1 cell line (GI_50_ = 14 μM). Additionally, compounds **12, 13, 15, 16**, and **18** were soaked to YTHDC1 enabling X-ray crystallography validation of their binding poses with high resolution (<1.5 Å).

**Figure 3:**
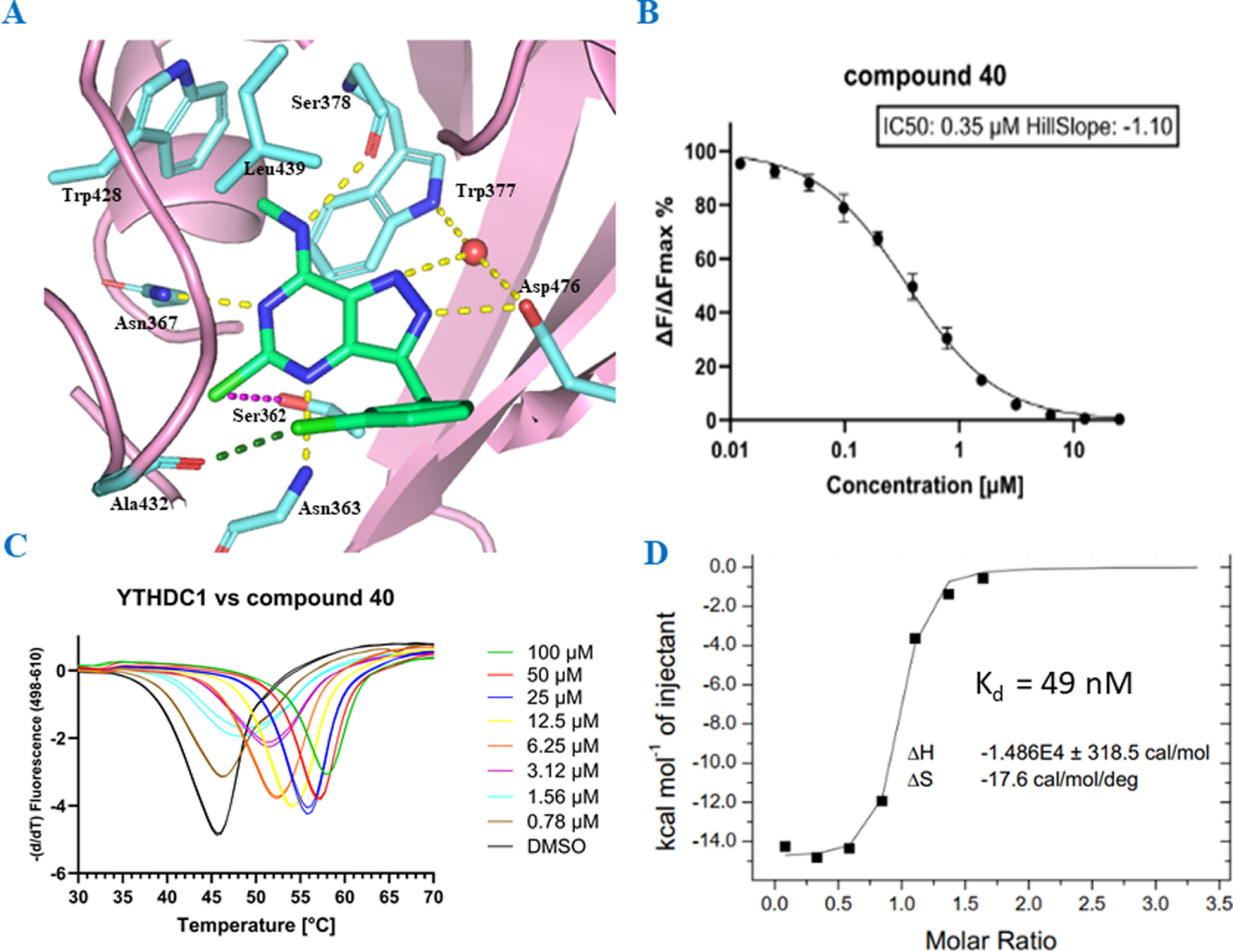
Biochemical evaluation of compound **40**. (A) Binding pose of compound **40** and interactions with YTHDC1 (PDB code 8Q4W). Halogen-hydrogen bond donor interaction is displayed in magenta (C–Cl···H angle = 73°, Cl···H distance 3.5 Å); halogen bond in forest green (C–Cl···O angle = 159°, distance Cl···O = 3.2 Å). (B) HTRF dose–response curves of YTHDC1 and compound **40**. (C) Dose–response thermal shift of YTHDC1 in the presence of compound **40**. (D) Isothermal titration calorimetry curve for YTHDC1 and compound **40**.

Among the *meta-*substituted derivatives, our focus primarily centered on carboxylic acid derivatives, as encouraged by the aforementioned significant potency increase between compounds **4** and **10**. To expand structural and functional diversity, we also evaluated purine analogues containing halogen and methoxy groups. As expected, *meta-*carboxylic acid analogue **20** demonstrated the highest affinity with an IC_50_ value of 0.51 μM. In contrast, the tetrazole heterocycle **21**, serving as carboxylic acid bioisoster, displayed lower affinity (IC_50_ = 1 μM) compared to the free carboxylic acid compound **20**. Conversely, *meta-*substituted compounds with amide (**22, 23**) and methyl ester moiety **19** exhibited affinity (IC_50_ = 2, 1, 1 μM) compared to nitrile group **26** (IC_50_ = 2 μM**)** and did not surpass the potency of compound **20**. We hypothesized that the improved binding toward YTHDC1 might be a result of ion–ion or ion–dipole interaction involving the positively charged side chain of Arg475. However, X-ray crystallography could not confirm this, due to the lack of density for both the carboxylic group and the Arg475 side chain. Regarding the antiproliferative activity against THP-1, the methyl ester derivative **19** exhibited low micromolar GI_50_ (6.2 μM) while carboxylic acid derivative **20**, which showed greater potency in the HTRF assay, had a minimal effect (GI_50_ > 100 μM), likely due to poor cell permeation of the charged carboxylic residue at physiological pH. Among the compounds tested, we also discovered that a chloride substituent in the *meta* position exhibited enhanced binding with an IC_50_ value of 0.96 μM (compound **25**) and displayed antiproliferative activity against THP-1 (GI_50_ = 11 μM). This compound also exhibited a very favorable LE value (0.41). The improved affinity could potentially arise from the formation of a halogen bond. X-ray crystallography did not provide evidence for an interaction between the chlorine substituent and the backbone carbonyl of Ala432 for compound **25**. However, the presence of halogen bond was later confirmed by similar compounds (**31**—Figure 2C, and **40**—Figure 3A). On the other hand, a bigger and more lipophilic bromide substituent present in compound **24** resulted in a 2-fold decrease in potency (IC_50_ = 2 μM) in comparison to compound **25**. Because of the positive effect observed with carboxylic acid functionality, we also prepared derivatives of carboxylic acid (**28**, **29**) and benzyl alcohol derivative **30** in the *para* position. However, these compounds did not exhibit improved binding compared to the ones with functional groups in *meta* position, presumably due to an unfavorable orientation of the substituents that lead toward solvent exposed area out of the pocket.

The combination of the most potent aromatic ring substitutions was tested by compound **31**, namely, methylsulfonamide in *ortho* position (compound **13**) and chloride in *meta* (compound **25**), but did not improve potency with respect to the monosubstituted analogue **13**. Although compound **31** showed an IC_50_ of 0.45 μM, the presence of the sulfonamide moiety once again resulted in mediocre activity against THP-1 growth (GI_50_ = 22 μM). On the other hand, compound **32** containing *meta-*chloro and *meta-*carboxyl substitution showed greater enhancement in binding resulting in the most potent compound (IC_50_ = 0.18 μM). Unfortunately, the biochemical potency increase was not transferred into the improved antiproliferative activity (GI_50_ > 100 μM) which is also true for its methyl ester analogue **33** (GI_50_ = 9.6 μM). Furthermore, while the combination of *para-*methoxy and *meta-*chloro substitution provided submicromolar potency (compound **34**, 0.59 μM), the presence of two chloro substituents in meta position diminished the binding (compound **35**, IC_50_ = 2 μM).

### Headgroup Optimization

After optimizing the benzylic side chain and identifying suitable substituents, we shifted our focus back to the heterocyclic core optimization. For this reason, we explored further modifications on the purine fragment while maintaining the *meta-*chlorobenzyl substituent at N^9^ (Table 3). First, we confirmed that the presence of a hydrogen bond between the methylamino group and the backbone of Ser378 is essential for the binding. This was verified by testing compounds **38** and **39**, both containing a chlorine substituent at position 6 of the purine ring. The presence of chlorine atom instead of methylamino moiety resulted in a notable decrease in potency. Additionally, compound **36**, bearing fluoro substituent instead of chloro (position 2 of purine ring), pointing toward a small lipophilic pocket, also exhibited weakened binding (IC_50_ = 2 μM). Moreover, the substitution of the methylamino group with cyclopropyl amino moiety **37** also led to weakened binding, indicating that the aromatic cage of YTHDC1 is intolerant to increased bulkiness.

**Table 3:**
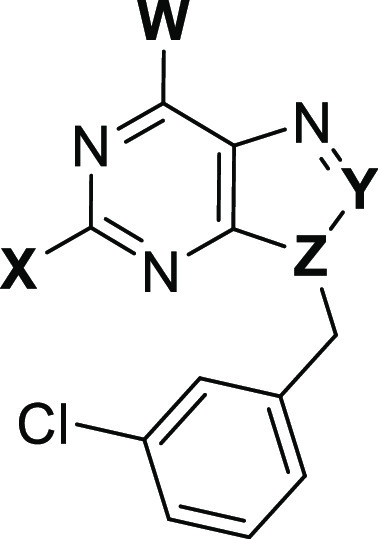

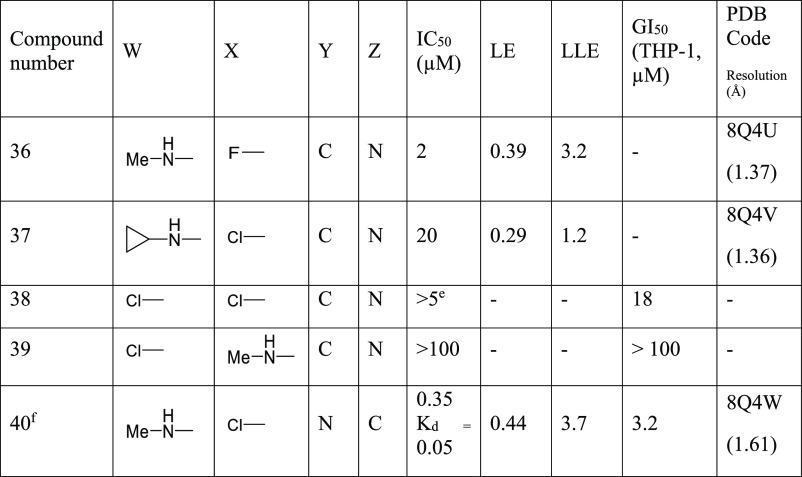
Additional Optimization of the Original Fragment^a^

a Same as Table 1. For compound **40**, the *K*_D_ determined by ITC is reported below the IC_50_ value.

e Tested only in single dose at 5 μM (103%)

f Compound **40** was tested in the form of HCl salt. The *K*_d_ determined by ITC is reported below the IC_50_ value.

**Figure 4:**
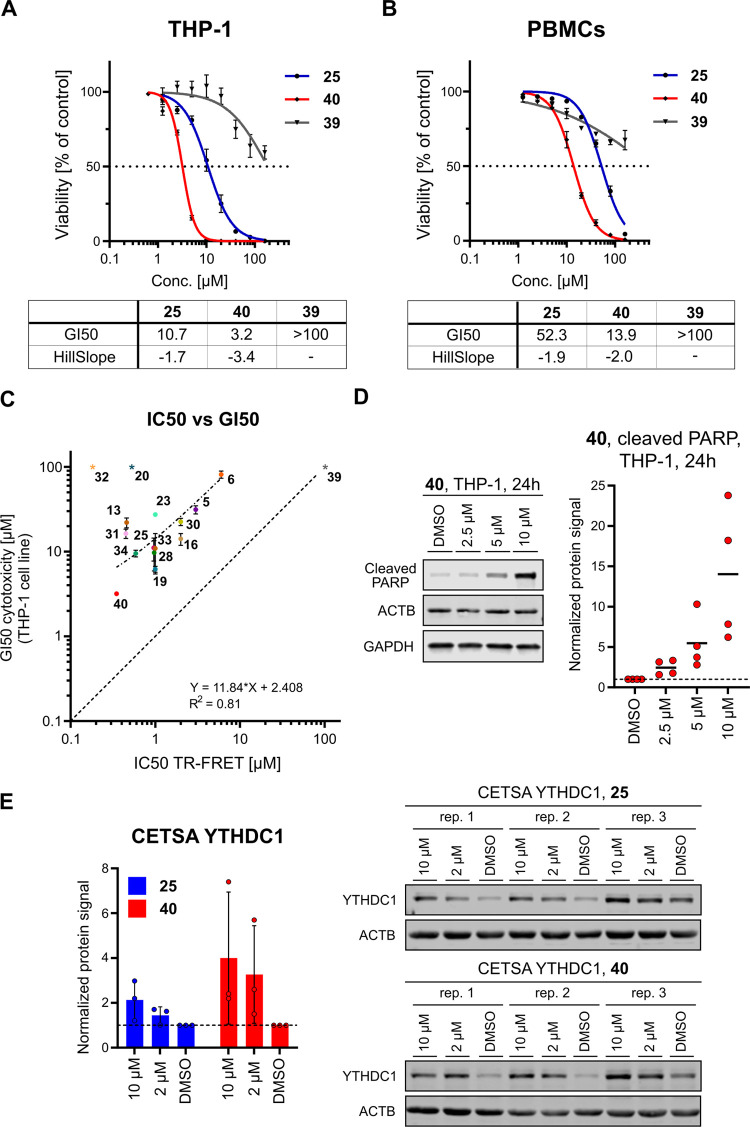
Biological evaluation of compound **40**. (A) Dose–response curves for the antiproliferative effect of compounds **25** and **40** against the THP-1 cell line. Compound **39** is a negative control. (B) Dose–response curves for the antiproliferative effect of compounds **25**, **39**, and **40** against human peripheral blood mononuclear cells (PBMCs). (C) Scatter plot of GI_50_ values for THP-1 vs biochemical IC_50_ values for those compounds that were measured in both cellular and biochemical assays. The correlation provides evidence of target engagement in the cell for this series of compounds. Note that the negative control **39** was excluded from the fitting. Compounds **20** and **32** were also excluded from the fitting as they feature a carboxylic acid which hinders the passage through the cell membrane. (D) Induction of apoptosis by compound **40** in THP-1 cell line after 24 h treatment. Apoptosis was monitored by detecting cleaved PARP using a Western blot. The protein signal was normalized to DMSO control. (E) Thermal stabilization of YTHDC1 upon treatment was quantified by CETSA at 48 °C in MOLM-13 cells. Both compounds **25** and **40** stabilize YTHDC1 in a concentration-dependent manner. The dashed line represents the protein level of the DMSO control used for normalization.

As outlined in our strategy, our primary objective was to combine the most promising (LE, GI_50_) substitution pattern optimized on purine heterocycle with the 5-chloropyrazolopyrimidine core. Thus, we decided to obtain compound **40**, which combines 5-chloropyrazolopyrimidine and chlorobenzyl substituent in position 3. As anticipated, X-ray analysis confirmed the interactions between compound **40** and YTHDC1, which are the same as observed with the purine analogue **25**, along with an additional hydrogen bond with Asp476 (Figure 3A). Furthermore, compound **40** exhibits an IC_50_ of 0.35 (Figure 3B), good LE (0.44), and antiproliferative activity against THP-1 (GI_50_ = 3.2 μM), MOLM-13 (5.6 μM) and NOMO-1 (8.2 μM) (Figures 4A and S2A). We further confirmed the binding of compound **40** to YTHDC1 by thermal shift assay (TSA), where the protein exhibited a thermal stabilization upon binding of pyrazolopyrimidine **40**, increasing the melting temperature by 12 °C at a compound concentration of 100 μM (Figure 3C). Moreover, an equilibrium dissociation constant (*K*_D_) of 49 nM was measured by ITC for compound **40** and YTHDC1 (Figure 3D).

Next, we tested the selectivity of **40** against YTHDF1–3. Compound **40** displayed an IC_50_ value of 89, 60, and 83 μM against YTHDF1, YTHDF2, and YTHDF3, respectively (Figure S2) which is about 200-fold difference in binding preference toward YTHDC1. To provide further evidence of the selectivity of compound **40**, its activity was evaluated against a panel of 58 protein kinases. At a concentration of 2 μM, compound **40** did not display significant inhibition of kinase activity across the entire panel (Figure S1).

With the evidence of target engagement and selectivity against off-targets in hand, we decided to further evaluate compound **40** by comparing its antiproliferative activity against cancer cell lines with its toxicity toward noncancerous cells. The GI_50_ value of compound **40** for THP-1 cancer (AML) cells is 3.2 μM (Figure 4A), whereas for human peripheral blood mononuclear cells (PBMCs, Figure 4B) and human embryonic kidney cells (HEK293T, Figure S2B), it is 14 μM. The 4-fold difference in activity is modest but acceptable considering the high nanomolar on-target potency of compound **40**. The comparatively low GI_50_ for noncancerous cells may reflect the importance of the YTHDC1 protein under physiological conditions, which is not very well studied beyond embryonic development.^18,32^

Compound **39** (adenine scaffold) features the same substituents as ligand **40** (pyrazolopyrimidine scaffold) with swapped –Cl and –NHCH_3_ groups (Table 3). Thus, we selected it as a negative control considering that it is inactive in the biochemical assay. As expected, the negative control **39** was also inactive in the antiproliferative assay with the THP-1 cells (Figure 4A) and the PBMC cells (Figure 4B).

The correlation between biochemical IC_50_ values measured by the HTRF assay and the GI_50_ values (THP-1) provides indirect evidence for the engagement of the YTHDC1 target in the cell (Figure 4C). To further validate target engagement, we performed the cellular thermal shift assay (CETSA) with compounds **25** and **40** (Figure 4E). The results demonstrated thermal stabilization of YTHDC1 upon ligand binding, confirming target engagement. Additionally, to gain insight into the mechanism of cell death underlying the observed cytotoxicity, we measured the cleavage of poly(ADP-ribose) polymerase (PARP), a recognized marker of apoptosis (Figure 4D). Notably, we observed concentration-dependent increases in the signal from cleaved PARP after 24 h of incubation with compound **40** in the THP-1 cell line. Apoptosis onset following the binding of a small-molecule antagonist (ligand **40**) to the m^6^A pocket of YTHDC1 aligns with earlier observations of the YTHDC1 gene knockout in AML cells.^21^

## Chemistry

**Scheme 1:**
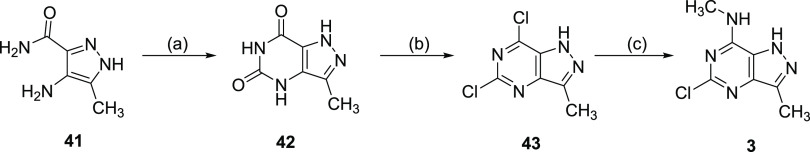
Preparation of Compound **3** Reagents and conditions: (a) Urea, 195 °C, neat (b) POCl_3_, DIPEA, rt. (c) 33% MeNH_2_ in EtOH, EtOH, rt.

During the initial stages of the campaign focused on fragment optimization, we successfully developed a synthetic route to prepare compound **3**. Compounds **1** and **2** had been purchased during the screening phase prior to this campaign.^23^ To synthesize chloropyrazolopyrimidine heterocycle **3**, first, we performed a cyclization reaction of **41** by heating it in the presence of urea forming a condensed ring **42**. The following deoxygenation and chlorination of the pyrimidine ring was achieved by POCl_3_. Finally, we carried out regioselective S_N_Ar using an ethanolic solution of MeNH_2_ (33%), yielding the desired fragment **3** with an overall yield of 9% (Scheme 1).

**Scheme 2:**
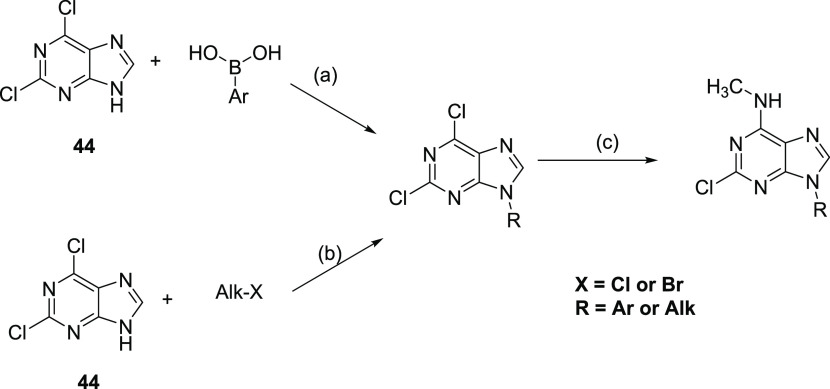
General Synthetic Scheme for N^9^-Substituted Purines Reagents and conditions: (a) Cu(OAc)_2_, 1,10-phenanthroline, DCM, MS 4 Å, rt; (b) K_2_CO_3_, DMF, rt; (c) 33% MeNH_2_ in EtOH, EtOH, rt.

The main synthetic part comprises the preparation of N^9^-substituted-4-methylaminopurines as promising YTHDC1 inhibitors. The strategy was based on the convenient purine N^9^ derivatization followed by a regioselective S_N_Ar. To achieve N^9^ arylations, we used reported Chan-Lam coupling between commercially available 2,6-dichloropurine and boronic acid derivatives.^33^ For the N^9^ alkylation, corresponding alkyl halides were used. During these reactions, we observed a formation of both N^9^ and N^7^ regioisomers. However, the desired N^9^ isomer was formed as the main product and could be easily separated during the purification. The final synthetic step, S_N_Ar, was carried out using MeNH_2_, except for compound **37**, where cyclopropylamine was used instead. If not stated otherwise, all of the final molecules were synthesized according to the General synthetic scheme (Scheme 2). However, compounds **8**, **9**, and **40** were acquired from a commercial supplier.^34^ For the preparation of compound **7**, the General synthetic procedure could not be applied. Instead, the targeted compound was obtained by a two-step reaction process using pTSA-catalyzed reaction of 2,6-dichloropurine **44** and 3,4-dihydropyran followed by nucleophilic aromatic substitution.

**Scheme 3:**
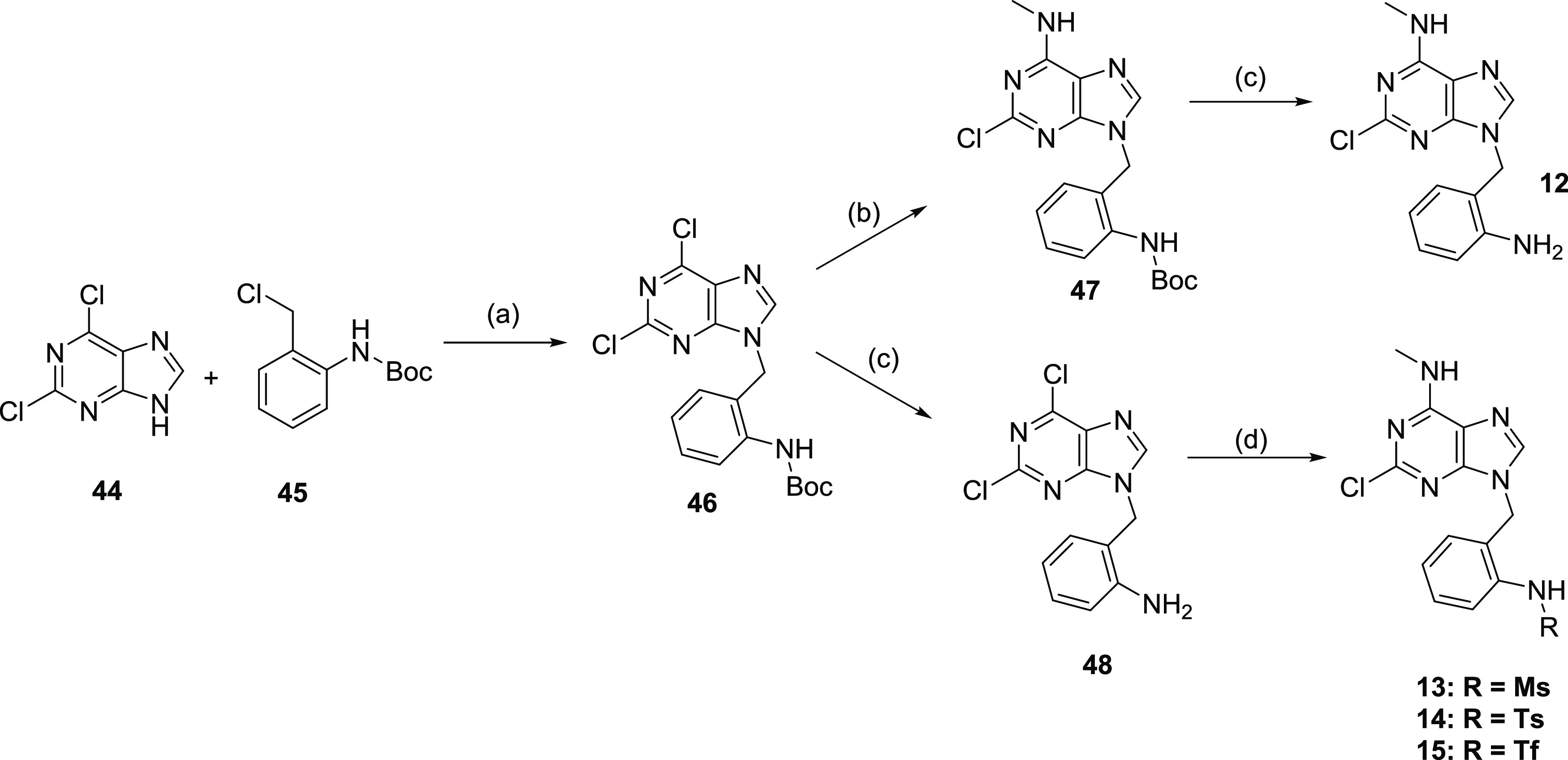
Synthetic Route for Compounds **12, 13, 14**, and **15** Reagents and conditions: (a) K_2_CO_3_, DMF, rt; (b) 33% MeNH_2_ in EtOH, EtOH, rt; (c) TFA, DCM, rt (d) TsCl, MsCl or TFAA, Py, DCM, 0 °C.

Compounds **12**–**15** were prepared from a shared intermediate **46**. The synthesis of **12** followed the reaction sequence outlined in the General synthetic scheme (Scheme 2), with subsequent removal of the Boc protecting group from **47**. The preparation of compounds **13**–**15** involved the initial Boc removal and formation of **48**, followed by free amino group modification using standard conditions for sulfonation and acylation, respectively. The S_N_Ar was carried out as the final step of the reaction sequence (Scheme 3). On the contrary, compound **31**, which also contains a sulfonamide moiety, was prepared according to the General synthetic scheme (Scheme 2). The 2,6-dichloropurine **44** alkylation was carried out with an intermediate already containing the sulfonamide group. Even though, this approach provided the final compound **31** in limited yield (6%, after three steps), the reaction sequence was not further optimized. These findings suggest, that the modified reaction sequence, used for compounds **13**–**15**, shall be preferentially used in combination with other sulfonamide derivatives.

**Scheme 4:**
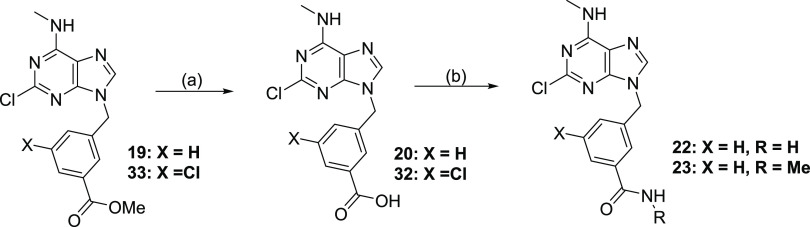
Synthetic Route for the Preparation of Carboxylic Acid Derivatives Reagents and conditions: (a) 37% HCl, dioxane, reflux; (b) RNH_2_, COMU, DIPEA, DMF, 0 °C to rt.

Compounds **20**, **29**, and **32** that contain carboxylic groups, as well as compounds **22**, and **23**, with amide moiety, were synthesized from corresponding alkyl esters **19**, **28**, and **33**, respectively. The methyl ester hydrolysis was achieved by heating the reaction mixture in the presence of 37% HCl for compounds **19**, **28**, **33**. In the case of compound **10**, its *tert*-butyl ester analogue was subjected to hydrolysis with TFA. For the synthesis of compounds **22**, and **23**, a combination of COMU as the coupling agent along with the presence of NH_3_ or MeNH_2_ was used (Scheme 4).

**Scheme 5:**
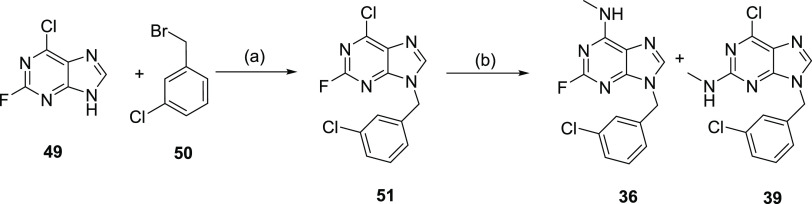
Synthetic Route for Compounds **36** and **39** Reagents and conditions: (a) K_2_CO_3_, DMF, rt; (b) 33% MeNH_2_ in EtOH, EtOH, rt.

Lastly, due to the nature of fluorine substituent, the nucleophilic aromatic substitution of **51** was not regioselective. As a result, this reaction led to the formation of two products **36** and **39** that were isolated and tested against YTHDC1 (Scheme 5).

## Conclusions

In summary, we used structure-based design to develop a small-molecule inhibitor targeting YTHDC1. Through step-by-step ligand optimization and leveraging the most potent substituents identified on a purine heterocycle, we successfully combined the structural features together with the 5-chloropyrazolopyrimidine scaffold. Supporting the campaign with high-resolution X-ray data, we evaluated the efficacy of the most potent compound **40** using TSA and ITC assays. Compound **40** exhibits a *K*_D_ value of 49 nM, very good LE and LLE values, and antiproliferative activity against acute myeloid leukemia cell lines (THP-1, MOLM-13, NOMO-1). Compound **40** is selective against the cytoplasmic YTHDF1–3 readers according to two different assays (HTRF assay, TSA) and against YTHDC2 according to TSA. Furthermore, compound **40** is inactive in a panel of 58 human protein kinases. The correlations between the biochemical SAR (HTRF IC_50_ values) and cellular SAR (THP-1 GI_50_ values) for the series that led to ligand **40**, together with CETSA validation, provide evidence of target engagement in the cell. Thus, we propose compound **40** as a small-molecule tool to study the role of the YTHDC1 m^6^A-reader in AML.

## Materials and Methods

### HTRF Assay (IC_50_)

GST-YTHDC1, GST-YTHDF1, GST-YTHDF2, and GST-YTHDF3 were purified as previously reported.^35^ The HTRF assay was assembled as detailed in ref (23) with the only difference being that the starting concentration of the dose–response experiments used for the IC_50_ determination was variated dependently from the tested compound. The same protocol applies to the four proteins. The competitive inhibition data of GST-YTHDC1 in the presence of the compounds were normalized using a blank assembled with all of the components of the assay, including DMSO, except for GST-YTHDC1. The competitive inhibition data of GST-YTHDF1, GST-YTHDF2, and GST-YTHDF3 in the presence of compound **40** were normalized using a blank assembled with all of the components of the assay, minus the protein, and 2-fold serial dilutions of compound **40**. This measure was adopted to mitigate the interference arising from high concentrations of compound **40**. In all cases, the signal was measured using a Spark plate reader (Tecan), with a 320 nm excitation filter and 620 nm (measurement 1) or 665 (measurement 2) emission filters, a dichroic 510 mirror, 75 flashes, and applying a lag time of 100 μs and an integration time of 400 μs.

### Thermal Shift Assay

The YTH domain of YTHDF1 (residues 361–559), YTHDF2 (residues 383–579), YTHDF3 (residues 387–585), YTHDC1 (residues 345–509), and YTHDC2 (residues 1285–1424) was cloned into pET-based vector harboring N-terminal hexa-histidine tag and a TEV cleavage site. All recombinant proteins were overexpressed at 20 °C in *Escherichia coli* BL21 (DE3) upon induction with 0.4 mM IPTG and purified by HiTrap nickel column. The His tag was cleaved by the addition of TEV protease (1:100) to the purified recombinant protein while dialysis to remove imidazole at 4 °C overnight. The samples were then passed through nickel column and further purified by size exclusion chromatography. YTHDC1, YTHDF1, YTHDF2, YTHDF3, and YTHDC2 were buffered in 50 mM HEPES pH 7.5, 150 mM NaCl, and tested in a white 96-well plate at a final concentration of 2 μM. SYPRO Orange dye (Sigma-Aldrich, S5692) was added to the mix with a final concentration of 3.75×. Compound **40** was also added to the mix and tested as a set of 2-fold dilutions. The fluorescence monitoring was performed using a LightCycler 480 System. The temperature was set up to increase with a ramp rate of 0.06 °C/s from 20 to 85 °C and 10 acquisitions per °C were taken in dynamic integration time mode and using red 610 (498–610) filter combination. The melting curves were calculated using the *T*_m_ calling analysis of the LightCycler 480 software release 1.5.1.62 SP3.

### Isothermal Titration Calorimetry

The Isothermal titration calorimetry (ITC) experiment was carried out at 18 °C using MicroCal ITC200 (GE Healthcare). Protein and compound were dissolved in 20 mM Tris pH 7.4, 150 mM NaCl along with 0.2% DMSO. Protein at the concentration of 100 μM was titrated into the sample cell containing 10 μM compound. After an initial injection of 1 μL, 13 injections of 3.0 μL each were performed. The raw data were integrated and analyzed using a single-binding site model, provided in the MicroCal Origin software package.^36^

### Crystallography

The crystals of YTHDC1 YTH domain were obtained by mixing 1 μL protein solution at 10 mg/mL with mother liquor containing 0.1 M Bis-Tris at pH 6.5, 0.2 M ammonium sulfate and 25% PEG 3350 at 22 °C in a hanging drop vapor diffusion setup. To obtain crystals of protein complexed with fragments, the crystals were transferred to a 1 μL drop containing 50–200 mM (depending on the solubility) fragment directly dissolved in 0.1 M Bis-Tris at pH 6.5, 0.2 M ammonium sulfate, and 30% PEG 3350, soaked overnight at 22 °C, harvested, and frozen in liquid nitrogen without additional cryoprotection. Diffraction data were collected at the Swiss Light Source (Villigen, Switzerland) using the beamline X06DA (PXIII) and processed using XDS.^37^ The structures were solved by molecular replacement using Phaser program^38^ from the Phenix package.^39^ The unliganded structure of YTHDC1 (PDB ID: 4R3H) was used as a search model. The model building and refinements were performed using COOT^40^ and phenix.refine.^41^

### Cell Culture

THP-1, MOLM-13, and NOMO-1 cell lines were obtained from DSMZ-German Collection of Microorganisms and Cell Cultures GmbH. Cells were cultured in RPMI 1640 medium (11875093, Thermo Fisher Scientific) containing 10% FBS (16140071, Thermo Fisher Scientific) and 1% penicillin-streptomycin (15140122, Thermo Fisher Scientific) in 5% CO_2_ at 37 °C in a humidified incubator. Cell lines were tested negative for mycoplasma contamination (PCR-based assay by Microsynth, Switzerland). Peripheral blood mononuclear cells (PBMCs) were isolated using Ficoll Paque Plus (17–1440–02, Cytiva) density centrifugation according to the manufacturer’s instructions. Human blood for PBMCs isolation was obtained from the Blood Donation Center Zurich (Ethics Committee approval number: 2021–00024).

### Cytotoxicity (GI_50_)

Cells were seeded in white clear-bottom 96-well plates at a density of 6 × 10^3^ cells/well in 50 μL of the complete RPMI medium and treated with 50 μL of increasing concentrations of the indicated compounds dissolved in DMSO (final concentration of compounds 0.6–160 μM) or DMSO only (0.5% (v/v)) as a negative control and incubated for 72 h at 37 °C with 5% CO2. Cell viability was determined using a CellTiter-Glo luminescent cell viability assay (Promega) based on the detection of ATP according to the manufacturer’s instructions. 100 μL of the reagent was added to each well and incubated for 10 min at room temperature on an orbital shaker. The luminescence was recorded using a Tecan Infinite 3046 M1000 microplate reader from the top. Background luminescence value was obtained from wells containing the CellTiter-Glo reagent and medium without cells. Cell viability curves were plotted in GraphPad Prism 9 and fitted with nonlinear regression, from which GI_50_ values were determined. The assay was carried out with two technical replicates for each concentration and repeated three to four times on different days. PBMCs were seeded in 96-well plates in technical triplicates at a density of 1 × 10^5^ cells/well in 100 μL of complete RPMI medium and repeated three times. Cell viability was determined using a CellTiter-Glo luminescent cell viability assay (Promega).

### Cellular Thermal Shift Assay (CETSA)

One million MOLM-13 cells were suspended in 100 μL of PBS (10010023, Thermo Fisher Scientific) supplemented with a 2× protease inhibitor cocktail (11697498001, Roche) for each experimental condition. The cells were incubated with compounds or DMSO control (1% (v/v)) for 1 h at 37 °C. Samples were then heated up to 48 °C for 3 min followed by cooling to room temperature. Next, samples were lysed by three freeze–thaw cycles in liquid nitrogen and centrifuged at 16,000*g* for 30 min, 4 °C. Equal volumes of control and tested samples (12 μL) were analyzed by Western blot. The protein signal was quantified by densitometry in Image Studio Lite software. The amount of YTHDC1 (ab259990, Abcam, 1:1000) protein was first normalized to β-actin (ab8226, Abcam, 1:2000) and then to DMSO control and analyzed in GraphPad Prism 9.

### Apoptosis Induction by Western Blot

THP-1 cells were seeded into 6-well plates at a density of 1 × 10^6^ cells/mL in 1 mL of complete RPMI media. The cells were treated with indicated concentrations of Compound 40 or DMSO control (0.1% (v/v)). After 24 h, cells were collected by centrifugation, washed twice with PBS, and resuspended in 50 μL of RIPA buffer (89900, Thermo Fisher Scientific) with added 2× protease inhibitor cocktail (11697498001, Roche). Cell lysates were centrifuged for 30 min at 16,000*g* at 4 °C, and the supernatant was collected. The protein concentration was quantified with Micro BCA Protein Assay Kit (23235, Thermo Fisher Scientific) and 20 μg of protein was loaded per well on a 10% Tris-Glycine polyacrylamide gel. Following electrophoresis, the proteins were transferred to a nitrocellulose membrane, blocked with 5% nonfat milk, 0.5% BSA in TBST buffer, and incubated with cleaved PARP (5625, Cell Signaling, 1:1000), β-actin (ab8226, Abcam, 1:2000), and GAPDH (2118S, Cell Signaling, 1:4000) antibodies overnight at 4 °C. For the detection, IRDye 800CW goat anti-rabbit IgG and IRDye680RD donkey anti-mouse IgG secondary antibodies (1:10,000) were used. Fluorescence signal was detected on Odyssey CLx Imaging System (LI-COR). The band intensity in each lane was quantified using Image Studio LiteVersion 5.2.5 (LI-COR) and analyzed in GraphPad Prism 9.

### Kinases Selectivity

For kinase inhibition testing, the Diversity Kinase [10 uM ATP] KinaseProfiler LeadHunter Panel—FR (50–015 KP10) was performed by Eurofins Discovery. Compound **40** was tested at a single concentration of 2 μM, in two replicates, against a panel of 58 protein kinases at an ATP concentration of 10 μM.

### Chemistry

All reagents were purchased from commercial suppliers and used as received. Reactions run at elevated temperatures were carried out in an oil bath. Our research group successfully synthesized all of the compounds as described, except for compounds **1, 2a, 2b, 8, 9**, and **40** which were obtained from a commercial supplier.^34^ All compounds have >95% purity (HPLC). All reactions were monitored by thin-layer chromatography (Aluminum plates coated with silica gel 60 F_254_). Flash column chromatography was carried out over silica gel (0.040–0.063 mm). ^1^H and ^13^C {^1^H} NMR spectra were recorded on AV2 400 MHz and AV600 Bruker spectrometers (400, 101, and 600, 150 MHz, respectively) in DMSO or CDCl_3_ Chemical shifts are given in ppm and their calibration was performed to the residual ^1^H and ^13^C signals of the deuterated solvents. Multiplicities are abbreviated as follows: singlet (s), doublet (d) multiplet (m), and broad signal (bs). The purity was acquired by Liquid chromatography high resolution electrospray ionization mass spectrometry (LC-HR- ESI-MS): Acquity UPLC (Waters, Milford) connected to an Acquity eλ diode array detector and a Synapt G2 HR-ESI-QTOF-MS (Waters, Milford); injection of 1 μL sample (*c* = ca. 10–100 μg mL^–1^ in the indicated solvent); Acquity BEH C18 HPLC column (1.7 μm particle size, 2 mm × 50 mm, Waters) kept at 30 °C elution at a flow rate of 400 μL/min with A: H_2_O + 0.02% TFA and B: CH_3_CN + 0.02% TFA, linear gradient from 10 to 95% B within 3 min, then isocratic 95% B for 2 min; UV spectra recorded from 190 to 300 at 1.2 nm resolution and 20 points s-1UV spectra recorded from 200 to 600 at 1.2 nm resolution and 20 points s^–1^; ESI: positive ionization mode, capillary voltage 3.0 kV, sampling cone 40 V, extraction cone 4 V, N2 cone gas 4 L h^–1^, N2 desolvation gas 800 L min^–1^, source temperature 120 °C; mass analyzer in resolution mode: mass range 100–2000 *m*/*z* with a scan rate of 1 Hz; mass calibration to <2 ppm within 50–2500 *m*/*z* with a 5 mM aq. soln. of HCO_2_Na, lockmasses: *m*/*z* 195.0882 (caffein, 0.7 ng mL^–1^) and 556.2771(Leucine-enkephalin, 2 ng mL^–1^).

#### 5-Chloro-*N*,3-dimethyl-1*H*-pyrazolo[4,3-*d*]pyrimidin-7-amine (**3**)

To a powder of 4-amino-3-methyl-1*H*-pyrazole-5-carboxamide **41** (0.13 g, 0.97 mmol), which was prepared following the reported procedure,^42^ was added urea (389 mg, 6.4 mmol). The neat reaction mixture was heated and stirred at 195 °C for 5 h. Upon the temperature increase, the solid reactants melted and after the product formation, the reaction mixture solidified. The reaction vessel was cooled to rt and the crude product **42** was used in the next step without further purification.

The pyrazolo[4,3-*d*]pyrimidine-5,7(6*H*)-dione **42** was suspended in POCl_3_ (4.6 mL) followed by the addition of DIPEA (0.403 mL, 2.3 mmol). The reaction mixture was heated at 70 °C for 14 h. The volatiles were removed *in vacuo* and the residue was poured over ice. The mixture was extracted into EtOAc (3 × 6 mL) and the combined organic layers were dried over MgSO_4_ and filtered. Activated charcoal was added to the filtrate, and the mixture was stirred for 10 min. After the charcoal removal (filtration paper), the solvent was removed under reduced pressure. The crude product **43** was dissolved in EtOH and 33% MeNH_2_ in EtOH (0.2 mL) was added into the reaction vessel. The reaction mixture was stirred at rt for 1 h and after the reaction completion (TLC), the volatiles were removed *in vacuo*. The crude product **3** was purified using flash column chromatography (SiO_2_; EtOAc/MeOH = 10:1) and the desired compound was obtained as a white solid (0.018 g, 9% after three steps). ^1^H NMR (400 MHz, MeOH - *d*^4^) δ 3.12 (s, 3H), 2.49 (s, 3H). ^13^C NMR (126 MHz, MeOH - *d*^4^) δ 155.8, 154.2, 138.6, 136.7, 128.3, 27.8, 9.3. LRMS (ESI) *m*/*z*: [M + H]^+^ calcd for C_7_H_9_ClN_5_; 198.054 found, 198.019.

#### General Procedure 1 (N^9^-Alkylation of Purines)

2,6-Dichloropurine **44** (1 equiv) was dissolved in DMF (0.5 M) and K_2_CO_3_ (2 equiv) was added. Corresponding alkyl halide was subsequently added to the reaction mixture (1 equiv). The resulting reaction mixture was stirred at rt until reaction completion (Monitored by TLC). The reaction mixture was quenched by the addition of water and extracted into EtOAc. Combined organic layers were washed by 10% aq. sol. of LiCl, dried over MgSO_4_, filtrated, and evaporated.

#### General Procedure 2 (Regioselective S_N_Ar with R-NH_2_)

9-Alkyl-2,6-dichloropurine (1 mmol) was suspended in EtOH (0.5 M) and 33% MeNH_2_ in EtOH (2 mL) and stirred at rt. After the reaction completion (TLC), the volatiles were removed *in vacuo.* The crude product was purified using flash column chromatography.

##### 2-Chloro-*N*-methyl-9-phenyl-9*H*-purin-6-amine (**4**)

The final compound was prepared following the General method 2 from corresponding 9-phenyl-2,6-dichloro-9*H*-purine (0.02 g, 0.075 mmol) that was prepared according to the reported Chan-Lam coupling procedure.^33^ The crude product was purified using flash column chromatography (SiO_2_; EtOAc/Hept = 1.2:1) and the desired compound was obtained as a white solid (0.017 g, 87%). ^1^H NMR (400 MHz, CDCl_3_ - *d*) δ 7.98 (s, 1H), 7.67–7.64 (m, 2H), 7.57–7.53 (m, 2H), 7.46–7.42 (m, 1H), 6.05–5.93 (bs, 1H), 3.22 (s, 3H); ^13^C NMR (101 MHz, CDCl_3_ - *d*) δ 156.4, 155.5, 149.7, 139.3, 134.6, 130.1, 128.4, 123.6, 119.6, 27.9. LRMS (ESI) *m*/*z*: [M + H]^+^ calcd for C_12_H_11_ClN_5_; 260.070 found, 260.070.

##### 9-Benzyl-2-chloro-*N*-methyl-9*H*-purin-6-amine (**5**)

The N^9^ alkylation was performed following General procedure 1 using 2,6-dichloropurine **44** (0.2 g, 1.06 mmol) and BnBr (0.126 mL, 1.06 mmol). The crude product was purified using flash column chromatography (SiO_2_; EtOAc/Hept = 1.2:2) and the desired compound was obtained as a white solid (0.160 g, 54%). ^1^H NMR (400 MHz, CDCl_3_ - *d*) δ 8.05 (s, 1H), 7.43–7.36 (m, 3H), 7.31 (m, 2H), 5.41 (s, 2H); ^13^C NMR (101 MHz, CDCl_3_ - *d*) δ 153.4, 153.3, 152.1, 145.7, 134.1, 130.8, 129.6, 129.3, 128.3, 48.2.

The final compound was prepared following the General method 2 from corresponding 9-benzyl-2,6-dichloro-9*H*-purine (0.08 g, 0.286 mmol). The crude product was purified using flash column chromatography (SiO_2_; EtOAc/Hept = 2.5:1 → 4:1) and the desired compound was obtained as a white solid (0.06 g, 76%). ^1^H NMR (400 MHz, CDCl_3_ - *d*) δ 7.63 (bs, 1H), 7.38–7.32 (m, 3H), 7.31–7.28 (m, 2H), 5.90–5.88 (bs, 1H), 5.32 (s, 2H), 3.19 (bs, 3H); ^13^C NMR (101 MHz, CDCl_3_ - *d*) δ 156.1, 154.9, 150.1, 139.8, 135.3, 129.1, 128.5, 128.0, 118.7, 47.2, 29.7. LRMS (ESI) *m*/*z*: [M + H]^+^ calcd for C_13_H_13_ClN_5_; 274.085 found, 274.085.

##### 2-Chloro-*N*-methyl-9-(pyridin-4-ylmethyl)-9*H*-purin-6-amine (**6**)

The N^9^ alkylation was performed following General procedure 1 using 2,6-dichloropurine **44** (0.2 g, 1.06 mmol) and 4-bromoethylpyridine hydrobromide (0.267 g, 1.06 mmol). The crude product was purified using flash column chromatography (SiO_2_; EtOAc/MeOH = 2:0.2) and the desired compound was obtained as a white solid (0.094 g, 32%). ^1^H NMR (400 MHz, CDCl_3_ - *d*) δ 8.64–8.62 (m, 2H), 8.11 (s, 1H), 7.08 (d, *J* = 6.0 Hz, 2H), 5.45 (s, 2H). ^13^C NMR (101 MHz, CDCl_3_ - *d*) δ 153.7, 153.3, 152.5, 151.0, 145.4, 143.0, 130.8, 122.2, 46.8.

The final compound **6** was prepared following the General method 2 from corresponding 9-alkyl-2,6-dichloro-9*H*-purine (0.069 g, 0.246 mmol). The crude product was purified using flash column chromatography (SiO_2_; EtOAc/MeOH = 2:0.2 → 2:0.8) and the desired compound was obtained as a white solid (0.045 g, 66%). ^1^H NMR (400 MHz, DMSO – *d*^6^) δ 8.53–8.52 (m, 2H), 8.29–8.27 (m, 1H), 8.26 (s, 1H), 7.16–7.15 (m, 2H), 5.42 (s, 2H), 2.93 (d, *J* = 4.6 Hz, 3H); ^13^C NMR (101 MHz, DMSO – *d*^6^) δ 155.6, 153.5, 150.0, 149.5, 145.5, 141.3, 121.8, 118.3, 45.2, 27.2. LRMS (ESI) *m*/*z*: [M + H]^+^ calcd for C_12_H_12_ClN_6_; 275.081 found, 275.081.

##### 2-Chloro-*N*-methyl-9-(tetrahydro-2*H*-pyran-2-yl)-9*H*-purin-6-amine (**7**)

The final compound **7** was prepared following the General method 2 from corresponding 9-alkyl-2,6-dichloro-9*H*-purine (0.2 g, 0.073 mmol) that was prepared following the reported procedure.^43^ The crude product was purified using flash column chromatography (SiO_2_; EtOAc/Hept = 2:1) and the desired compound was obtained as a white solid (0.165 g, 84%). ^1^H NMR (400 MHz, DMSO – *d*^6^) δ 8.35 (s, 1H), 8.25–8.22 (m, 1H), 5.56 (dd, *J* = 11.0, 2.2 Hz, 1H), 4.03–3.96 (m, 1H), 3.75–3.61 (m, 1H), 2.92 (d, *J* = 4.6 Hz, 3H), 2.37–2.18 (m, 1H), 2.08–1.92 (m, 2H), 1.86–1.72 (m, 1H), 1.68–1.56 (m, 2H). ^13^C NMR (101 MHz, DMSO – *d*^6^) δ 155.5, 153.4, 148.9, 139.2, 118.2, 80.9, 67.7, 30.0, 27.2, 24.5, 22.3. LRMS (ESI) *m*/*z*: [M + H]^+^ calcd for C_11_H_15_ClN_5_O; 268.096 found, 268.097.

##### 3-(2-Chloro-6-(methylamino)-9*H*-purin-9-yl)benzoic acid (**10**)

The final compound **10** was prepared following the General method 2 from corresponding *tert*-butyl 3-(2,6-dichloro-9*H*-purin-9-yl)benzoate (0.02 g, 0.055 mmol) that was prepared following reported Chan-Lam coupling procedure.^33^ The crude product was purified using flash column chromatography (SiO_2_; EtOAc/Hept = 2:1). and the desired compound was obtained as a white solid (0.018 g, 91%). ^1^H NMR (400 MHz CDCl_3_ - *d*) δ 8.21 (t, *J* = 1.9 Hz, 1H), 8.07–8.02 (m, 2H), 7.93 (dd, *J* = 8.1, 1.2 Hz, 1H), 7.61 (t, *J* = 7.9 Hz, 1H), 6.14–6.03 (bs, 1H), 3.29–3.15 (bs, 3H), 1.62 (s, 9H). ^13^C NMR (101 MHz, CDCl_3_ - *d*) δ 164.6, 156.4, 155.6, 139.0, 134.8, 134.0, 130.0, 129.2, 127.5, 124.1, 119.7, 82.1, 77.4, 28.3.

The *tert*-butyl ester (0.014, 0.039 mmol) was dissolved in DCM (0.5 mL) and TFA was added (0.02 mL). The reaction was stirred at rt overnight and neutralized with DIPEA. The volatiles were evaporated *in vacuo* and the crude product was recrystallized from H_2_O. The final product was obtained as a white solid (0.011 g, 93%). ^1^H NMR (400 MHz, DMSO – *d*^6^) δ 8.64 (s, 1H), 8.41–8.37 (m, 1H), 8.35–8.34 (m, 1H), 8.07–8.01 (m, 2H), 7.76–7.72 (m, 1H), 2.96 (d, *J* = 4.5 Hz, 3H); ^13^C NMR (101 MHz, DMSO – *d*^6^) δ 166.5, 155.8, 153.9, 149.0, 140.1, 134.9, 132.3, 130.0, 128.5, 127.6, 123.9, 118.9, 27.3.LRMS (ESI) *m*/*z*: [M + H]^+^ calcd for C_13_H_11_ClN_5_O_2_; 304.057 found, 304.039.

##### 2-(2-Chloro-6-(methylamino)-9*H*-purin-9-yl)-*N*-phenylacetamide (**11**)

The N^9^ alkylation was performed following General procedure 1 using 2,6-dichloropurine **44** (0.2 g, 1.06 mmol) and *N-*phenylchloroacetamide (1.0 g, 5.9 mmol), which was prepared following the reported procedure.^44^ The crude product was purified using flash column chromatography (SiO_2_; EtOAc/Hept = 2:1 to 4:1) and the desired compound was obtained as a white solid (0.54 g, 28%). ^1^H NMR (400 MHz, DMSO – *d*^6^) δ 10.53 (s, 1H), 8.88 (s, 1H), 7.57–7.54 (m, 2H), 7.35–7.31 (m, 2H), 7.11–7.09 (m, 1H), 5.42 (s, 1H). ^13^C NMR (101 MHz, DMSO – *d*^6^) δ 164.8, 163.1, 153.5, 151.0, 143.4, 138.3, 128.9, 123.9, 122.4, 119.3, 49.4.

The final compound **11** was prepared following the General method 2 from corresponding 9-alkyl-2,6-dichloro-9*H*-purine (0.05 g, 0.155 mmol). The crude product was purified using flash column chromatography (SiO_2_; EtOAc/Hept = 4:1 to 10:1) and the desired compound was obtained as a white solid (0.038 g, 77%). ^1^H NMR (400 MHz, DMSO – *d*^6^) δ 10.45 (bs, 1H), 8.23–8.22 (m, 1H), 8.13 (s, 1H), 7.59 (d, *J* = 7.3 Hz, 2H), 7.35–7.31 (m, 2H), 7.08 (t, *J* = 7.4 Hz, 1H), 5.05 (s, 2H), 2.94 (d, *J* = 4.6 Hz, 3H). ^13^C NMR (101 MHz, DMSO – *d*^6^) δ 164.9, 155.5, 153.3, 149.8, 142.2, 138.5, 128.9, 123.7, 119.1, 117.9, 45.7, 27.2. LRMS (ESI) *m*/*z*: [M + H]^+^ calcd for C_14_H_14_ClN_6_O; 317.091 found, 317.092.

##### 9-(2-Aminobenzyl)-2-chloro-*N*-methyl-9*H*-purin-6-amine (**12**)

The N^9^ alkylation was performed following General procedure 1 using 2,6-dichloropurine **44** (0.531 g, 2.81 mmol) and *tert*-butyl (2-(chloromethyl)phenyl)carbamate **45** that was prepared following the reported procedure^45^ (0.68 g, 2.81 mmol). The crude product **46** was purified using flash column chromatography (SiO_2_; EtOAc/Hept = 1:1) and the desired compound was obtained as a white solid (0.346 g, 31%). ^1^H NMR (400 MHz, DMSO – *d*^6^) δ 8.93 (s, 1H), 8.62 (s, 1H), 7.33–7.28 (m, 2H), 7.15–7.10 (m, 1H), 7.08 (d, *J* = 7.0 Hz, 1H), 5.48 (s, 2H), 1.41 (s, 9H); ^13^C NMR (101 MHz, DMSO – *d*^6^) δ 154.1, 154.0, 151.5, 150.2, 148.9, 136.5, 130.8, 130.2, 129.1, 129.0, 126.6, 125.9, 79.6, 44.4, 28.5.

The next step was performed using the General method 2 from corresponding 9-alkyl-2,6-dichloro-9H-purine **46** (0.15 g, 0.38 mmol). The crude product **47** was purified using flash column chromatography (SiO_2_; EtOAc/Hept = 2:1) and the desired compound was obtained as a white solid (0.121 g, 81%). ^1^H NMR (400 MHz, DMSO – *d*^6^) 9.06 (s, 1H), 8.28–8.22 (m, 1H), 8.11 (s, 1H), 7.38 (d, *J* = 8.7 Hz, 1H), 7.29 (td, *J* = 7.6, 1.6 Hz, 1H), 7.11 (td, *J* = 7.5, 1.4 Hz, 1H), 7.00 (d, *J* = 6.8 Hz, 1H), 5.32 (s, 2H), 2.92 (d, *J* = 4.6 Hz, 3H), 1.46 (s, 9H). ^13^C NMR (101 MHz, DMSO – *d*^6^) δ 155.6, 153.6, 153.3, 149.5, 141.1, 136.0, 130.1, 128.4, 125.6, 125.2, 118.1, 79.2, 43.0, 28.1, 27.3.

The final compound **12** was prepared by deprotection of **47** (0.05 g, 0.128 mmol) in DCM (0.6 mL) using TFA (0.097 mL, 1.28 mmol). The reaction mixture was stirred at room temperature for 6 h. The reaction was quenched by aq. sol. of Na_2_CO_3_ and extracted into EtOAc. Combined organic layers were dried over MgSO_4_. The volatiles were removed *in vacuo* and the crude product was purified using flash column chromatography (SiO_2_; EtOAc/MeOH = 2:1 → 10:1) and the desired compound was obtained as a brownish solid (0.024 g, 64%). ^1^H NMR (400 MHz, DMSO – *d*^6^) δ 8.26–8.21 (m, 1H), 8.13 (s, 1H), 7.01 (td, *J* = 7.6, 1.6 Hz, 1H), 6.78 (dd, *J* = 7.6, 1.6 Hz, 1H), 6.69 (dd, *J* = 8.0, 1.2 Hz, 1H), 6.51 (td, *J* = 7.4, 1.2 Hz, 1H), 5.16 (s, 2H), 2.92 (d, *J* = 4.6 Hz, 3H). ^13^C NMR (101 MHz, DMSO – *d*^6^) δ 155.54, 153.23, 149.44, 145.93, 141.10, 128.80, 128.62, 119.68, 118.11, 116.53, 115.46, 43.09, 27.18. LRMS (ESI) *m*/*z*: [M + H]^+^ calcd for C_13_H_14_ClN_6_; 289.096 found, 289.096.

##### *N*-(2-((2-Chloro-6-(methylamino)-9*H*-purin-9-yl)methyl)phenyl)methanesulfonamide (**13**)

The compound **46** (0.47 g, 1.19 mmol) was dissolved in DCM (5 mL) followed by the addition of TFA (0.45 mL). The reaction mixture was stirred at rt for 16 h. After the reaction completion, the reaction was quenched by aq. sol. Na_2_CO_3_ and extracted into DCM. Combined organic layers were dried over MgSO_4_ and the volatiles were removed *in vacuo.* The crude product was purified using flash column chromatography (SiO_2_; EtOAc/Hept = 1:1) and the intermediate **48** was obtained as a white solid (0.346 g, 31%). ^1^H NMR (400 MHz, DMSO – *d*^6^) δ 8.93 (s, 1H), 8.62 (s, 1H), 7.33–7.28 (m, 2H), 7.15–7.10 (m, 1H), 7.08 (d, *J* = 7.0 Hz, 1H), 5.48 (s, 2H), 1.41 (s, 9H); ^13^C NMR (101 MHz, DMSO – *d*^6^) δ 154.1, 154.0, 151.5, 150.2, 148.9, 136.5, 130.8, 130.2, 129.1, 129.0, 126.6, 125.9, 79.6, 44.4, 28.5.

To a solution of compound **48** (0.067 g, 0.227 mmol) in DCM (2.2 mL) was added pyridine (0.024 mL, 0.295 mmol). The solution was cooled down to 0 °C and MsCl (0.021 mL, 0.274 mmol) was added dropwise after 30 min. After the reaction completion (TLC), the volatiles were removed *in vacuo*. The crude product was subsequently used in the next step without further purification. The final product **13** was prepared following the general method for S_N_Ar. The crude product was purified using flash column chromatography (SiO_2_; EtOAc/MeOH = 4:1 → 4:0) and the desired compound was obtained as a white solid (0.025 g, 30% after two steps). ^1^H NMR (400 MHz, DMSO – *d*^6^) δ 9.50 (s, 1H), 8.31- 8.25 (m, 1H), 8.18 (s, 1H), 7.40 (dd, *J* = 7.9, 1.5 Hz, 1H), 7.35 (td, *J* = 7.5, 1.5 Hz, 1H), 7.22 (td, *J* = 7.4, 1.5 Hz, 1H), 6.87 (dd, *J* = 7.8, 1.5 Hz, 1H), 5.45 (s, 2H), 3.09 (s, 3H), 2.92 (d, *J* = 4.6 Hz, 2H); ^13^C NMR (101 MHz, DMSO – *d*^6^) δ 155.6, 153.3, 149.6, 141.4, 134.8, 133.2, 128.7, 128.0, 127.0, 126.8, 118.2, 42.9, 27.2. LRMS (ESI) *m*/*z*: [M + H]^+^ calcd for C_12_H_12_N_5_; 367.073 found, 367.075.

##### *N*-(2-((2-Chloro-6-(methylamino)-9*H*-purin-9-yl)methyl)phenyl)-4-methylbenzenesulfonamide (**14**)

To a solution of Compound **48** (0.1 g, 0.339 mmol) in DCM (2 mL) was added pyridine (0.032 mL, 0.407 mmol). The solution was cooled down to 0 °C and TsCl (0.064 mL, 0.339 mmol) was added portion-wise after 30 min. After the reaction completion (TLC), the volatiles were removed *in vacuo*. The crude product was subsequently used in the next step without further purification. The final product **14** was prepared following the general method for S_N_Ar. The crude product was purified using flash column chromatography (SiO_2_; EtOAc/Hept = 2:1) and the desired compound was obtained as a white solid (0.078 g, 52% after two steps). ^1^H NMR (400 MHz, CDCl_3_ - *d*) 7.74 (d, *J* = 8.0 Hz, 2H), 7.72–7.68 (bs, 1H), 7.29–7.21 (m, 4H), 7.18–7.10 (m, 1H), 6.27–6.13 (bs, 1H), 4.93 (s, 2H), 3.20–3.03 (bs, 3H), 2.40 (s, 3H). ^13^C NMR (101 MHz, CDCl_3_ - *d*) δ 156.0, 154.7, 149.0, 143.5, 139.4, 138.2, 135.9, 130.7, 130.5, 130.2, 129.8, 127.5, 127.2, 1276.0, 118.7, 44.2, 27.8, 21.7. LRMS (ESI) *m*/*z*: [M + H]^+^ calcd for C_20_H_20_ClN_6_O_2_S; 443.105 found, 443.099.

##### *N*-(2-((2-Chloro-6-(methylamino)-9*H*-purin-9-yl)methyl)phenyl)-2,2,2-trifluoroacetamide (**15**)

To a solution of compound **48** (0.07 g, 0.237 mmol) in DCM (2 mL) was added pyridine (0.024 mL, 0.308 mmol). The solution was cooled down to 0 °C and trifluoroacetic anhydride (0.039 mL, 0.280 mmol) was added dropwise after 30 min. After the reaction completion (TLC), the volatiles were removed *in vacuo*. The crude product was subsequently used in the next step without further purification. The final compound **15** was prepared following the General method 2 for S_N_Ar. The crude product was purified using flash column chromatography (SiO_2_; EtOAc/MeOH = 2:1 → 3:1) and the desired compound was obtained as a white solid (0.030 g, 30% after two steps). ^1^H NMR (400 MHz, DMSO – *d*^6^) δ 11.26 (s, 1H), 8.28–8.26 (m, 1H), 8.10 (s, 1H), 7.42–7.31 (m, 3H), 7.18 (dd, J = 7.6, 1.5 Hz, 1H), 5.33 (s, 2H), 2.92 (d, J = 4.5 Hz, 1H); ^13^C NMR (101 MHz, DMSO – *d*^6^) δ 155.8 (q, J = 36.7 Hz), 155.5, 153.4, 149.4, 141.0, 132.6, 132.3, 128.8, 128.7, 128.1, 127.4, 118.1, 117.4, 114.5, 42.7, 27.2. ^19^F NMR (376 MHz, DMSO) δ −73.90. LRMS (ESI) *m*/*z*: [M + H]^+^ calcd for C_15_H_12_ClF_3_N_6_O; 385.079 found, 385.078.

##### 2-Chloro-9-(2-(difluoromethyl)benzyl)-*N*-methyl-9*H*-purin-6-amine (**16**)

2-(Difluoromethyl)benzoic acid (0.500 g, 2.90 mmol) was dissolved in THF (5 mL) and BH_3_. DMS (0.687 mL, 7.25 mmol) was added dropwise. The reaction mixture was stirred under dinitrogen atmosphere at rt overnight. After the reaction completion (monitored by TLC), the reaction was quenched by aq. sol. of NaHCO_3_. The mixture was extracted into EtOAc (3 × 7 mL). The combined organic layers were dried over MgSO_4_, filtrated, and evaporated. The resulting alcohol was dissolved in DCM (5.2 mL) together with one drop of DMF, followed by the addition of SOCl_2_ (0.315 mL, 4.35 mmol). The reaction mixture was stirred at rt overnight. The volatiles were removed *in vacuo* and the resulting alkyl chloride was used in the next step without further purification.

The N^9^ alkylation was performed following General procedure 1 using 2,6-dichloropurine **44** (0.48 g, 2.54 mmol) and 1-chloromethyl-2-difluoromethylbenzen (0.449 g, 2.54 mmol). The crude product was purified using flash column chromatography (SiO_2_; EtOAc/Hept = 1:1) and the desired compound was obtained as a white solid (0.210 g, 25%). ^1^H NMR (400 MHz, CDCl_3_ - *d*) δ 8.06 (s, 1H), 7.54–7.46 (m, 3H), 7.34–7.32 (m, 1H), 6.84 (t, *J* = 54.8 Hz, 1H), 5.63 (s, 2H); ^13^C NMR (101 MHz, CDCl_3_ - *d*) δ 153.4, 152.2, 145.9 (t, *J* = 2.6 Hz), 132.7, 132.1 (t, *J* = 2.0 Hz), 131.9 (t, *J* = 21.6 Hz), 130.8, 130.7, 129.4, 128.3 (t, *J* = 8.2 Hz), 116.0 (t, *J* = 238.9 Hz), 44.4 (t, *J* = 2.7 Hz).

The final compound **16** was prepared following the General method 2 for S_N_Ar starting from corresponding 9-alkyl-2,6-dichloro-9*H*-purine (0.080 g, 0.243 mmol). The crude product was purified using flash column chromatography (SiO_2_; EtOAc/Hept = 1.1:1) and the desired compound was obtained as a white solid (0.071 g, 90%). ^1^H NMR (400 MHz, CDCl_3_ - *d*) δ 7.65 (s, 1H), 7.54–7.51 (m, 1H), 7.47–7.40 (m, 2H), 7.23 (d, *J* = 8.3 Hz,, 1H), 6.87 (t, *J* = 54.9 Hz, 1H), 6.09–5.94 (bs, 1H), 5.52 (s, 2H), 3.28–3.08 (bs, 3H); ^13^C NMR (101 MHz, CDCl_3_ - *d*) δ 156.2, 140.1, 133.98 (t, *J* = 2.2 Hz), 131.86 (t, *J* = 21.4 Hz), 131.8 (t, *J* = 2.1 Hz), 130.1, 128.8, 127.63 (t, *J* = 8.1 Hz), 115.49 (t, *J* = 238.8 Hz), 43.5. LRMS (ESI) *m*/*z*: [M + H]^+^ calcd for C_14_H_13_ClF_2_N_5_; 324.082 found, 324.082.

##### 2-Chloro-*N*-methyl-9-(2-(trifluoromethyl)benzyl)-9*H*-purin-6-amine (**17**)

2-(Trifluoromethyl)benzoic acid (0.500 g, 2.63 mmol) was dissolved in THF (5.2 mL) and BH_3_. DMS (0.748 mL, 7.89 mmol) was added dropwise. The reaction mixture was stirred under dinitrogen atmosphere at rt overnight. After the reaction completion (monitored by TLC), the reaction was quenched by aq. sol. of NaHCO_3_. The mixture was extracted into EtOAc (3 × 7 mL). The combined organic layers were dried over MgSO_4_, filtrated, and evaporated. The resulting alcohol was dissolved in DCM (5.2 mL) together with one drop of DMF, followed by the addition of SOCl_2_ (0.504 mL, 6.95 mmol). The reaction mixture was stirred at rt overnight. The volatiles were removed *in vacuo* and the resulting alkyl chloride was used in the next step without further purification.

The N^9^ alkylation was performed following General procedure 1 using 2,6-dichloropurine **44** (0.525 g, 2.78 mmol) and 1-chloromethyl-2-trifluoromethylbenzen (0.540 g, 2.78 mmol). The crude product was purified using flash column chromatography (SiO_2_; EtOAc/Hept = 1:1.2) and the desired compound was obtained as a white solid (0.234 g, 24% after three steps). ^1^H NMR (400 MHz, CDCl_3_ - *d*) δ 8.02 (s, 1H), 7.77 (d, *J* = 7.0 Hz, 1H), 7.58–7.47 (m, 2H), 7.29 (d, *J* = 7.6 Hz, 1H), 5.63 (s, 2H); ^13^C NMR (101 MHz, CDCl_3_ - *d*) δ 153.6, 153.5, 152.3, 133.1, 132.5, 130.7, 130.5, 129.4, 128.5 (q, *J* = 30.7 Hz), 128.3, 126.9 (q, *J* = 5.6 Hz), 125.6, 122.9, 44.5 (q, *J* = 2.9 Hz).

The final compound was prepared following the General method 2 for S_N_Ar from corresponding 9-alkyl-2,6-dichloro-9*H*-purine (0.1 g, 0.288 mmol). The crude product was purified using flash column chromatography (SiO_2_; EtOAc/Hept = 1:1) and the desired compound was obtained as a white solid (0.045 g, 46%). ^1^H NMR (400 MHz, DMSO – *d*^6^) δ 8.33–8.27 (m, 1H), 8.17 (s, 1H), 7.82 (d, *J* = 7.7 Hz, 1H), 7.61 (t, *J* = 7.7 Hz, 1H), 7.54 (t, *J* = 7.8 Hz, 1H), 6.85 (d, *J* = 7.7 Hz, 1H), 5.55 (s, 2H), 2.94 (d, *J* = 4.6 Hz, 3H); ^13^C NMR (101 MHz, DMSO – *d*^6^) δ 156.1, 154.01, 150.1, 141.9, 135.0, 133.7, 128.8, 128.5, 126.77 (q, *J* = 5.7 Hz), 126.6, 126.3, 126.1, 126.0, 123.4, 118.8, 43.6, 27.7. LRMS (ESI) *m*/*z*: [M + H]^+^ calcd for C_14_H_12_ClF_3_N_5_; 342.073 found, 342.074.

##### 2-Chloro-9-(2-methoxybenzyl)-*N*-methyl-9*H*-purin-6-amine (**18**)

The N^9^ alkylation was performed following General procedure 1 using 2,6-dichloropurine **44** (0.2 g, 1.06 mmol) and 1-chloromethyl-2-methoxybenzen (0.165 g, 1.06 mmol) that was prepared following reported procedure.^46^ The crude product was purified using flash column chromatography (SiO_2_; EtOAc/Hept = 1.2:1) and the desired compound was obtained as a white solid (0.103 g, 31%). ^1^H NMR (400 MHz, CDCl_3_ - *d*) δ 8.14 (s, 1H), 7.39 (dd, *J* = 7.5, 1.7 Hz, 1H), 7.35 (td, *J* = 7.9, 1.7 Hz, 1H), 6.96 (td, *J* = 7.5, 1.1 Hz, 1H), 6.91 (d, *J* = 8.2 Hz, 1H), 5.38 (s, 2H), 3.86 (s, 3H); ^13^C NMR (101 MHz, CDCl_3_ - *d*) δ 157.5, 153.4, 152.9, 151.5, 146.6, 131.1, 131.0, 130.7, 122.3, 121.2, 110.9, 55.6, 43.8.

The final compound **18** was prepared following the General method 2 starting from corresponding 9-alkyl-2,6-dichloro-9*H*-purine (0.048 g, 0.155 mmol). The crude product was purified using flash column chromatography (SiO_2_; EtOAc/Hept = 3:1) and the desired compound was obtained as a white solid (0.036 g, 76%). ^1^H NMR (400 MHz, CDCl_3_ - *d*) δ 7.72 (s, 1H), 7.31 (td, *J* = 7.9, 1.7 Hz, 1H), 7.26 (dd, *J* = 7.5, 1.7 Hz, 1H), 6.94–6.8 (m, 2H), 6.22–6.02 (bs, 1H), 5.30 (s, 2H), 3.86 (s, 3H), 3.27–3.06 (bs, 3H); ^13^C NMR (101 MHz, CDCl_3_ - *d*) δ 157.4, 156.1, 140.6, 130.6, 130.5, 130.3, 127.7, 123.5, 121.0, 118.6, 110.7, 55.5, 42.7, 27.8. LRMS (ESI) *m*/*z*: [M + H]^+^ calcd for C_14_H_15_ClN_5_O; 304.096 found, 304.097.

##### Methyl-3-((2-chloro-6-(methylamino)-9*H*-purin-9-yl)methyl)benzoate (**19**)

The N^9^ alkylation was performed following General procedure 1 using 2,6-dichloropurine **44** (0.825 g, 4.37 mmol) and methyl-3-bromomethylbenzoate (1.0 g, 4.37 mmol). The crude product was purified using flash column chromatography (SiO_2_; EtOAc/Hept = 2:1) and the desired compound was obtained as a white solid (0.465 g, 31%). ^1^H NMR (400 MHz, CDCl_3_ - *d*) δ 8.08 (s, 1H), 8.04 (dt, *J* = 7.3, 1.7 Hz, 1H), 8.01–8.00 (bs, 1H), 5.46 (s, 2H), 3.92 (s, 3H); ^13^C NMR (101 MHz, CDCl_3_ - *d*) δ 166.2, 153.4, 153.1, 152.1, 145.4, 134.5, 132.5, 131.4, 130.7, 130.3, 129.6, 129.1, 52.4, 47.6.

The final compound **19** was prepared following the General method 2 from corresponding 9-alkyl-2,6-dichloro-9*H*-purine (0.465 g, 1.38 mmol). The crude product was purified using flash column chromatography (SiO_2_; EtOAc/Hept = 3:1) and the desired compound was obtained as a white solid (0.374 g, 82%). ^1^H NMR (400 MHz, DMSO – *d*^6^) δ 8.28 (s, 1H), 8.29–8.21 (m, 1H), 7.90–7.87 (m, 2H), 7.56–7.49 (m, 2H), 5.43 (s, 2H), 3.83 (s, 3H), 2.92 (d, *J* = 4.6 Hz, 1H); ^13^C NMR (101 MHz, DMSO – *d*^6^) δ 165.9, 155.6, 153.5, 149.5, 141.2, 137.5, 132.3, 130.1, 129.4, 128.6, 128.0, 118.3, 52.3, 45.9, 27.2. LRMS (ESI) *m*/*z*: [M + H]^+^ calcd for C_15_H_15_ClN_5_O_2_; 332.091 found, 332.091.

##### 3-((2-Chloro-6-(methylamino)-9*H*-purin-9-yl)methyl)benzoic Acid (**20**)

The corresponding methyl ester **19** (0.1 g, 0.301 mmol) was dissolved in dioxane (3 mL) followed by the addition of 38% HCl (2 mL). The reaction mixture was refluxed for 5 h. The reaction mixture was cooled to 0 °C and left for 4 h at this temperature. The white precipitate was filtered off and dried on air. The carboxylic acid **20** was isolated as a white solid (60 mg, 63%). ^1^H NMR (400 MHz, DMSO – *d*^6^) δ 8.32 (s, 1H), 8.32–8.25 (bs, 1H), 7.88–7.83 (m, 2H), 7.54–7.47 (m, 2H), 5.43 (s, 2H), 2.92 (d, *J* = 3.9 Hz, 3H). δ ^13^C NMR (101 MHz, DMSO – *d*^6^) δ 166.9, 155.4, 153.6, 149.4, 141.2, 137.2, 131.9, 131.2, 129.1, 128.8, 128.1, 117.9, 46.0, 27.2. LRMS (ESI) *m*/*z*: [M + H]^+^ calcd for C_14_H_13_ClN_5_O_2_; 318.075 found, 318.075.

##### 9-(3-(2*H*-Tetrazol-5-yl)benzyl)-2-chloro-*N*-methyl-9*H*-purin-6-amine (**21**)

To a solution of **26** (0.05 g, 0.167 mmol) in DMSO (1.6 mL) was added anhydrous CuSO_4_ (0.066 g, 0.417 mmol) and NaN_3_ (0.010 g, 0.167 mmol). The reaction mixture was heated at 100 °C for 16 h. The reaction mixture was quenched with 10% HCl and extracted into EtOAc. Combined organic layers were washed with 10% aq. sol. of LiCl and dried over MgSO_4_ and concentrated under reduced pressure. The crude product was purified using flash column chromatography (SiO_2_; DCM/MeOH = 5:1 → 1:1) and the desired compound was obtained as a white solid (0.021 g, 36%). ^1^H NMR (400 MHz, DMSO – *d*^6^) δ 8.28 (s, 1H), 8.26–8.23 (m, 1H), 7.89 (d, *J* = 7.7 Hz, 1H), 7.84 (s, 1H), 7.35 (t, *J* = 7.7 Hz, 1H), 7.15 (d, *J* = 7.6 Hz, 1H), 5.39 (s, 2H), 2.92 (d, *J* = 4.7 Hz, 2H).^13^C NMR (101 MHz, DMSO – *d*^6^) δ 160.3, 155.6, 153.5, 149.5, 141.3, 136.8, 133.1, 128.8, 125.8, 125.2, 124.5, 118.3, 46.3, 27.2.

##### 3-((2-Chloro-6-(methylamino)-9*H*-purin-9-yl)methyl)benzamide (**22**)

Corresponding carboxylic acid **20** (0.064 g, 0.201 mmol) was suspended in dry DMF (1.6 mL) followed by the addition of DIPEA (0.052 mL, 0.301 mmol). The reaction mixture was cooled to 0 °C and COMU (0.128 g, 0.301 mmol) was added after 30 min. To the reaction mixture was added 7 N NH_3_ in THF (0.2 mL) after an additional 30 min. The reaction was slowly warmed to rt and stirred overnight. The reaction mixture was then extracted into EtOAc and the combined organic layers were dried over MgSO_4_ and evaporated. The crude product was purified using flash column chromatography (SiO_2_; EtOAc/MeOH = 2:0.1 → 2:0.2) and the desired compound was obtained as a white solid (0.015 g, 24%). ^1^H NMR (400 MHz, DMSO – *d*^6^) δ 8.25 (s, 1H), 8.25–8.20 (bs, 1H), 7.98 (s, 1H), 7.79 (dt, *J* = 7.3, 1.7 Hz, 1H), 7.75 (s, 1H), 7.46–7.35 (m, 3H), 5.39 (s, 2H), 2.92 (d, *J* = 4.6 Hz, 2H); ^13^C NMR (101 MHz, DMSO – *d*^6^) δ 168.0, 156.0, 153.9, 149.9, 141.7, 137.4, 135.2, 130.6, 129.2, 127.2, 127.0, 118.7, 46.5, 27.7. LRMS (ESI) *m*/*z*: [M + H]^+^ calcd for C_14_H_14_ClN_6_O; 317.091 found, 317.091.

##### 3-((2-Chloro-6-(methylamino)-9*H*-purin-9-yl)methyl)-*N*-methylbenzamide (**23**)

Corresponding carboxylic acid **20** (0.027 g, 0.084 mmol) was suspended in dry DMF (0.6 mL) followed by the addition of DIPEA (0.021 mL, 0.126 mmol). The reaction mixture was cooled to 0 °C and COMU (0.053 g, 0.126 mmol) was added after 30 min. To the reaction mixture was added 2 M solution of MeNH_2_ in THF (0.15 mL) after an additional 30 min. The reaction was slowly warmed to rt and stirred overnight. The reaction mixture was then extracted into EtOAc and the combined organic layers were dried over MgSO_4_ and evaporated. The crude product was purified using flash column chromatography (SiO_2_; EtOAc/MeOH = 2:0.1 → 2:0.2) and the desired compound was obtained as a white solid (0.017 g, 60%). ^1^H NMR (400 MHz, DMSO – *d*^6^) δ 8.44–8.43 (m, 1H), 8.28 (s, 2H), 7.75–7.71 (m, 2H), 7.44 (t, *J* = 7.4 Hz, 1H), 7.39 (dt, *J* = 7.7, 1.6 Hz, 1H), 5.39 (s, 2H), 2.92 (d, *J* = 4.7 Hz, 3H), 2.76 (d, *J* = 4.6 Hz, 3H). ^13^C NMR (101 MHz, DMSO – *d*^6^) δ 166.8, 156.0, 153.9, 149.9, 141.7, 137.4, 135.5, 130.4, 129.2, 126.7, 126.6, 118.7, 46.5, 27.7, 26.7. LRMS (ESI) *m*/*z*: [M + H]^+^ calcd for C_15_H_16_ClN_6_; 331.107 found, 331.106.

##### 9-(3-Bromobenzyl)-2-chloro-*N*-methyl-9*H*-purin-6-amine (**24**)

The N^9^ alkylation was performed following General procedure 1 using 2,6-dichloropurine **44** (0.5 g, 2.65 mmol) and 1-bromo-3-bromomethylbenzen (0.661 g, 2.65 mmol). The crude product was purified using flash column chromatography (SiO_2_; EtOAc/Hept = 1.2:2) and the desired compound was obtained as a white solid (0.35 g, 37%). ^1^H NMR (400 MHz, DMSO – *d*^6^) δ 8.84 (s, 1H), 7.63–7.61 (bs, 1H), 7.54–7.51 (m, 1H), 7.34–7.29 (m, 2H), 5.50 (s, 2H); ^13^C NMR (101 MHz, DMSO – *d*^6^) δ 153.4, 151.1, 149.8, 148.4, 138.2, 131.0, 131.0, 130.6, 130.5, 126.8, 121.9, 46.4.

The final compound **24** was prepared following the General method 2 starting from corresponding 9-alkyl-2,6-dichloro-9*H*-purine (0.150 g, 0.419 mmol). The crude product was purified using flash column chromatography (SiO_2_; EtOAc/Hept = 1:1) and the desired compound was obtained as a white solid (0.12 g, 81%). ^1^H NMR (400 MHz, DMSO – *d*^6^) δ 8.26 (s, 1H), 7.54–7.49 (m, 2H), 7.32 (t, *J* = 7.7 Hz, 1H), 7.24 (d, *J* = 7.8 Hz, 1H), 5.35 (s, 2H), 2.92 (d, *J* = 4.7 Hz, 3H). ^13^C NMR (101 MHz, DMSO – *d*^6^) δ 155.6, 153.5, 149.4, 141.1, 139.4, 131.0, 130.7, 130.2, 126.5, 121.9, 118.3, 45.6, 27.2. LRMS (ESI) *m*/*z*: [M + H]^+^ calcd for C_13_H_12_BrClN_5_; 351.995 found, 351.996.

##### 2-Chloro-9-(3-chlorobenzyl)-*N*-methyl-9*H*-purin-6-amine (**25**)

The final compound was prepared following the General method 2 starting from corresponding compound **38** (0.272 g, 0.867 mmol). The crude product was purified using flash column chromatography (SiO_2_; EtOAc/Hept = 3:1) and the desired compound was obtained as a white solid (0.191 g, 71%). ^1^H NMR (400 MHz, DMSO – *d*^6^) δ 8.26 (s, 1H), 8.28–8.21 (bs, 1H), 7.40–7.35 (m, 3H), 7.23–7.18 (m, 1H), 5.35 (s, 2H), 2.92 (d, *J* = 4.7 Hz, 3H); ^13^C NMR (101 MHz, DMSO – *d*^6^) δ 155.6, 153.5, 149.4, 141.2, 139.2, 133.3, 130.8, 127.9, 127.4, 126.1, 118.3, 45.7, 27.2. LRMS (ESI) *m*/*z*: [M + H]^+^ calcd for C_13_H_12_Cl_2_N_5_; 308.045 found, 308.046.

##### 3-((2-Chloro-6-(methylamino)-9*H*-purin-9-yl)methyl)benzonitrile (**26**)

The N^9^ alkylation was performed following General procedure 1 using 2,6-dichloropurine **44** (0.337 g, 1.79 mmol) and 3-(bromomethyl)benzonitrile (0.35 g, 1.79 mmol). The crude product was purified using flash column chromatography (SiO_2_; EtOAc/Hept = 2.5:1) and the desired compound was obtained as a white solid (0.15 g, 28%). ^1^H NMR (400 MHz, CDCl_3_ - *d*) δ 8.11 (s, 1H), 7.67 (dt, *J* = 7.1, 1.7 Hz, 1H), 7.61–7.60 (m, 1H), 7.57–7.41 (m, 2H), 5.47 (s, 2H). ^13^C NMR (101 MHz, CDCl_3_ - *d*) δ 153.7, 153.2, 152.5, 145.2, 135.9, 132.8, 132.3, 131.4, 130.8, 130.5, 117.9, 113.8, 47.2.

The final compound **26** was prepared following the General method 2 from corresponding 9-alkyl-2,6-dichloro-9*H*-purine (0.082 g, 0.262 mmol). The crude product was purified using flash column chromatography (SiO_2_; EtOAc/MeOH = 10:1) and the desired compound was obtained as a white solid (0.057 g, 71%). ^1^H NMR (400 MHz, DMSO – *d*^6^) δ 8.29–8.21 (m, 2H), 7.80–7.75 (m, 2H), 7.58–7.55 (m, 2H), 5.41 (s, 2H), 2.92 (d, *J* = 4.6 Hz, 1H). ^13^C NMR (101 MHz, DMSO – *d*^6^) δ 155.6, 153.4, 149.4, 141.1, 138.2, 132.3, 131.7, 131.1, 130.0, 118.5, 118.3, 111.6, 45.5, 27.2. LRMS (ESI) *m*/*z*: [M + H]^+^ calcd for C_14_H_12_ClN_6_; 299.081 found, 299.080.

##### 2-Chloro-9-(3-methoxybenzyl)-*N*-methyl-9*H*-purin-6-amine (**27**)

The N^9^ alkylation was performed following General procedure 1 using 2,6-dichloropurine **44** (0.2 g, 1.06 mmol) and 1-bromomethyl-3-methoxybenzen (0.212 g, 1.06 mmol). The crude product was purified using flash column chromatography (SiO_2_; EtOAc/Hept = 2:1.2) and the desired compound was obtained as a white solid (0.115 g, 35%). ^1^H NMR (400 MHz, CDCl_3_ - *d*) δ 8.02 (s, 1H), 7.25 (d, *J* = 13.5, 5.6 Hz, 1H), 6.87–6.80 (m, 3H), 5.33 (s, 2H), 3.75 (s, 3H); ^13^C NMR (101 MHz, CDCl_3_ - *d*) δ 160.4, 153.3, 153.3, 152.0, 145.7, 135.5, 130.8, 130.6, 120.3, 114.4, 114.1, 55.5, 48.1.

The final compound **27** was prepared following the General method 2 starting from corresponding 9-alkyl-2,6-dichloro-9*H*-purine (0.050 g, 0.161 mmol). The crude product was purified using flash column chromatography (SiO_2_; EtOAc/Hept = 2:1) and the desired compound was obtained as a white solid (0.034 g, 69%). ^1^H NMR (400 MHz, CDCl_3_ - *d*) δ 7.64 (s, 1H), 7.28–7.24 (m, 1H), 6.87–6.81 (m, 3H), 6.20–6.01 (bs, 1H), 5.27 (s, 2H), 3.77 (s, 3H), 3.25–3.08 (bs, 3H). ^13^C NMR (101 MHz, CDCl_3_ - *d*) δ 160.2, 156.2, 155.0, 150.2, 139.9, 136.8, 130.3, 120.2, 118.7, 114.0, 113.8, 55.4, 47.3, 27.8. LRMS (ESI) *m*/*z*: [M + H]^+^ calcd for C_14_H_15_ClN_5_O; 304.096 found, 304.097.

##### Methyl-4-((2-chloro-6-(methylamino)-9*H*-purin-9-yl)methyl)benzoate (**28**)

The N^9^ alkylation was performed following General procedure 1 using 2,6-dichloropurine **44** (0.2 g, 1.06 mmol) and methyl-4-(bromomethyl)benzoate (0.242 g, 1.06 mmol). The crude product was purified using flash column chromatography (SiO_2_; EtOAc/Hept = 1:2) and the desired compound was obtained as a white solid (0.145 g, 40%). ^1^H NMR (400 MHz, CDCl_3_ - *d*) δ 8.08 (s, 1H), 8.05–8.03 (m, 2H), 7.38–7.34 (m, 2H), 5.47 (s, 2H), 3.91 (s, 3H). ^13^C NMR (101 MHz, CDCl_3_ - *d*) δ 166.3, 153.5, 153.3, 152.2, 145.5, 138.9, 131.0, 130.8, 130.7, 128.0, 52.5, 47.7.

The final compound **28** was prepared following the General method 2 from corresponding 9-alkyl-2,6-dichloro-9*H*-purine (0.052 g, 0.154 mmol). The crude product was purified using flash column chromatography (SiO_2_; EtOAc/Hept = 2:1) and the desired compound was obtained as a white solid (0.038 g, 74%). ^1^H NMR (400 MHz, DMSO – *d*^6^) δ 8.26 (s, 2H), 7.93 (d, 2H), 7.35 (d, 2H), 5.45 (s, 2H), 3.83 (s, 3H), 2.92 (bs, 3H); ^13^C NMR (101 MHz, DMSO) δ 165.9, 155.6, 153.5, 149.5, 142.0, 141.3, 129.6, 129.0, 127.4, 118.3, 52.2, 45.9, 27.2. HRMS (ESI) *m*/*z*: [M + H]^+^ calcd for C_15_H_15_ClN_5_O_2_; 332.091 found, 332.091.

##### 3-((2-Chloro-6-(methylamino)-9*H*-purin-9-yl)methyl)benzoic Acid (**29**)

The corresponding methyl ester **28** (0.2 g, 0.602 mmol) was dissolved in dioxane (6 mL) followed by the addition of 38% HCl (4 mL). The reaction mixture was refluxed for 5 h. The reaction mixture was cooled to 0 °C and left for 4 h at this temperature. The white precipitate was filtered off and dried on air. The carboxylic acid **29** was isolated as a white solid (164 mg, 85%). ^1^H NMR (400 MHz, DMSO – *d*^6^) δ 8.52 (s, 1H), 8.48–8.38 (bs, 1H), 7.92–7.88 (m, 2H), 7.35 (d, *J* = 8.1 Hz, 2H), 5.47 (s, 2H), 2.93 (s, 3H); ^13^C NMR (101 MHz, DMSO) δ 167.0, 155.1, 154.0, 149.3, 141.2, 141.0, 130.4, 129.8, 127.5, 116.8, 46.4, 27.3. LRMS (ESI) *m*/*z*: [M + H]^+^ calcd for C_14_H_13_ClN_5_O_2_; 318.075 found, 318.075.

##### (4-((2-Chloro-6-(methylamino)-9*H*-purin-9-yl)methyl)phenyl)methanol (**30**)

The N^9^ alkylation was performed following General procedure 1 using 2,6-dichloropurine **44** (0.2 g, 1.06 mmol) and (4-(bromomethyl)phenyl)methanol (0.212 g, 1.06 mmol) that was prepared following reported procedure.^47^ The crude product was purified using flash column chromatography (SiO_2_; EtOAc/Hept = 3:1 → 5:1) and the desired compound was obtained as a white solid (0.12 g, 37%). ^1^H NMR (400 MHz, DMSO – *d*^6^) δ 8.83 (s, 1H), 8.74 (s, 1H), 7.32–7.27 (m, 4H), 5.47 (s, 2H), 5.24–5.07 (bs, 1H), 4.47 (s, 2H); ^13^C NMR (101 MHz, DMSO) δ 153.4, 151.1, 149.8, 148.4, 142.6, 133.9, 130.5, 127.5, 126.8, 62.5, 47.0.

The final compound **30** was prepared following the General method 2 from corresponding 9-alkyl-2,6-dichloro-9*H*-purine (0.05 g, 0.264 mmol). The crude product was purified using flash column chromatography (SiO_2_; EtOAc/MeOH = 1:0.1) and the desired compound was obtained as a white solid (0.075 g, 92%). ^1^H NMR (400 MHz, DMSO – *d*^6^) δ 8.22 (s, 1H), 8.23–8.18 (bs, 1H), 7.28 (d, *J* = 8.1 Hz, 2H), 7.22 (d, *J* = 8.2 Hz, 2H), 5.31 (s, 2H), 5.17 (t, *J* = 5.7 Hz, 1H), 4.45 (d, *J* = 5.7 Hz, 1H), 2.91 (d, *J* = 4.6 Hz, 3H); 13C NMR (101 MHz, DMSO) δ ^13^C NMR (101 MHz, DMSO) δ 155.6, 153.4, 149.5, 142.2, 141.2, 135.1, 127.2, 126.8, 118.3, 62.6, 46.2, 27.2. LRMS (ESI) *m*/*z*: [M + H]^+^ calcd for C_14_H_14_ClN_5_O; 304.096 found, 304.096

##### *N*-(4-Chloro-2-((2-chloro-6-(methylamino)-9*H*-purin-9-yl)methyl)phenyl)methanesulfonamide (**31**)

To a solution of *N*-(4-chloro-2-(hydroxymethyl)phenyl)methanesulfonamide (0.747 g, 3.17 mmol) in DCM (6.3 mL), prepared following the reported procedure,^48^ was added SOCl_2_ (0.342 mL, 4.75 mmol) dropwise. The reaction mixture was stirred at rt for 90 min. The volatiles were removed *in vacuo* and the crude product was dissolved in DMF (6.2 mL), followed by the addition of 2,6-dichloropurine **44** (0.599 g, 3.17 mmol) and K_2_CO_3_ (0.649 g, 4.71 mmol). The reaction was quenched by aq. sol. of NH_4_Cl after 5 h. The reaction mixture was extracted into EtOAc (3 × 12 mL) and combined organic layers were dried over MgSO_4_, filtrated, and evaporated. The residue was dissolved in EtOH (4 mL) and 33% MeNH_2_ in EtOH (1 mL) was added. The reaction mixture was stirred at rt for 1 h. After the reaction completion, the volatiles were removed *in vacuo*. The crude compound was purified using flash column chromatography (SiO_2_; EtOAc/Hept = 4:1 → 6:1) and the desired compound was obtained as a white solid (0.074 g, 6% after three steps). ^1^H NMR (400 MHz, DMSO – *d*^6^) δ 9.61 (s, 1H), 8.32–8.27 (m, 1H), 8.21 (s, 1H), 7.45–7.40 (m, 2H), 6.98–6.96 (bs, 1H), 5.43 (s, 2H), 3.10 (s, 3H), 2.93 (d, *J* = 4.6 Hz, 3H); ^13^C NMR (101 MHz, DMSO – *d*^6^) δ 155.6, 153.3, 149.4, 141.3, 135.6, 133.8, 133.1, 128.8, 128.6, 118.2, 42.6, 27.2. LRMS (ESI) *m*/*z*: [M + H]^+^ calcd for C_14_H_15_Cl_2_N_6_O_2_S; 401.035 found, 401.035.

##### 3-Chloro-5-((2-chloro-6-(methylamino)-9*H*-purin-9-yl)methyl)benzoic Acid (**32**)

The corresponding methyl ester **33** (0.06 g, 0.161 mmol) was dissolved in dioxane (2.5 mL) followed by the addition of 38% HCl (1 mL). The reaction mixture was refluxed for 5 h. The reaction mixture was cooled to 0 °C and left for 4 h at this temperature. The white precipitate was filtered off and dried on air. The carboxylic acid **32** was isolated as a white solid (0.045 g, 79%). ^1^H NMR (400 MHz, DMSO – *d*^6^) δ 8.36 (s, 1H), 8.35–8.28 (bs, 1H), 7.83 (t, *J* = 1.8 Hz, 1H), 7.77 (t, *J* = 1.6 Hz, 1H), 7.68 (t, *J* = 1.9 Hz, 1H), 5.44 (s, 2H), 2.92 (d, *J* = 3.6 Hz, 3H). ^13^C NMR (101 MHz, DMSO – *d*^6^) δ 165.7, 155.4, 153.6, 149.3, 141.1, 139.5, 133.6, 133.3, 131.7, 128.3, 126.9, 117.8, 45.5, 27.2. LRMS (ESI) *m*/*z*: [M + H]^+^ calcd for C_14_H_12_Cl_2_N_5_O_2_; 352.036 found, 352.036.

##### Methyl-3-chloro-5-((2-chloro-6-(methylamino)-9*H*-purin-9-yl)methyl)benzoate (**33**)

The N^9^ alkylation was performed following General procedure 1 using 2,6-dichloropurine **44** (0.459 g, 2.43 mmol) and methyl-3-(bromomethyl)-5-chlorobenzoate (0.641 g, 2.43 mmol) that was prepared following reported procedure.^49^ The crude product was purified using flash column chromatography (SiO_2_; EtOAc/Hept = 1:1) and the desired compound was obtained as a white solid (0.51 g, 56%). ^1^H NMR (400 MHz, CDCl_3_ - *d*) δ 8.10 (s, 1H), 8.02–8.00 (m, 1H), 7.88–7.86 (m, 1H), 7.48 (t, *J* = 1.9 Hz, 1H), 5.44 (s, 2H), 3.92 (s, 3H). ^13^C NMR (101 MHz, CDCl_3_ - *d*) δ 165.2, 153.7, 153.2, 152.4, 145.3, 136.5, 135.9, 133.1, 132.3, 130.8, 130.5, 127.2, 52.9, 47.2.

The final compound **33** was prepared following the General method 2 from corresponding 9-alkyl-2,6-dichloro-9*H*-purine (0.337 g, 0.906 mmol). The crude product was purified using flash column chromatography (SiO_2_; EtOAc/Hept = 4:1) and the desired compound was obtained as a white solid (0.176 g, 53%). ^1^H NMR (400 MHz, DMSO – *d*^6^) δ 8.32–8.24 (m, 2H), 7.86–7.81 (m, 2H), 7.71–7.70 (m, 1H), 5.44 (s, 2H), 3.84 (s, 3H), 2.92 (d, *J* = 4.6 Hz, 3H); ^13^C NMR (101 MHz, DMSO – *d*^6^) δ 164.7, 155.6, 153.5, 149.4, 141.1, 139.8, 133.7, 132.2, 132.0, 128.2, 126.8, 118.3, 52.6, 45.4, 27.2. LRMS (ESI) *m*/*z*: [M + H]^+^ calcd for C_15_H_14_Cl_2_N_5_O_2_; 366.052 found, 366.052.

##### 2-Chloro-9-(3-chloro-4-methoxybenzyl)-*N*-methyl-9*H*-purin-6-amine (**34**)

The compound was prepared following the general procedure using 2,6-dichloropurine **44** (0.566 g, 3.00 mmol) and 2-chloro-4-(chloromethyl)-1-methoxybenzene (0.573 g, 3.00 mmol). The crude product was purified using flash column chromatography (SiO_2_; EtOAc/Hept = 3:2) and the desired compound was obtained as a white solid (0.71 g, 69%). ^1^H NMR (400 MHz, CDCl_3_ - *d*) δ 8.05 (s, 1H), 7.34 (d, *J* = 2.2 Hz, 1H), 7.21 (dd, *J* = 8.5, 2.3 Hz, 1H), 6.90 (d, *J* = 8.5 Hz, 1H), 5.32 (s, 2H), 3.88 (s, 3H); ^13^C NMR (101 MHz, CDCl_3_ - *d*) δ 155.7, 153.3, 153.1, 152.0, 145.5, 130.8, 130.2, 128.0, 127.0, 123.4, 112.6, 56.4, 47.2.

The final compound **34** was prepared following the General method 2 from corresponding 9-alkyl-2,6-dichloro-9*H*-purine (0.134 g, 0.397 mmol). The crude product was purified using flash column chromatography (SiO_2_; EtOAc/Hept = 2:3 → 1:4) and the desired compound was obtained as a white solid (0.08 g, 59%). ^1^H NMR (400 MHz, DMSO – *d*^6^) δ 8.24 (s, 1H), 8.26–8.18 (bs, 1H), 7.44 (d, *J* = 2.2 Hz, 1H), 7.25 (dd, *J* = 8.5, 2.2 Hz, 1H), 7.12 (d, *J* = 8.5 Hz, 1H), 5.26 (s, 2H), 3.82 (s, 3H), 2.91 (d, *J* = 4.6 Hz, 3H); ^13^C NMR (101 MHz, DMSO – *d*^6^) δ 155.5, 154.2, 153.4, 149.3, 141.0, 129.8, 129.3, 127.9, 121.0, 118.3, 113.0, 56.1, 45.3, 27.2. LRMS (ESI) *m*/*z*: [M + H]^+^ calcd for C_14_H_14_Cl_2_N_5_O; 338.057 found, 338.057.

##### 2-Chloro-9-(3,5-dichloro-4-methoxybenzyl)-*N*-methyl-9*H*-purin-6-amine (**35**)

3,5-Dichloro-4-methoxybenzoic acid (0.500 g, 2.26 mmol) was dissolved in THF (5 mL) and BH_3_. DMS (0.529 mL, 5.65 mmol) was added dropwise. The reaction mixture was stirred under dinitrogen atmosphere at rt overnight. After the reaction completion (monitored by TLC), the reaction was quenched by aq. sol. of NaHCO_3_. The mixture was extracted into EtOAc (3 × 5 mL). The combined organic layers were dried over MgSO_4_, filtrated, and evaporated. The resulting alcohol was dissolved in DCM (3 mL) together with one drop of DMF, followed by the addition of SOCl_2_ (0.261 mL, 3.63 mmol). The reaction mixture was stirred at rt overnight. The volatiles were removed *in vacuo* and the resulting 1,3-dichloro-5-(chloromethyl)-2-methoxybenzene was used in the next step without further purification.

The N^9^ alkylation was performed following General procedure 1 using 2,6-dichloropurine **44** (0.457 g, 2.42 mmol) and 1,3-dichloro-5-(chloromethyl)-2-methoxybenzene (0.449 g, 2.54 mmol). The crude product was purified using flash column chromatography (SiO_2_; EtOAc/Hept = 1:1) and the desired compound was obtained as a white solid (0.325 g, 35%). ^1^H NMR (400 MHz, DMSO – *d*^6^) δ 8.81 (s, 1H), 7.54 (s, 2H), 5.46 (s, 2H), 3.80 (t, 3H); ^13^C NMR (101 MHz, DMSO – *d*^6^) δ 153.5, 151.4, 151.1, 149.8, 148.3, 133.7, 130.7, 128.8, 128.5, 60.6, 45.6.

The final compound **35** was prepared following the General method 2 for S_N_Ar starting from corresponding 9-alkyl-2,6-dichloro-9*H*-purine (0.080 g, 0.211 mmol). The crude product was purified using flash column chromatography (SiO_2_; EtOAc/Hept = 1.1:1) and the desired compound was obtained as a white solid (0.064 g, 81%). ^1^H NMR (400 MHz, DMSO – *d*^6^) δ 8.29–8.23 (bs, 1H), 8.25 (s, 1H), 7.45 (s, 2H), 5.31 (s, 2H), 3.80 (s, 3H), 2.92 (d, *J* = 4.7 Hz), 1H. ^13^C NMR (101 MHz, DMSO – *d*^6^) δ 155.6, 153.5, 151.2, 149.3, 141.1, 134.8, 128.5, 128.5, 118.3, 60.6, 44.9, 27.2. LRMS (ESI) *m*/*z*: [M + H]^+^ calcd for C_14_H_13_Cl_3_N_5_O; 372.018 found, 372.018.

##### 9-(3-Chlorobenzyl)-2-fluoro-*N*-methyl-9*H*-purin-6-amine (36) and 6-chloro-9-(3-chlorobenzyl)-*N*-methyl-9*H*-purin-2-amine (**39**)

The N^9^ alkylation was performed following the General procedure 1 using 6-chloro-2-fluoro-9*H*-purine **49** (0.578 g, 3.35 mmol) and 1-(bromomethyl)-3-chlorobenzene **50** (0.688 g, 3.35 mmol). The crude product was purified using flash column chromatography (SiO_2_; EtOAc/Hept = 1:1) and the desired compound was obtained as a white solid (0.178 g, 18%). ^1^H NMR (400 MHz, CDCl_3_ - *d*) δ 8.08 (s, 1H), 7.36–7.28 (m, 3H), 7.20 (dt, *J* = 6.7, 1.9 Hz, 1H), 5.36 (s, 2H). ^13^C NMR (101 MHz, CDCl_3_ - *d*) δ 157.66 (d, *J* = 220.8 Hz), 153.73 (d, *J* = 16.9 Hz), 153.24 (d, *J* = 17.5 Hz), 145.46 (d, *J* = 3.3 Hz), 136.1, 136.4, 130.8, 130.34 (d, *J* = 4.9 Hz), 129.5, 128.2, 126.2, 47.5.

The final compounds **36** and **39** were prepared following the General method 2 for S_N_Ar starting from corresponding 9-alkyl-2,6-dichloro-9*H*-purine (0.080 g, 0.211 mmol). The reaction provided both products that were separated using flash column chromatography (SiO_2_; EtOAc/Hept = 1.2:1 to 2:1) and the desired compound was obtained as a white solid (**36:** 0.021 g, 20%, **39**: 0.042 g, 40%). **36:**^1^H NMR (400 MHz, CDCl_3_ - *d*) δ 7.66 (s, 1H), 7.30–7.15 (m, 3H), 7.17–7.14 (m, 1H), 6.13–6.05 (bs, 1H), 5.26 (s, 2H), 3.24–3.09 (bs, 3H). ^13^C NMR (101 MHz, CDCl_3_ - *d*) δ 159.98 (d, *J* = 208.6 Hz), 157.31 (d, *J* = 20.5 Hz), 139.63 (d, *J* = 3.1 Hz), 137.5, 135.1, 130.5, 128.9, 127.9, 126.0, 118.2, 46.7, 27.8. ^19^F NMR (376 MHz, CDCl_3_ - *d*) δ −49.37. LRMS (ESI) *m*/*z*: [M + H]^+^ calcd for C_13_H_12_ClFN_5_; 292.076 found, 292.077. **39**: ^1^H NMR (400 MHz, CDCl3) δ 7.70 (s, 1H), 7.34–7.27 (m, 3H), 7.18–7.14 (m, 1H), 5.28–5.22 (bs, 1H), 5.23 (s, 2H), 3.04 (d, *J* = 5.0 Hz, 1H). ^13^C NMR (101 MHz, CDCl_3_ - *d*) δ 160.0, 154.0, 151.5, 141.2, 137.5, 135.1, 130.5, 128.9, 128.3, 126.1, 124.4, 46.7, 29.0. LRMS (ESI) *m*/*z*: [M + H]^+^ calcd for C_13_H_12_Cl_2_N_5_; 308.046 found, 308.048.

##### 2-Chloro-9-(3-chlorobenzyl)-*N*-cyclopropyl-9*H*-purin-6-amine (**37**)

The compound was prepared following the general method for S_N_Ar starting from corresponding 9-alkyl-2,6-dichloro-9*H*-purine (0.07 g, 0.223 mmol) and cyclopropylamine (0.03 mL, 0.433 mmol). The crude product was purified using flash column chromatography (SiO_2_; EtOAc/Hept = 3:1) and the desired compound was obtained as a white solid (0.071 g, 95%). ^1^H NMR (400 MHz, CDCl_3_ - *d*) δ 7.66 (s, 1H), 7.32–7.28 (m, 2H), 7.24 (s, 1H), 7.17–7.12 (m, 1H), 6.18–5.95 (bs, 1H), 5.29 (s, 2H), 3.25–2.98 (bs, 1H), 1.87 (s, 1H), 0.96–0.91 (m, 2H), 0.67–0.63 (m, 2H); ^13^C NMR (101 MHz, CDCl_3_ - *d*) δ 156.6, 155.0, 140.00, 137.4, 135.1, 130.6, 128.9, 128.0, 126.1, 118.7, 46.7, 24.2, 7.7. LRMS (ESI) *m*/*z*: [M + H]^+^ calcd for C_15_H_14_Cl_2_N_5_; 334.062 found, 334.062.

##### 2,6-Dichloro-9-(3-chlorobenzyl)-9*H*-purine (**38**)

The N^9^ alkylation was performed following General procedure 1 using 2,6-dichloropurine **44** (0.5 g, 2.65 mmol) and 1-bromomethyl-3-chlorbenzen (0.543 g, 2.65 mmol). The crude product was purified using flash column chromatography (SiO_2_; EtOAc/Hept = 2:1) and the desired compound was obtained as a white solid (0.495 g, 59%). ^1^H NMR (400 MHz, CDCl_3_ - *d*) δ 8.07 (s, 1H), 7.37–7.29 (m, 3H), 7.19 (dt, *J* = 6.8, 1.8 Hz, 1H), 5.39 (s, 2H). ^13^C NMR (101 MHz, CDCl_3_ - *d*) δ 153.6, 153.2, 152.3, 145.4, 136.1, 135.5, 130.9, 130.8, 129.5, 128.2, 126.2, 47.5. LRMS (ESI) *m*/*z*: [M + H]^+^ calcd for C_12_H_8_Cl_3_N_4_; 312.981 found, 312.982.

## Supporting Information Available

Kinase panel, and dose–response curves for antiproliferative effect; HTRF and TSA dose–response curves; isothermal titration calorimetry curve; ^1^H, ^19^F, and ^13^C NMR spectra of final molecules and the HPLC purity trace (PDF)
Molecular formula strings (CSV)

## Abbreviations Used

AML: acute myeloid leukemia
COMU: (1-cyano-2-ethoxy-2-oxoethylidenaminooxy)dimethylamino-morpholino-carbenium hexafluorophosphate
DIPEA: *N*,*N*-diisopropylethylamine
HTRF assay: homogeneous time-resolved fluorescence
ITC: isothermal titration calorimetry
LE: ligand efficiency
LLE: lipophilic ligand efficiency
MS: molecular sieves
PARP: poly(ADP-ribose) polymerase
PBMCs: peripheral blood mononuclear cells
pTSA: *para*-toluenesulfonic acid
S_N_Ar: nucleophilic aromatic substitution
TSA: thermal shift assay
