# Supplementary material for: Structure-Based Design of a Potent and Selective YTHDC1 Ligand
Source: J Med Chem. 2024 May 24;67(11):9516–35. doi: 10.1021/acs.jmedchem.4c00599 (PMC11181329; doi:10.1021/acs.jmedchem.4c00599)

# Supporting information

# Structure-based design of a potent and selective YTHDC1 ligand

*František Zálešák, Francesco Nai, Marcin Herok, Elena Bochenkova, Rajiv K. Bedi, Yaozong Li, Francesco Errani, Amedeo Caflisch**

Department of Biochemistry, University of Zurich

Winterthurerstrasse 190, CH-8057 Zurich, Switzerland

*To whom correspondence should be addressed. Tel: +41 44 635 5521; email: caflisch@bioc.uzh.ch

## Table of Contents


1. **Figure S1:** Inhibitory activity of compound **40** at 2 μM on a panel of 58 kinases

2. **Figure S2:** Dose-response curve for the antiproliferative effect of compound **40** against MOLM-13, NOMO-1 and HEK293T cell line.

3. **Figure S3:** Representative Western blot membranes for the data presented in Figure 4 (A) CETSA YTHDC1 (B) Cleaved PARP

4. **Figure S4:** HTRF dose-response curves of compound **40** against YTHDF1, YTHDF2, and YTHDF3, respectively.

5. **Figure S5:** Dose-response thermal shift of YTHDF1, YTHDF2, YTHDF3, and YTHDC2 in presence of compound **40**

6. **GST-YTHDC1 HTRF dose-response curves**

7. **Figure S6:** Isothermal titration calorimetry curve of compound **3**

8. **NMR traces of final compounds**

9. **HPLC traces of final compounds**

1. **Figure S1** - Inhibitory activity of compound **40** at 2 μM on a panel of 58 kinases

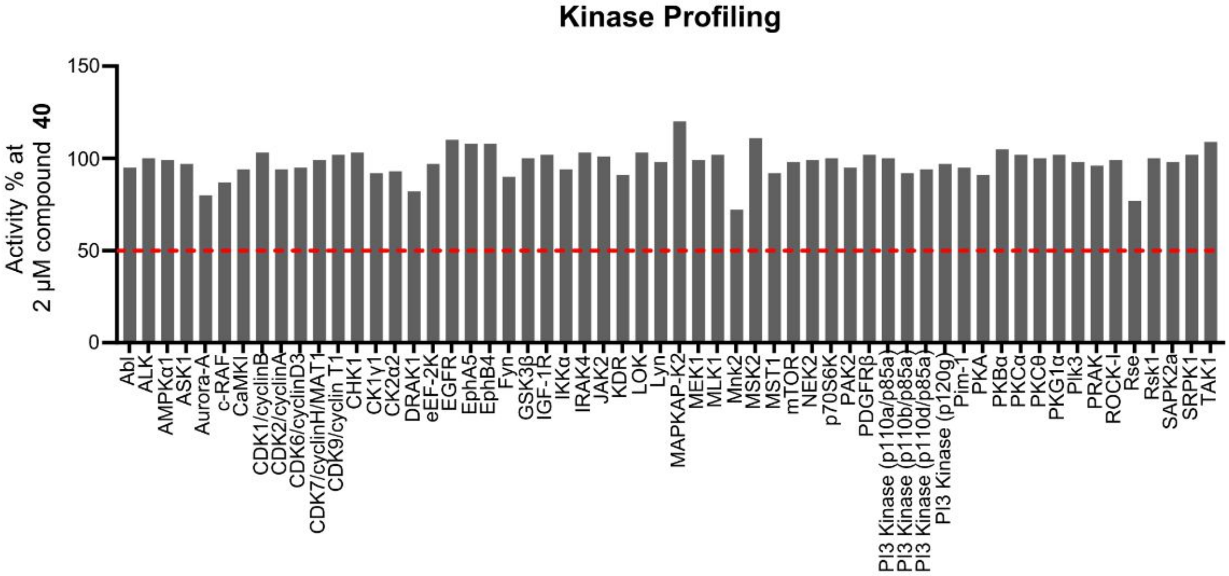


2. **Figure S2:** Dose-response curve for the antiproliferative effect of compound **40** against (A) MOLM-13, NOMO-1 and (B) HEK293T cell lines.

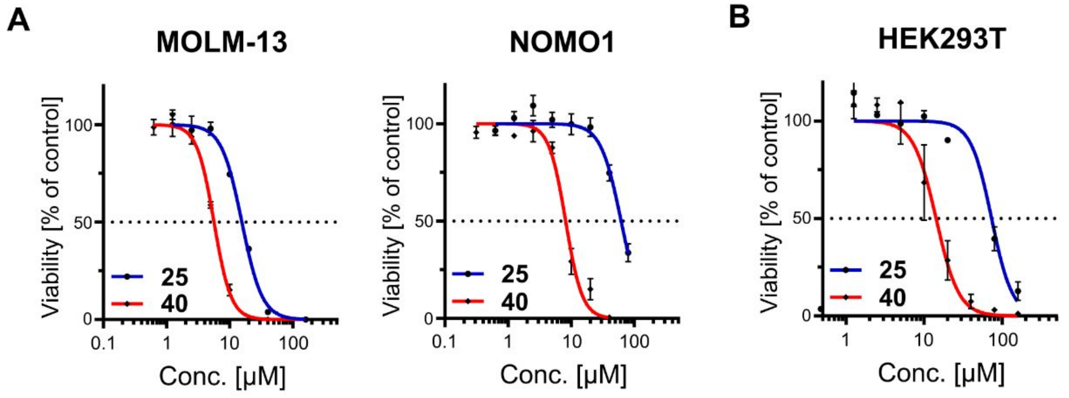


| | 25 | 40 |
|---|---|---|
| IC50 | 15.6 | 5.6 |
| HillSlope | -2.7 | -3.2 |

| | 25 | 40 |
|---|---|---|
| IC50 | 61.6 | 8.2 |
| HillSlope | -2.7 | -3.3 |

| | 25 | 40 |
|---|---|---|
| IC50 | 73.5 | 14.3 |
| HillSlope | -3.3 | -3.0 |

3. **Figure S3:** Representative Western blot membranes for the data presented in Figure 4. (A) CETSA YTHDC1 (B) Cleaved PARP

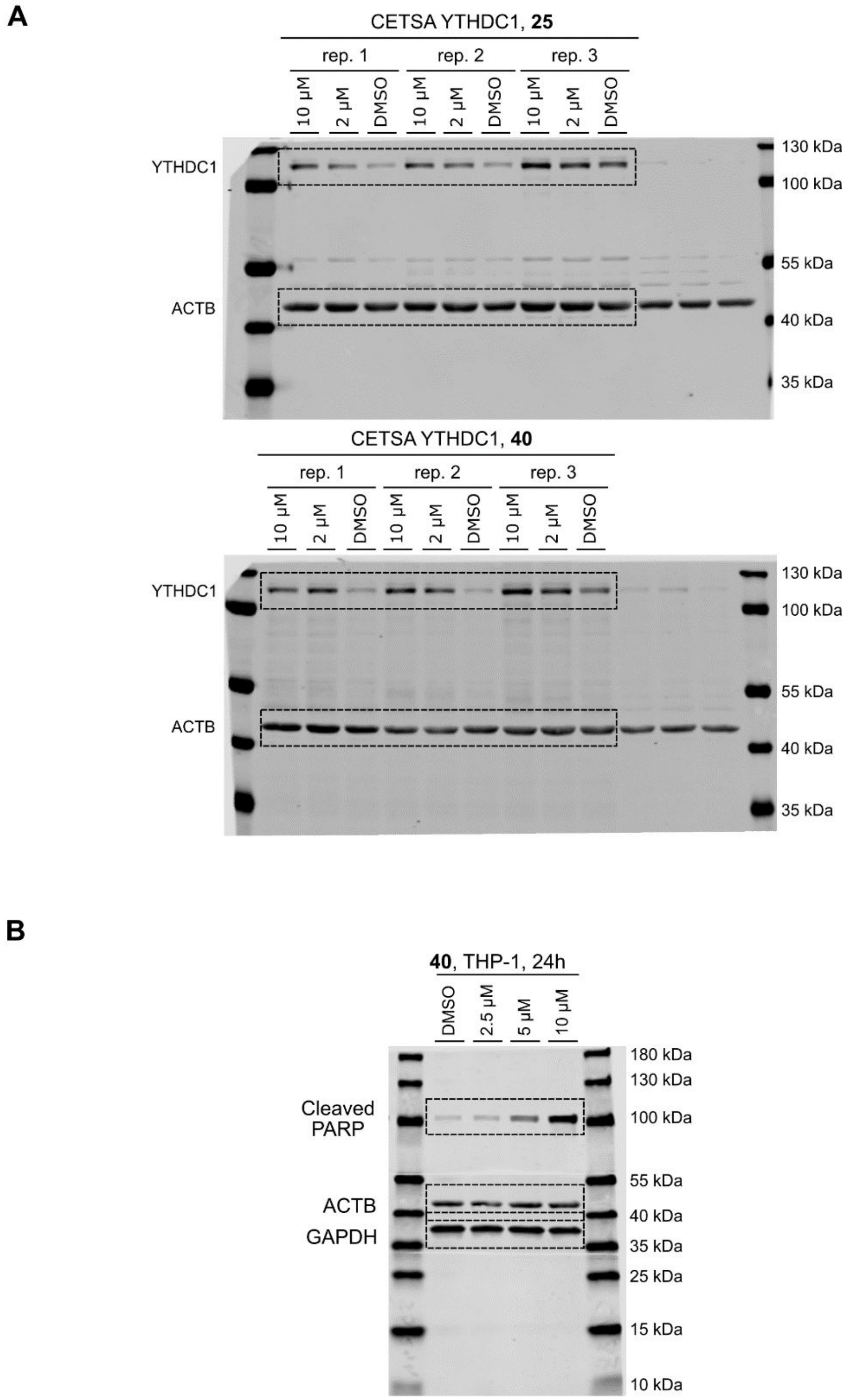

4. **Figure S4:** HTRF dose-response curves of compound **40** against YTHDF1, YTHDF2, and YTHDF3, respectively.

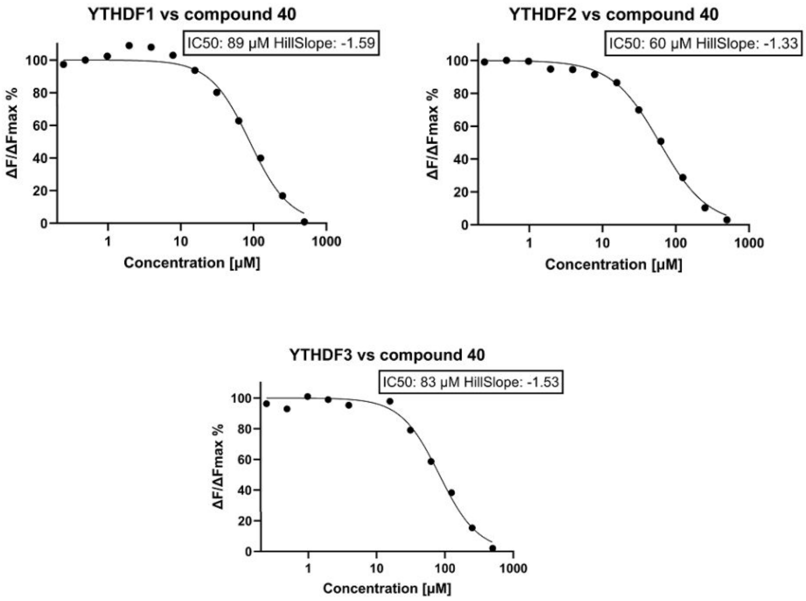


5. **Figure S5:** Dose-response thermal shift of YTHDF1, YTHDF2, YTHDF3, and YTHDC2 in presence of compound **40.**

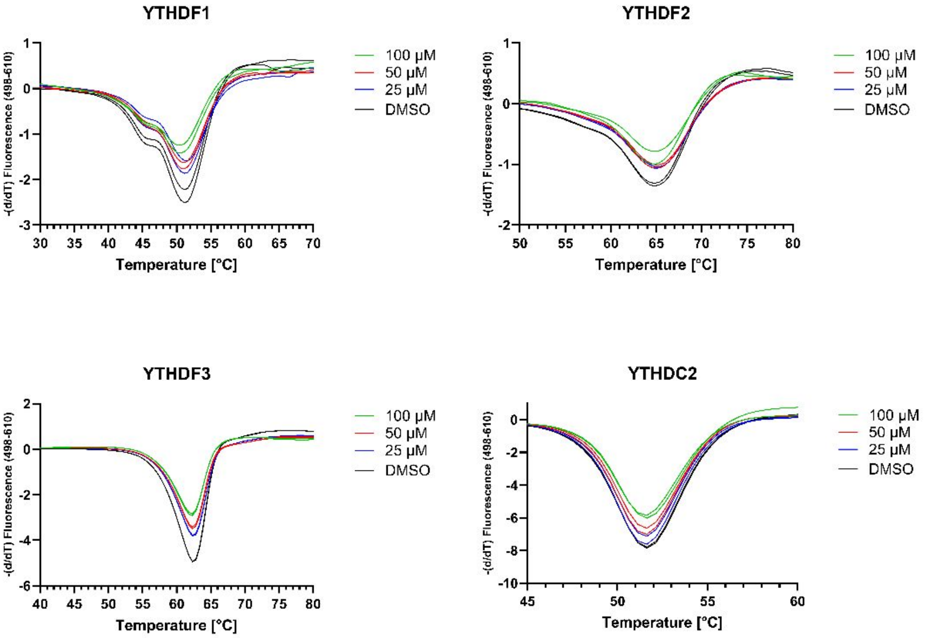

6. **GST-YTHDC1 HTRF dose-response curves.** With the exceptions of compounds **2b**, **4**, **7**, **8**, **9**, **15**, **24**, **29**, **33**, and **39** the curves come from the average of two or more biological replicates, and each biological replicate is the average of two technical replicates. The error bars represent the standard deviation for the biological triplicates and quadruplicate. Compound **38** was only tested in single dose at 5 micromolar (103%).

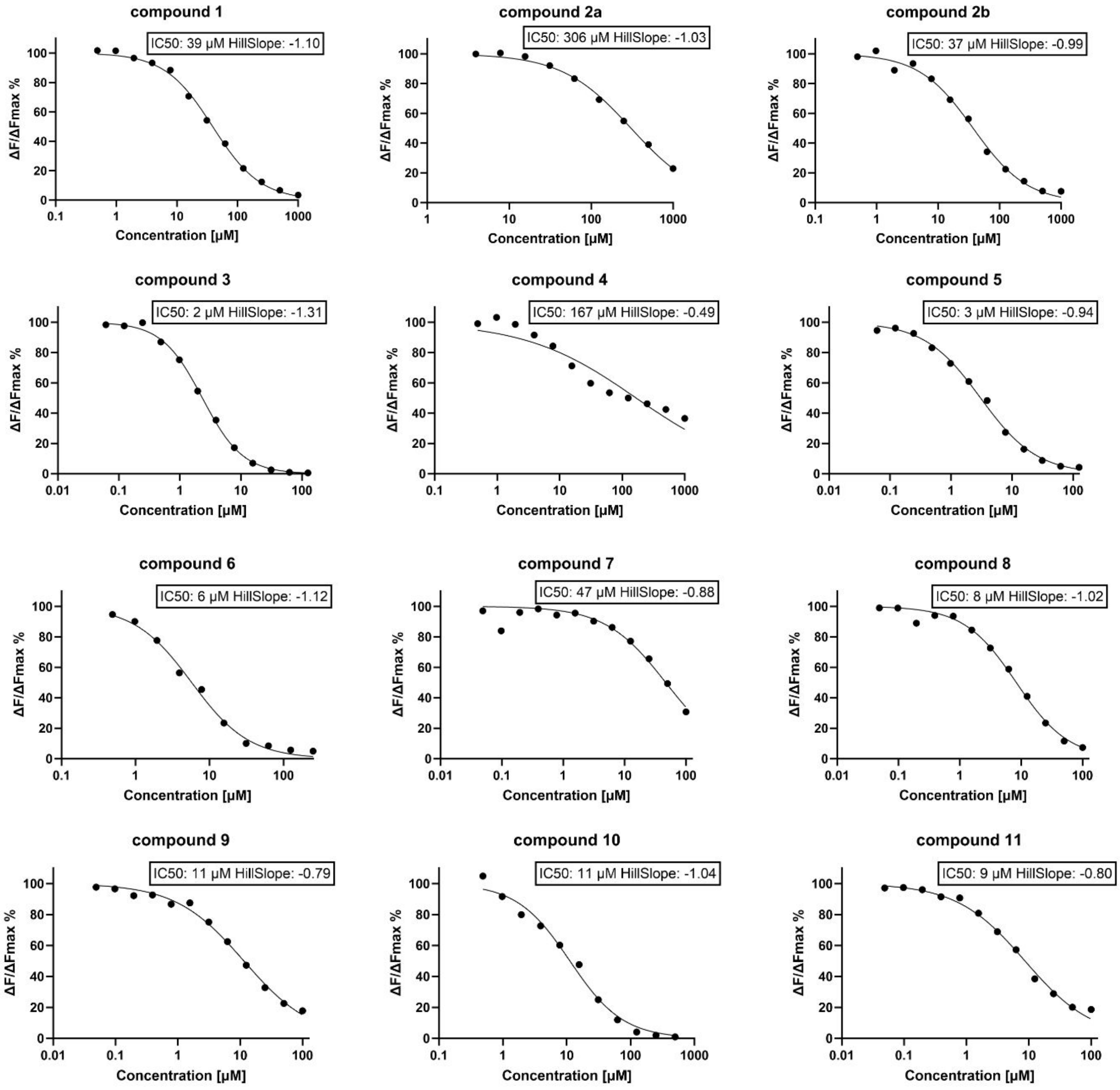

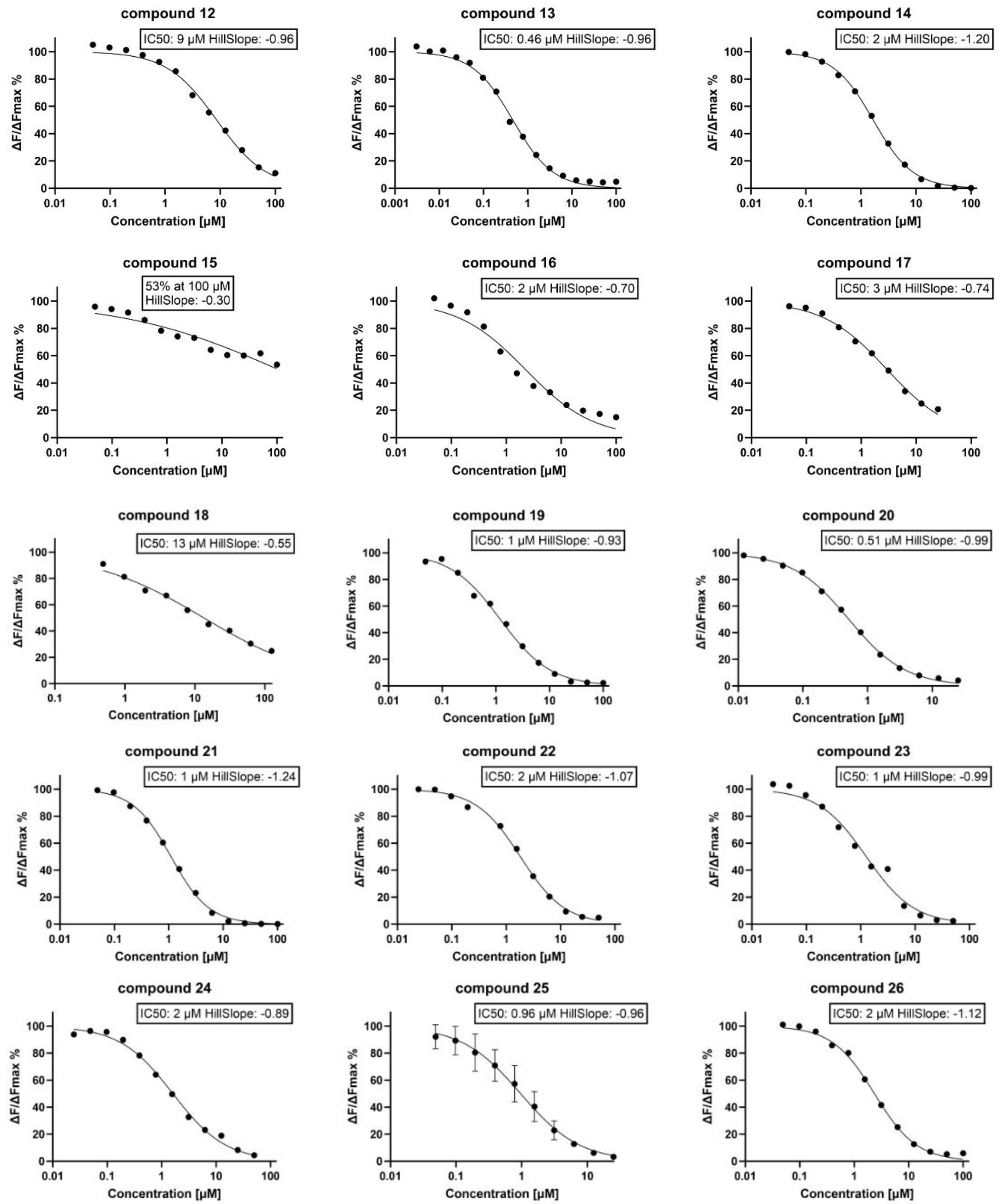
compound 12
IC50: 9 μM HillSlope: -0.96
compound 13
IC50: 0.46 μM HillSlope: -0.96
compound 14
IC50: 2 μM HillSlope: -1.20
compound 15
53% at 100 μM
HillSlope: -0.30
compound 16
IC50: 2 μM HillSlope: -0.70
compound 17
IC50: 3 μM HillSlope: -0.74
compound 18
IC50: 13 μM HillSlope: -0.55
compound 19
IC50: 1 μM HillSlope: -0.93
compound 20
IC50: 0.51 μM HillSlope: -0.99
compound 21
IC50: 1 μM HillSlope: -1.24
compound 22
IC50: 2 μM HillSlope: -1.07
compound 23
IC50: 1 μM HillSlope: -0.99
compound 24
IC50: 2 μM HillSlope: -0.89
compound 25
IC50: 0.96 μM HillSlope: -0.96
compound 26
IC50: 2 μM HillSlope: -1.12
ΔF/ΔFmax %
Concentration [μM]

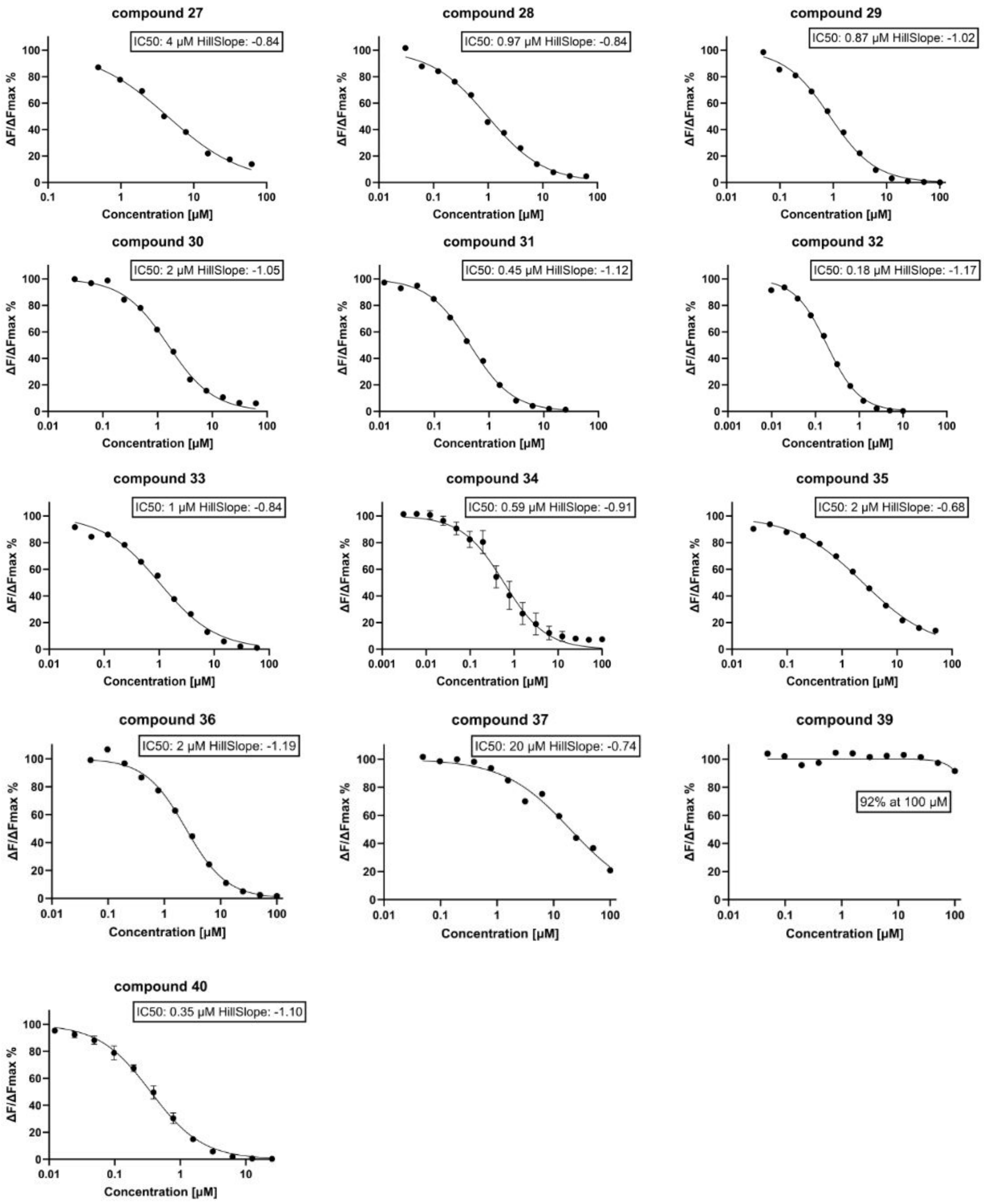
compound 27
IC50: 4 μM HillSlope: -0.84
compound 28
IC50: 0.97 μM HillSlope: -0.84
compound 29
IC50: 0.87 μM HillSlope: -1.02
compound 30
IC50: 2 μM HillSlope: -1.05
compound 31
IC50: 0.45 μM HillSlope: -1.12
compound 32
IC50: 0.18 μM HillSlope: -1.17
compound 33
IC50: 1 μM HillSlope: -0.84
compound 34
IC50: 0.59 μM HillSlope: -0.91
compound 35
IC50: 2 μM HillSlope: -0.68
compound 36
IC50: 2 μM HillSlope: -1.19
compound 37
IC50: 20 μM HillSlope: -0.74
compound 39
92% at 100 μM
compound 40
IC50: 0.35 μM HillSlope: -1.10
ΔF/ΔFmax %
Concentration [μM]

7. Figure S6: Isothermal titration calorimetry curve of compound 3

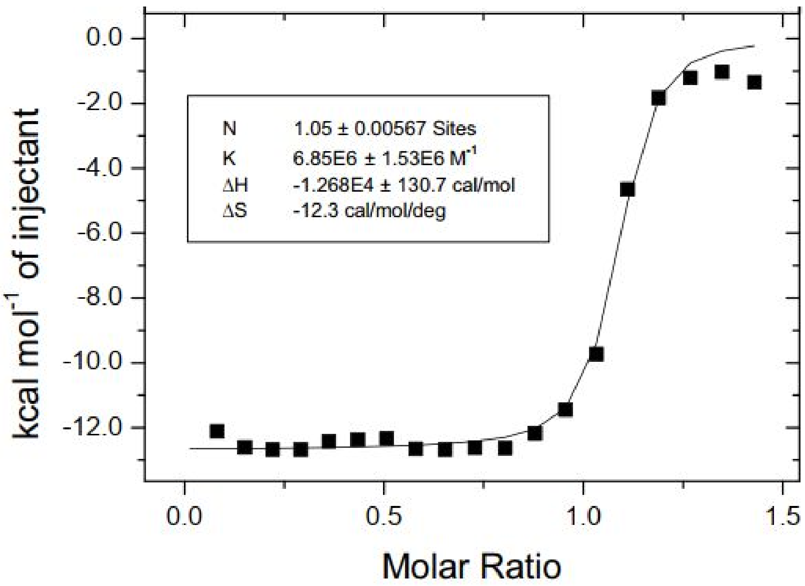

## 8. NMR traces of final compounds

### 5-chloro-N,3-dimethyl-1H-pyrazolo[4,3-d]pyrimidin-7-amine 3

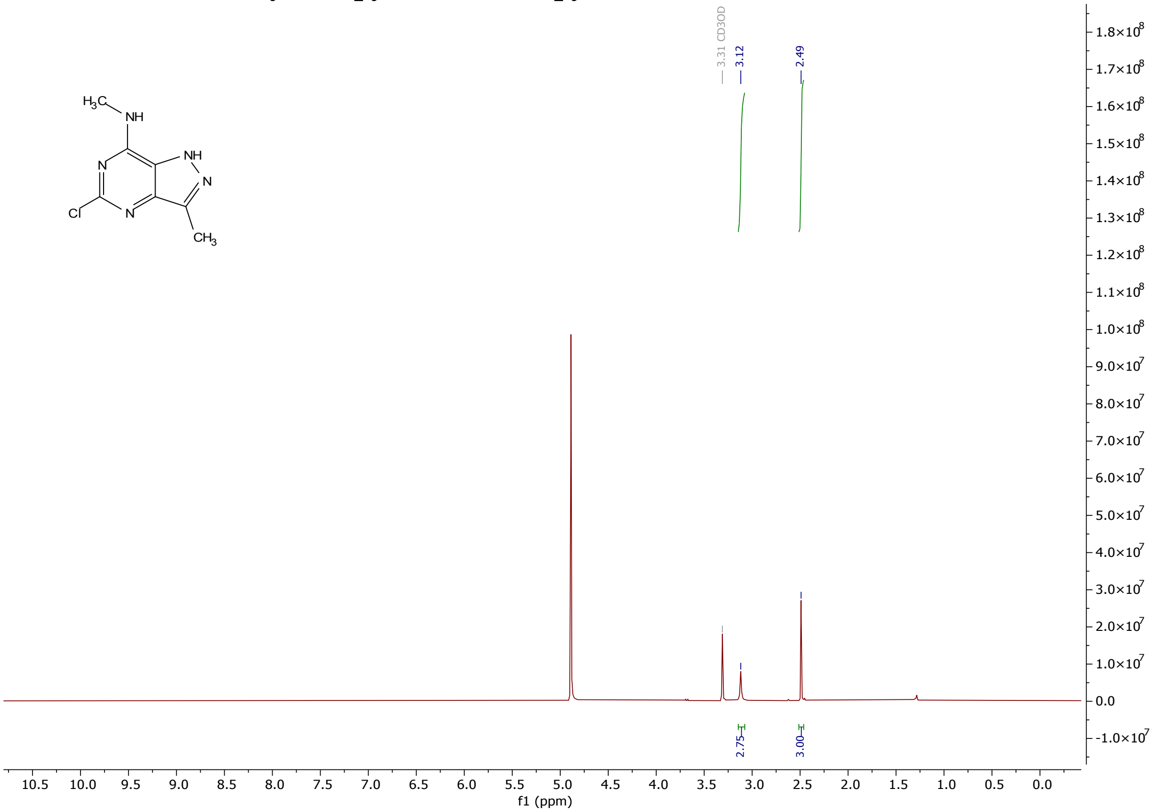


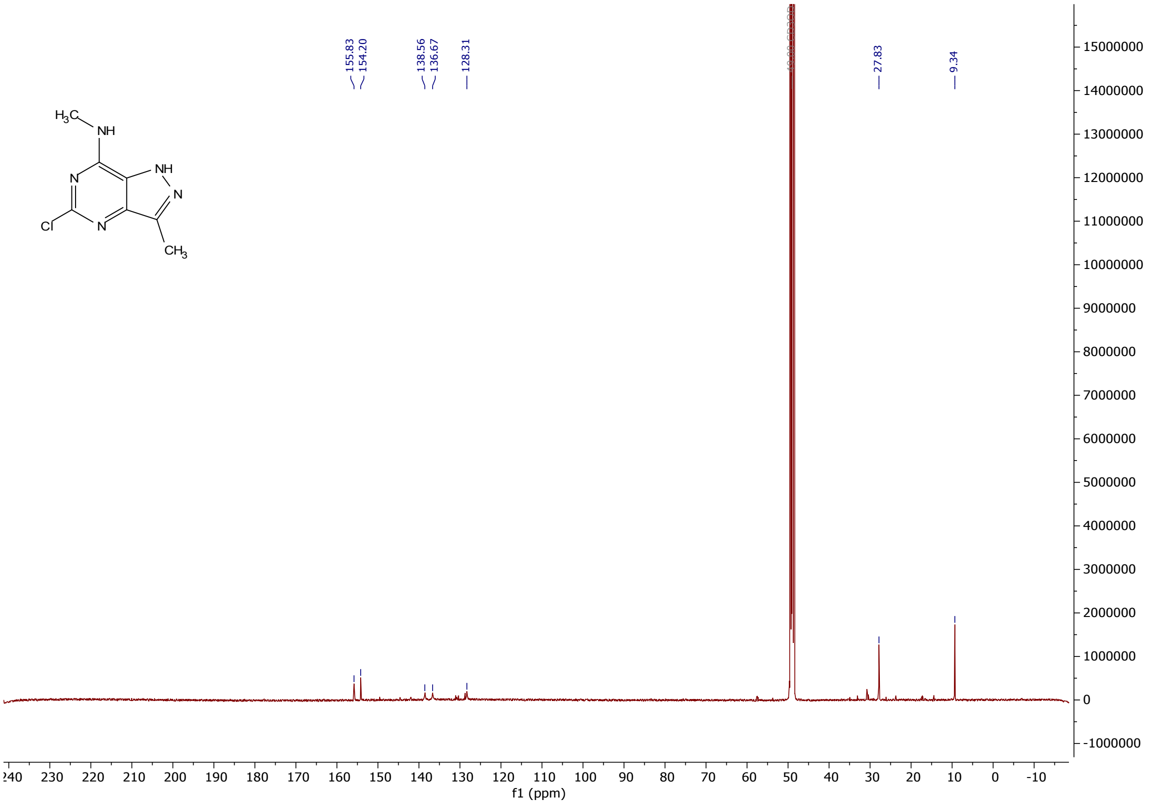

# 2-chloro-N-methyl-9-phenyl-9H-purin-6-amine 4

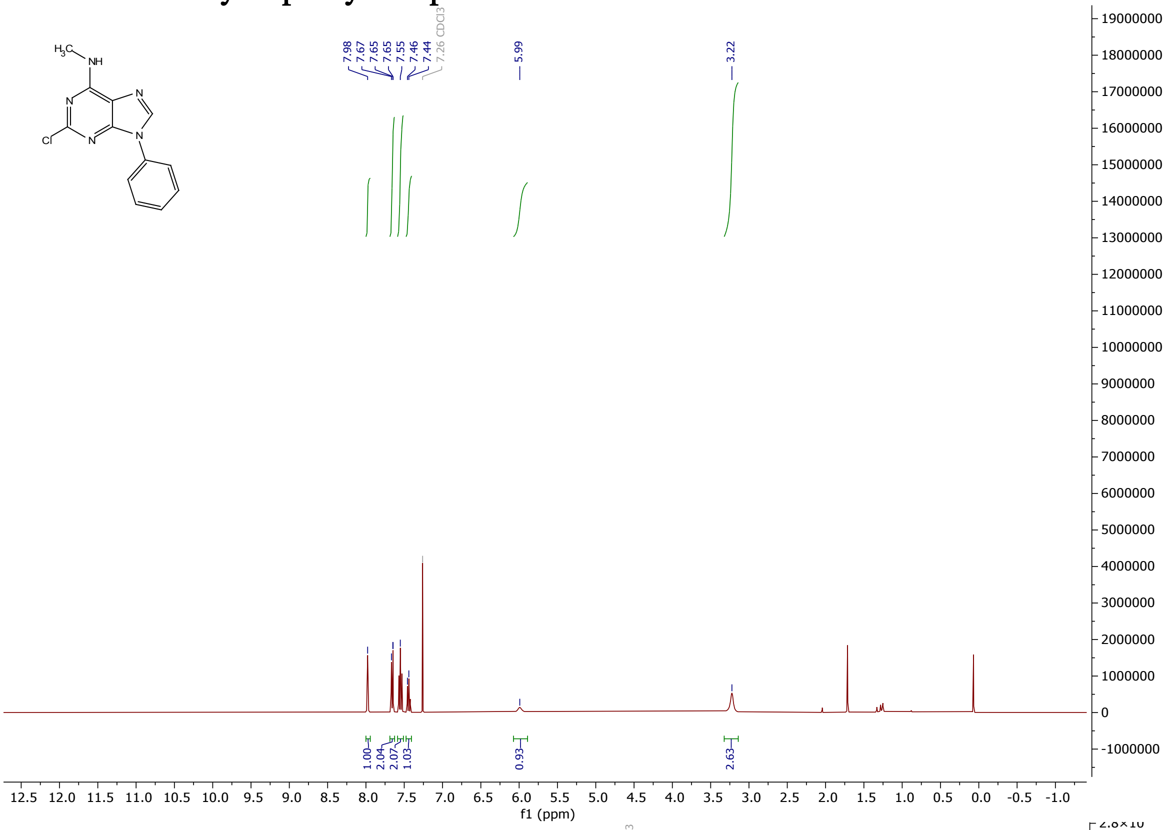


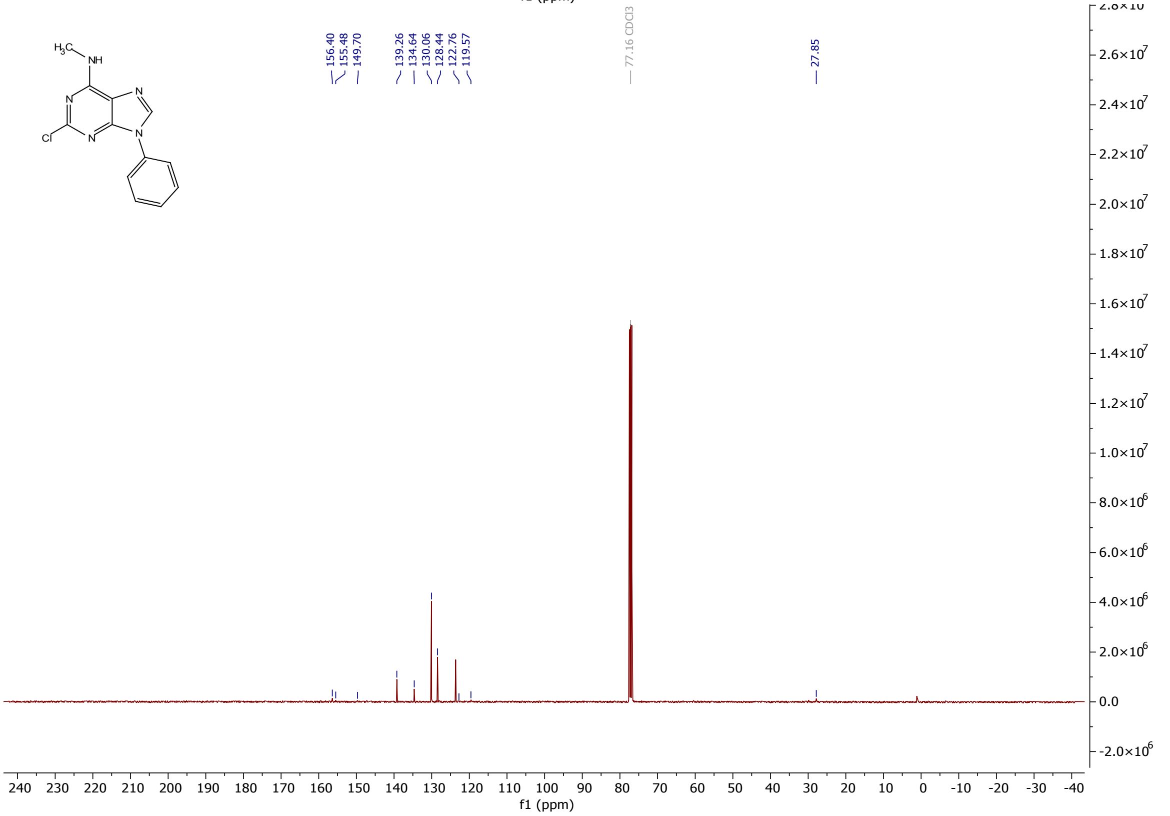

# 9-benzyl-2-chloro-N-methyl-9H-purin-6-amine 5

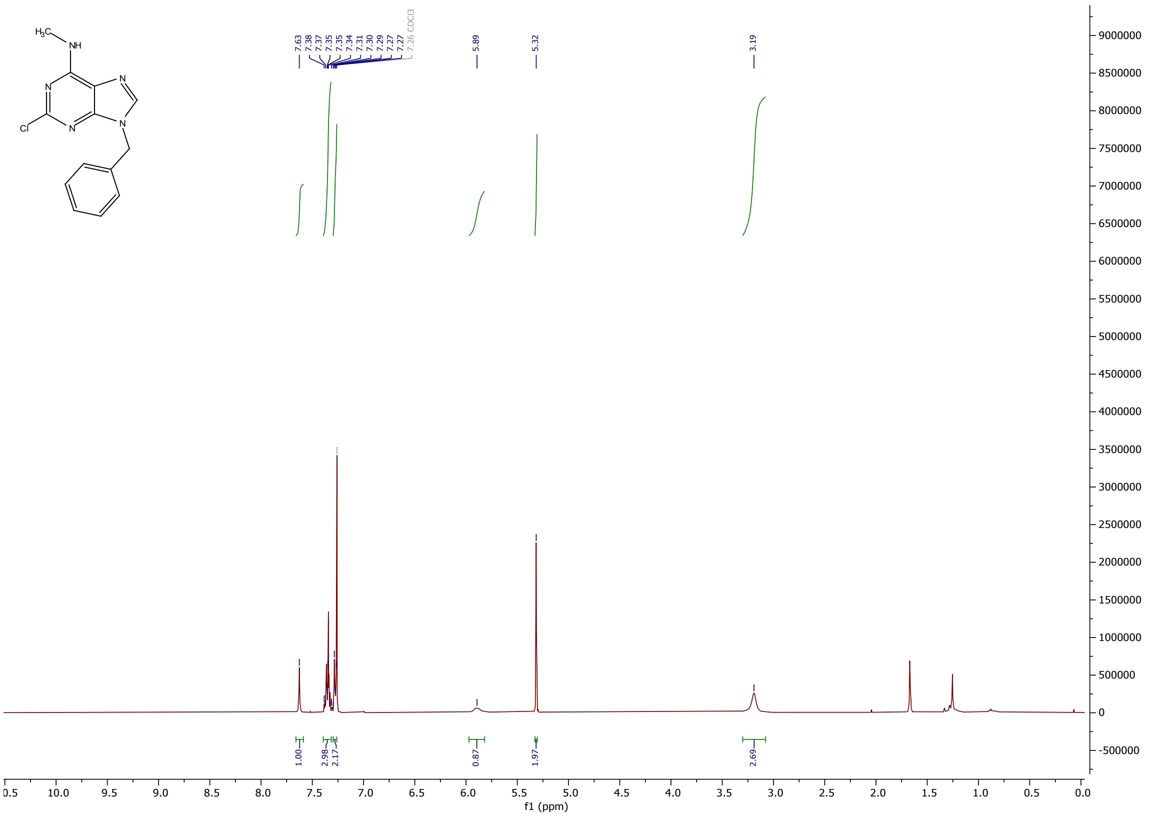


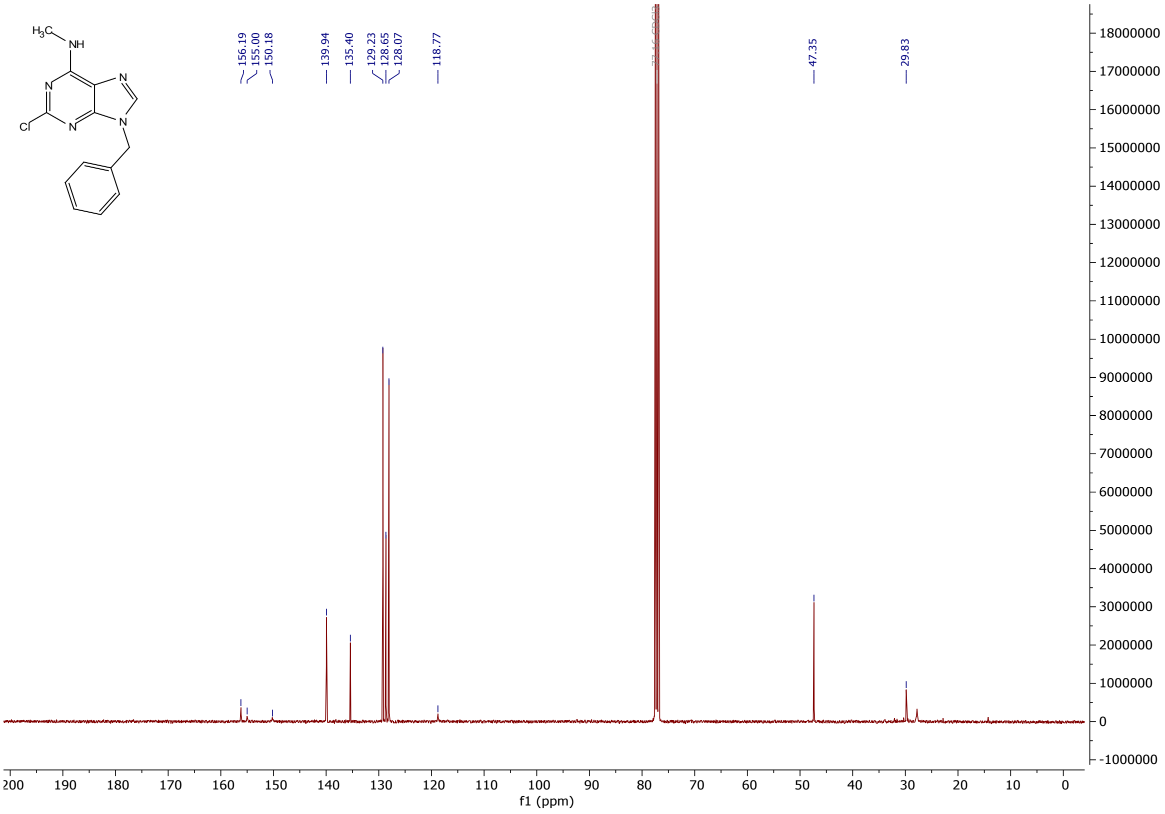

# 2-chloro-N-methyl-9-(pyridin-4-ylmethyl)-9H-purin-6-amine 6

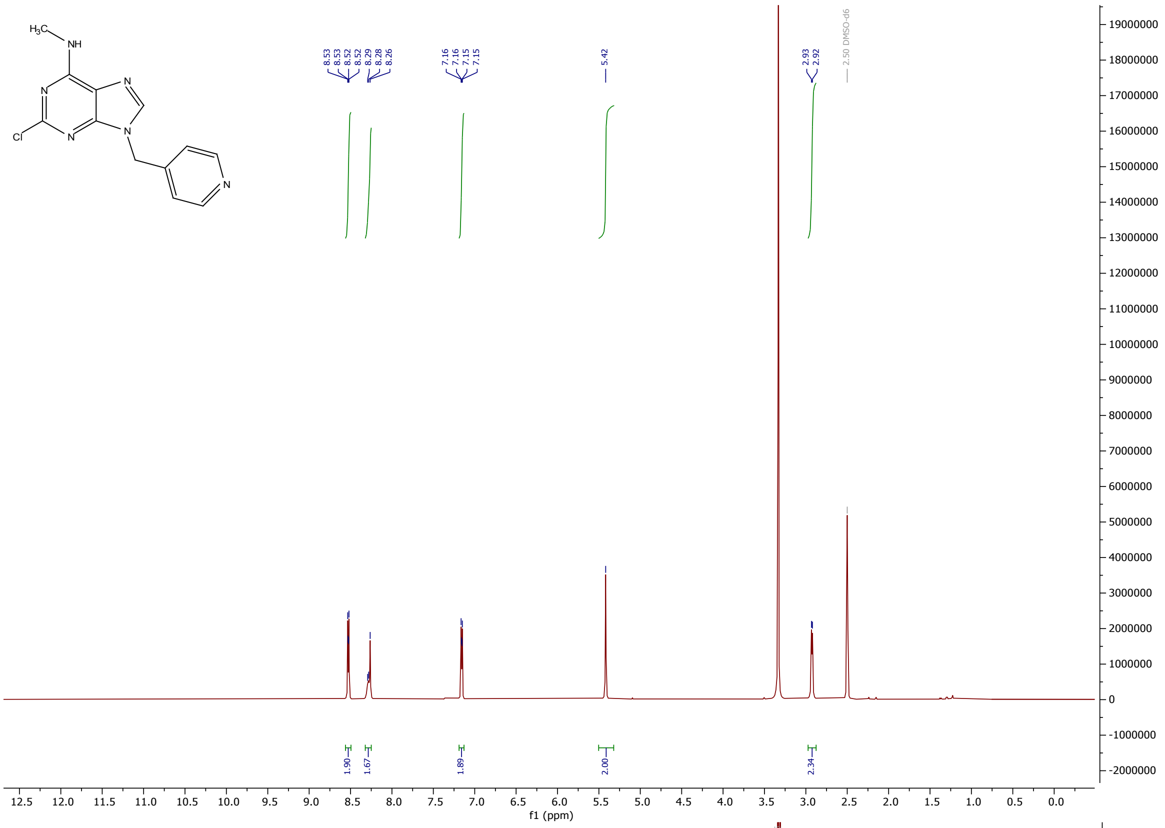


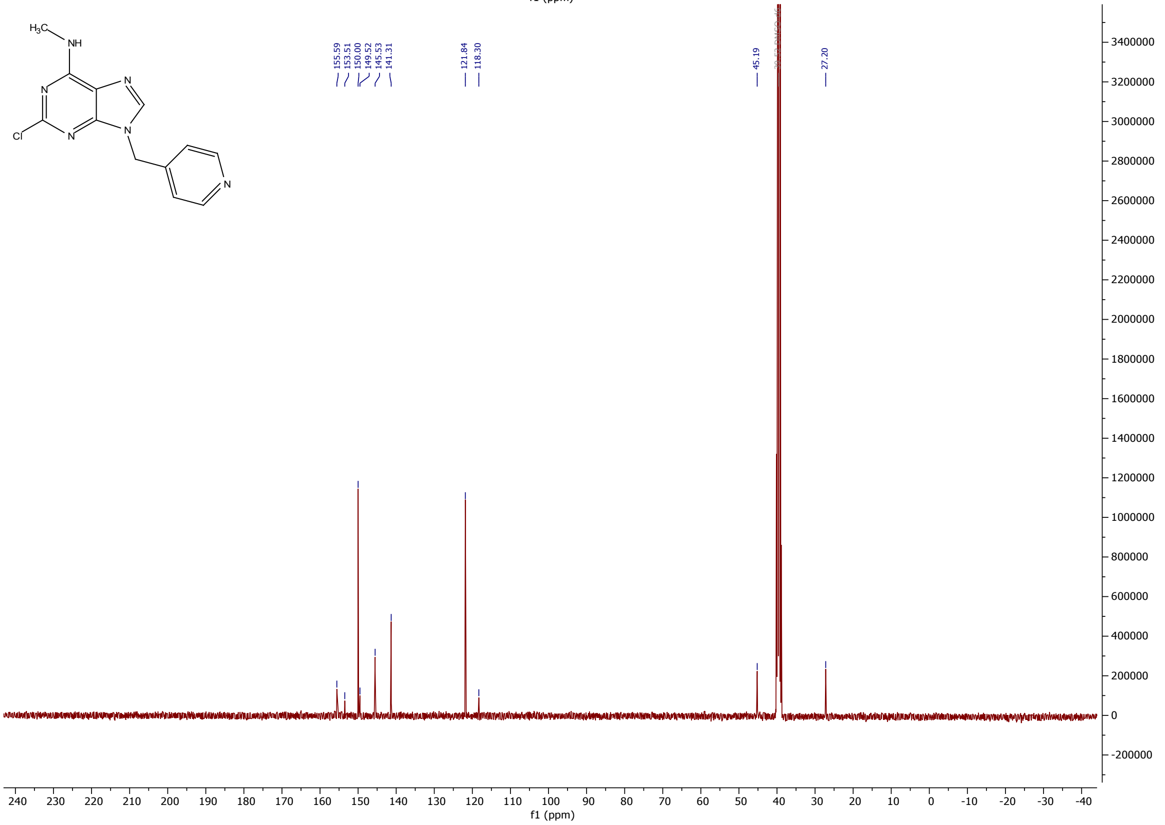

# 2-chloro-N-methyl-9-(tetrahydro-2H-pyran-2-yl)-9H-purin-6-amine 7

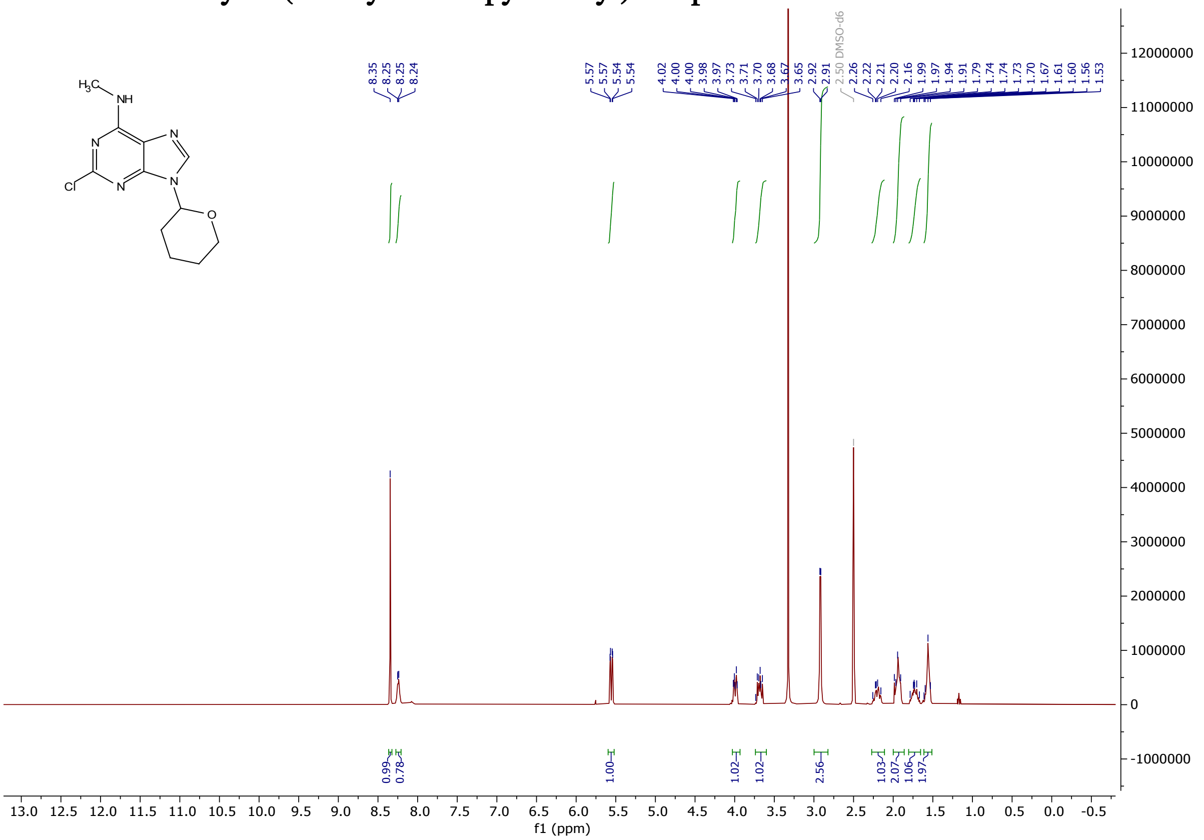


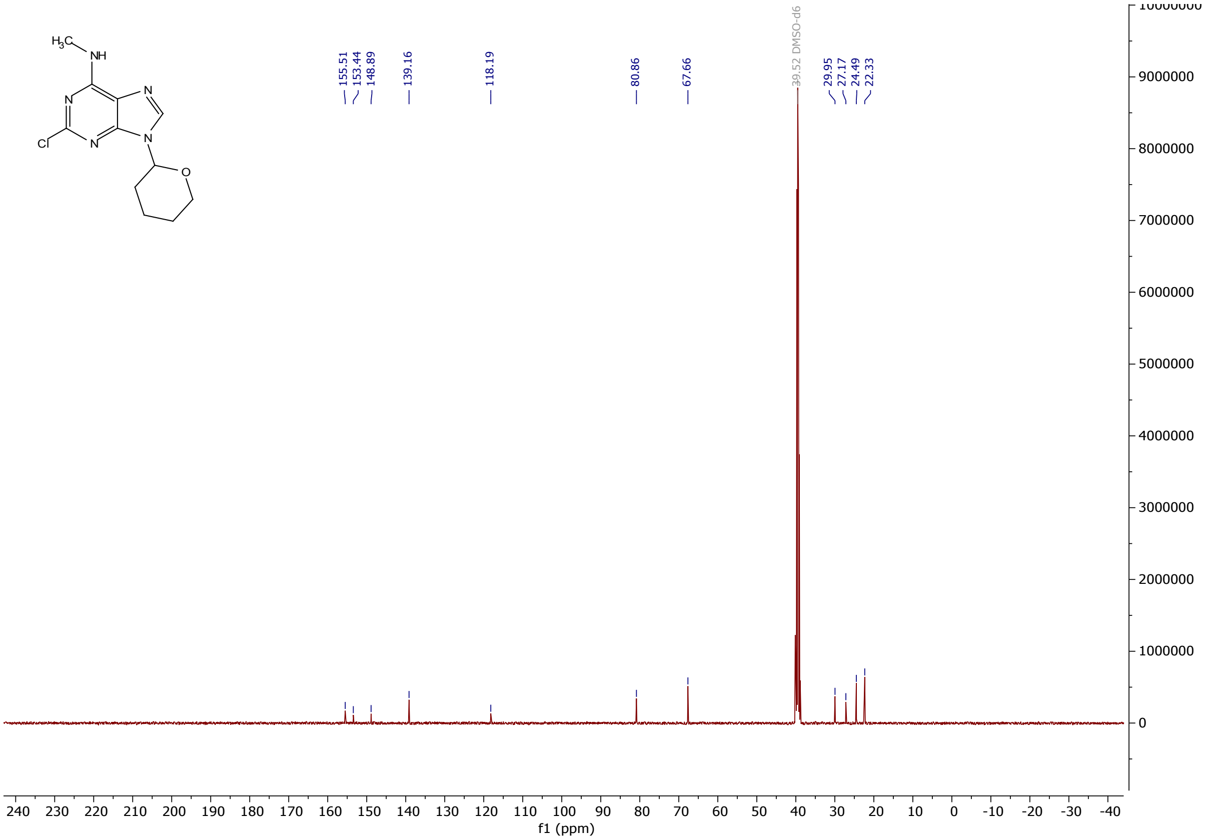

# 3-(2-chloro-6-(methylamino)-9H-purin-9-yl)benzoic acid 10

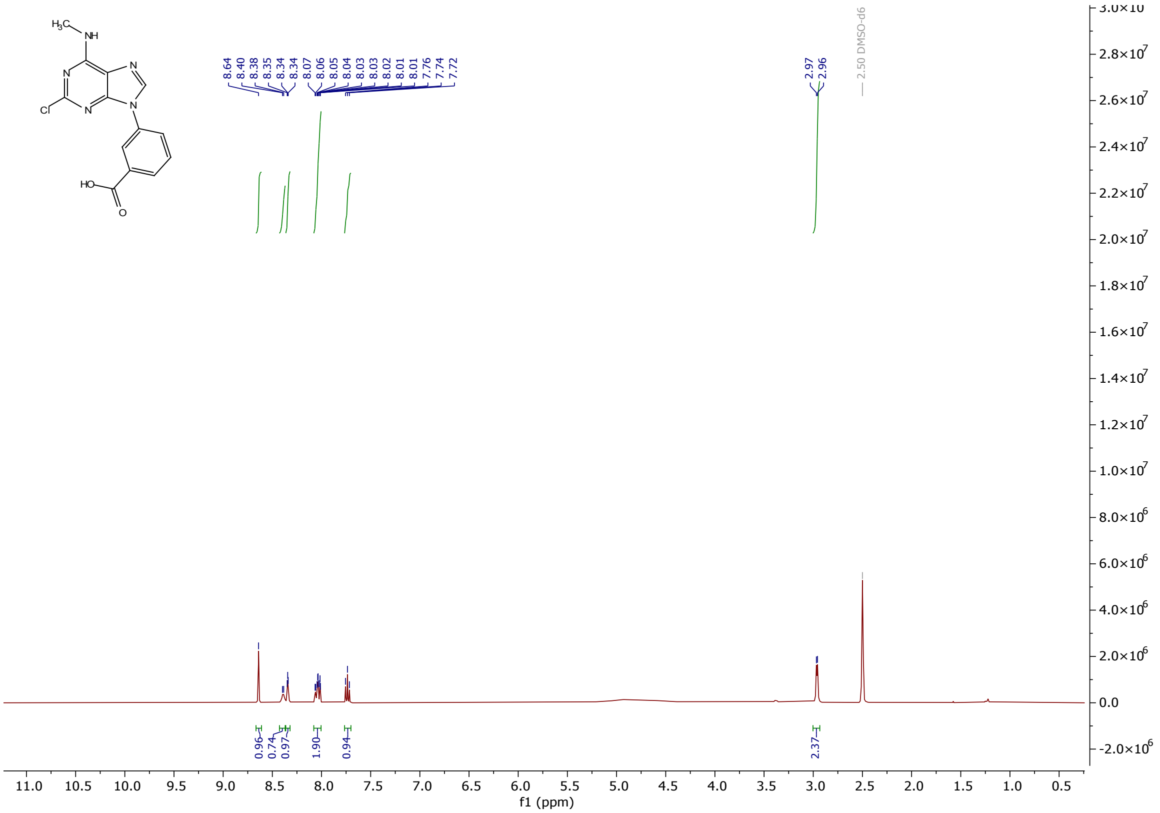


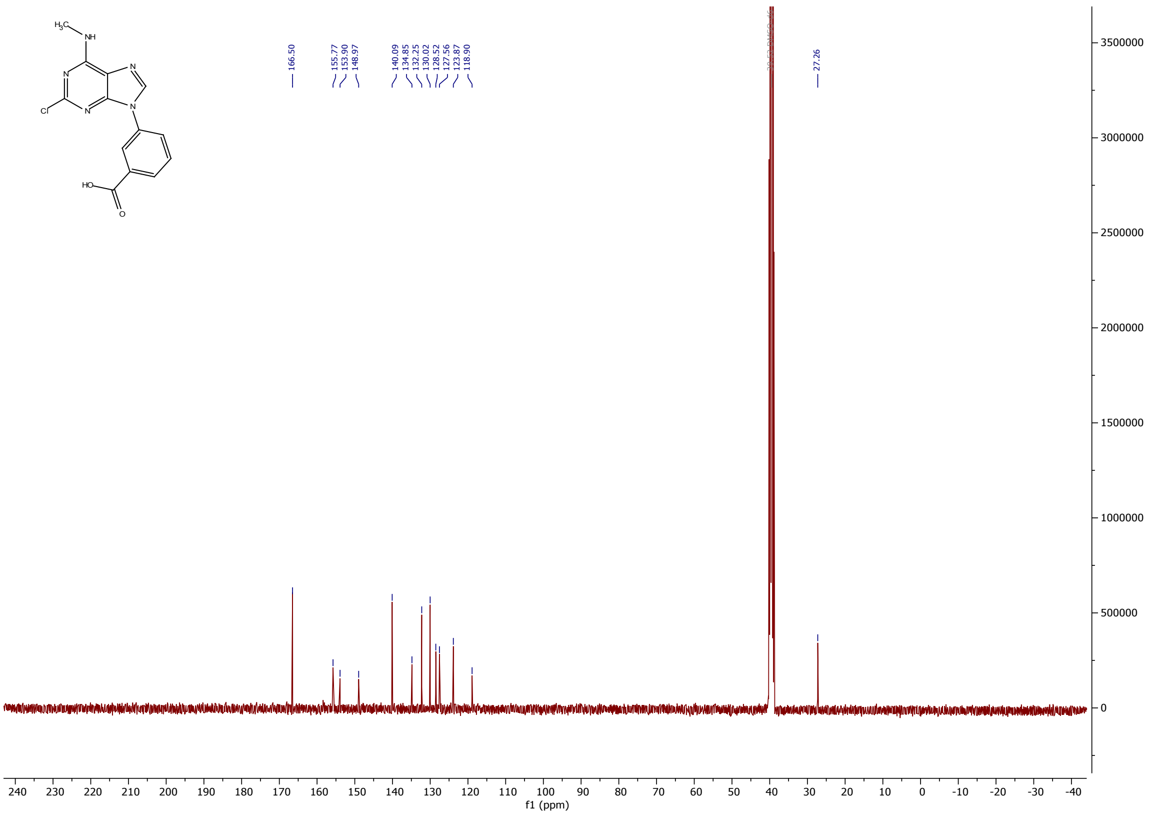

# 2-(2-chloro-6-(methylamino)-9H-purin-9-yl)-N-phenylacetamide 11

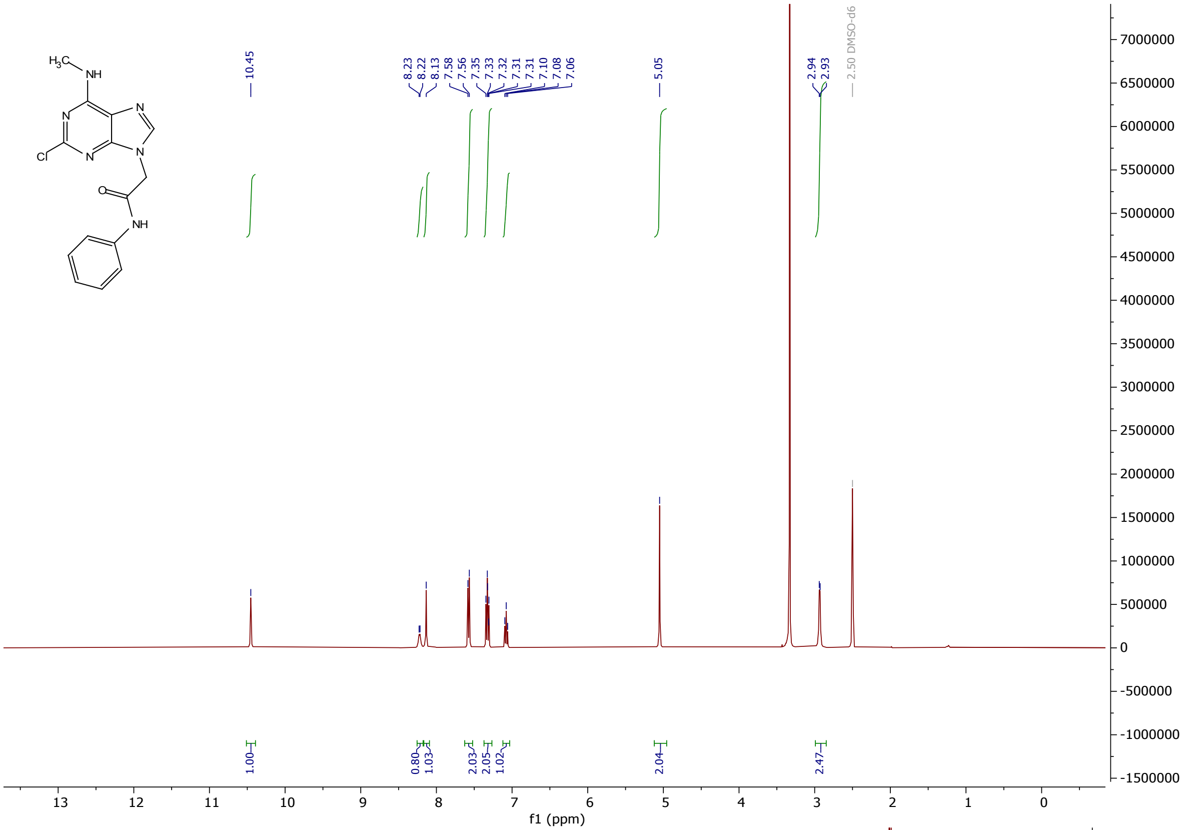


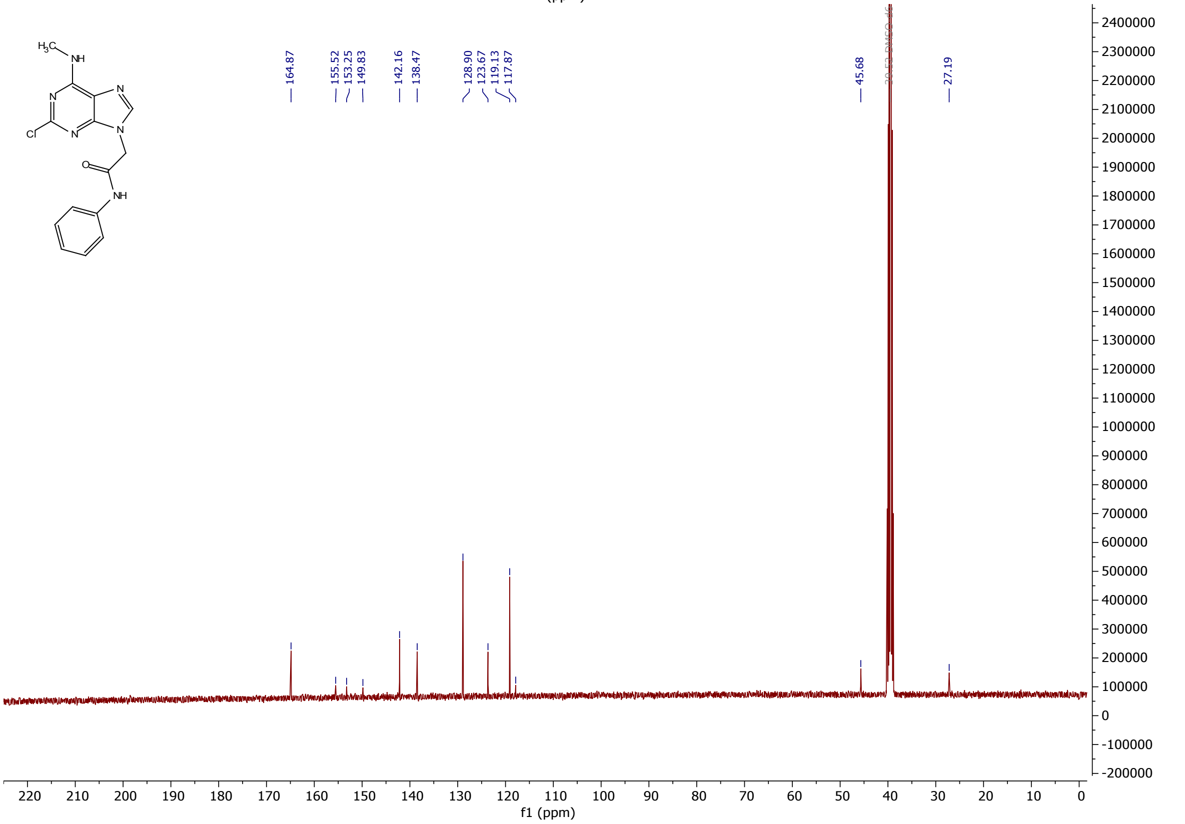

# 9-(2-aminobenzyl)-2-chloro-N-methyl-9H-purin-6-amine 12

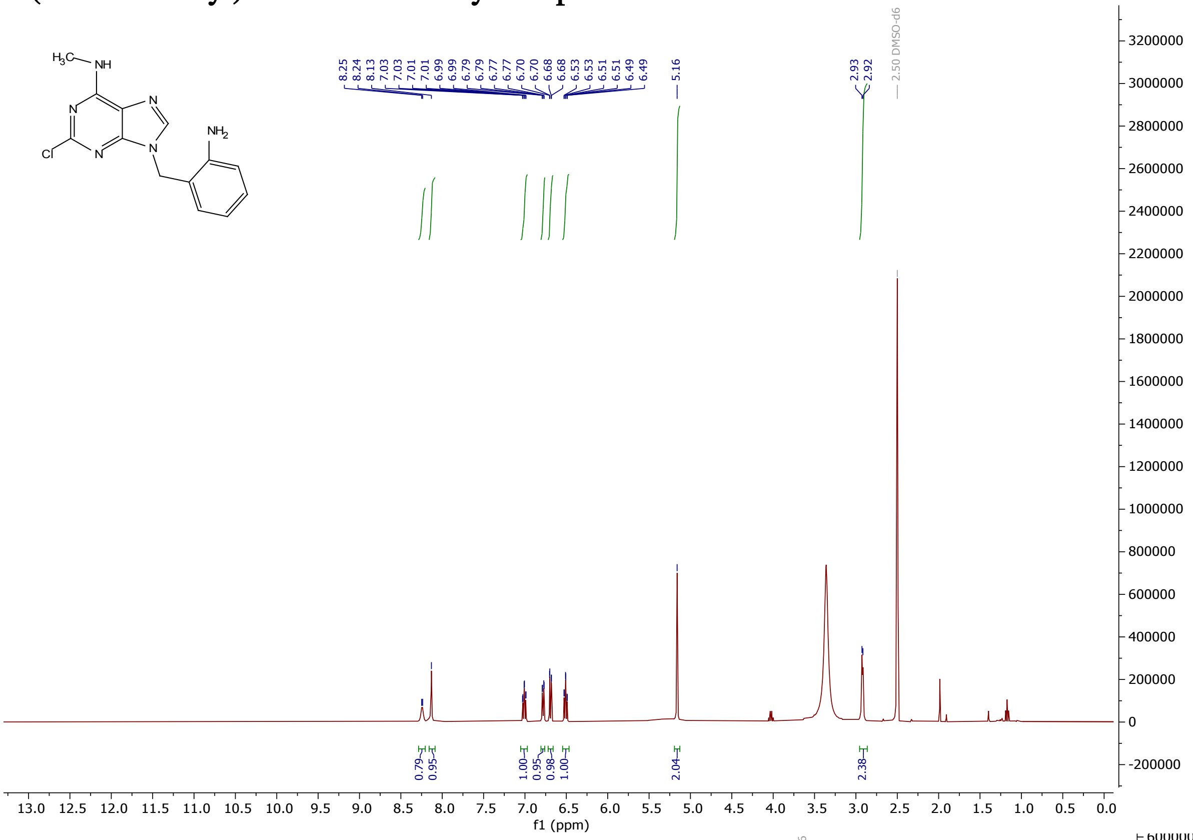


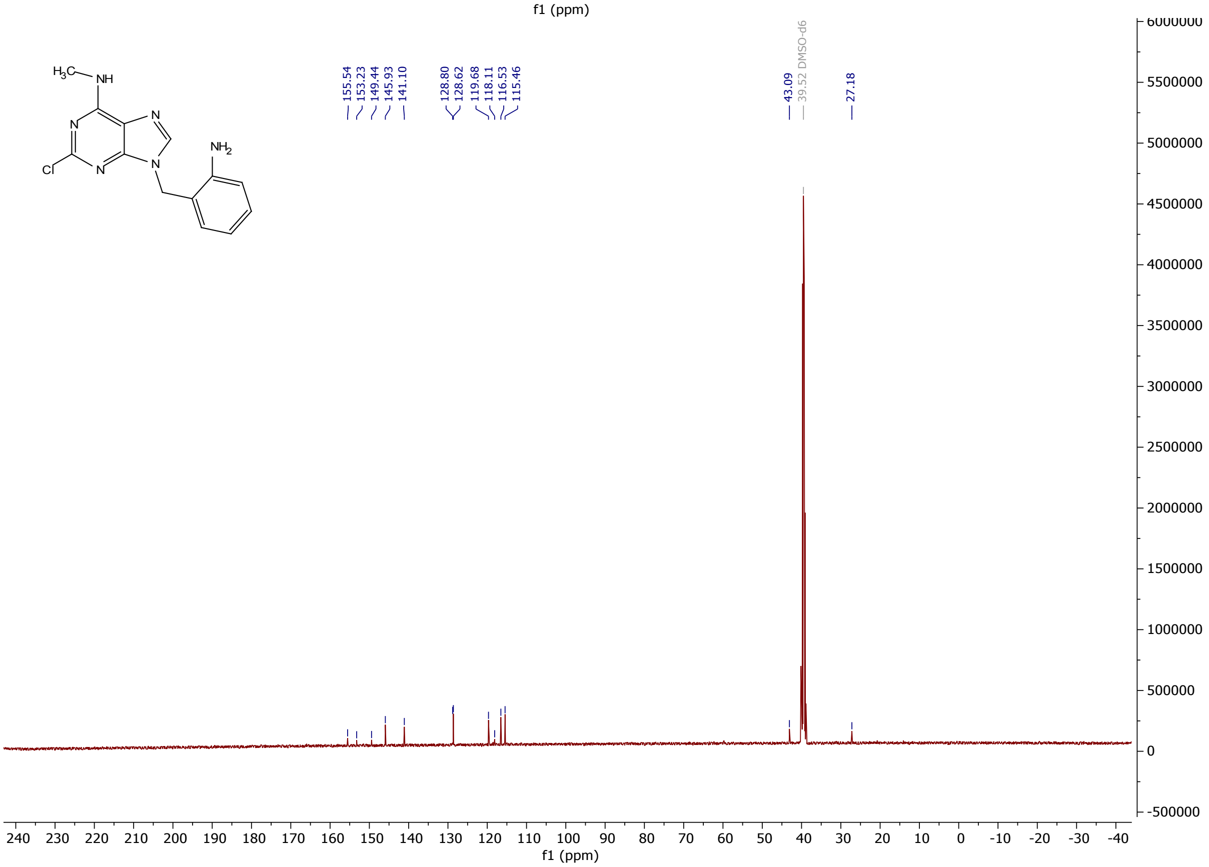

## N-(2-((2-chloro-6-(methylamino)-9H-purin-9- yl) methyl)phenyl) methanesulfonamide 13

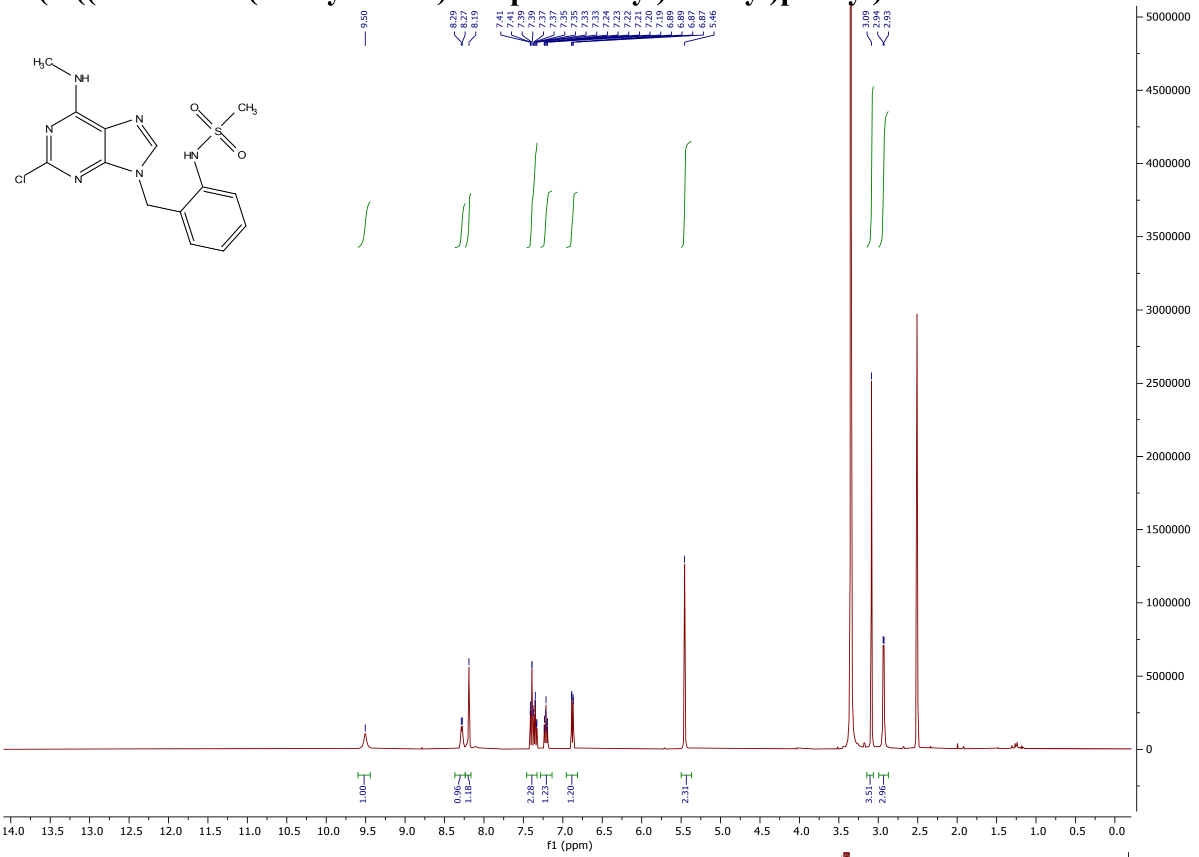

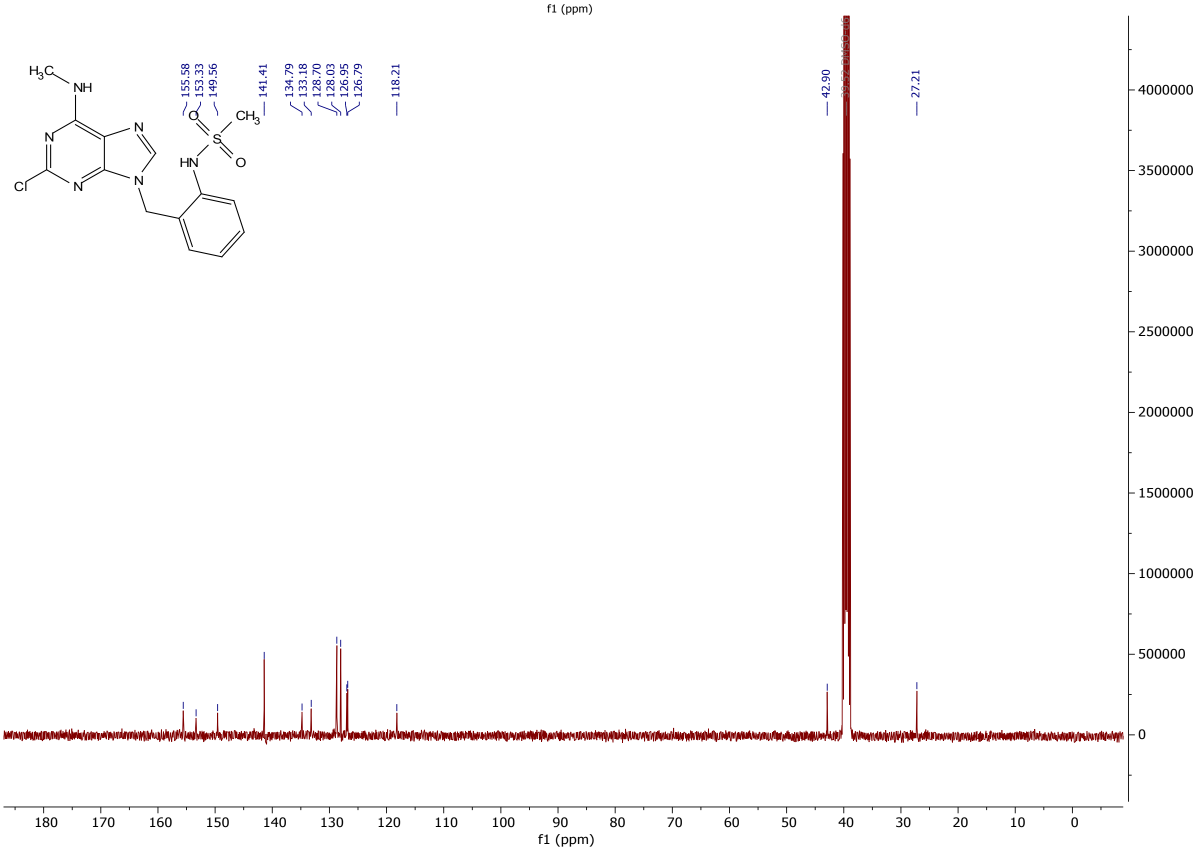

# N-(2-((2-chloro-6-(methylamino)-9H-purin-9-yl)methyl)phenyl)-4-methylbenzenesulfonamide

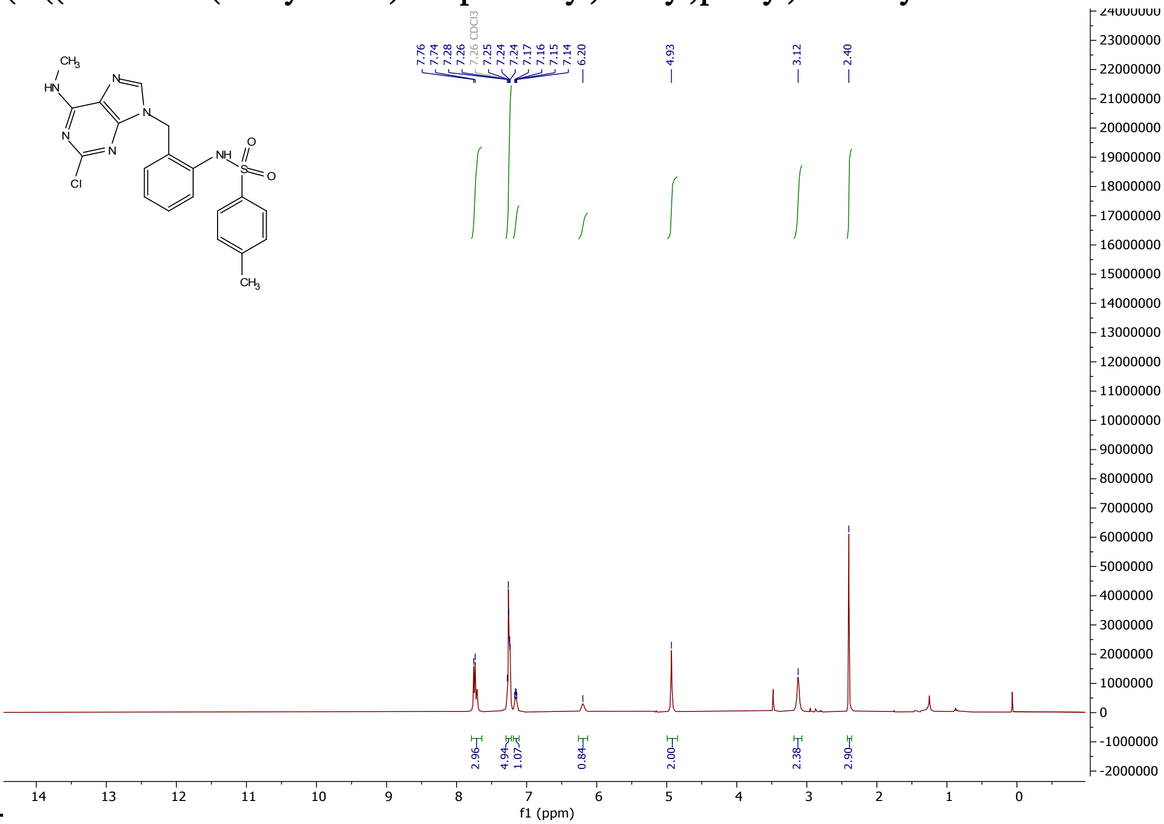


14

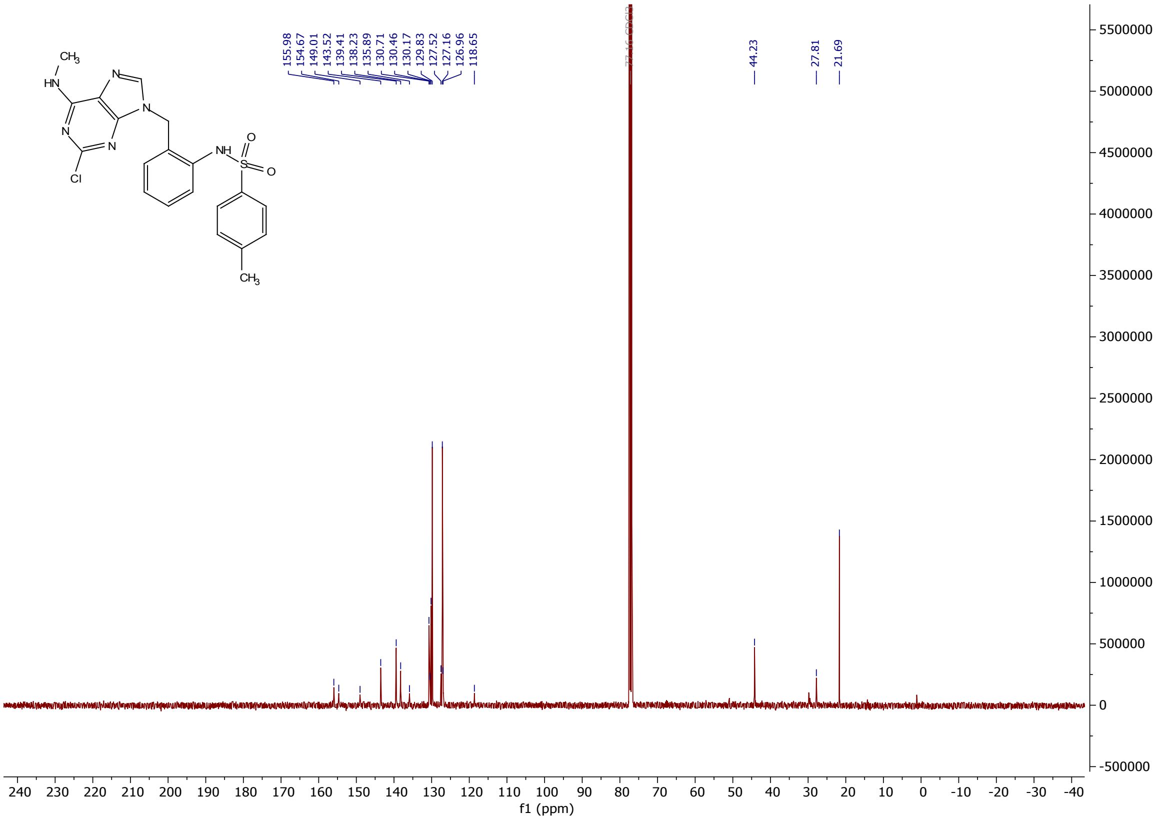

# N-(2-((2-chloro-6-(methylamino)-9H-purin-9-yl)methyl)phenyl)-2,2,2-trifluoroacetamide 15

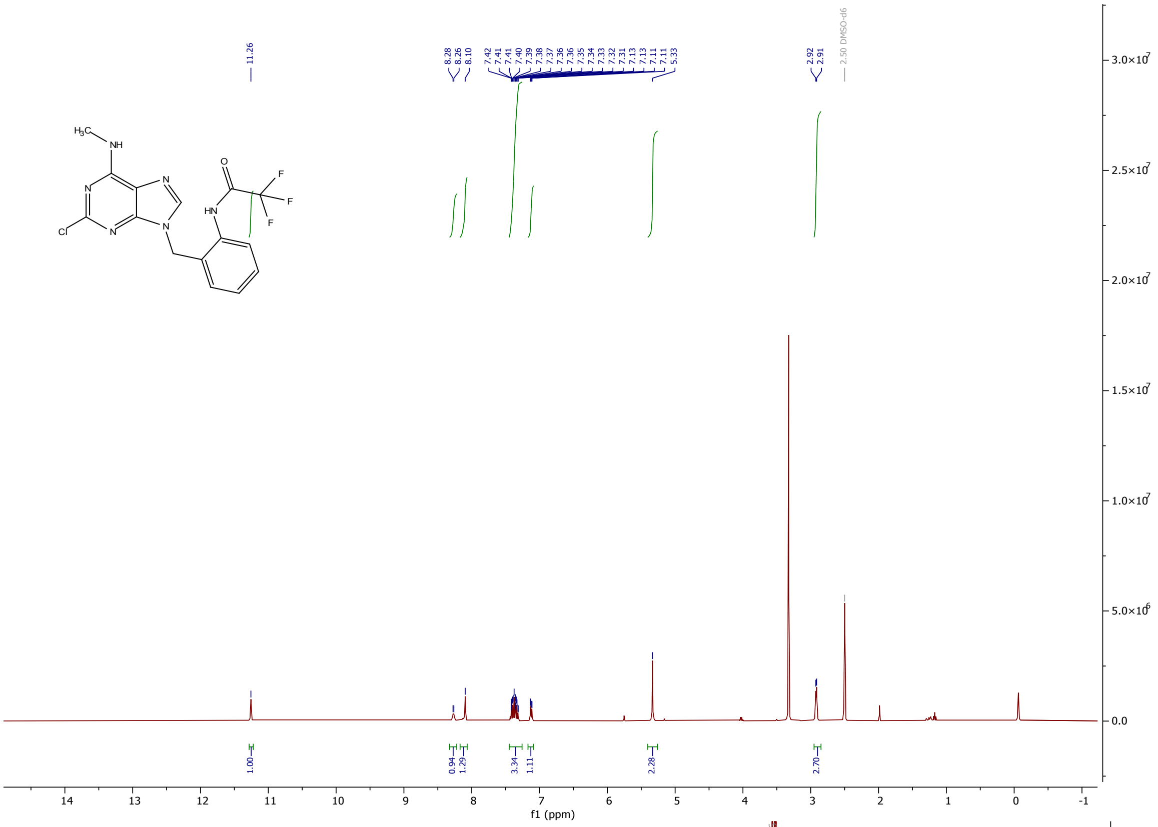


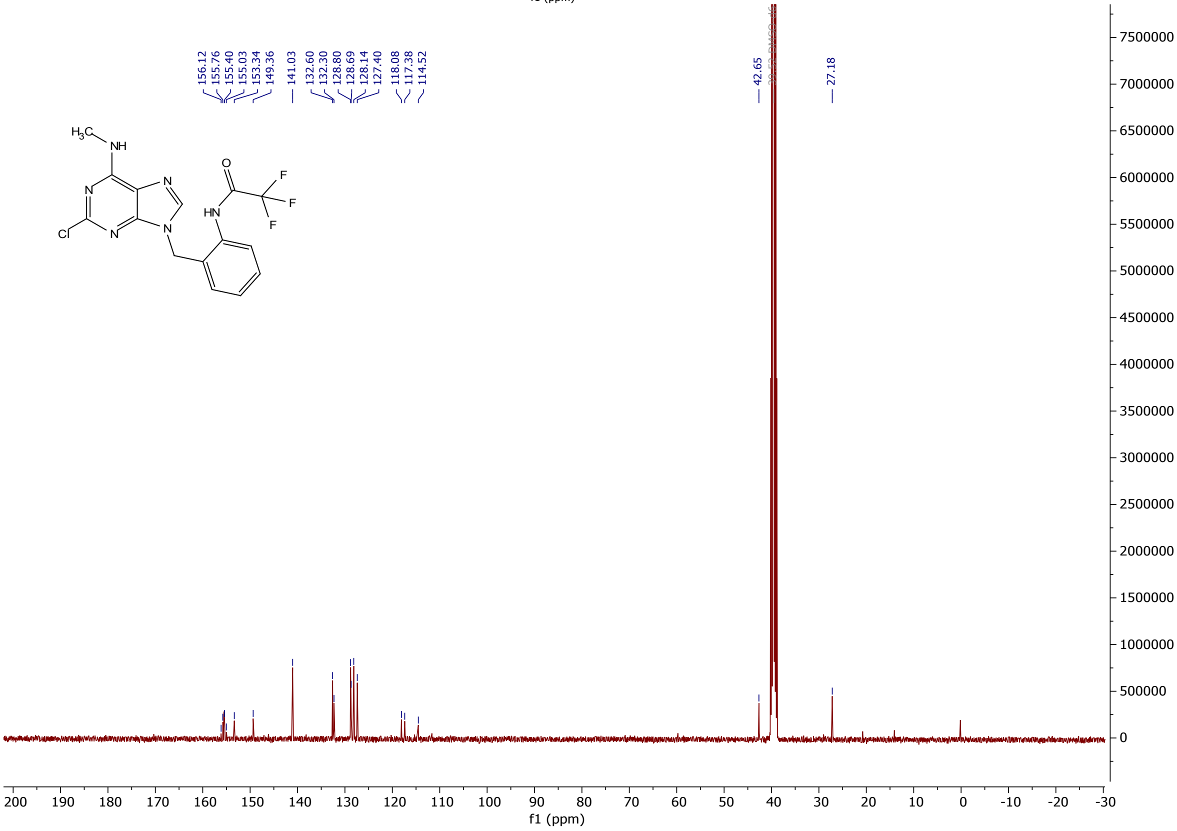

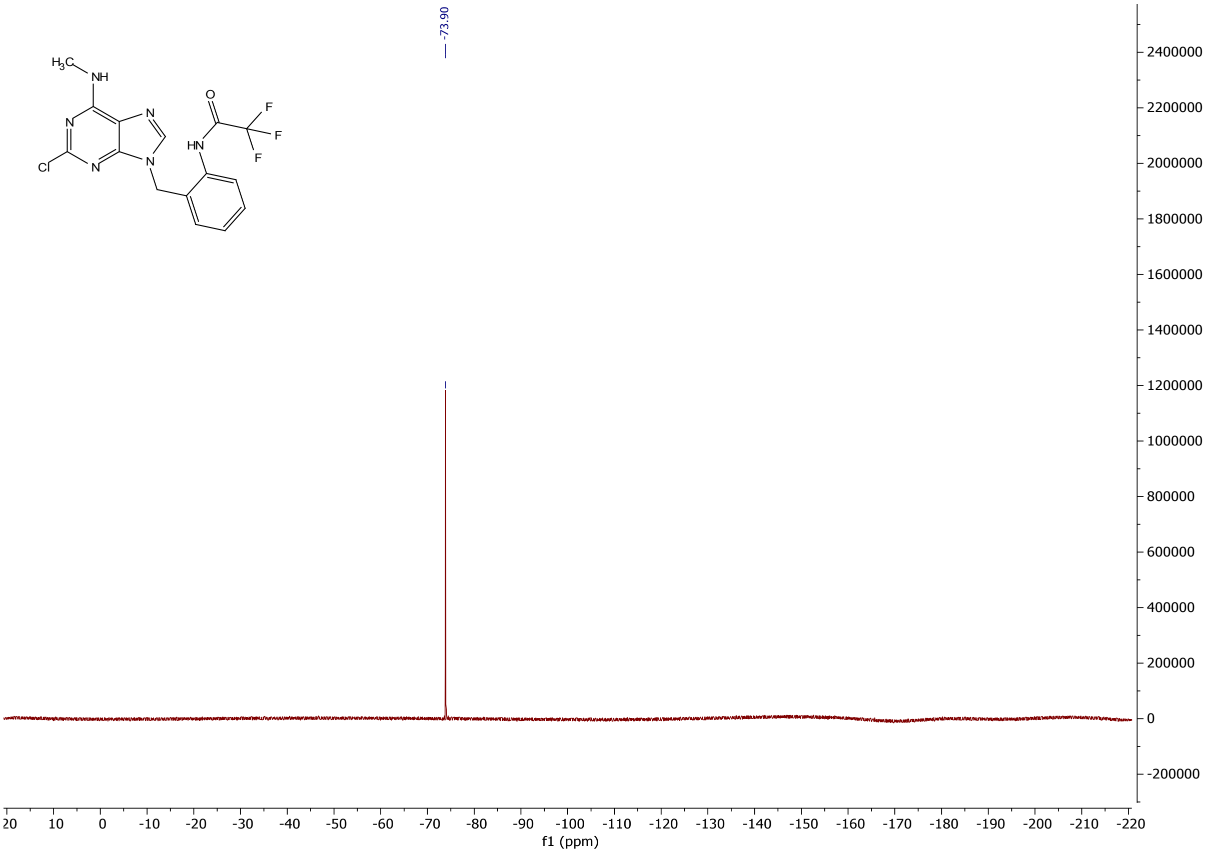
-73.90
f1 (ppm)

2-chloro-9-(2-(difluoromethyl)benzyl)-N-methyl-9H-purin-6-amine 16

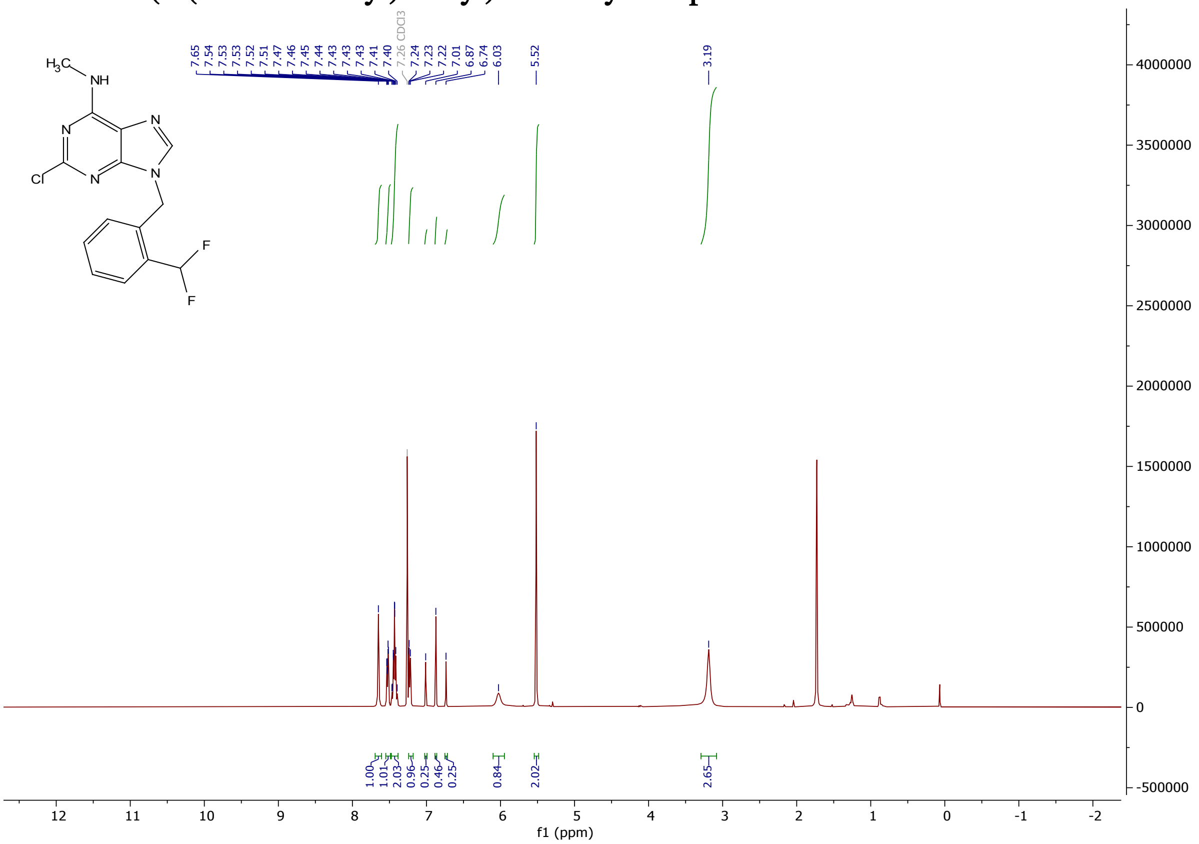


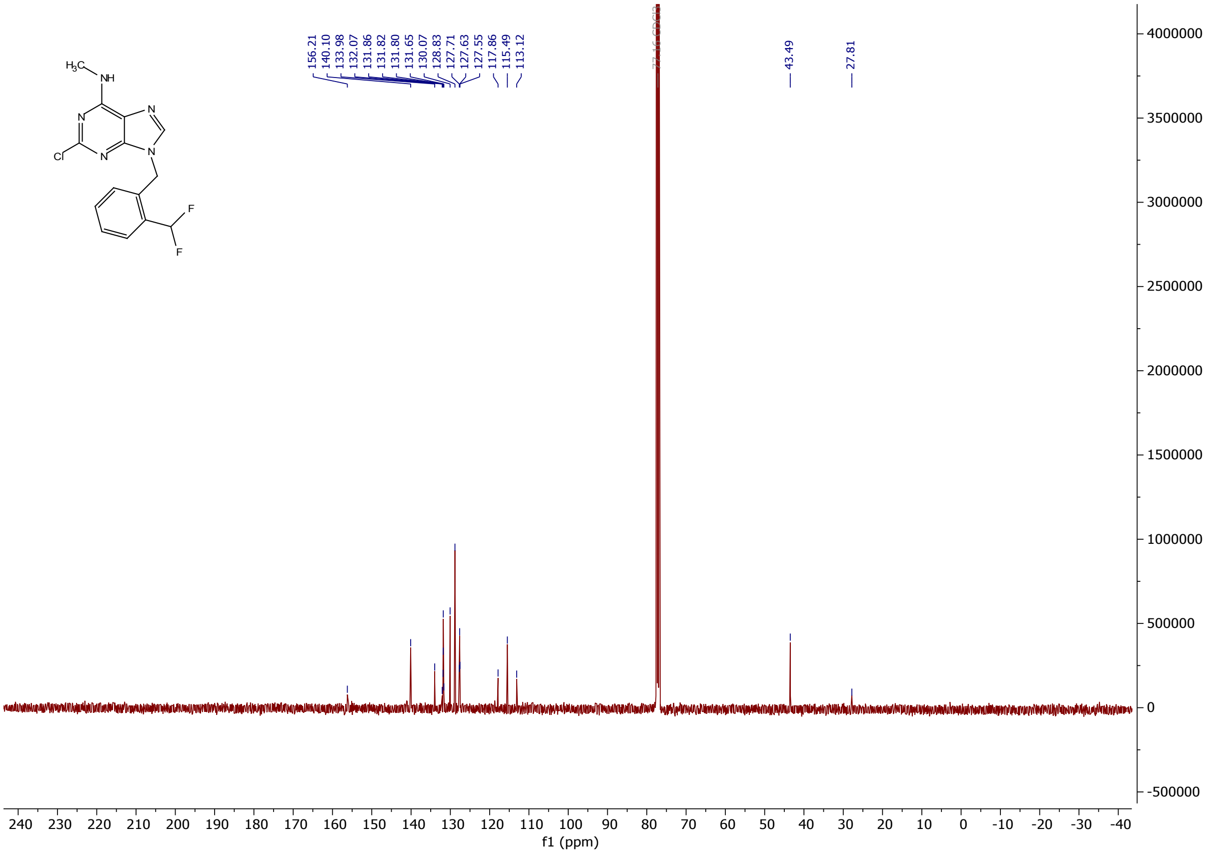

# 2-chloro-N-methyl-9-(2-(trifluoromethyl)benzyl)-9H-purin-6-amine 17

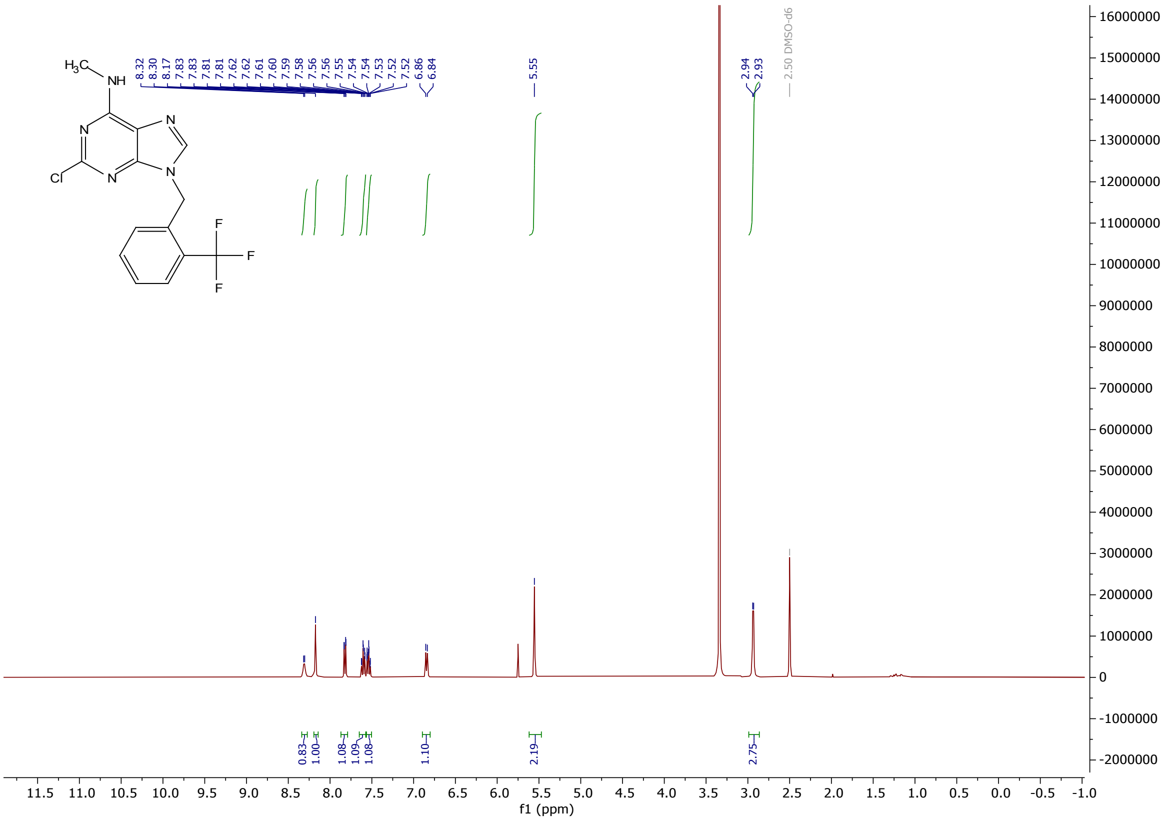


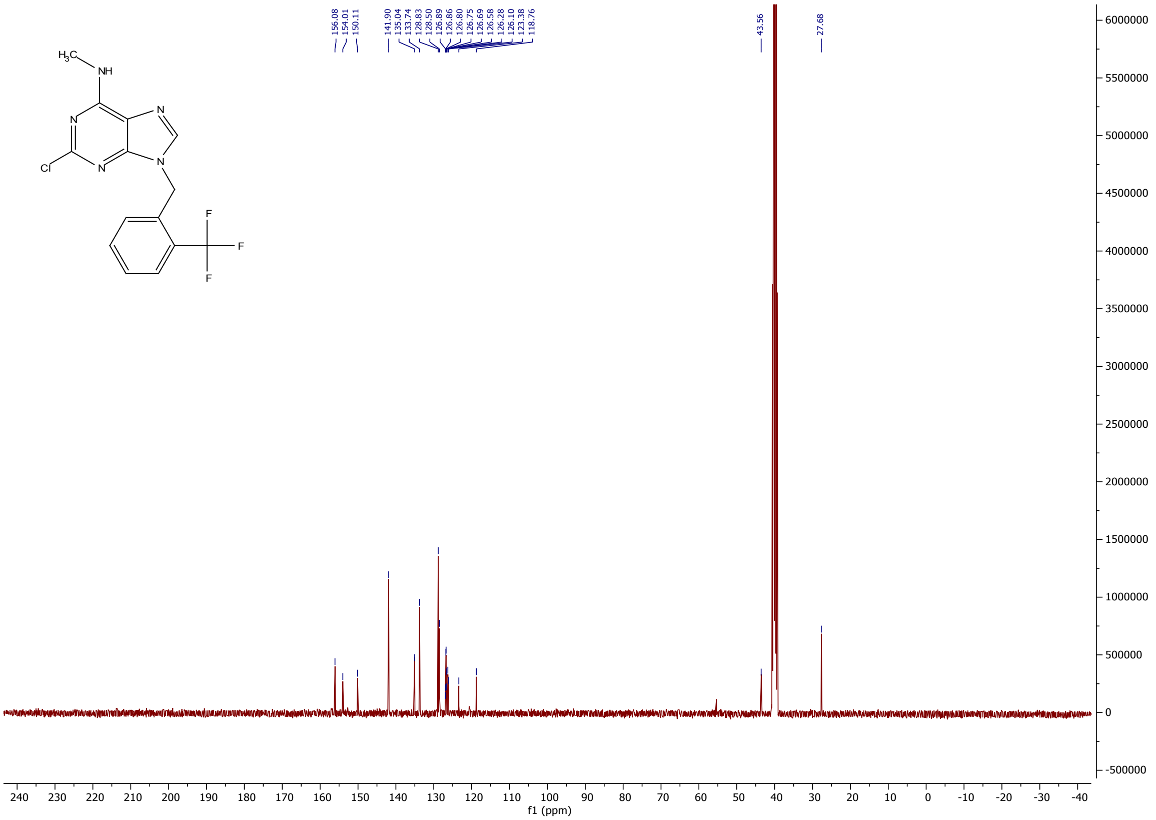

# 2-chloro-9-(2-methoxybenzyl)-N-methyl-9H-purin-6-amine 18

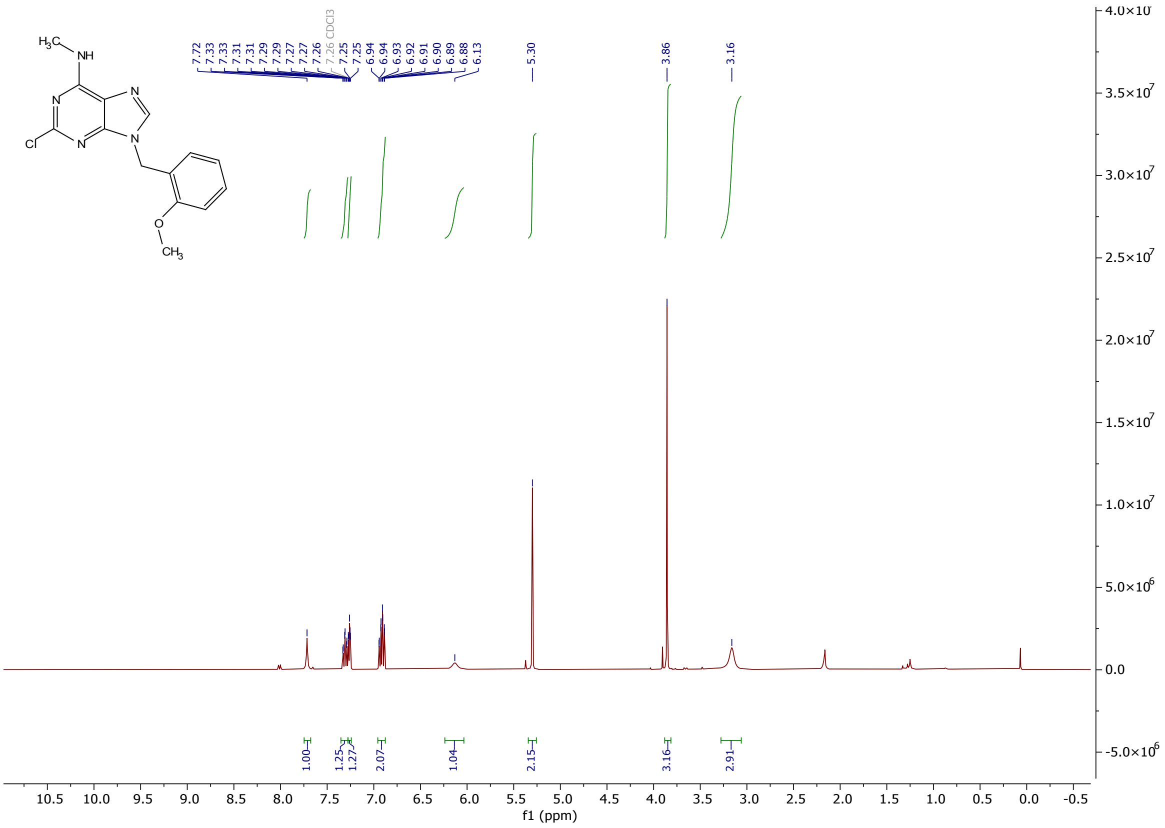


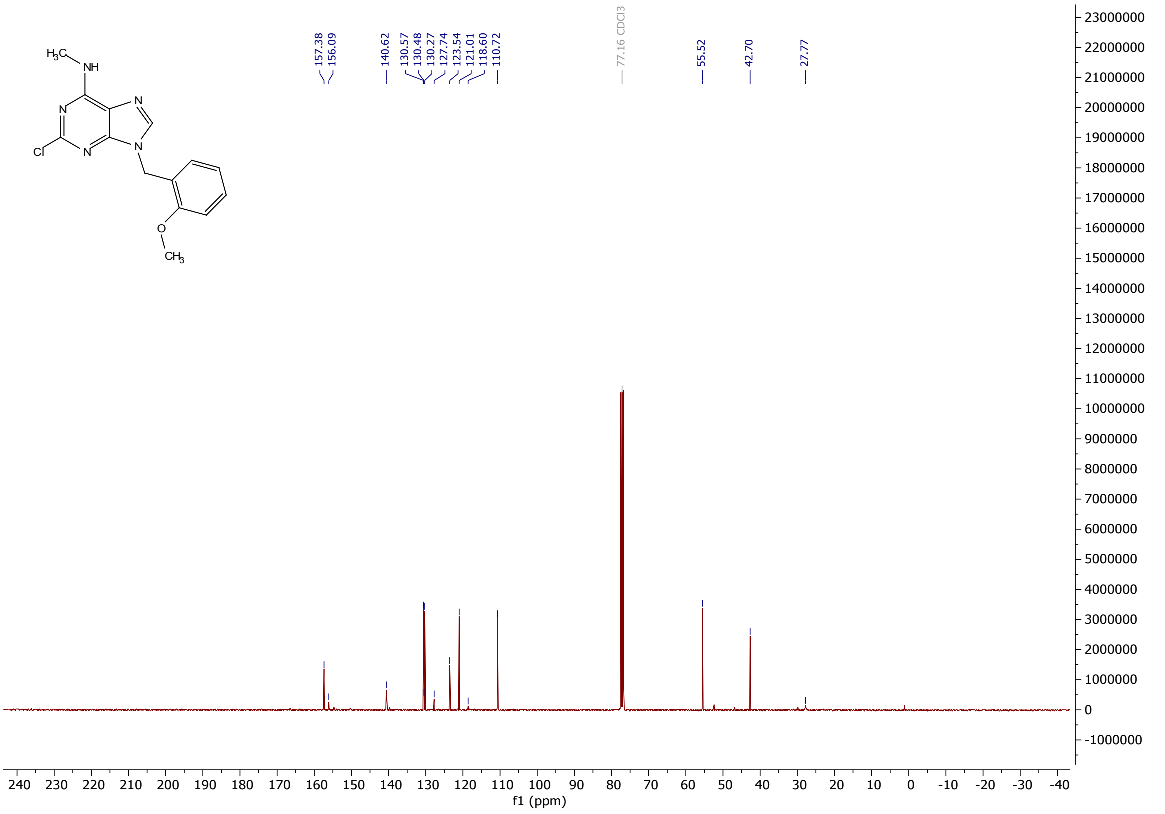

# methyl 3-((2-chloro-6-(methylamino)-9H-purin-9-yl)methyl)benzoate 19

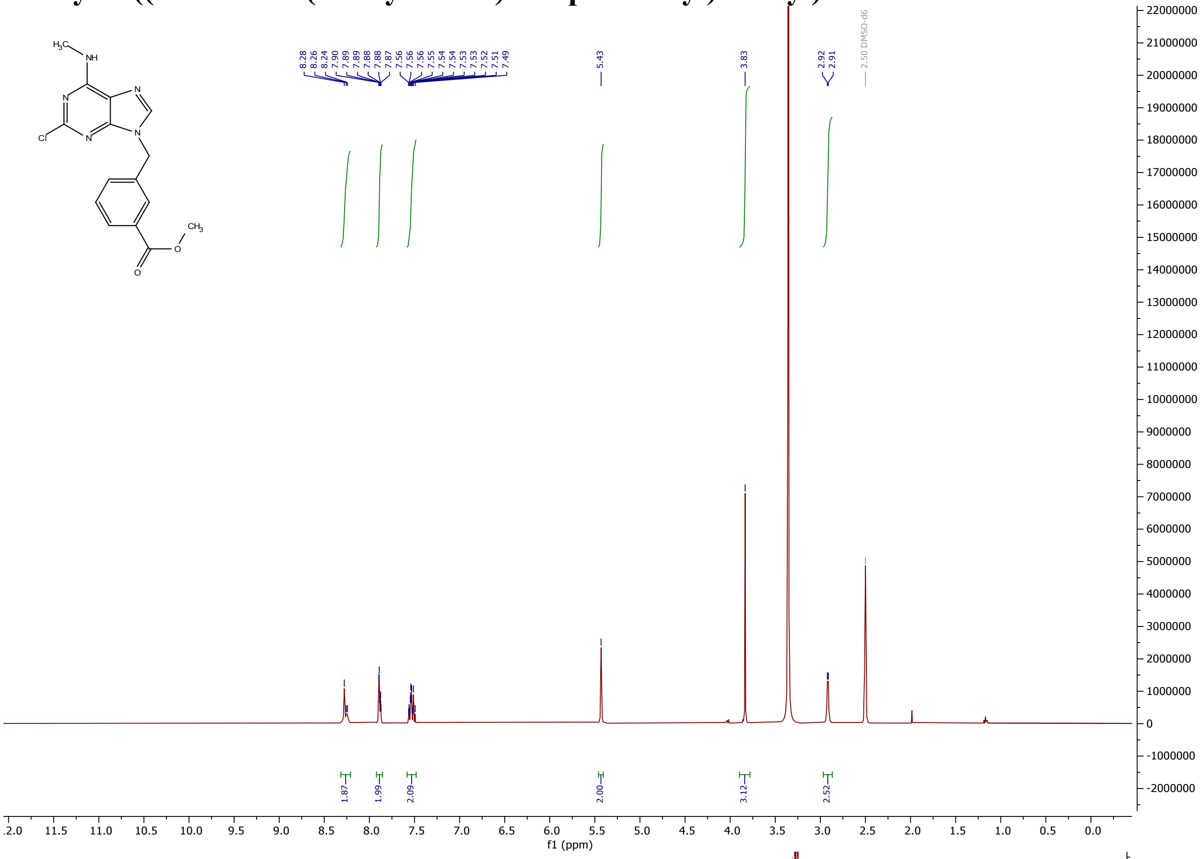


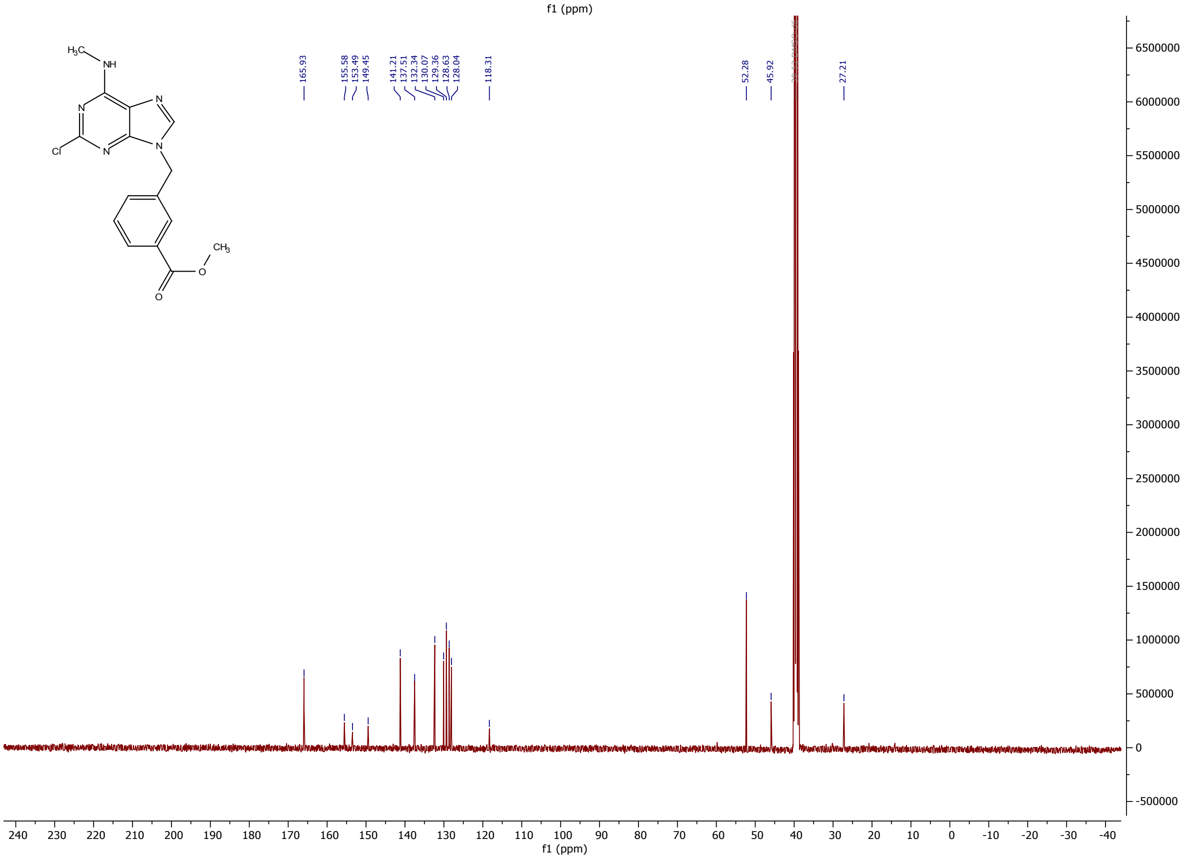

3-((2-chloro-6-(methylamino)-9H-purin-9-yl)methyl)benzoic acid 20

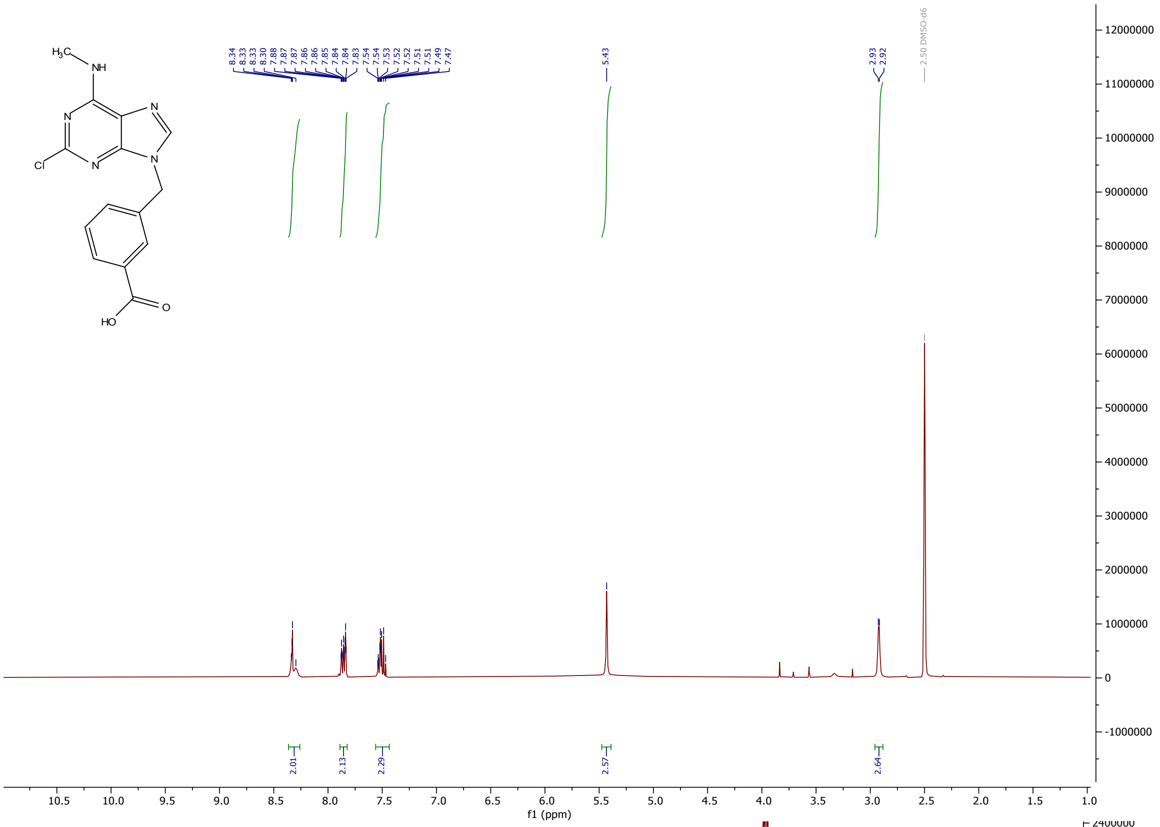
8.34
8.33
8.33
8.30
7.88
7.87
7.87
7.86
7.85
7.84
7.84
7.83
7.54
7.54
7.53
7.52
7.52
7.51
7.51
7.49
7.47
5.43
2.93
2.92
2.50 DMSO-d6
2.01
2.13
2.29
2.57
2.64
f1 (ppm)
H3C
NH
N
N
N
N
Cl
HO
O

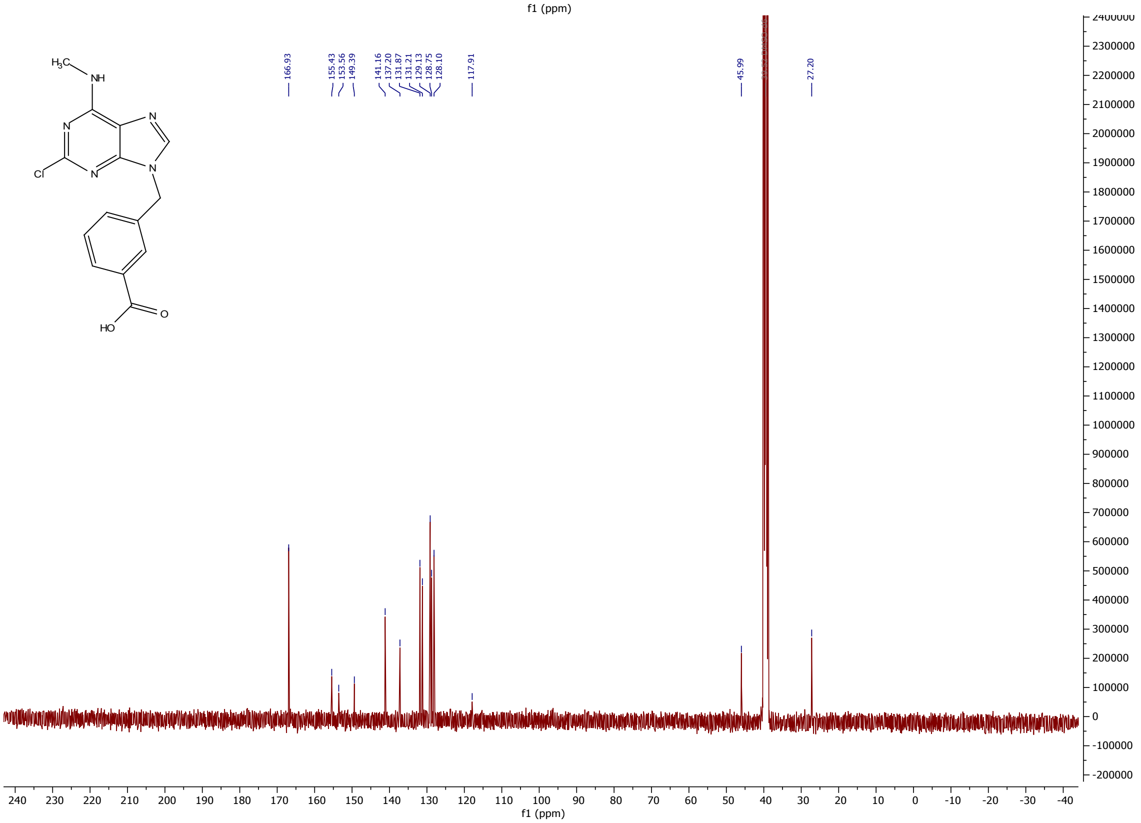
166.93
155.43
153.56
149.39
141.16
137.20
131.87
131.21
129.13
128.75
128.10
117.91
45.99
27.20
f1 (ppm)
H3C
NH
N
N
N
N
Cl
HO
O

# 9-(3-(2H-tetrazol-5-yl)benzyl)-2-chloro-N-methyl-9H-purin-6-amine 21

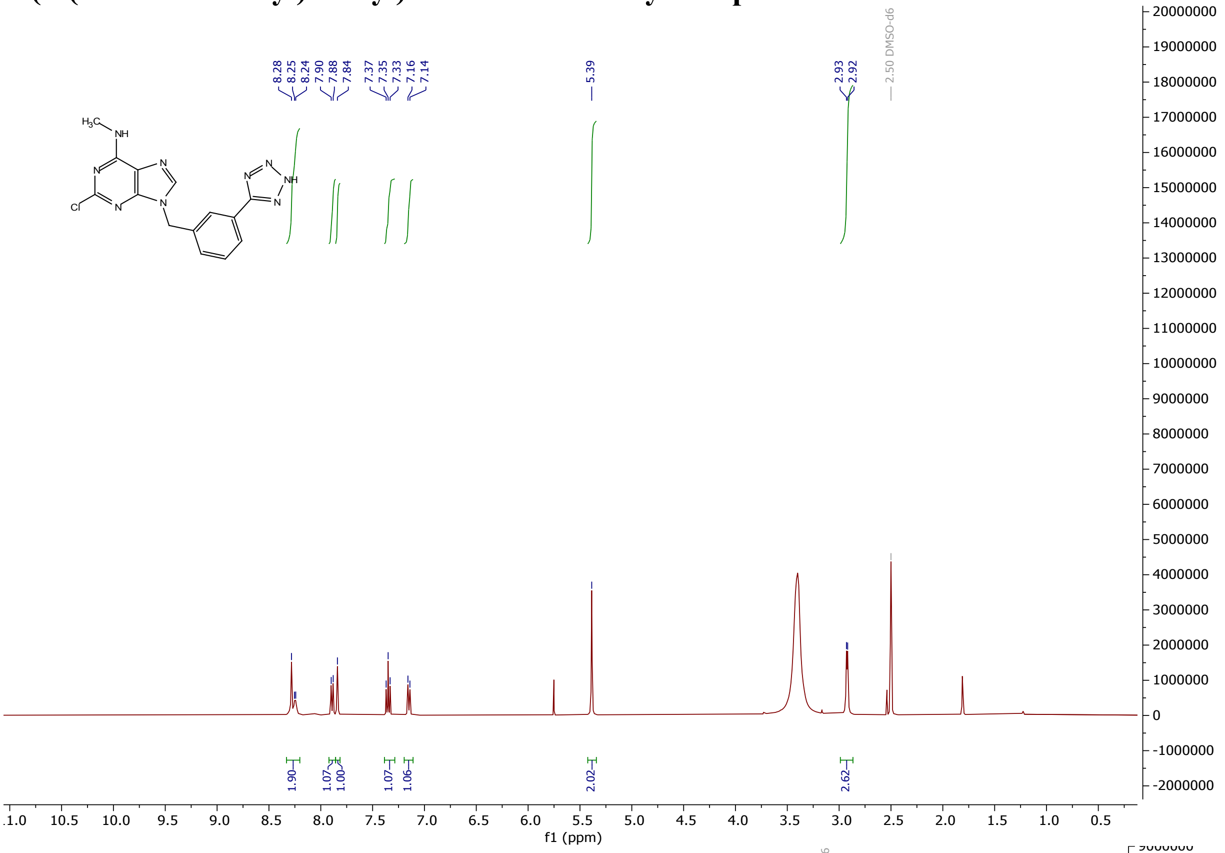


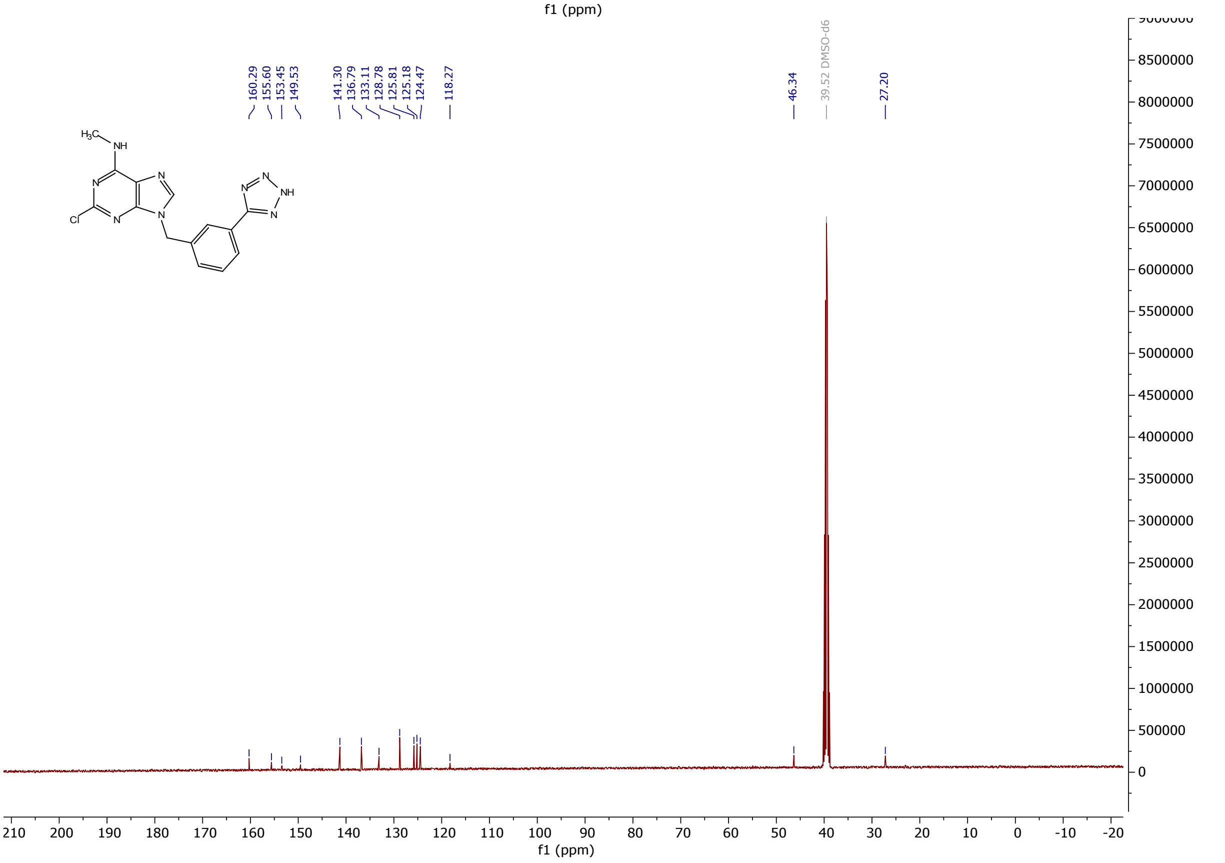

# 3-((2-chloro-6-(methylamino)-9H-purin-9-yl)methyl)benzamide 22

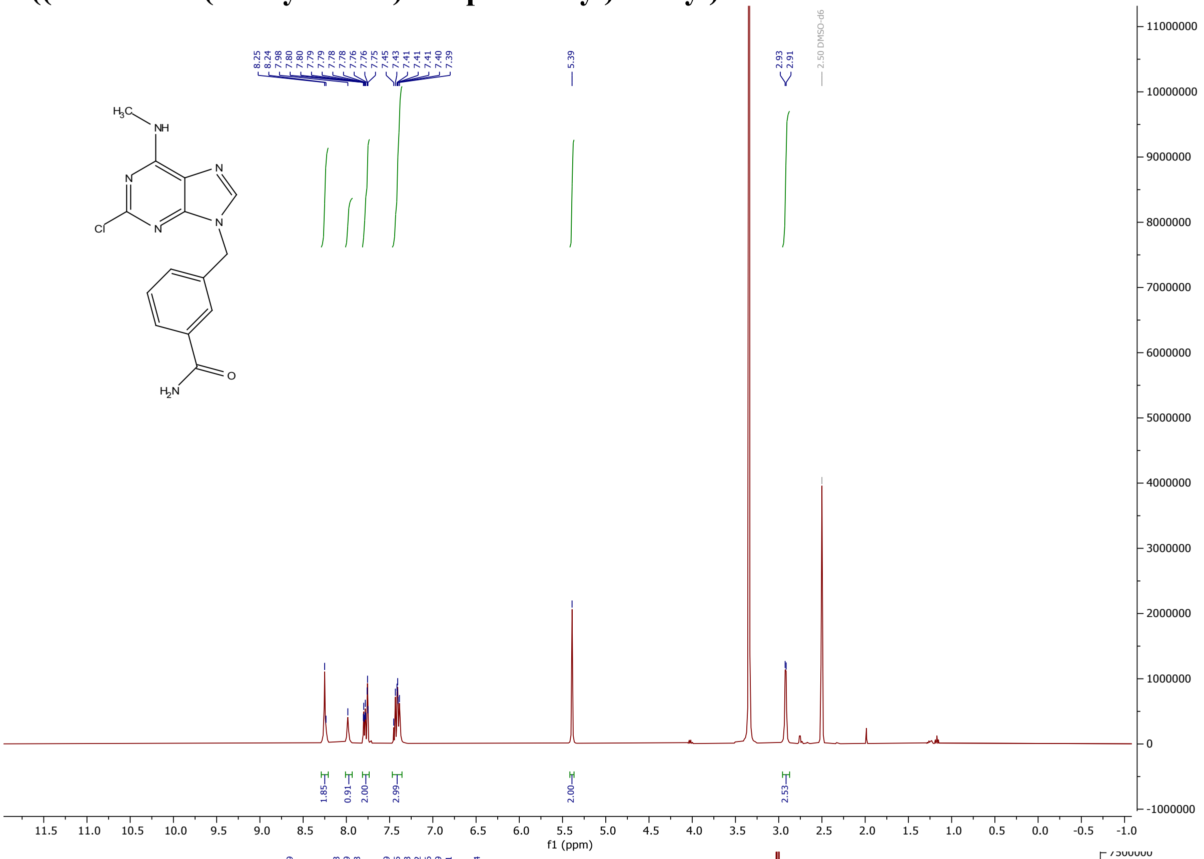


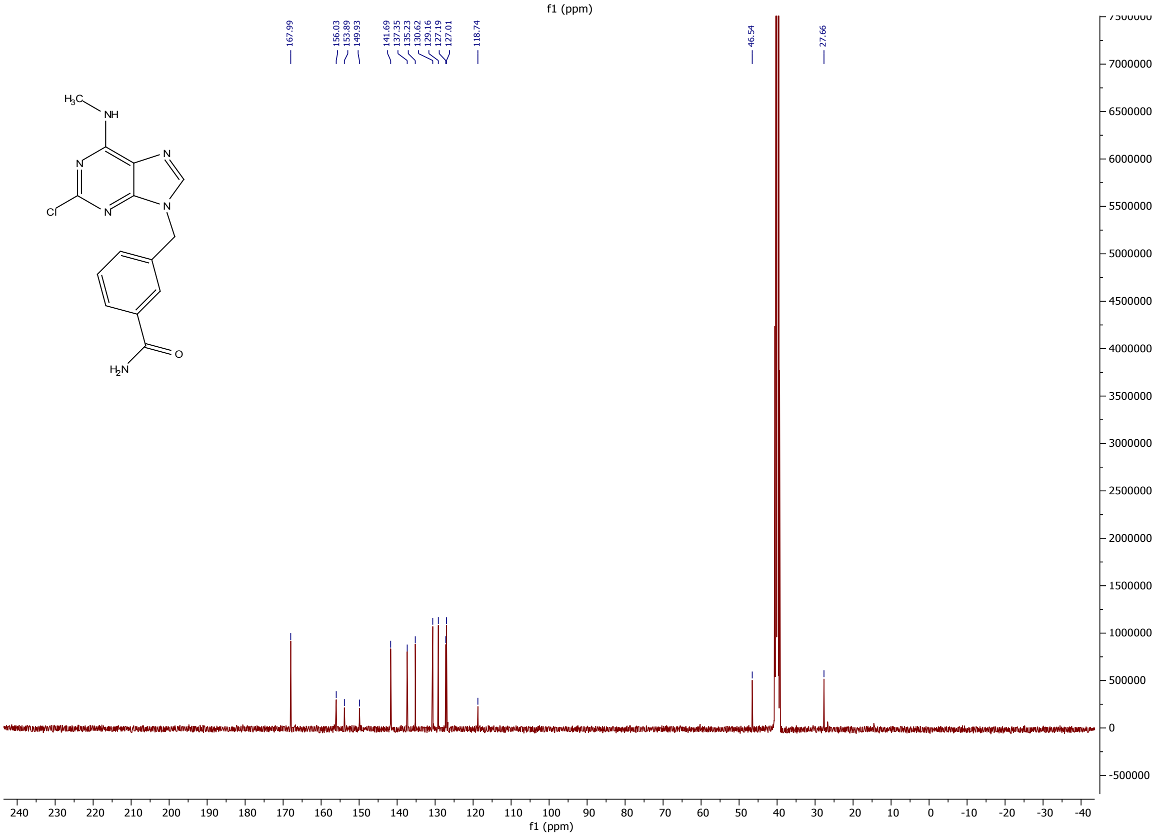

# 3-((2-chloro-6-(methylamino)-9H-purin-9-yl)methyl)-N-methylbenzamide 23

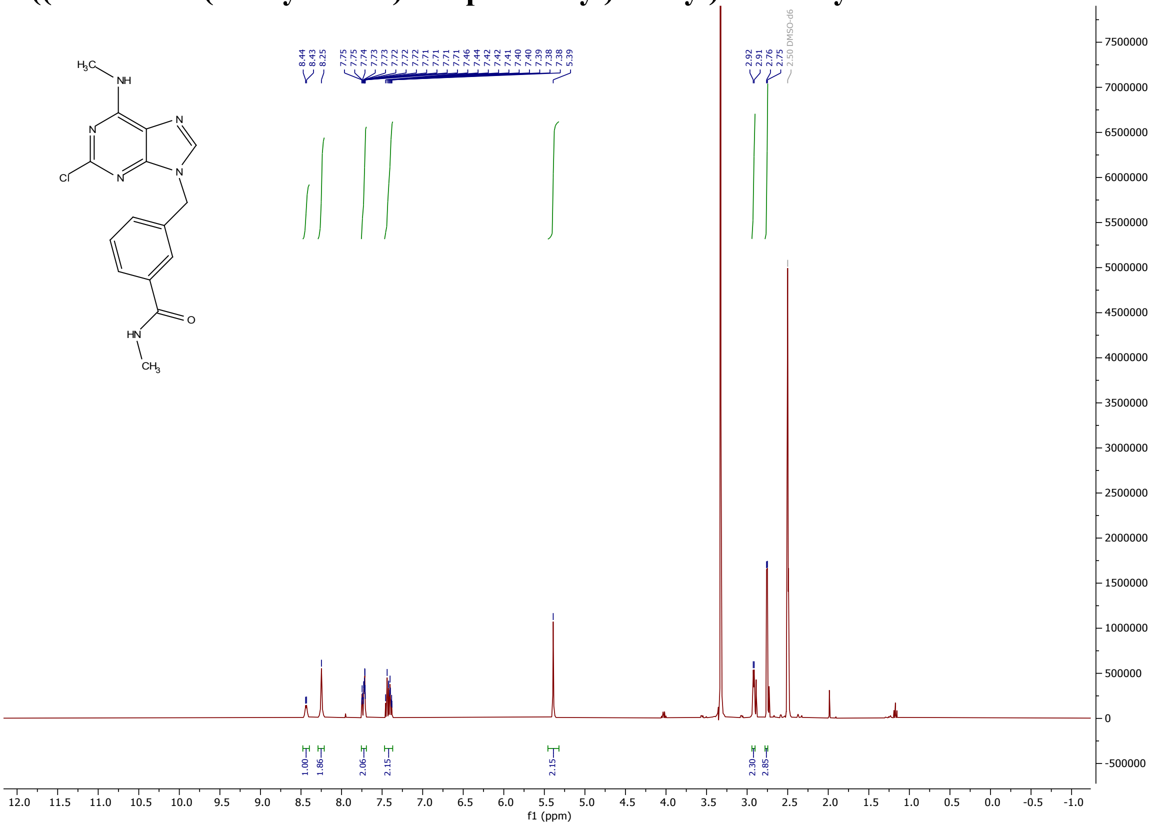


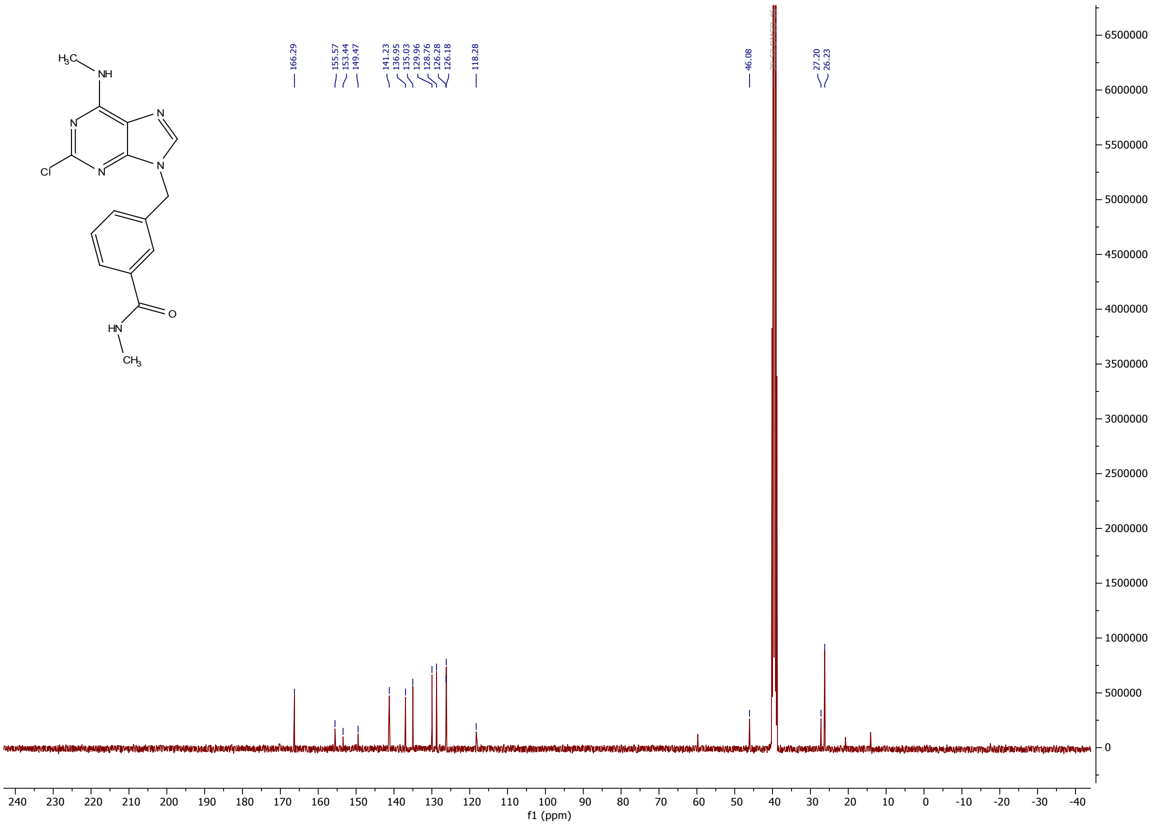

# 9-(3-bromobenzyl)-2-chloro-N-methyl-9H-purin-6-amine 24

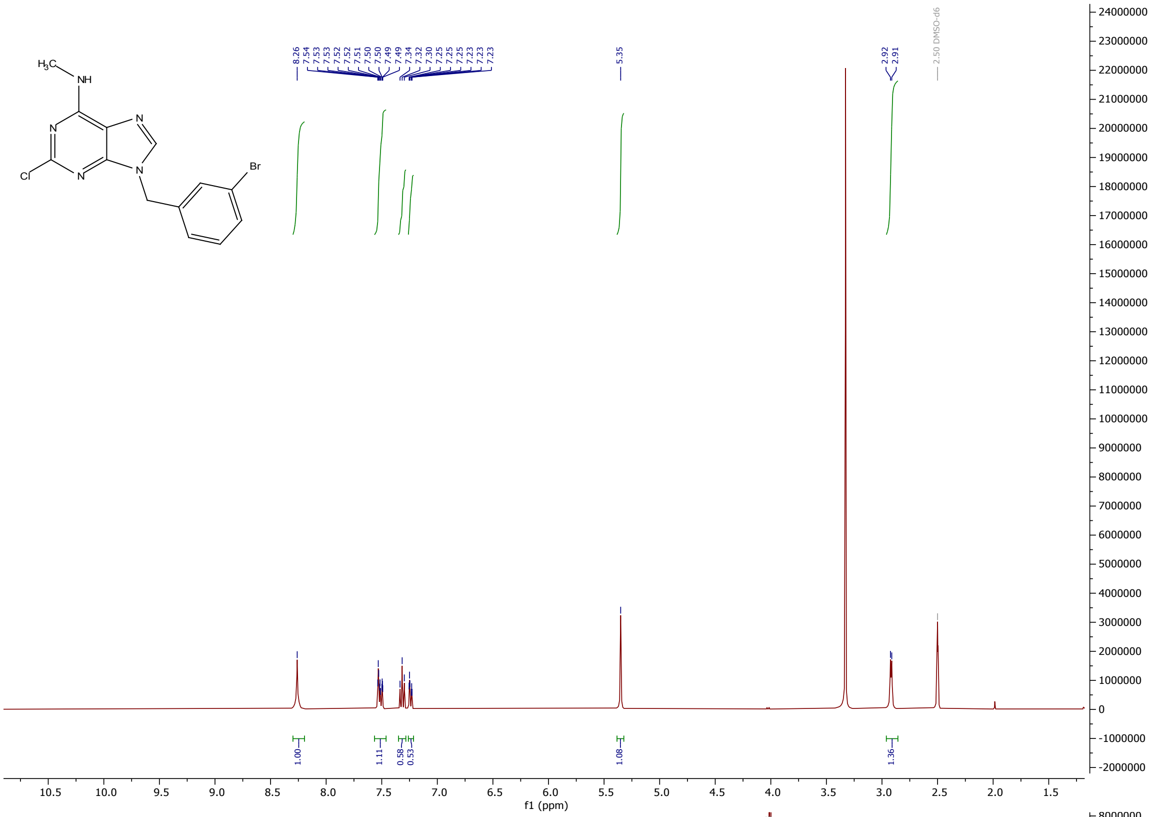


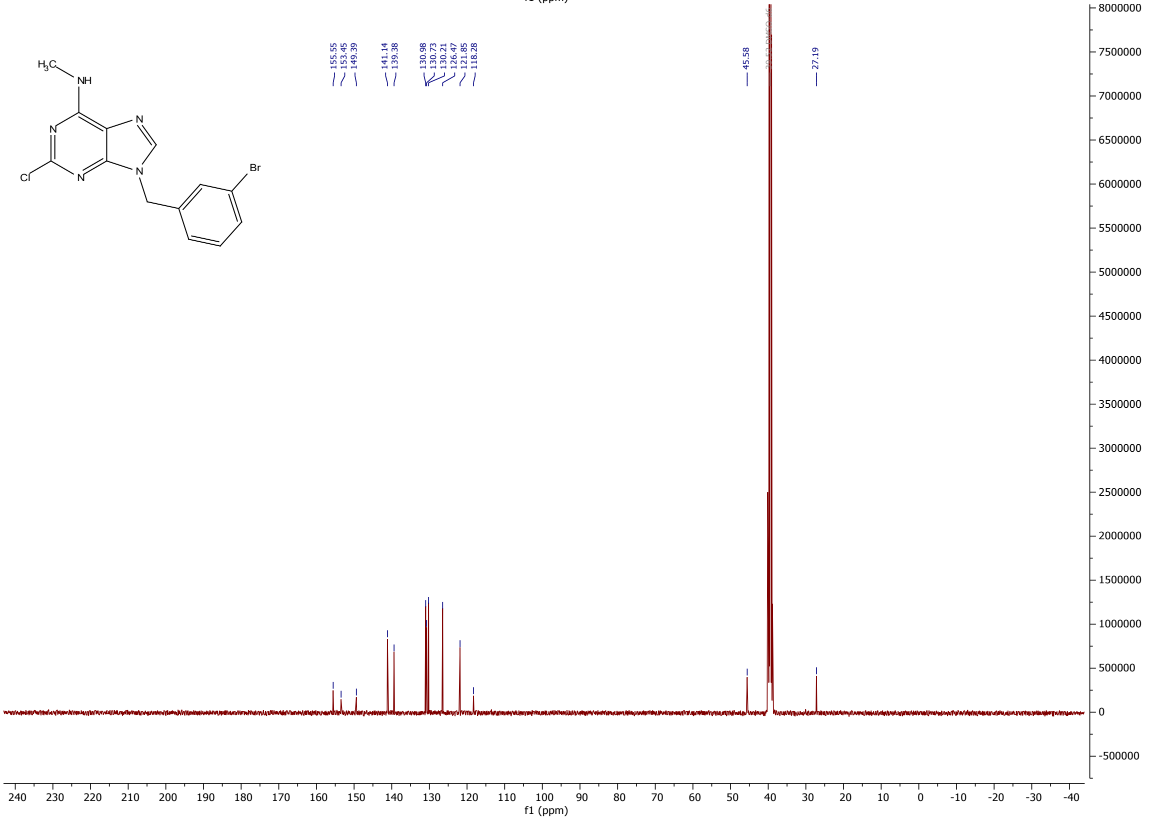

# $^{1}$H spectrum of 2-chloro-9-(3-chlorobenzyl)-N-methyl-9H-purin-6-amine 25

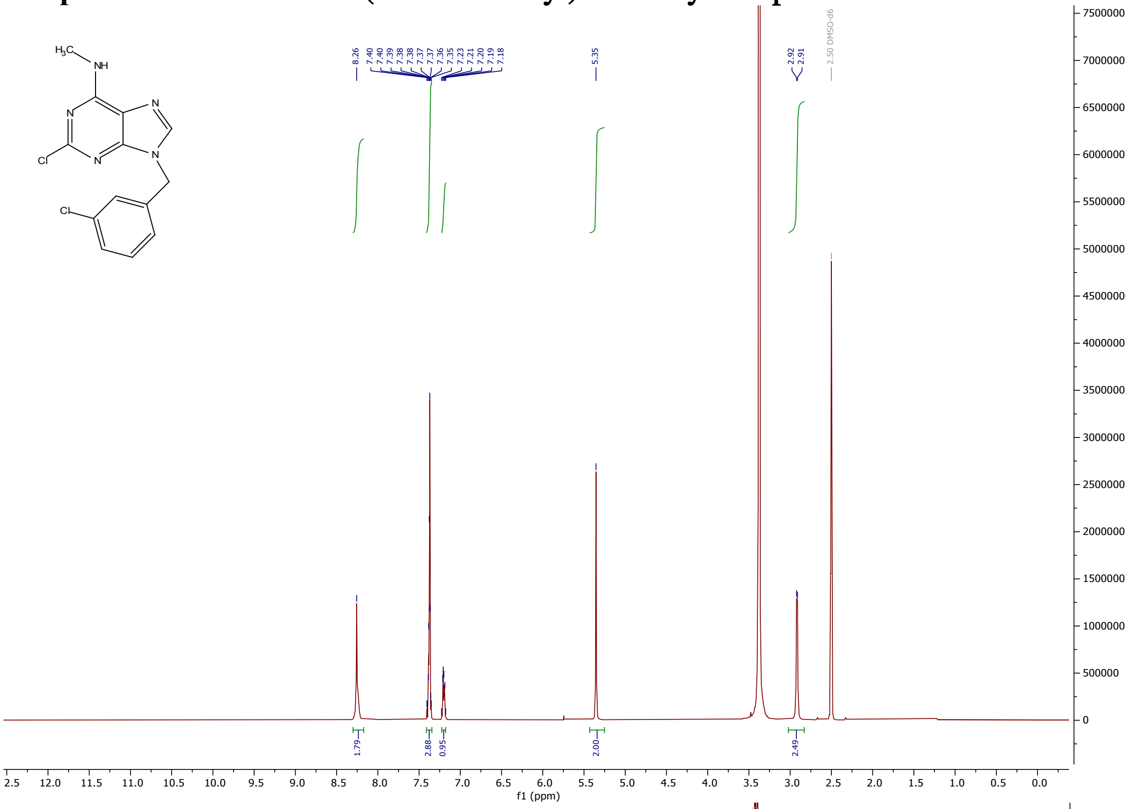


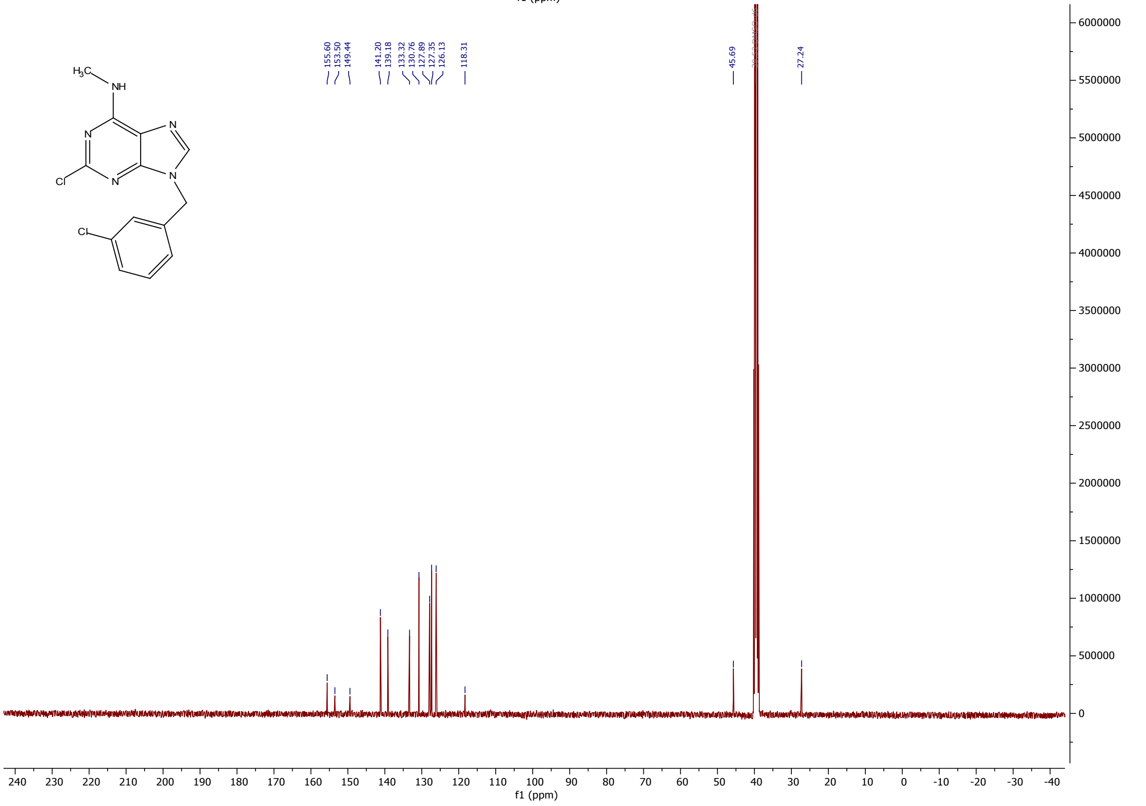

# 3-((2-chloro-6-(methylamino)-9H-purin-9-yl)methyl)benzonitrile 26

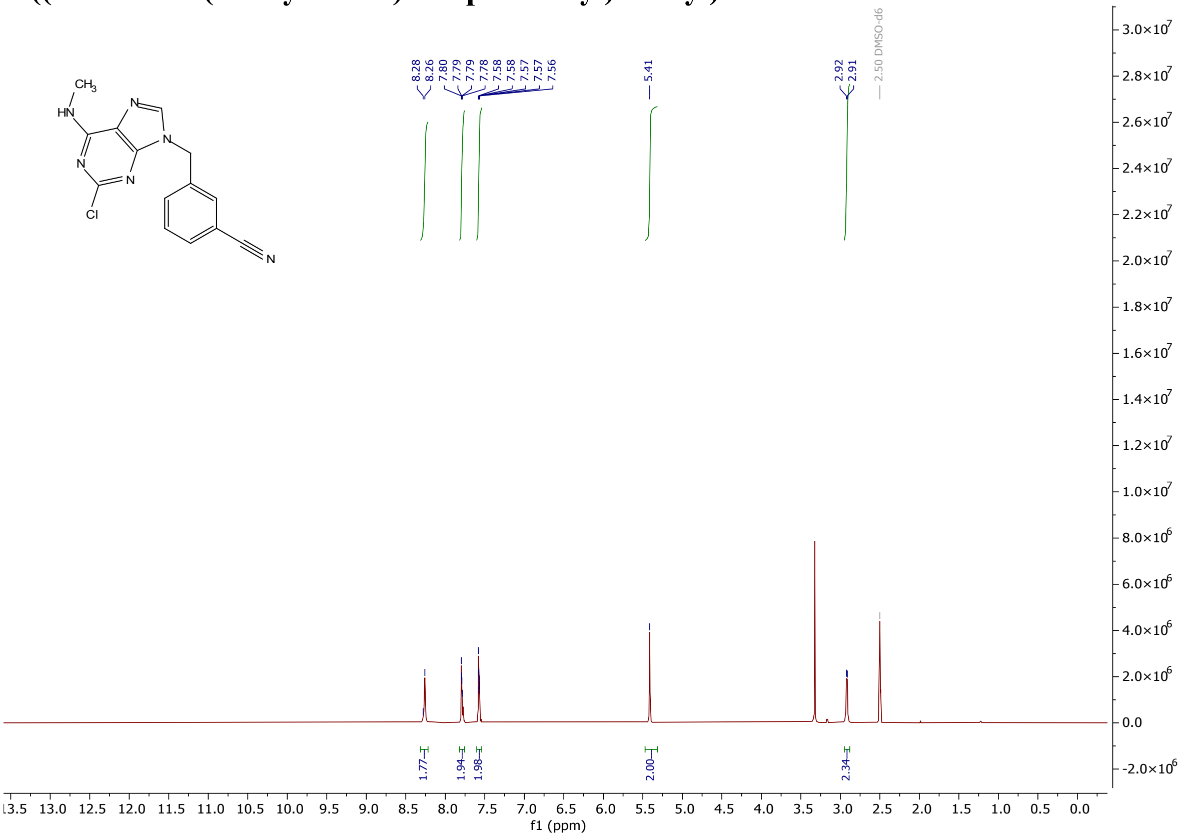


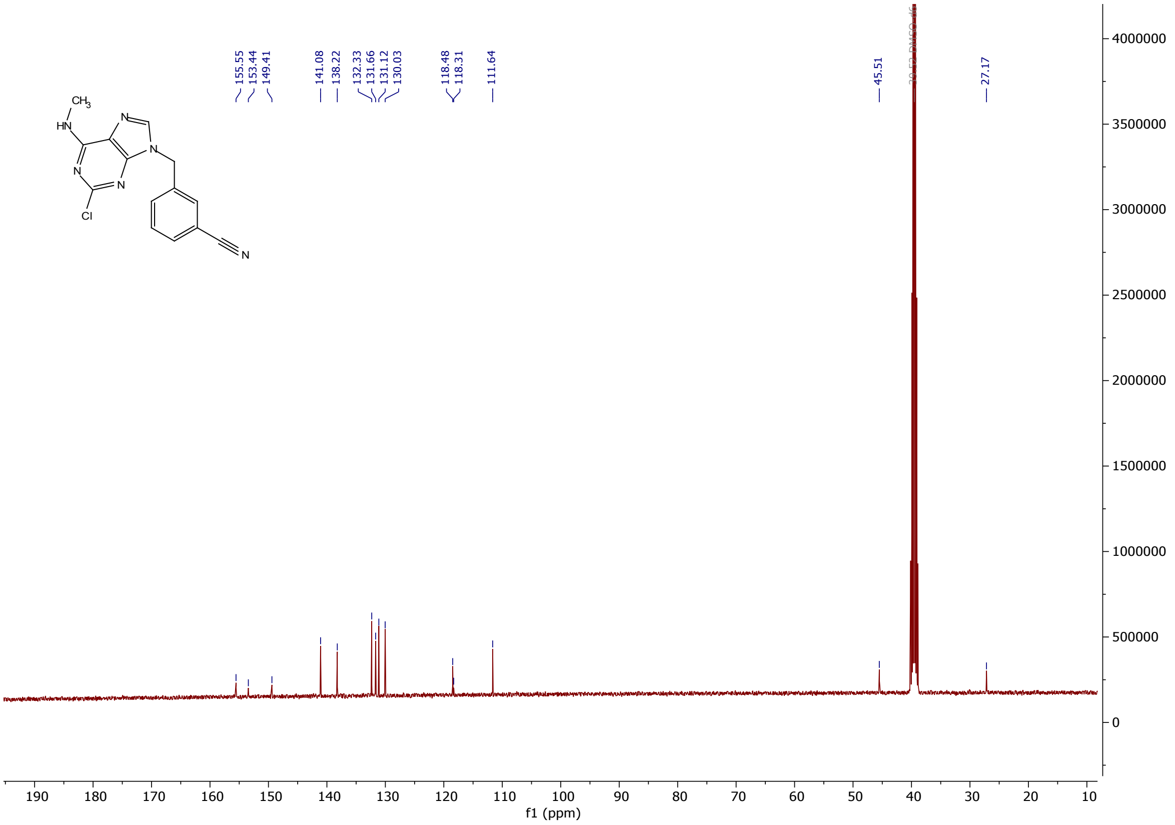

# 2-chloro-9-(3-methoxybenzyl)-N-methyl-9H-purin-6-amine 27

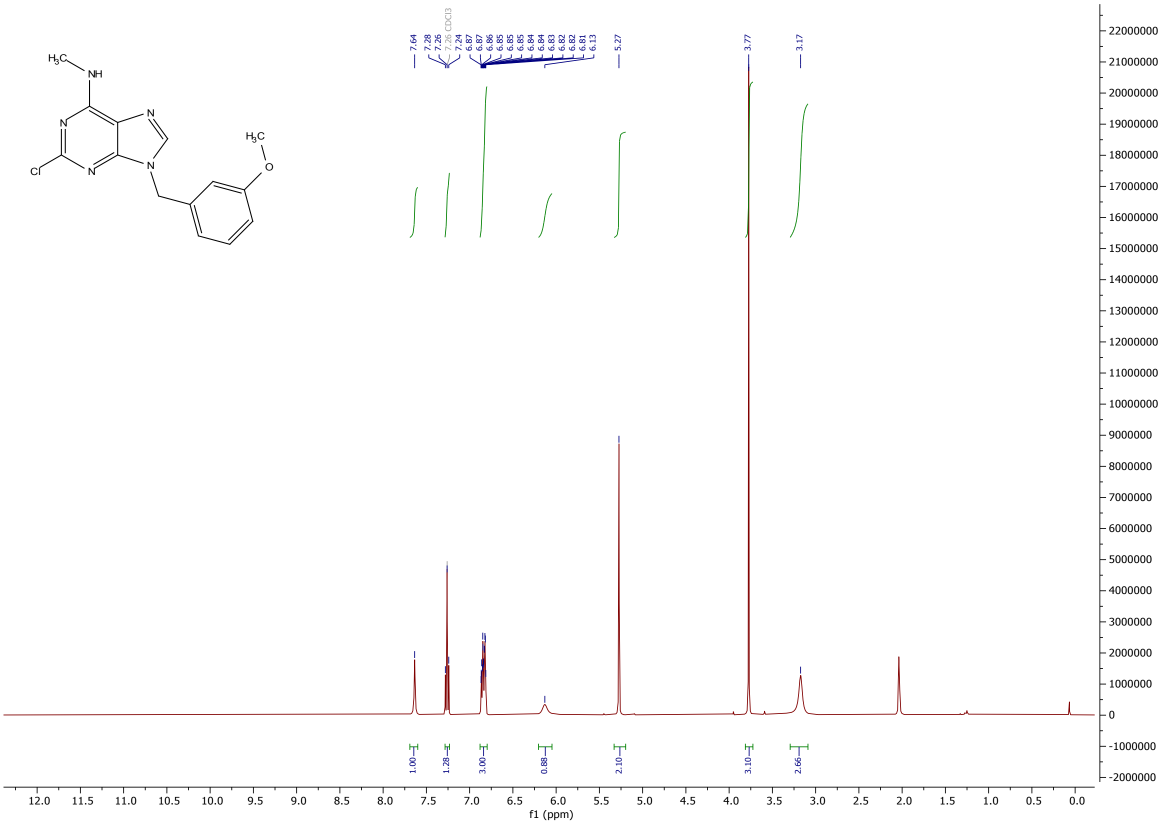

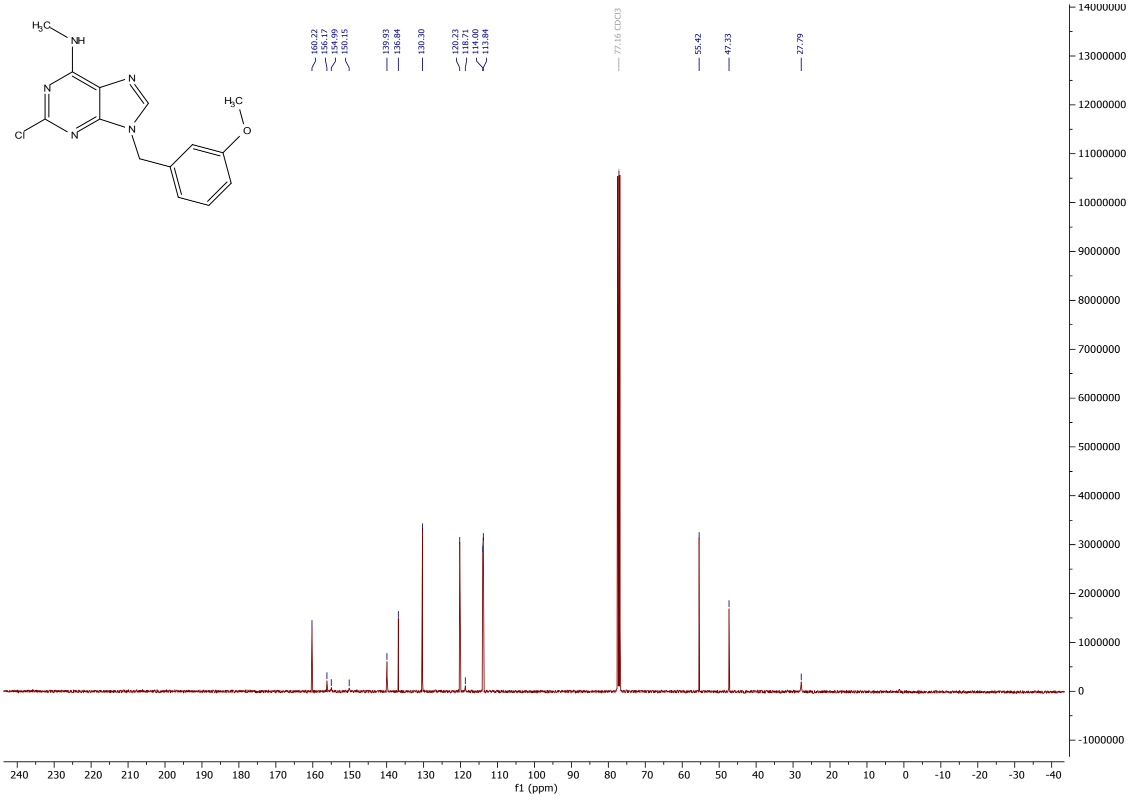

# methyl 4-((2-chloro-6-(methylamino)-9H-purin-9-yl)methyl)benzoate 28

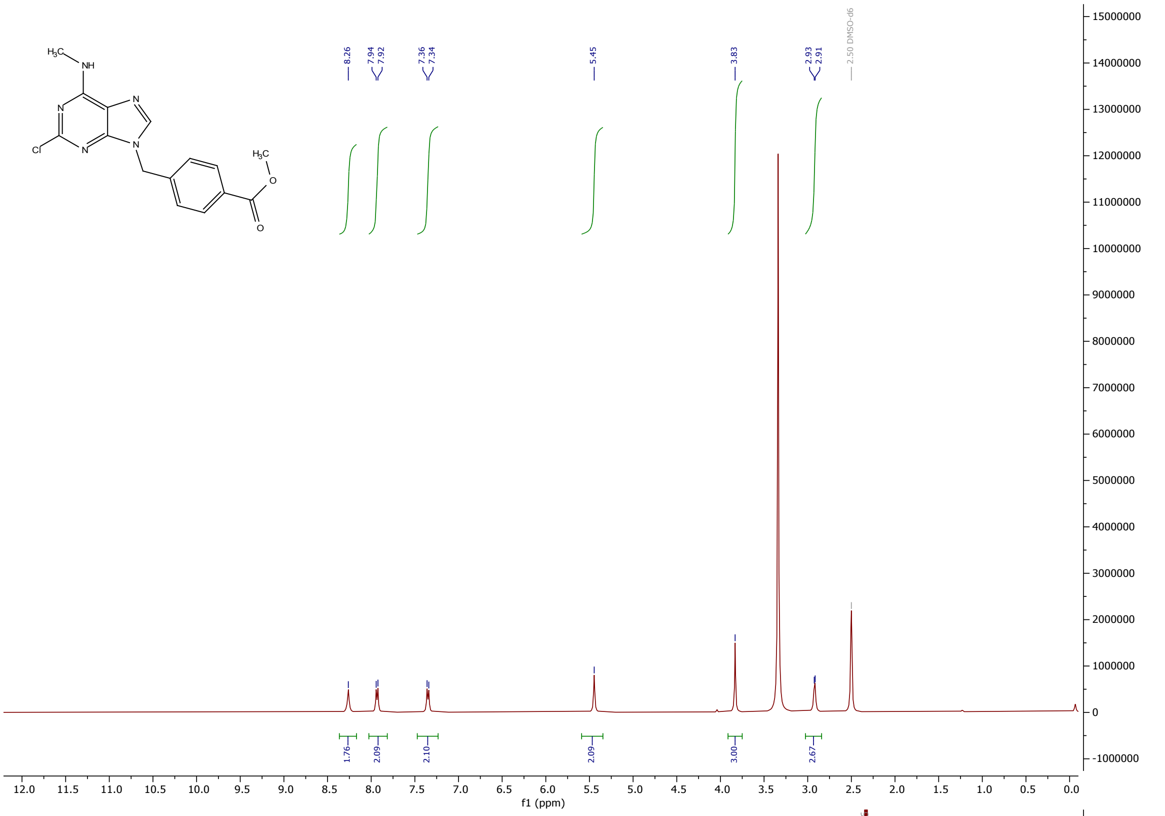


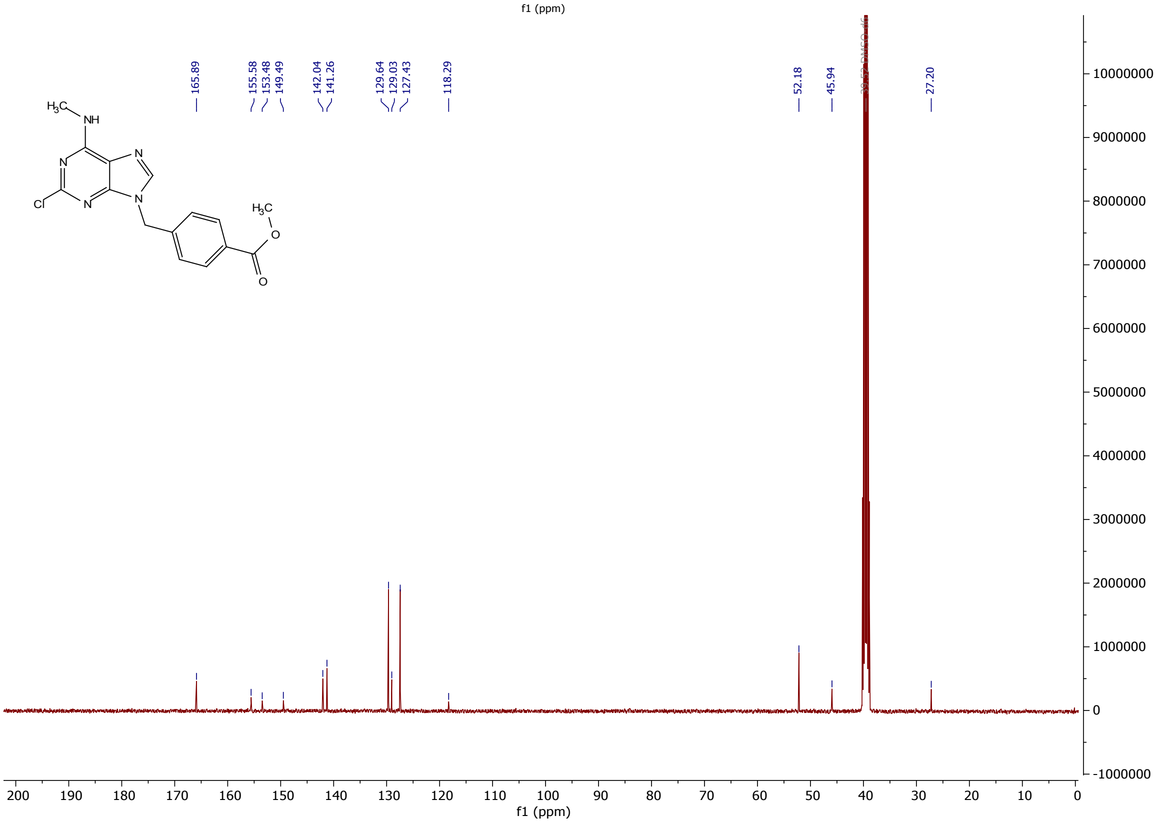

# 3-((2-chloro-6-(methylamino)-9H-purin-9-yl)methyl)benzoic acid 29

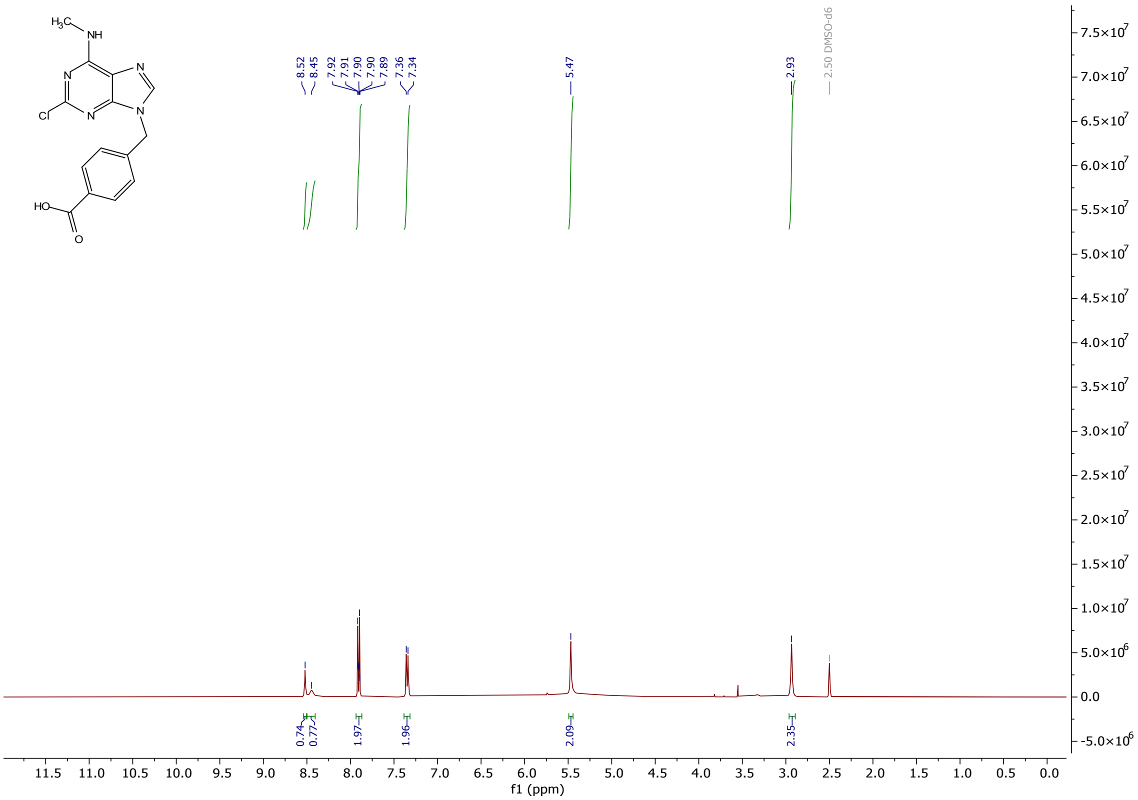


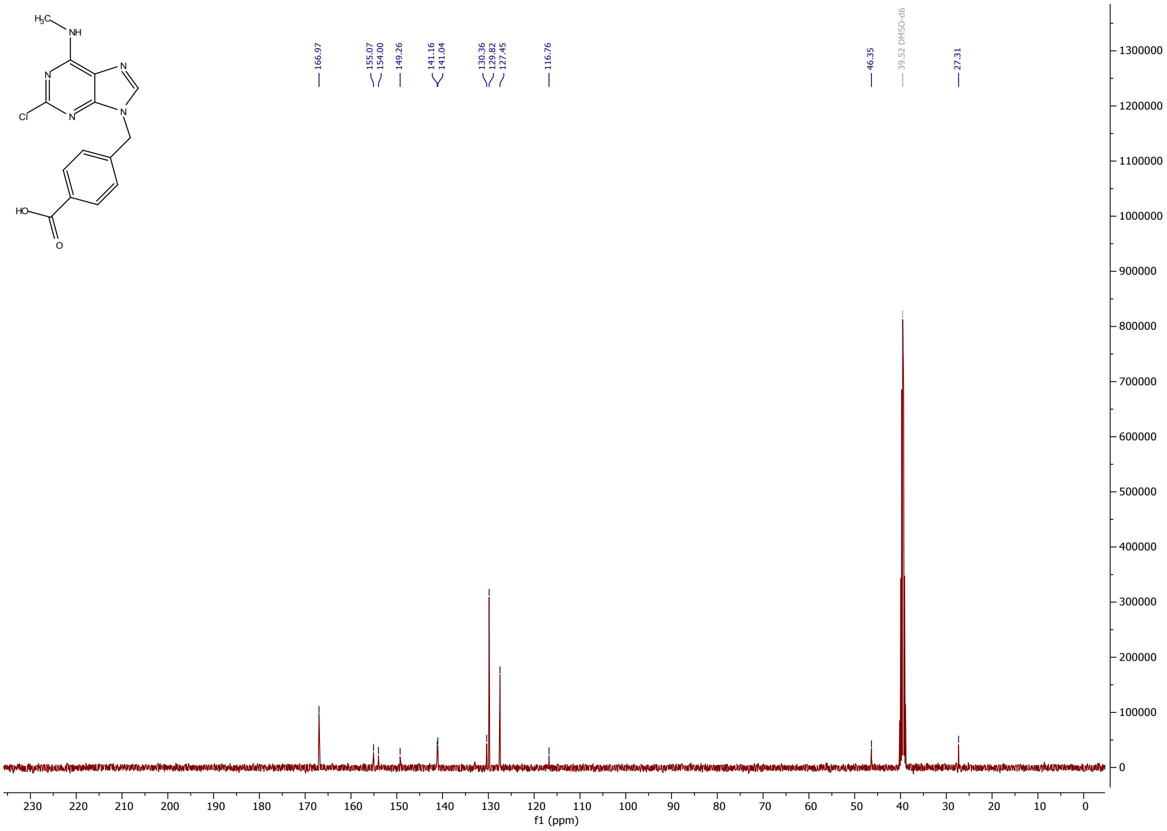

# (4-((2,6-dichloro-9H-purin-9-yl)methyl)phenyl)methanol 30

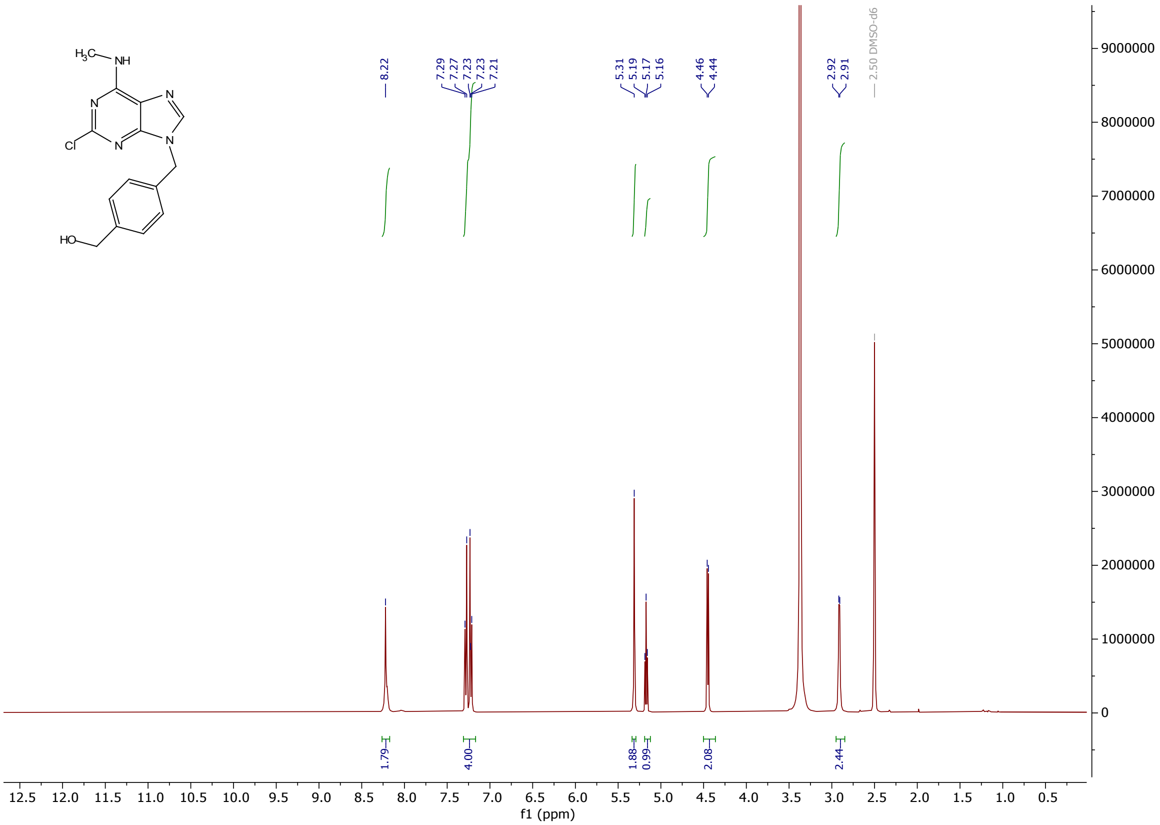


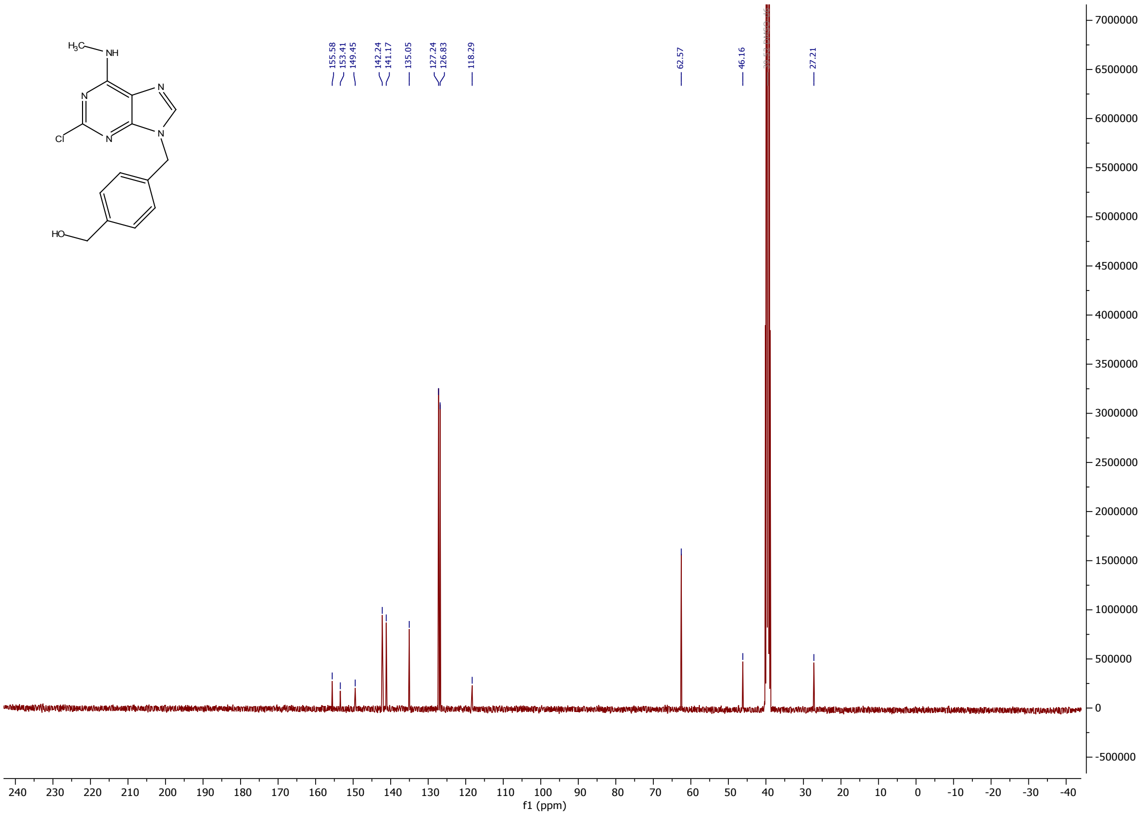

**N-(4-chloro-2-((2-chloro-6-(methylamino)-9H-purin-9-yl)methyl)phenyl) methanesulfonamide 31**

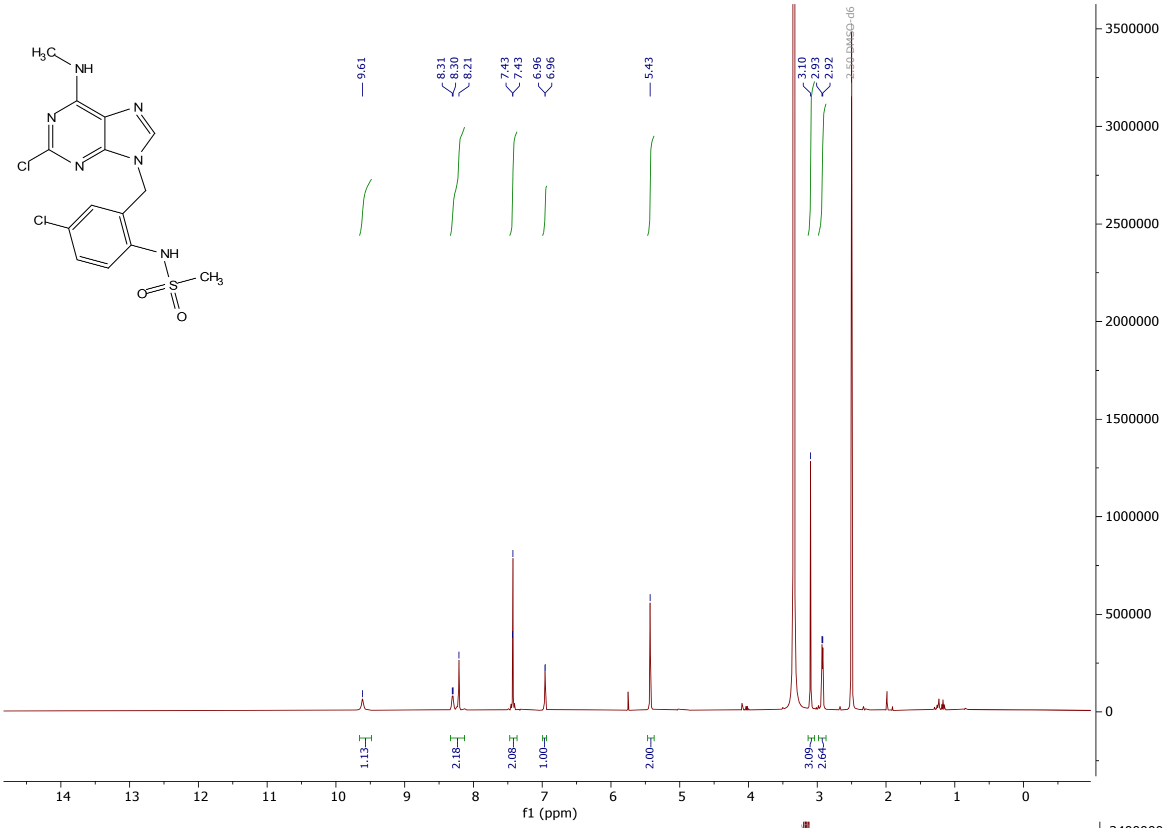

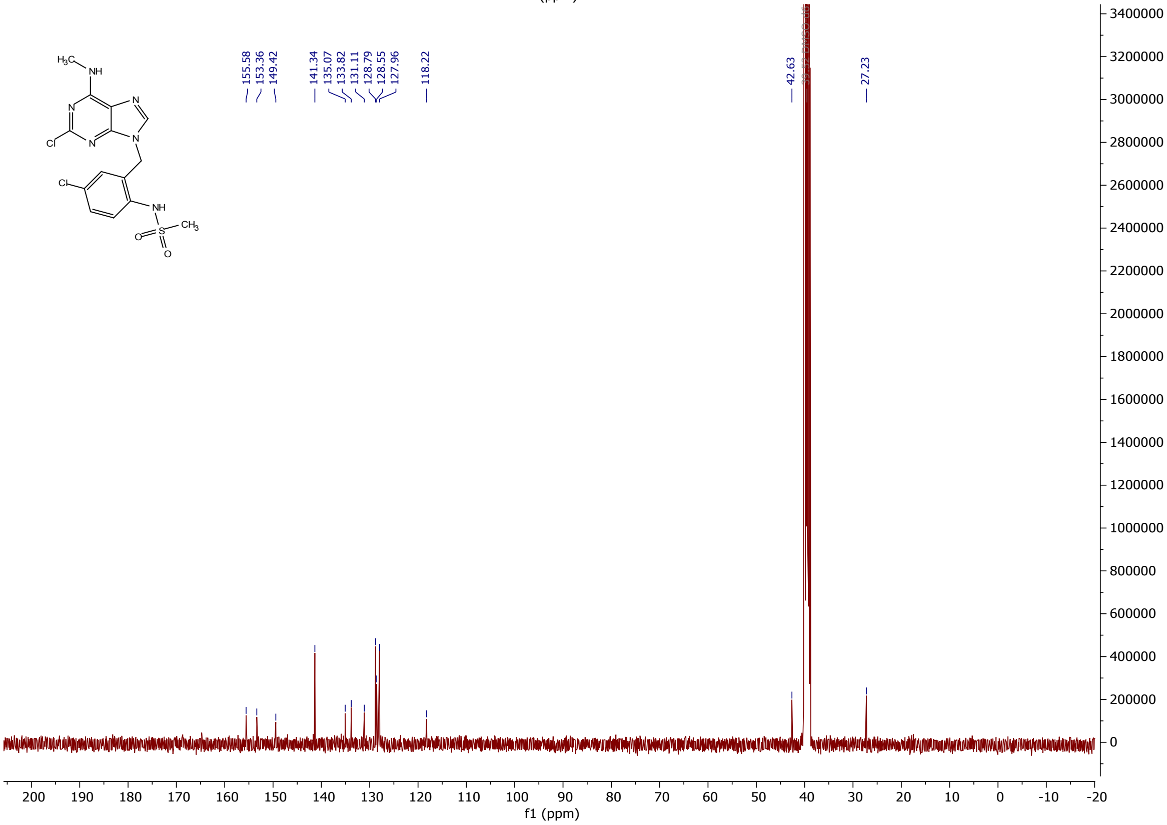

# 3-chloro-5-((2-chloro-6-(methylamino)-9H-purin-9-yl)methyl)benzoic acid 32

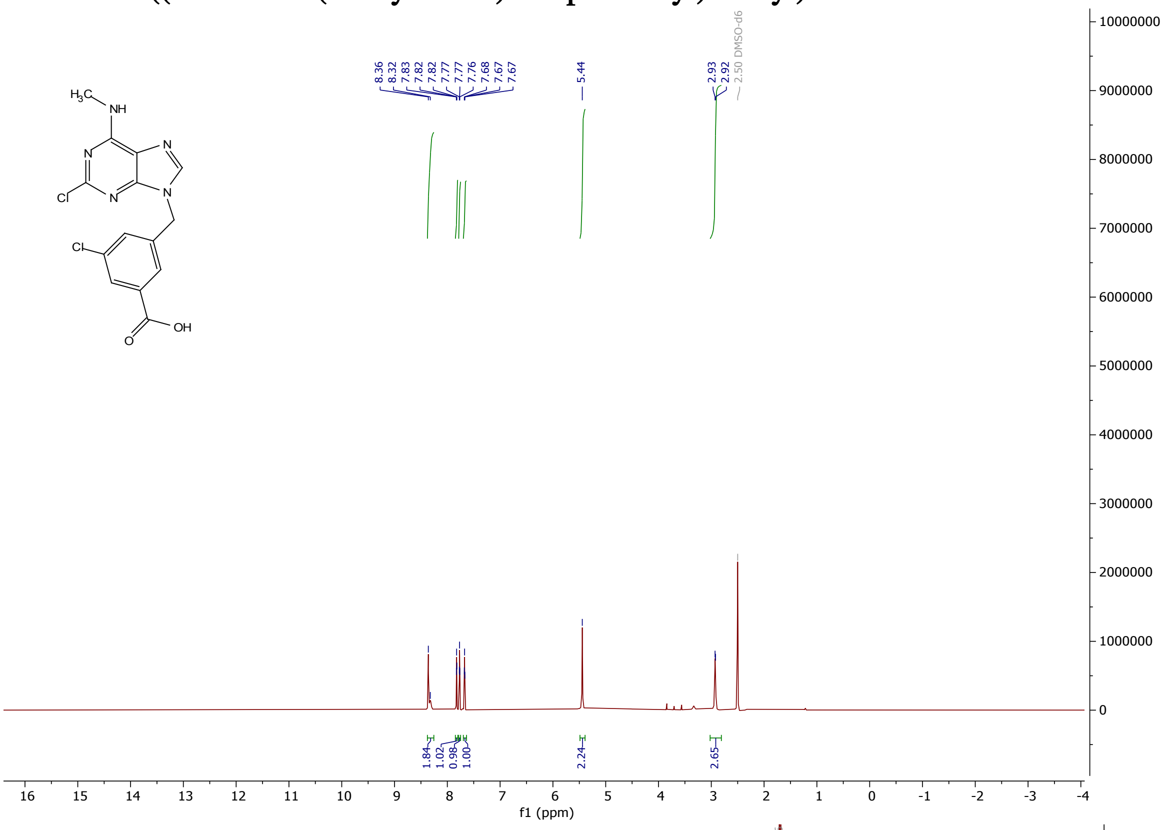


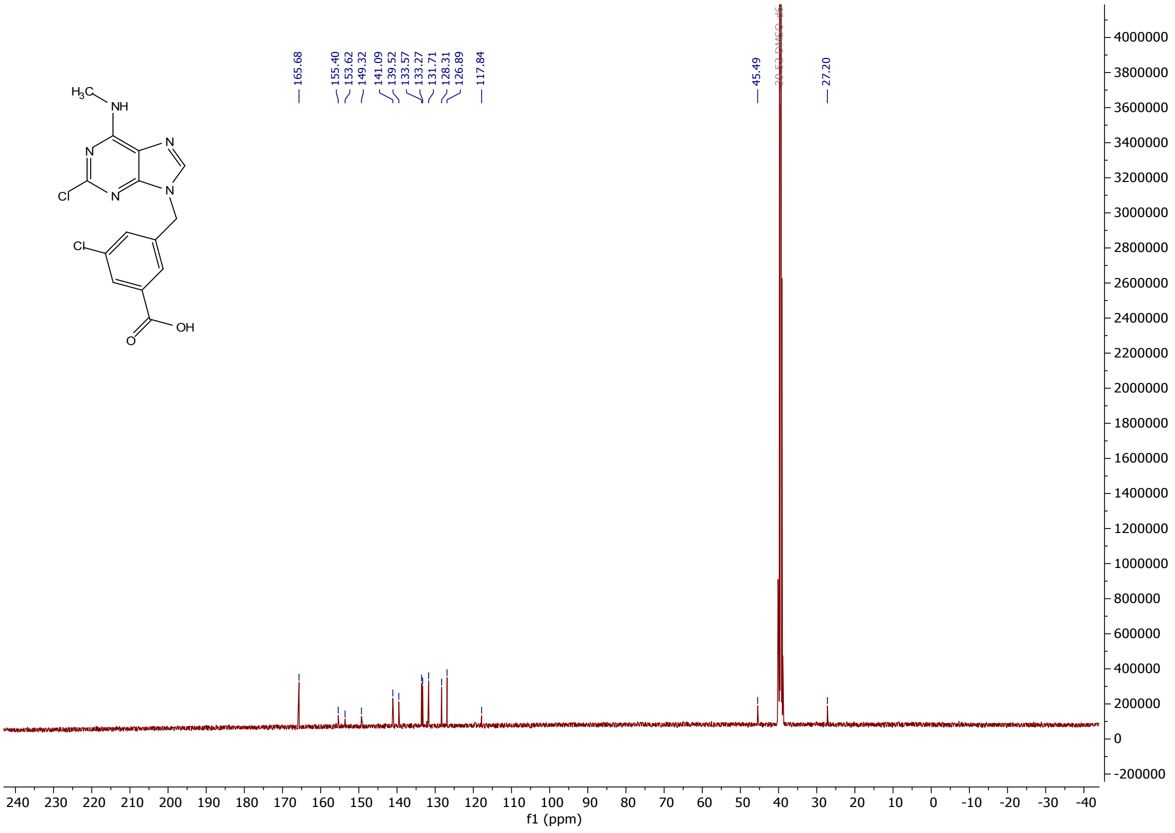

# methyl 3-chloro-5-((2,6-dichloro-9H-purin-9-yl)methyl)benzoate 33

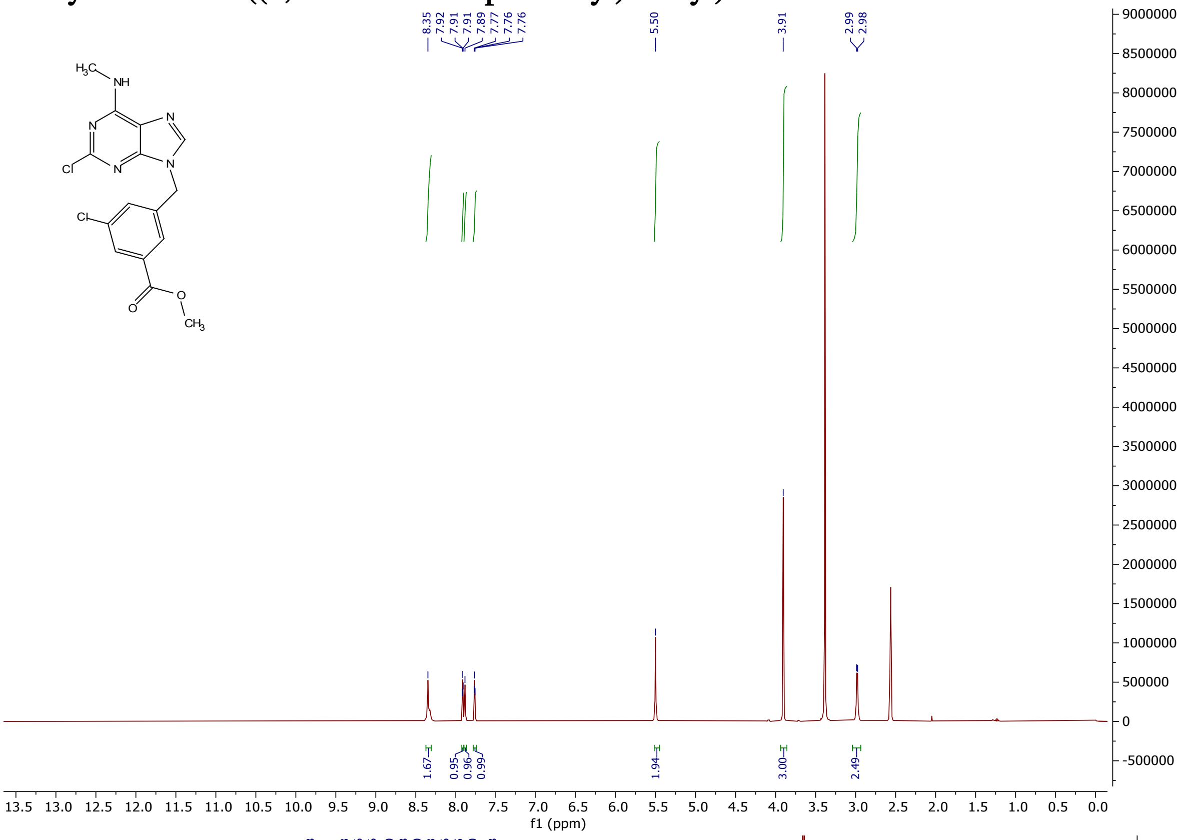


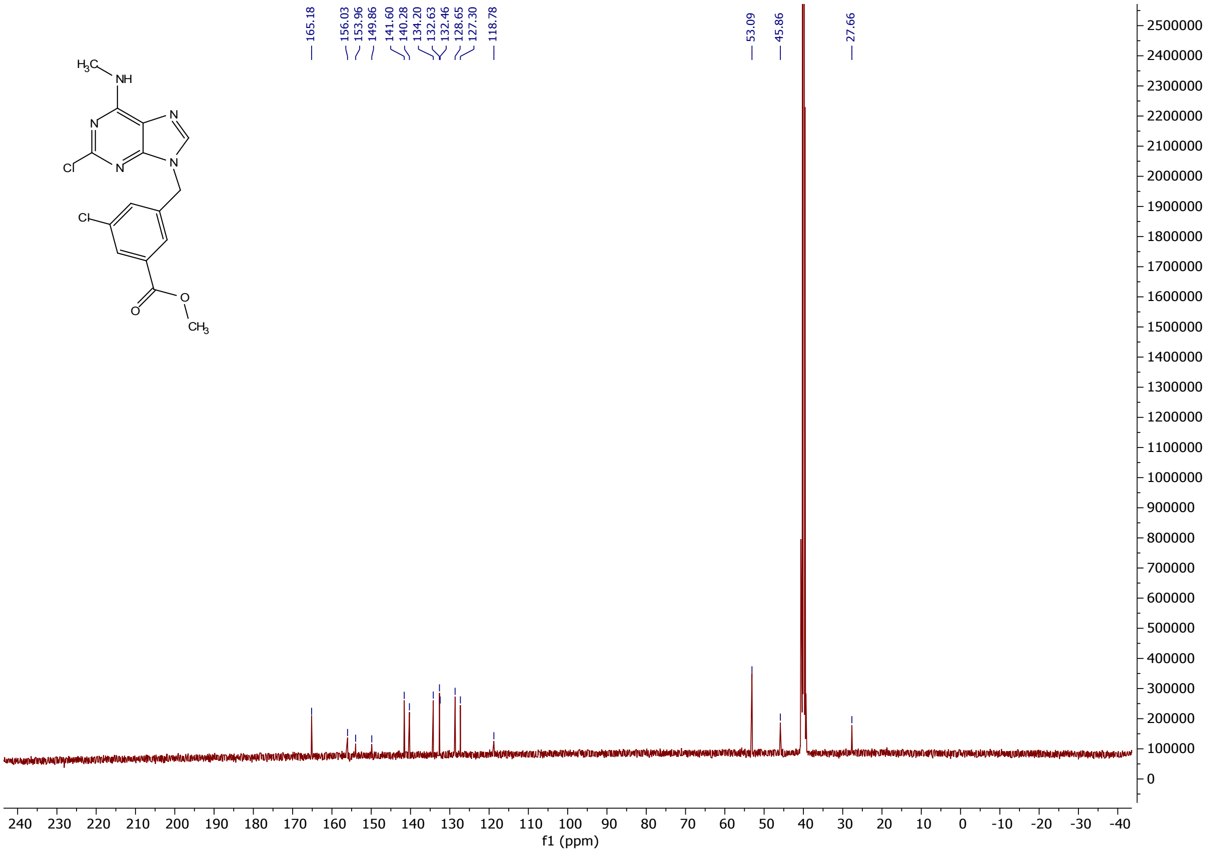

# 2-chloro-9-(3-chloro-4-methoxybenzyl)-N-methyl-9H-purin-6-amine 34

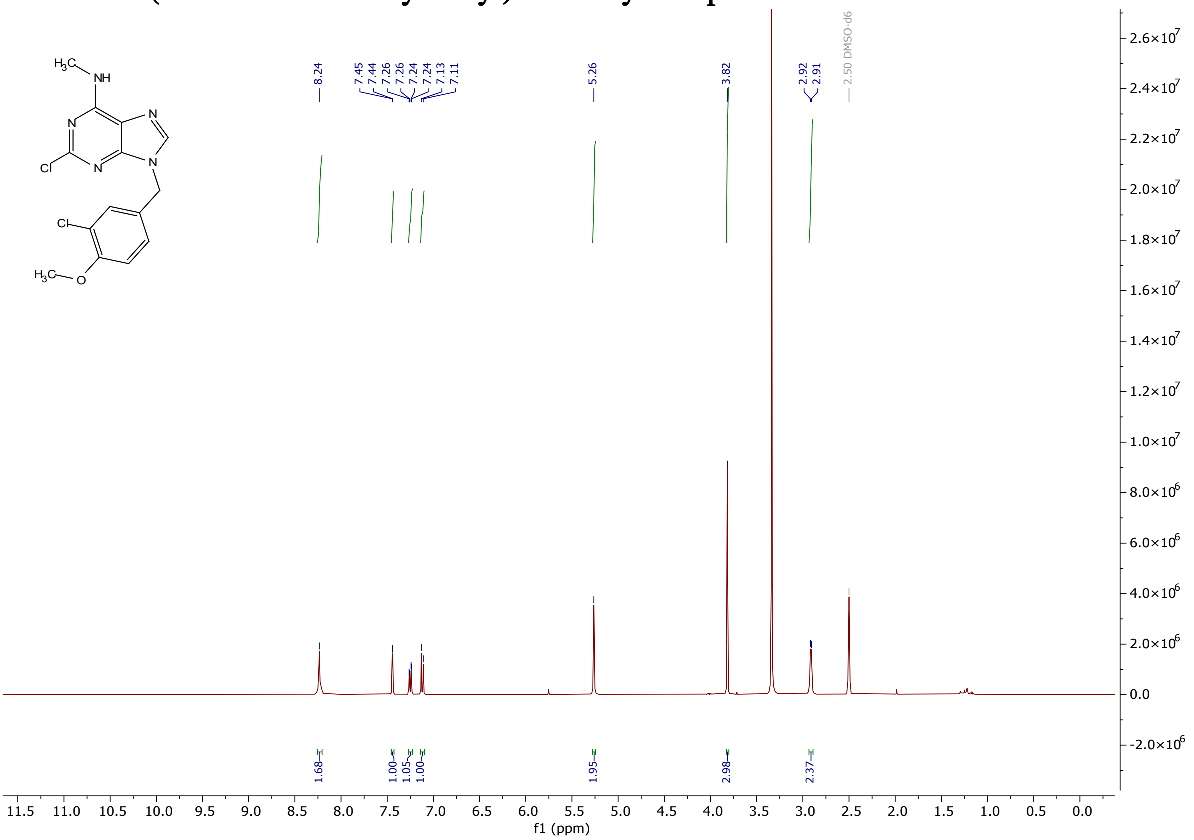


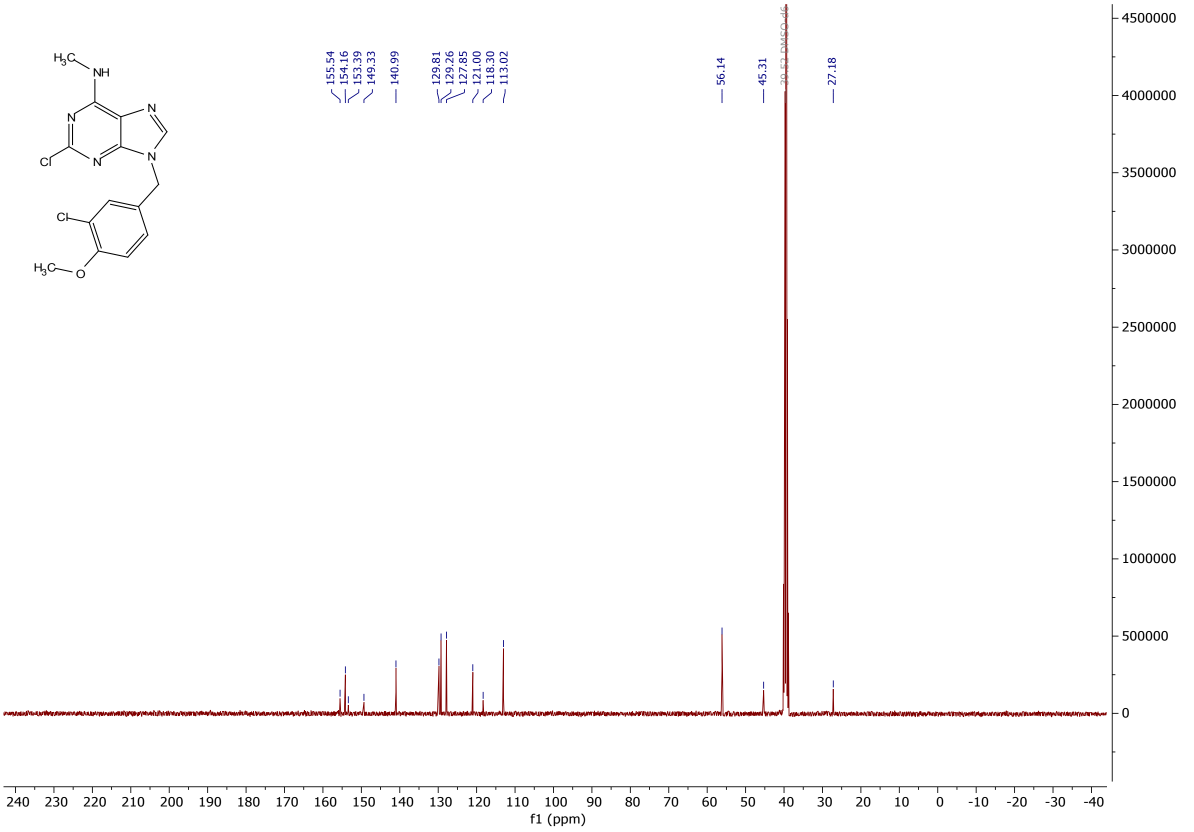

## 2-chloro-9-(3,5-dichloro-4-methoxybenzyl)-N-methyl-9H-purin-6-amine 35

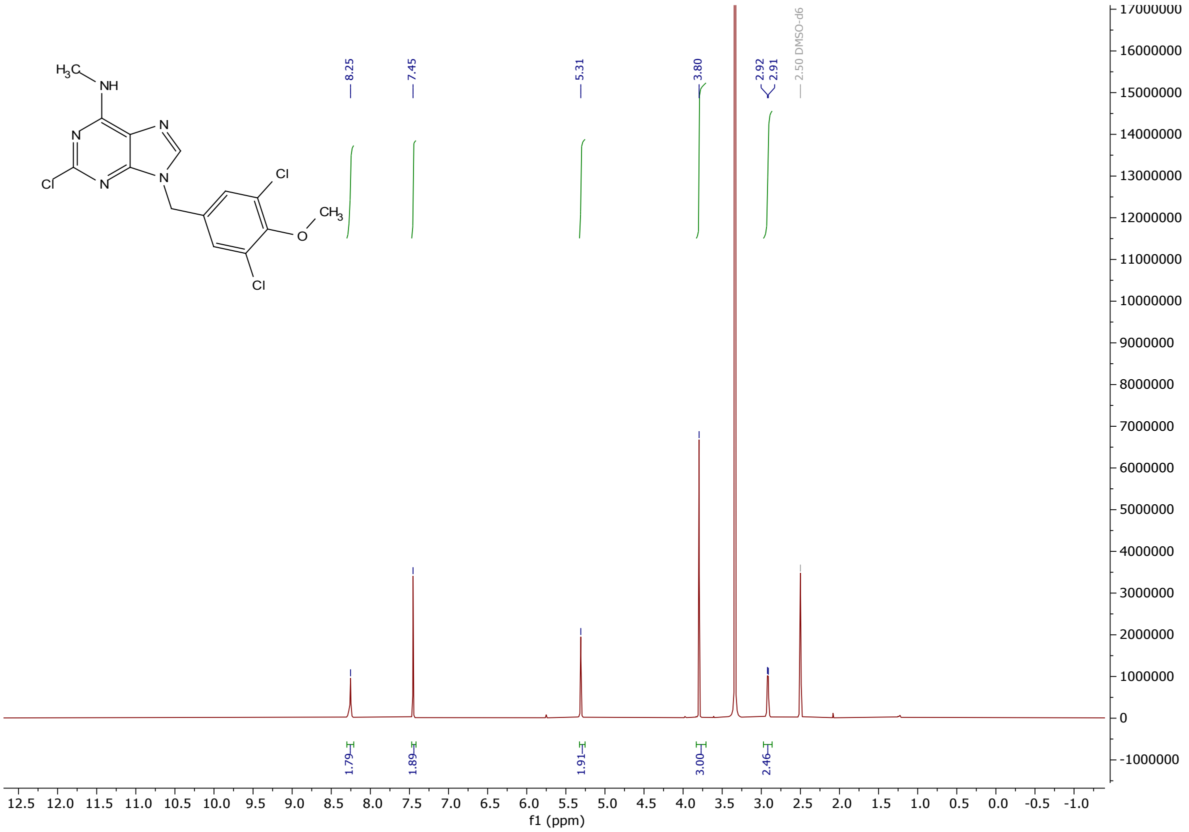


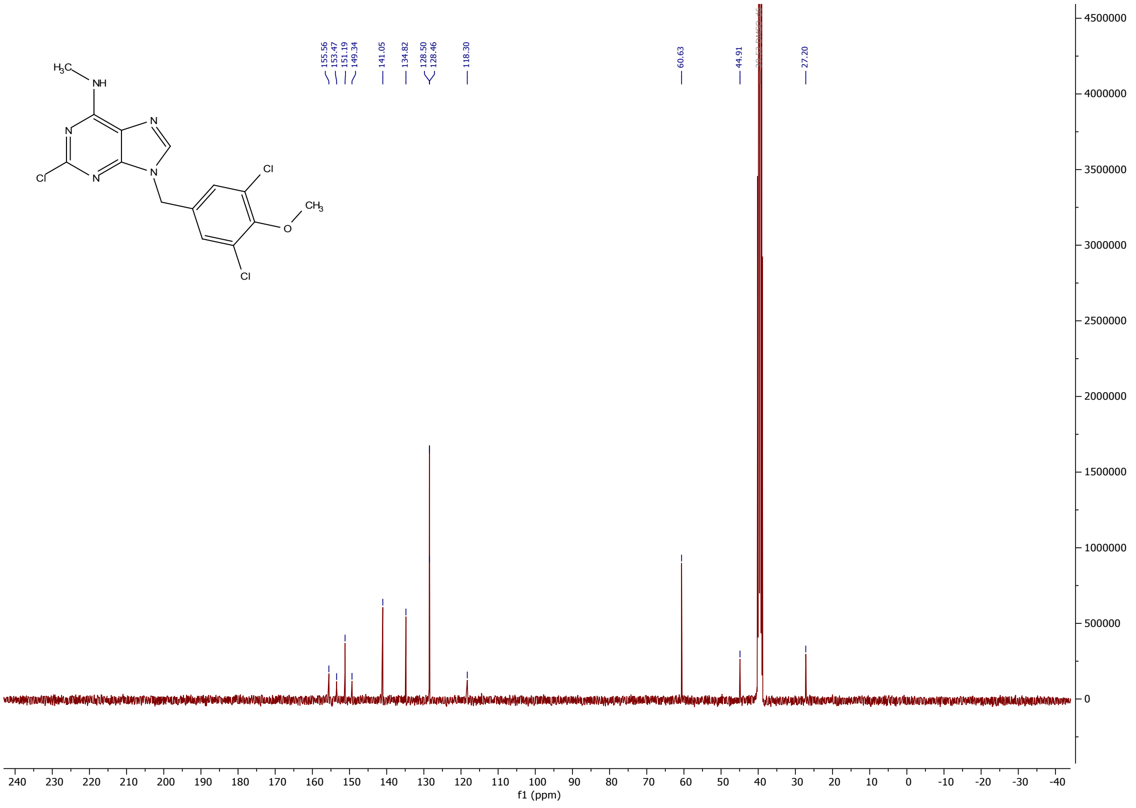

## 9-(3-chlorobenzyl)-2-fluoro-N-methyl-9H-purin-6-amine 36

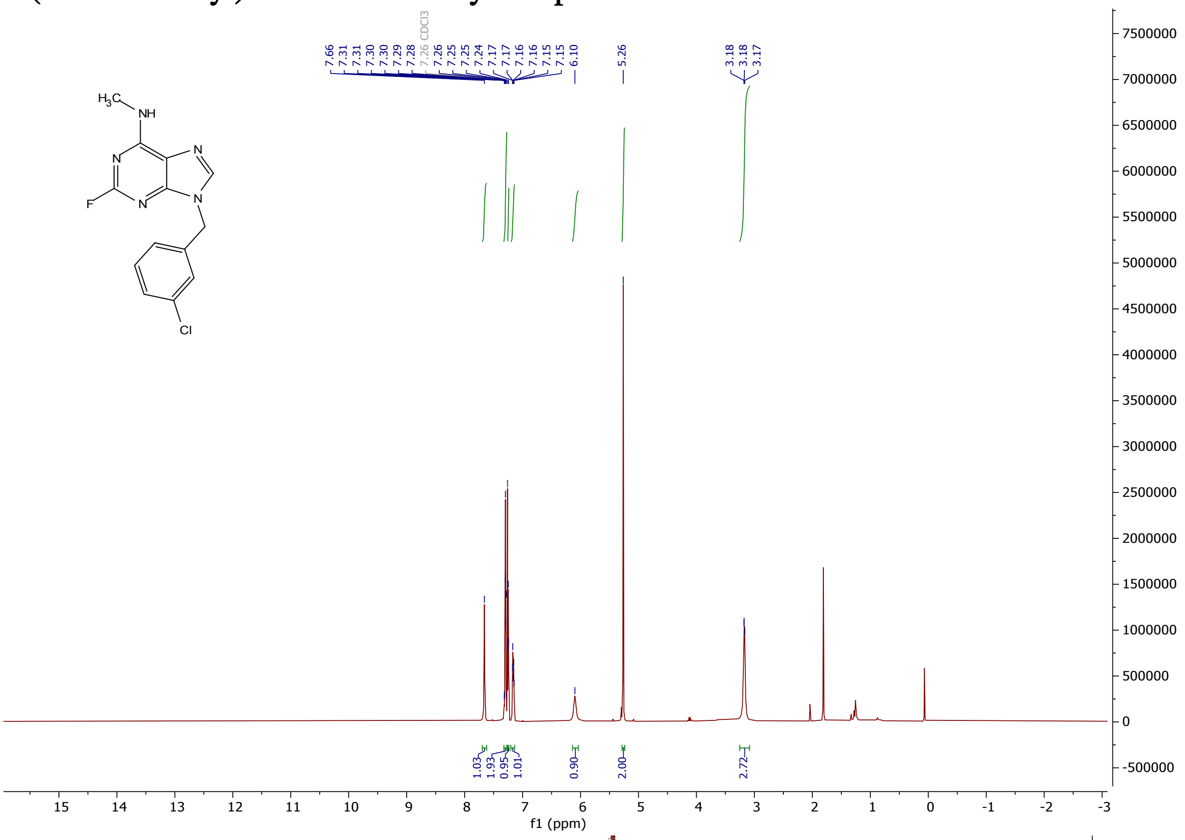

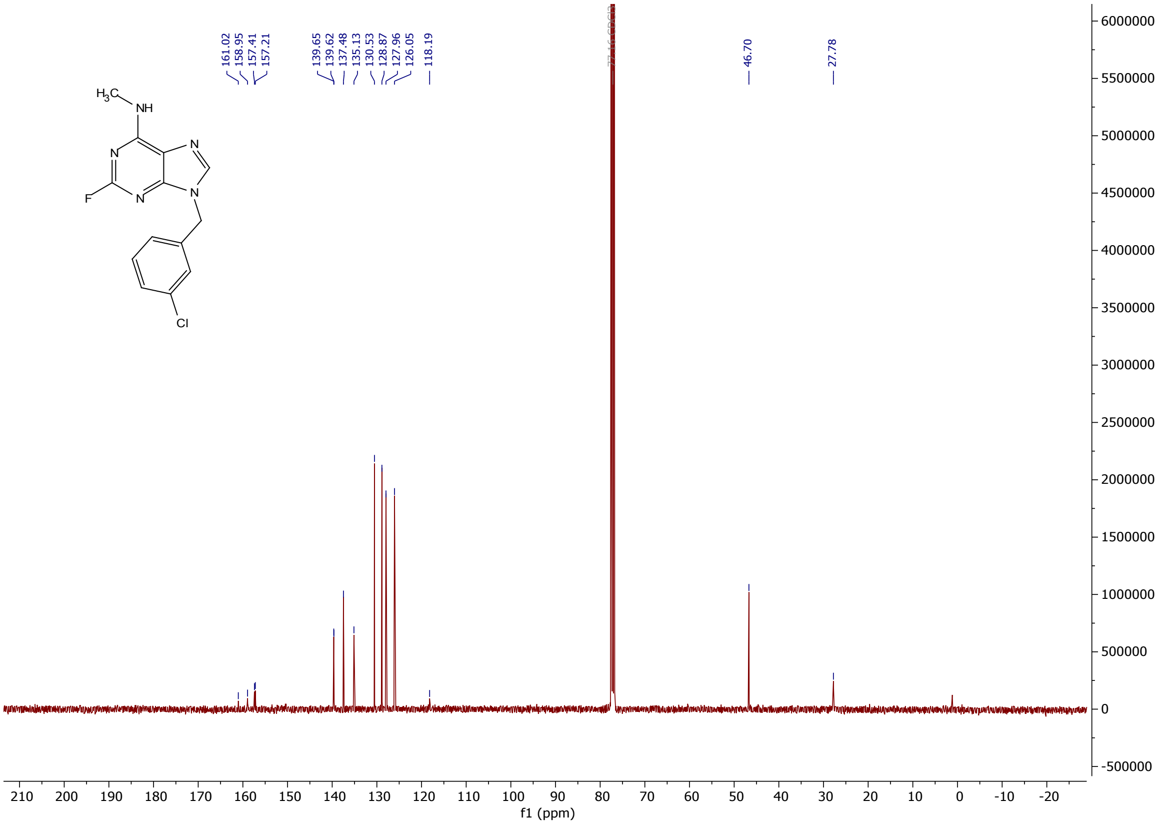

## 2-chloro-9-(3-chlorobenzyl)-N-cyclopropyl-9H-purin-6-amine 37

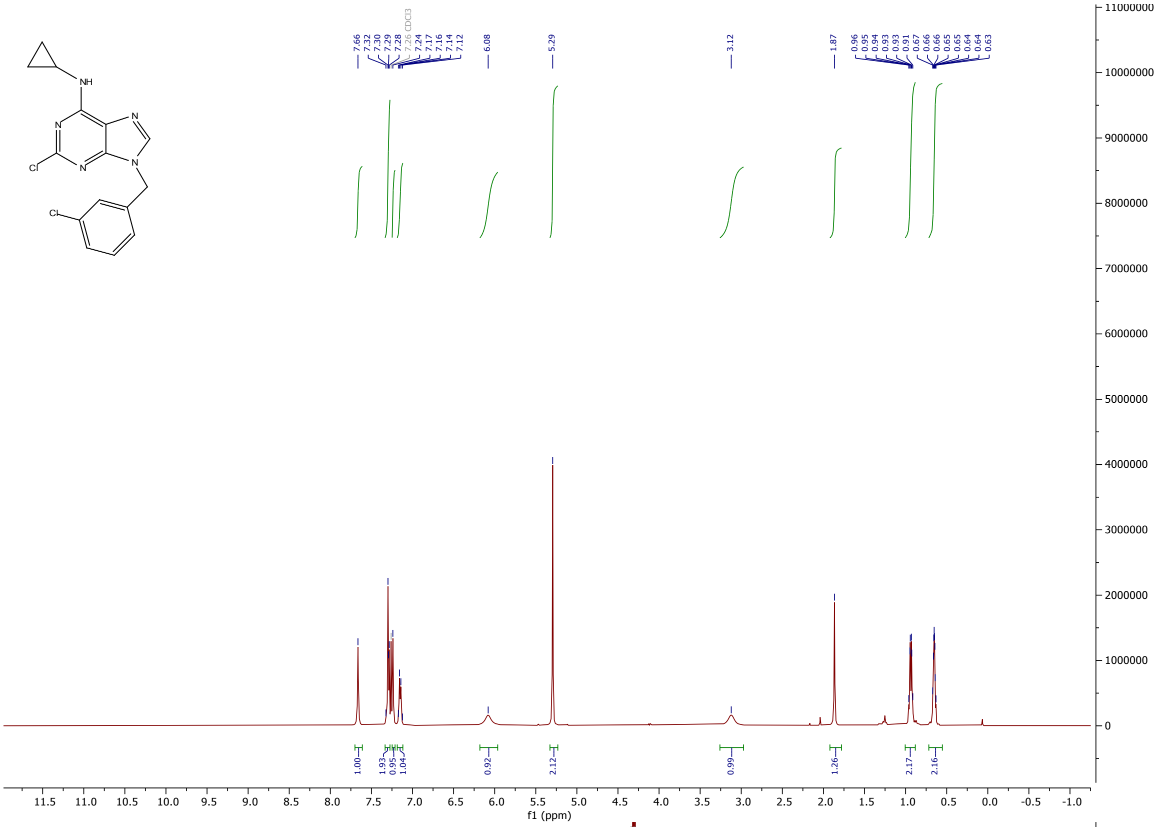

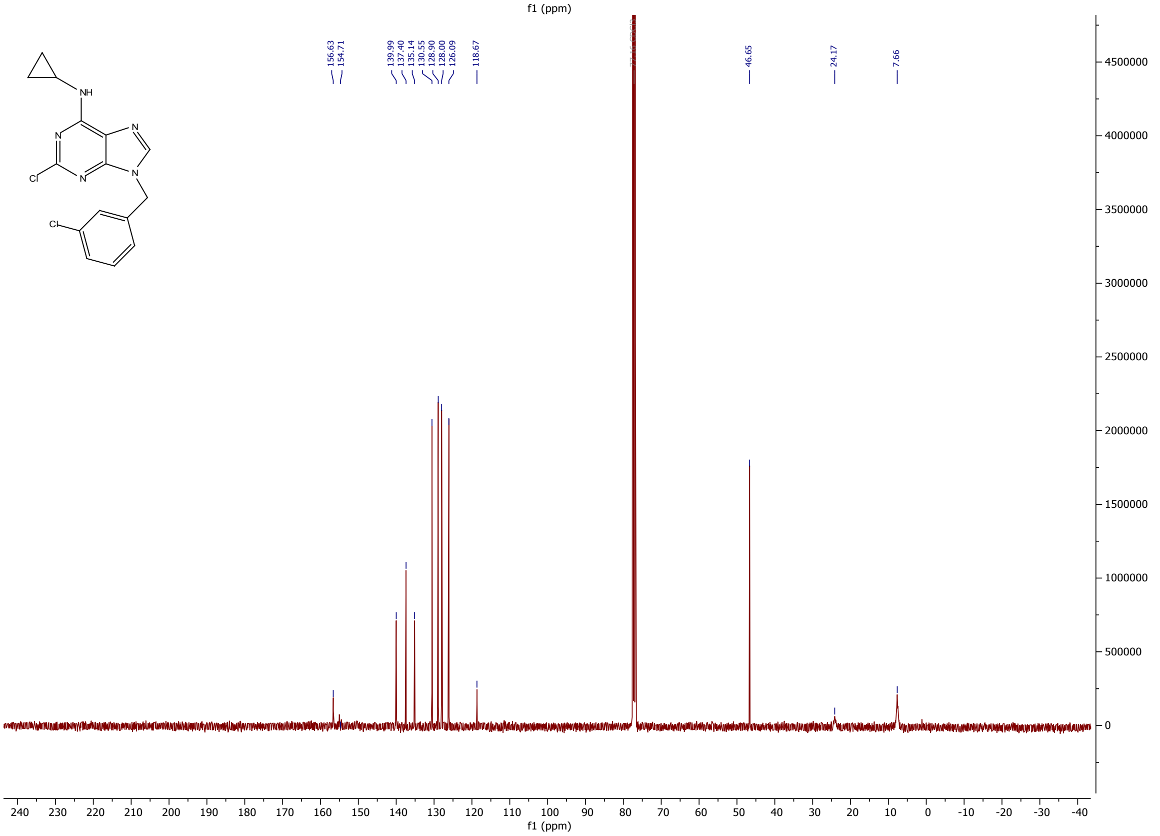

# 2,6-dichloro-9-(3-chlorobenzyl)-9H-purine 38

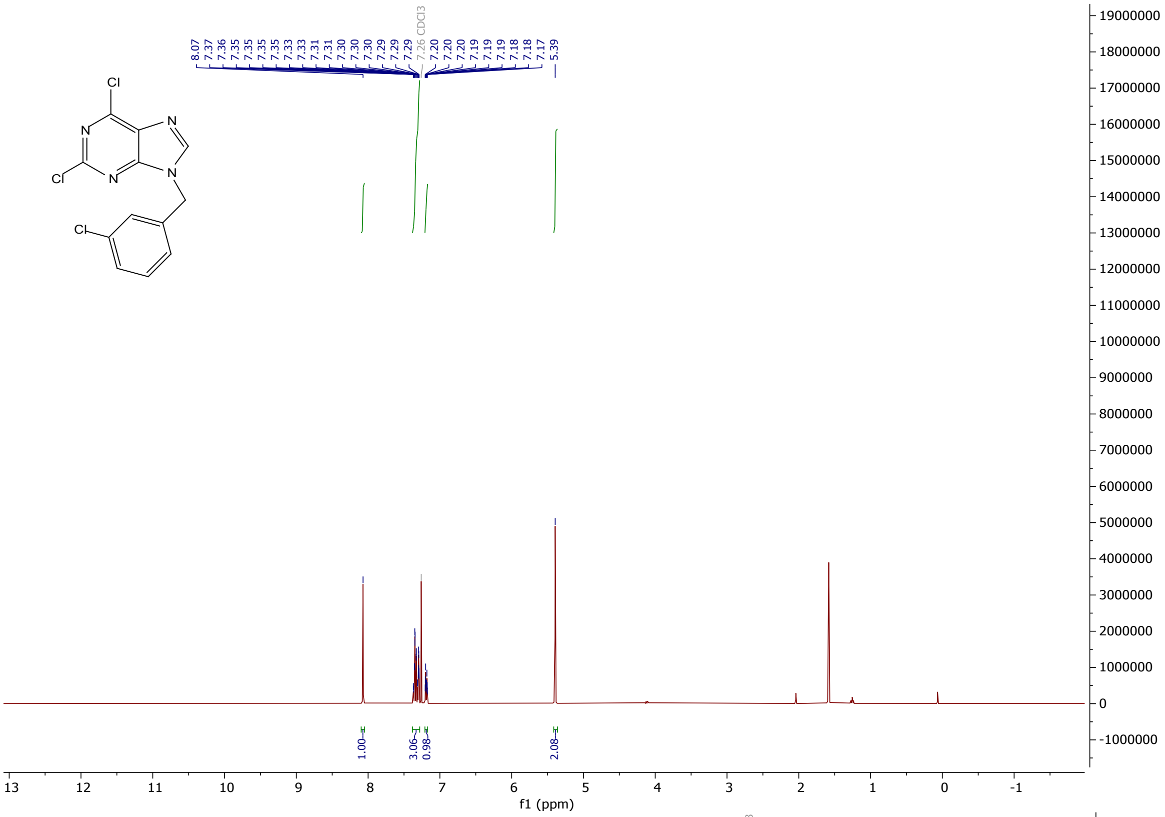


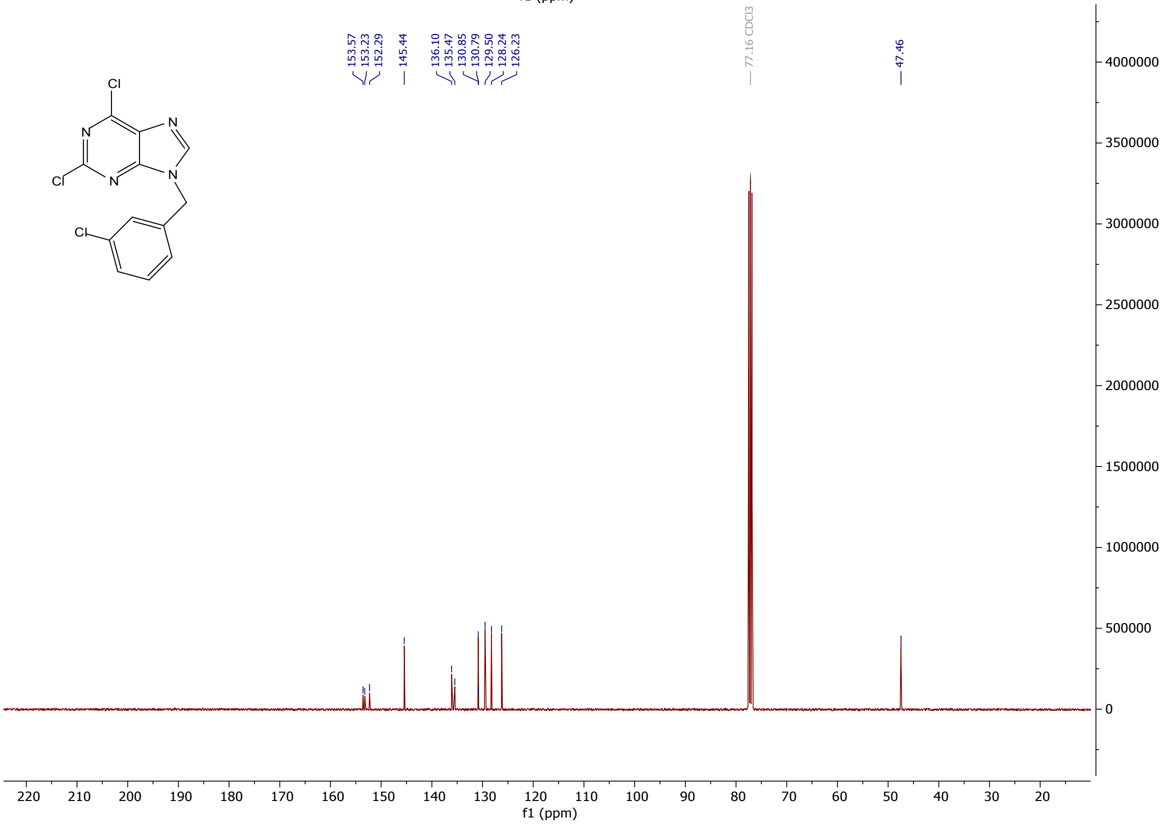

# 6-chloro-9-(3-chlorobenzyl)-N-methyl-9H-purin-2-amine 39

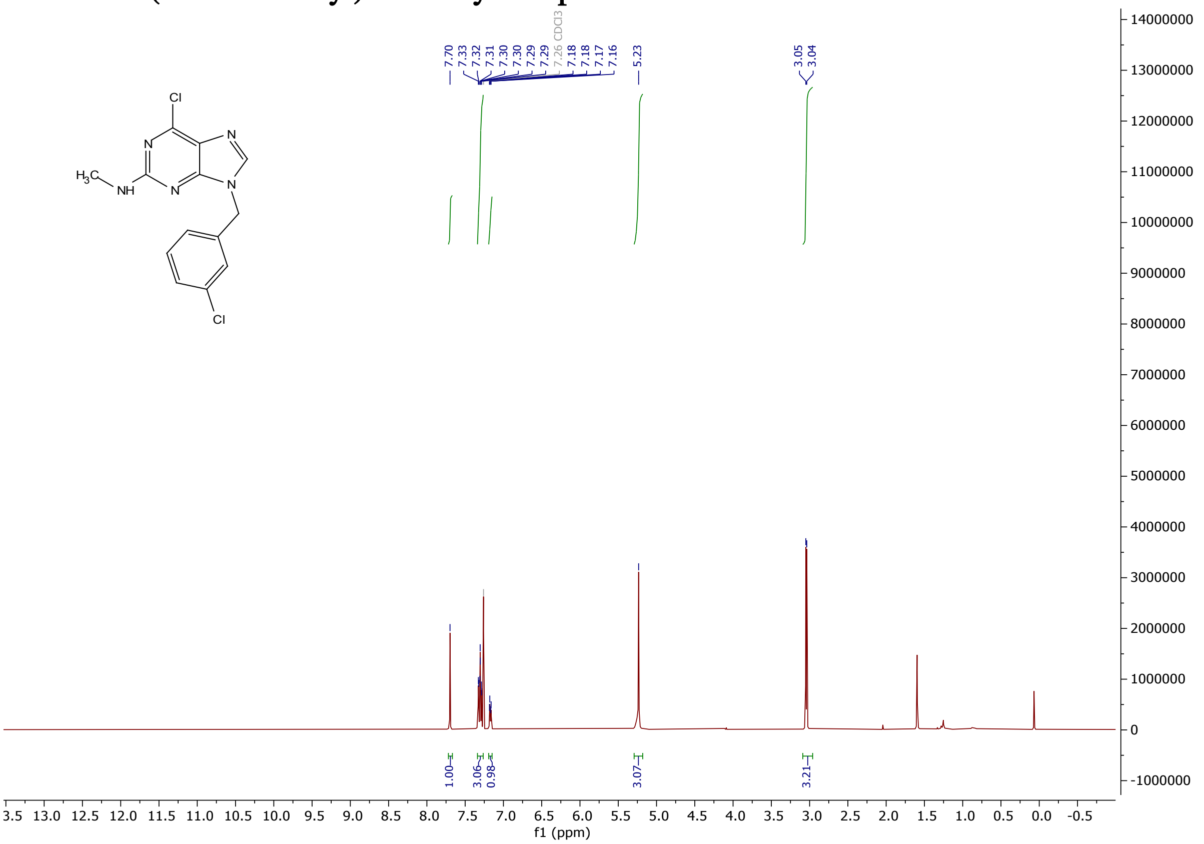


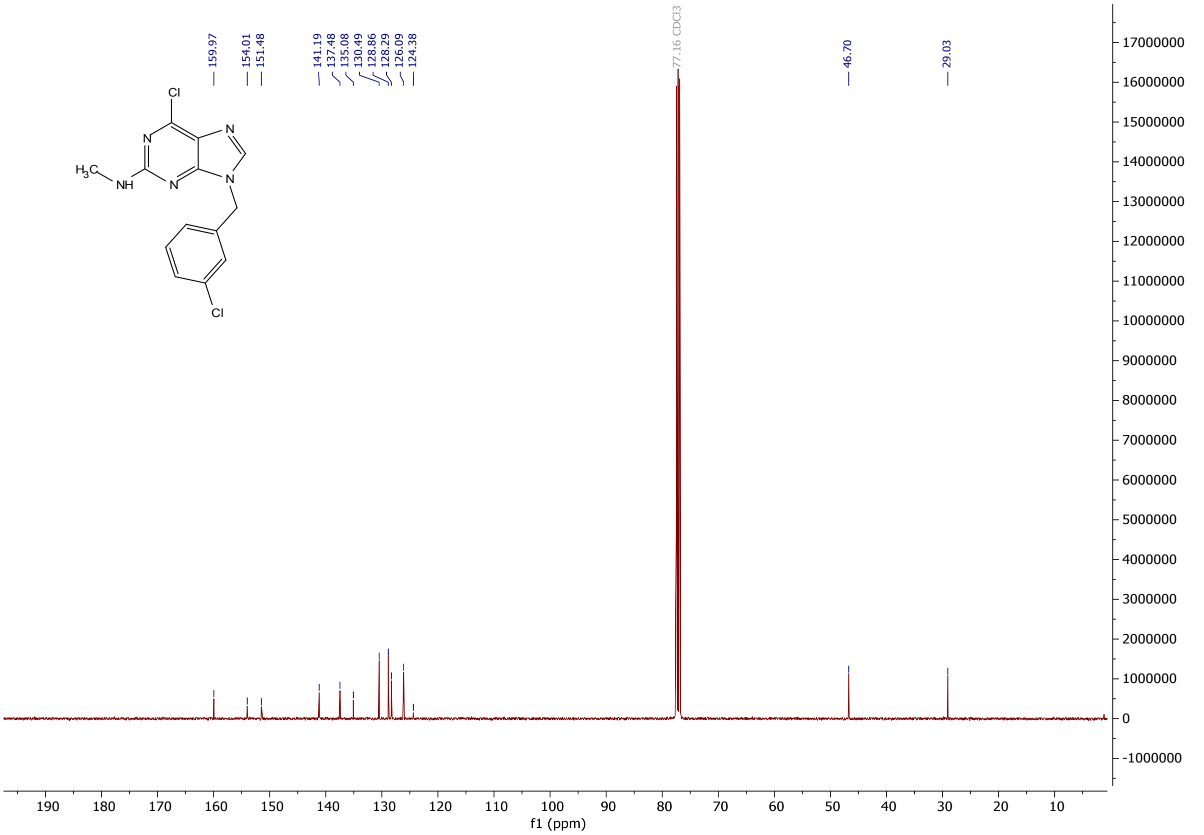

## 9. HPLC traces of final compounds

### 2-chloro-N-methyl-9-phenyl-9H-purin-6-amine 4

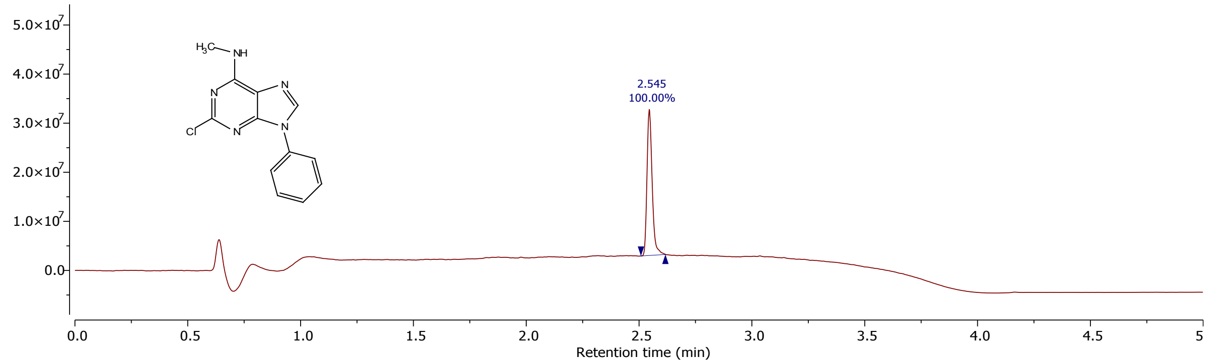


### 9-benzyl-2-chloro-N-methyl-9H-purin-6-amine 5

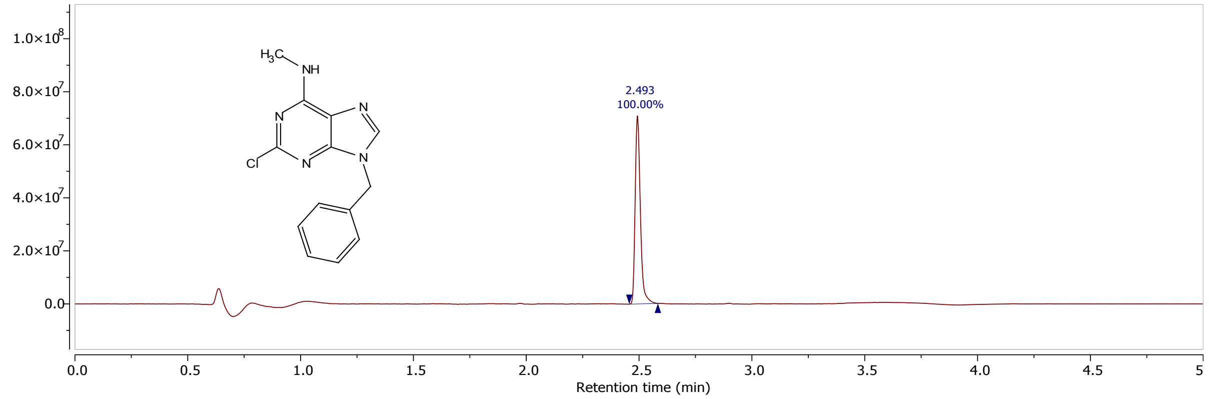


### 2-chloro-N-methyl-9-(pyridin-4-ylmethyl)-9H-purin-6-amine 6

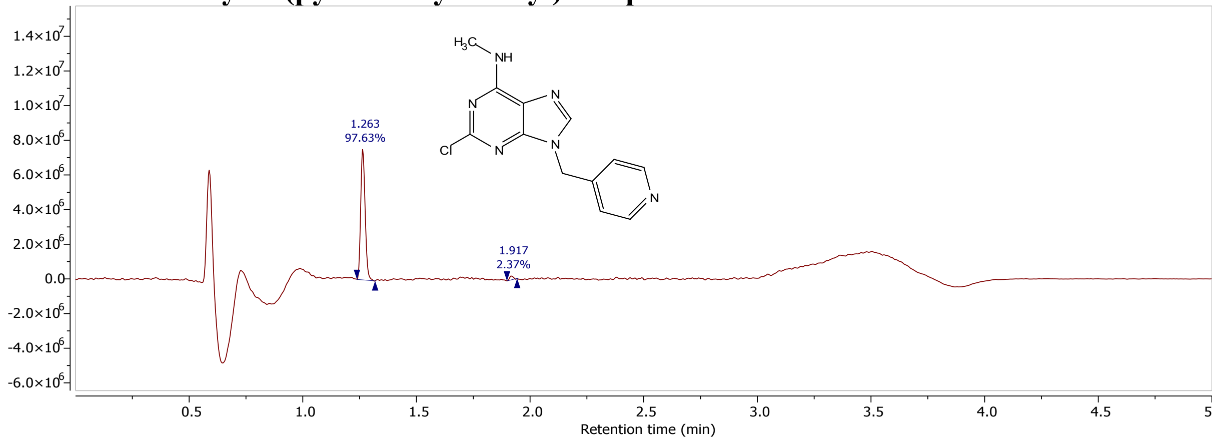

## 2-chloro-N-methyl-9-(tetrahydro-2H-pyran-2-yl)-9H-purin-6-amine 7

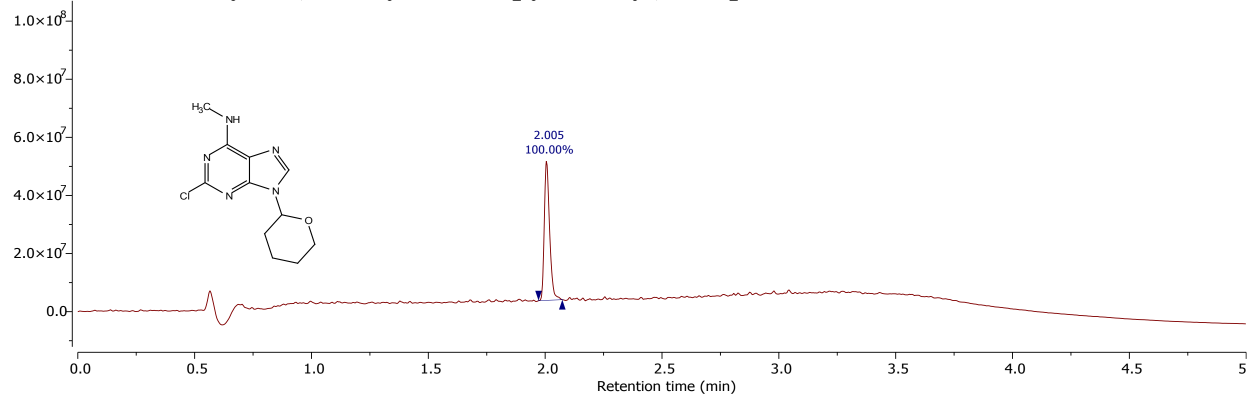


## 2-chloro-9-(cyclohexylmethyl)-N-methyl-9H-purin-6-amine 8

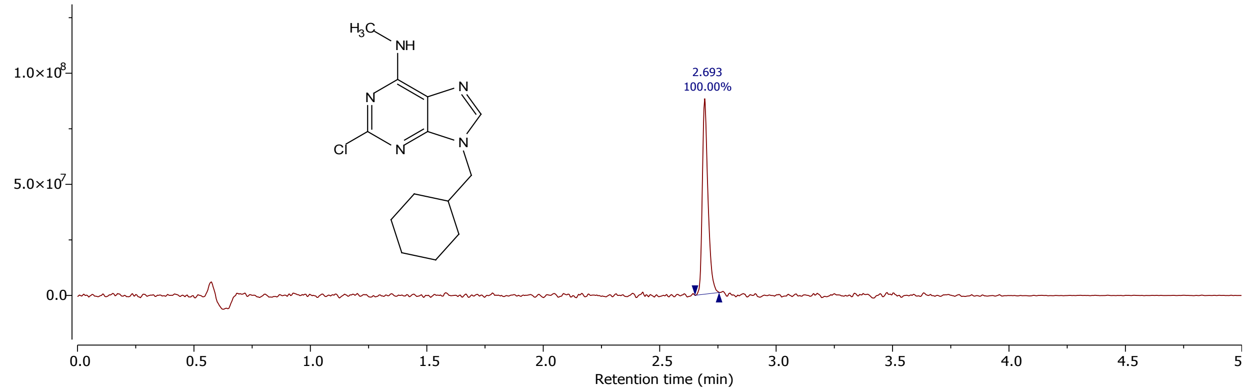


## 2-chloro-N-methyl-9-((3-(trifluoromethyl)cyclohexyl)methyl)-9H-purin-6-amine

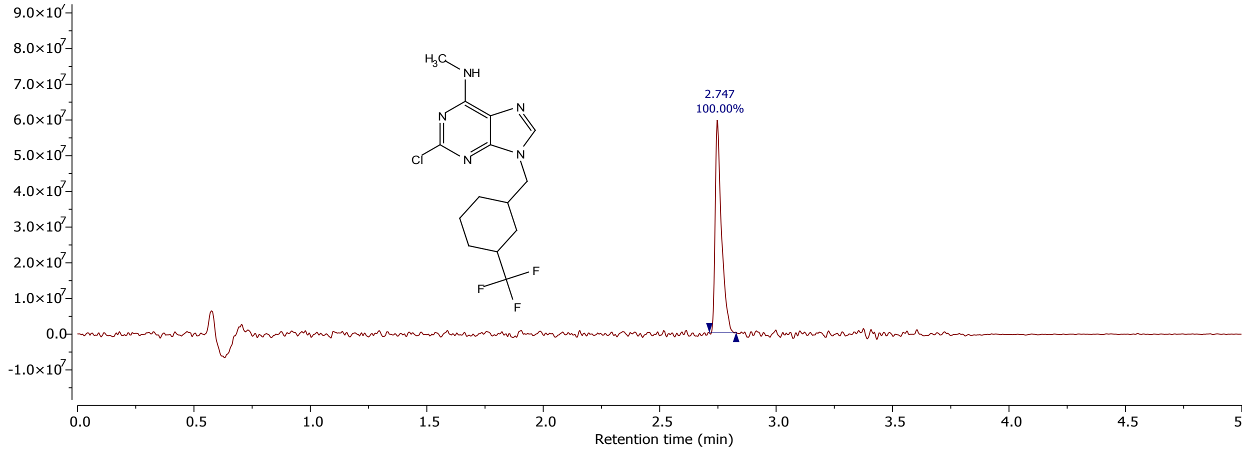

## 3-(2-chloro-6-(methylamino)-9H-purin-9-yl)benzoic acid 10

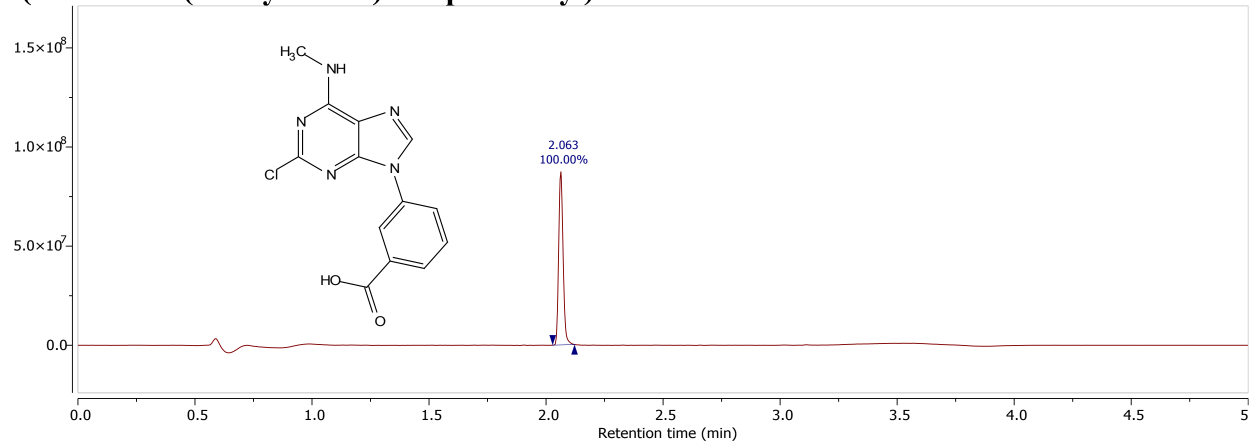


## 2-(2-chloro-6-(methylamino)-9H-purin-9-yl)-N-phenylacetamide 11

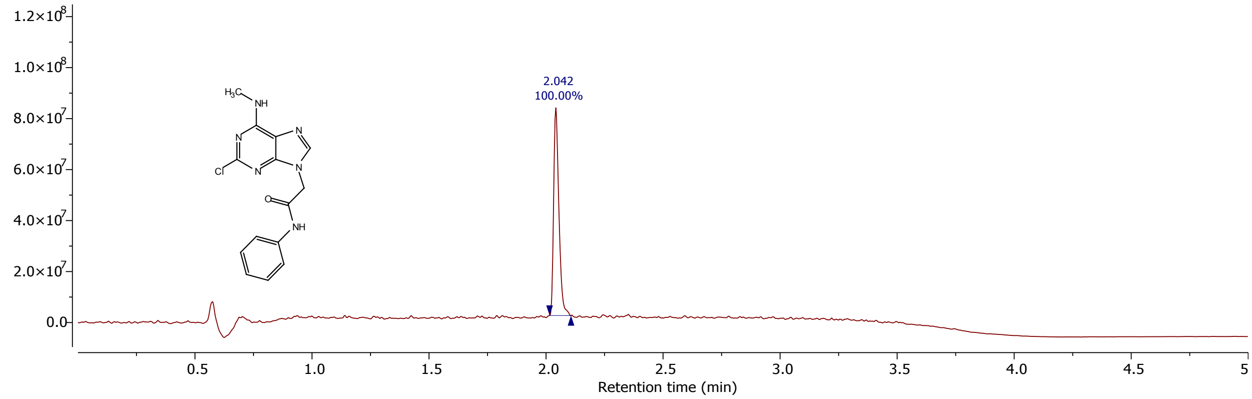


## 9-(2-aminobenzyl)-2-chloro-N-methyl-9H-purin-6-amine 12

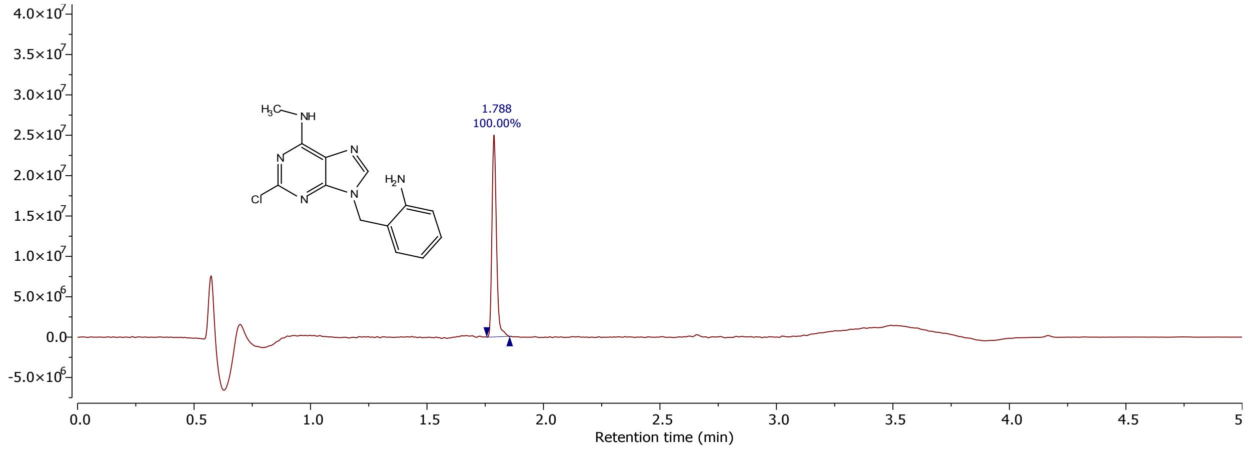

## N-(2-((2-chloro-6-(methylamino)-9H-purin-9- yl) methyl)phenyl) methanesulfonamide 13

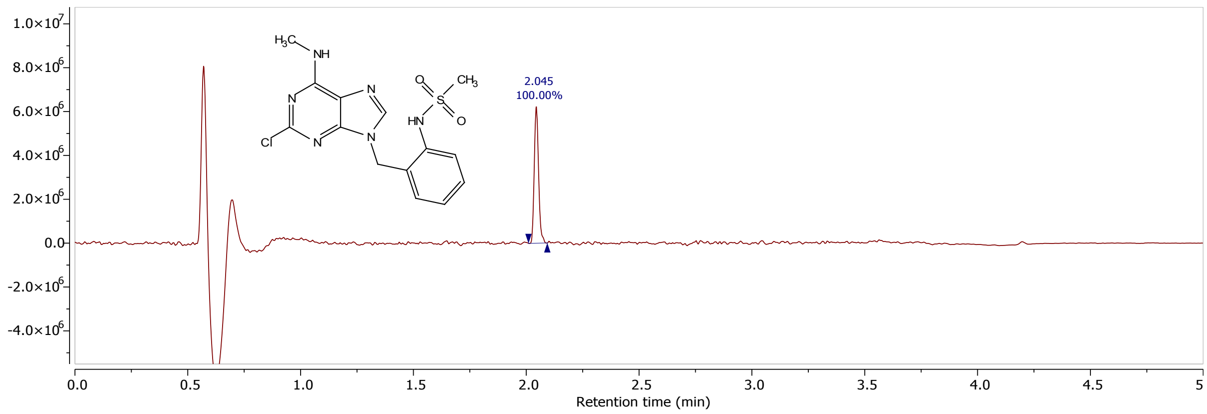


## N-(2-((2-chloro-6-(methylamino)-9H-purin-9-yl)methyl)phenyl)-4-methylbenzene sulfonamide 14

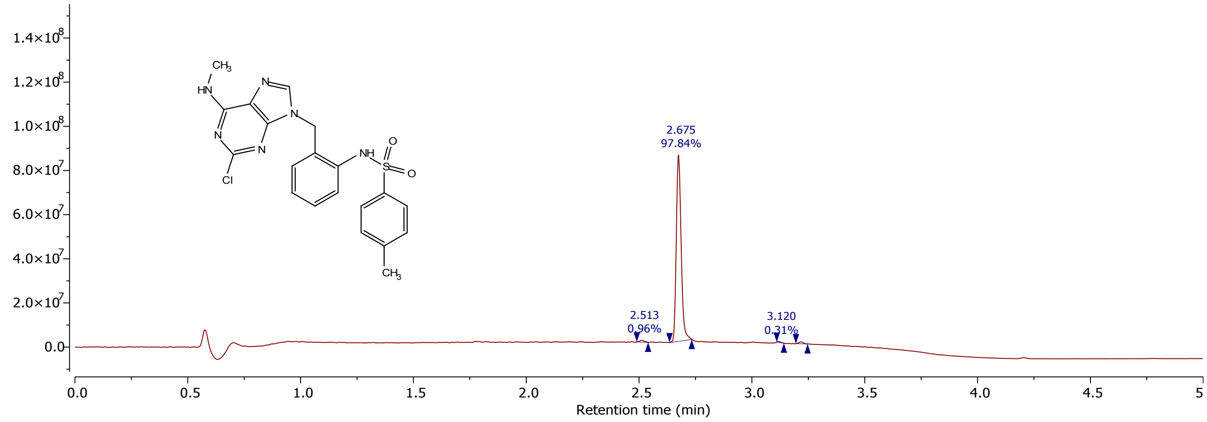


## N-(2-((2-chloro-6-(methylamino)-9H-purin-9-yl)methyl)phenyl)-2,2,2-trifluoroacetamide 15

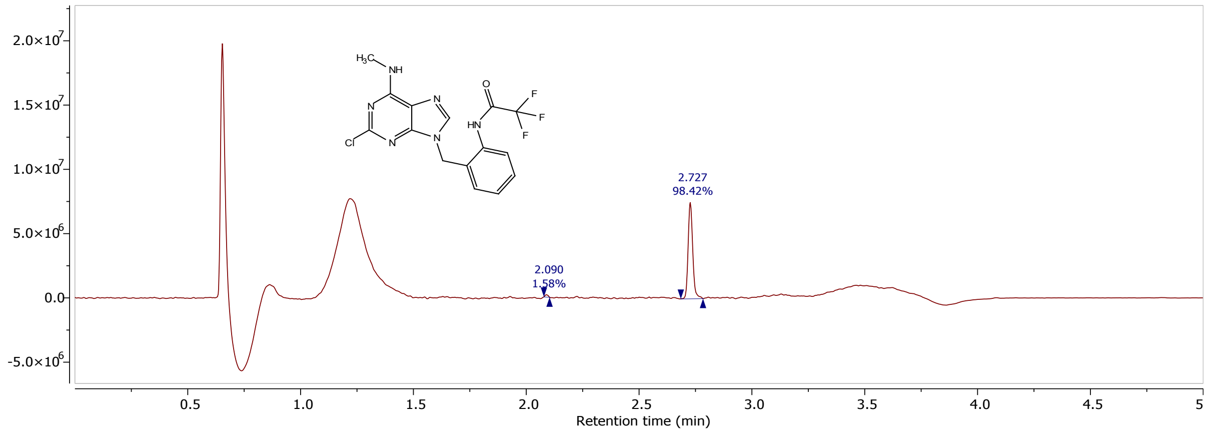

## *2*-chloro-9-(2-(difluoromethyl)benzyl)-N-methyl-9H-purin-6-amine 16

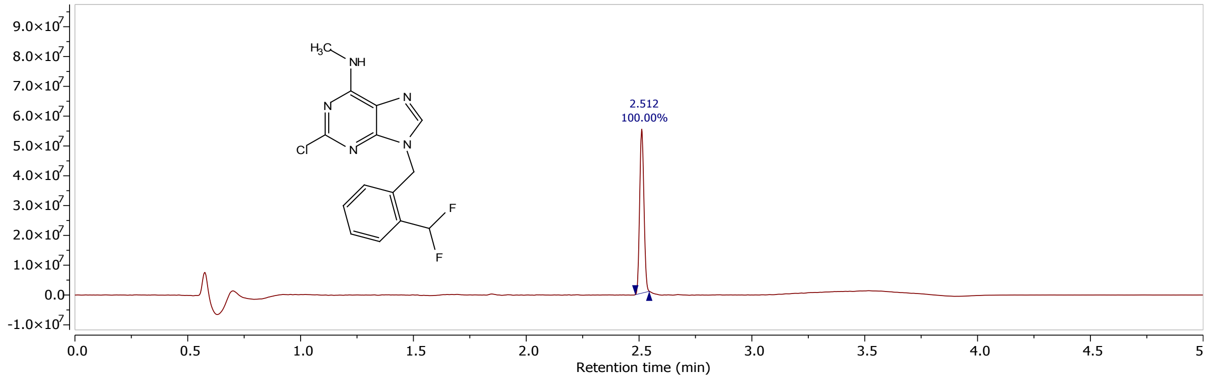


## 2-chloro-N-methyl-9-(2-(trifluoromethyl)benzyl)-9H-purin-6-amine 17

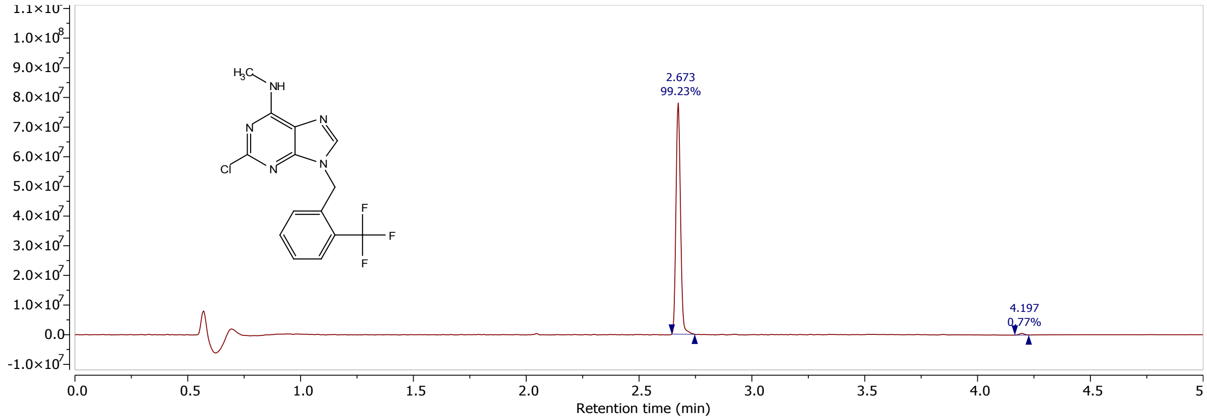


## 2-chloro-9-(2-methoxybenzyl)-N-methyl-9H-purin-6-amine 18

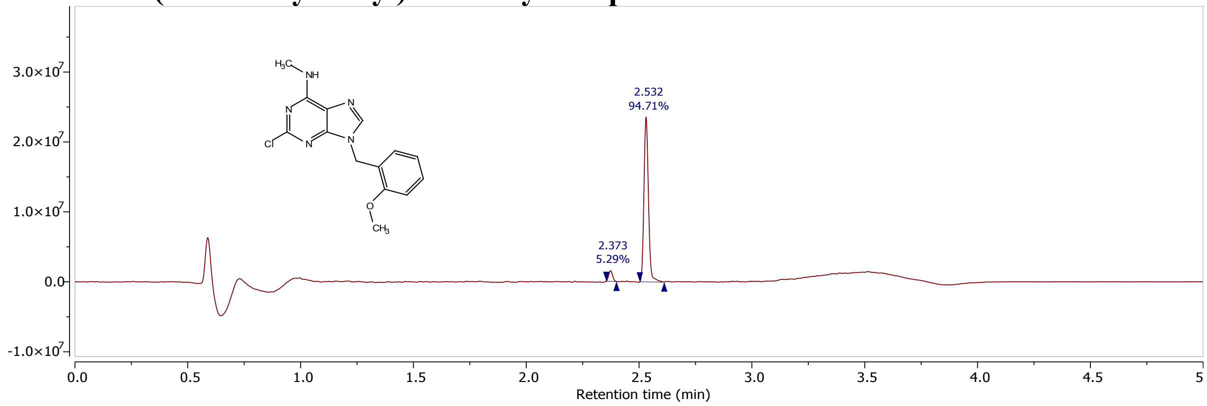

## methyl 3-((2-chloro-6-(methylamino)-9H-purin-9-yl)methyl)benzoate 19

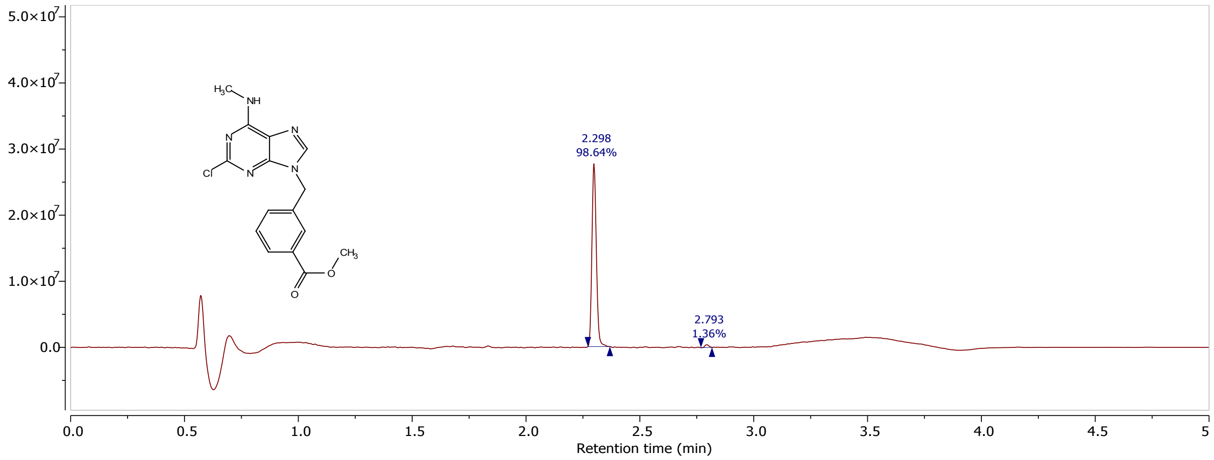


## 3-((2-chloro-6-(methylamino)-9H-purin-9-yl)methyl)benzoic acid 20

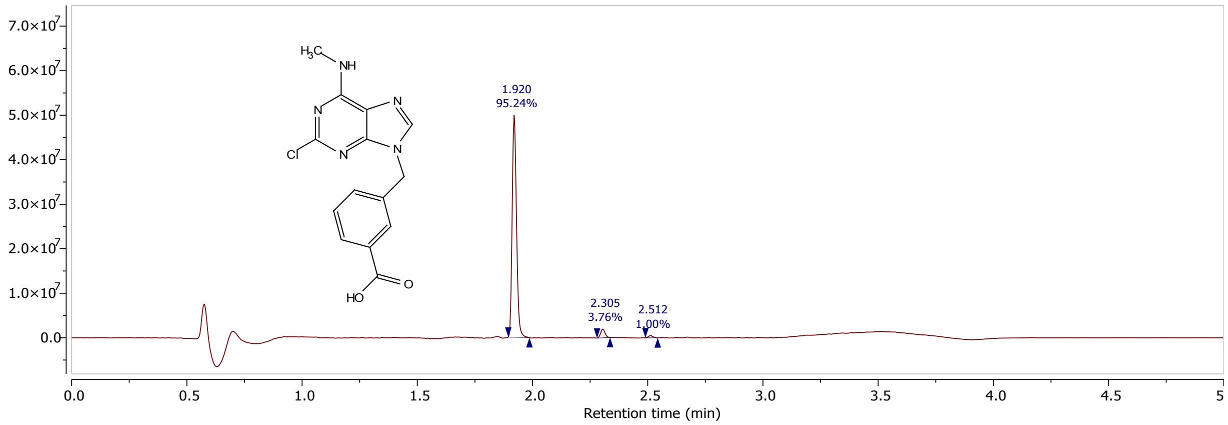


## 3-(2H-tetrazol-5-yl)benzyl)-2-chloro-N-methyl-9H-purin-6-amine 21

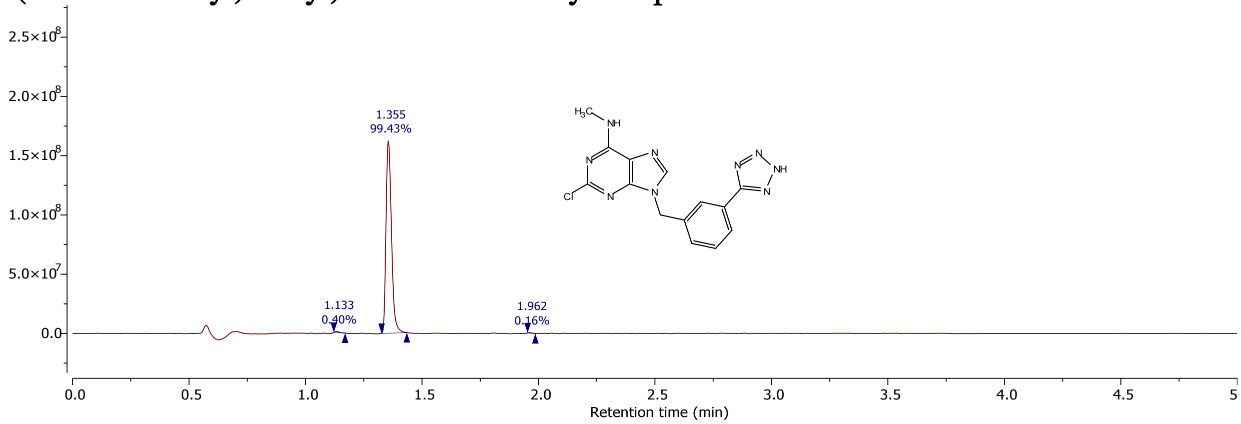

## 3-((2-chloro-6-(methylamino)-9H-purin-9-yl)methyl)benzamide 22

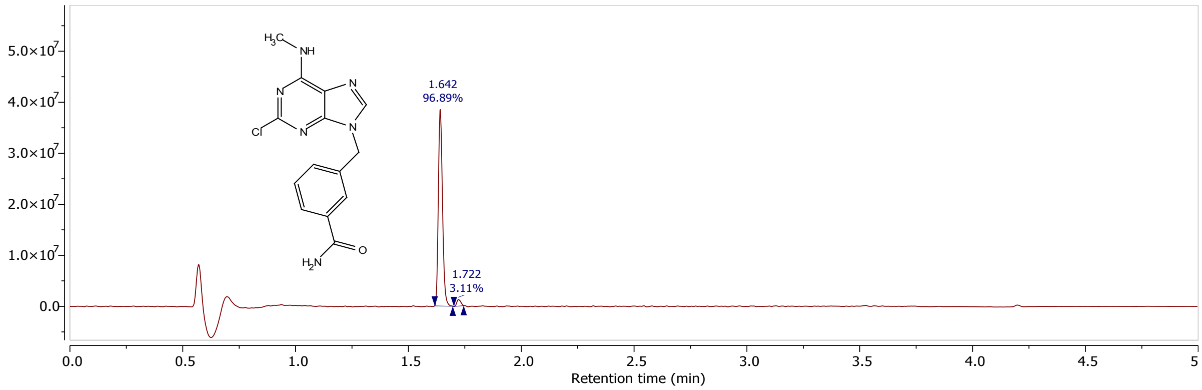


## 3-((2-chloro-6-(methylamino)-9H-purin-9-yl)methyl)-N-methyl benzamide 23

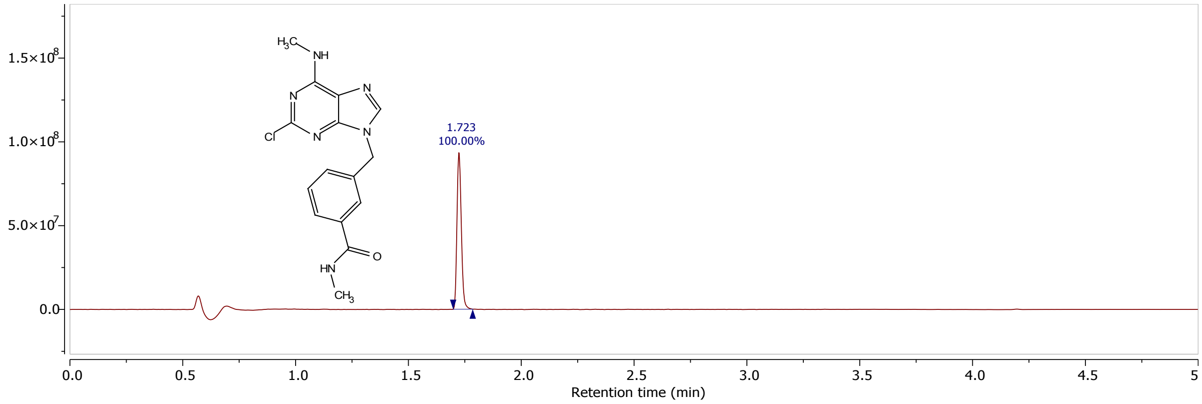


## 9-(3-bromobenzyl)-2-chloro-N-methyl-9H-purin-6-amine 24

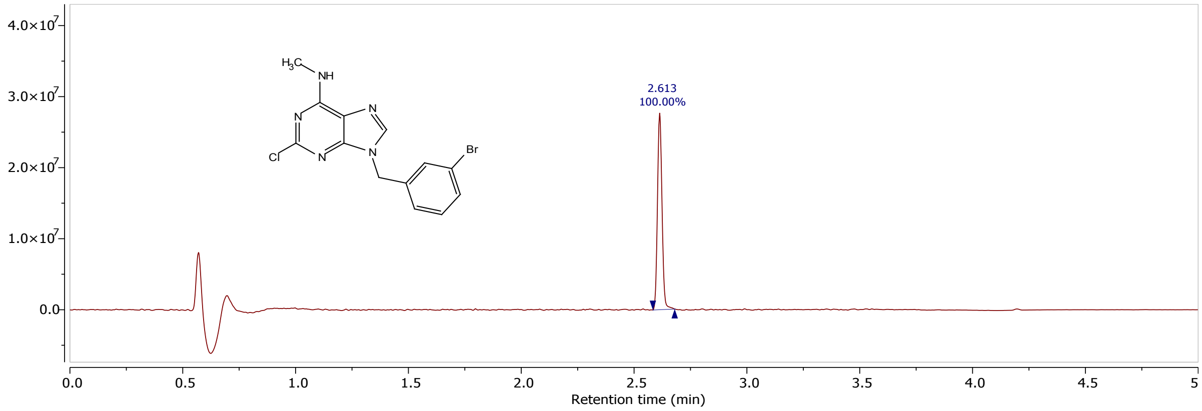

## 2-chloro-9-(3-chlorobenzyl)-N-methyl-9H-purin-6-amine 25

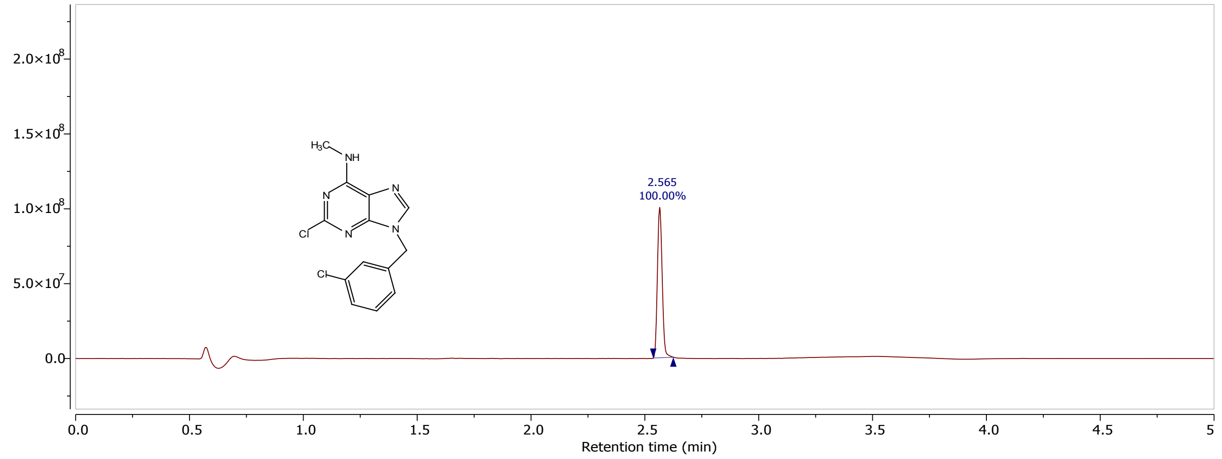


## 3-((2-chloro-6-(methylamino)-9H-purin-9-yl)methyl)benzonitrile 26

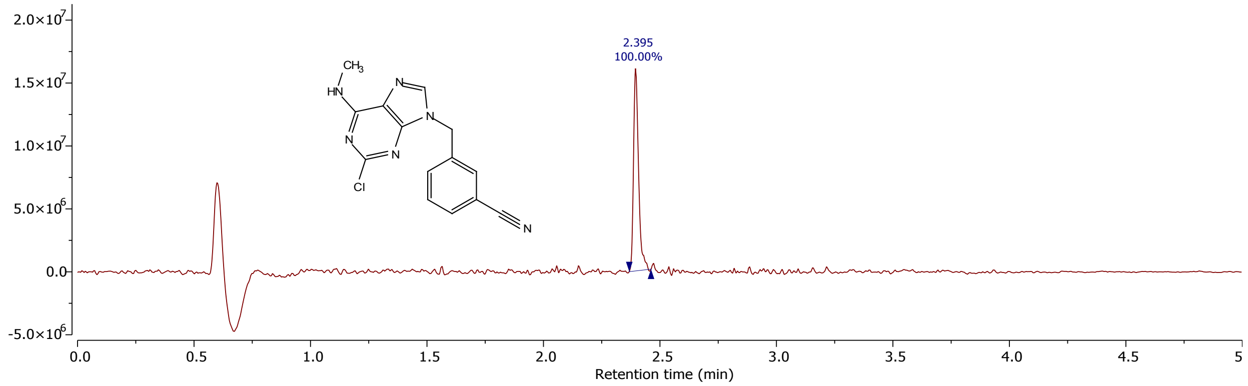


## 2-chloro-9-(3-methoxybenzyl)-N-methyl-9H-purin-6-amine 27

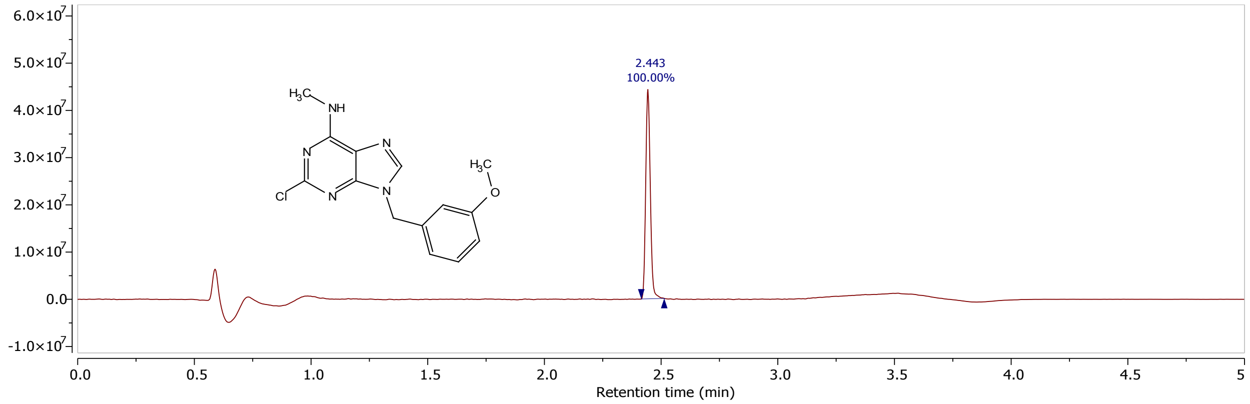

## methyl 4-((2-chloro-6-(methylamino)-9H-purin-9-yl)methyl)benzoate 28

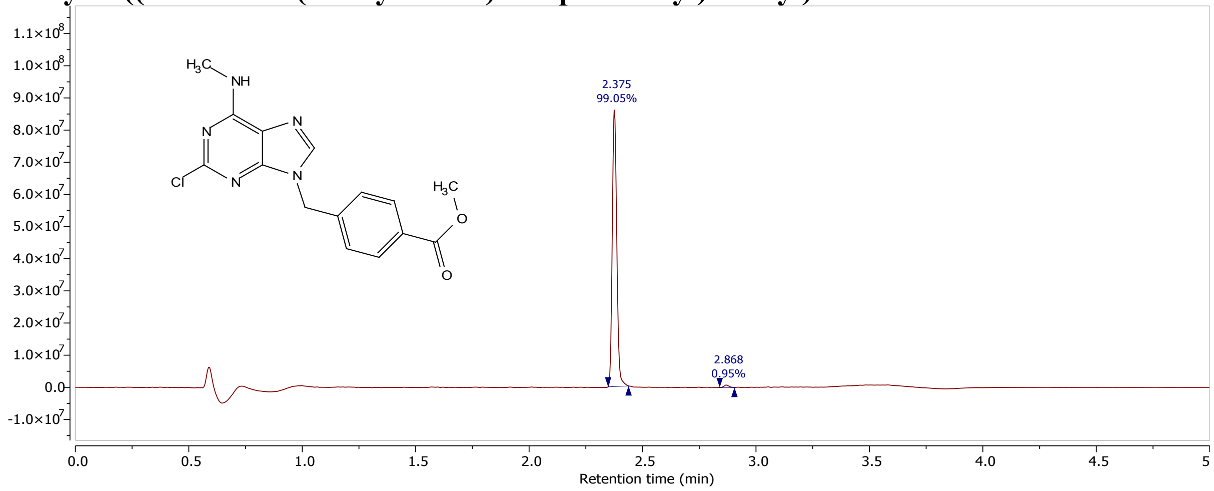


## 3-((2-chloro-6-(methylamino)-9H-purin-9-yl)methyl)benzoic acid 29

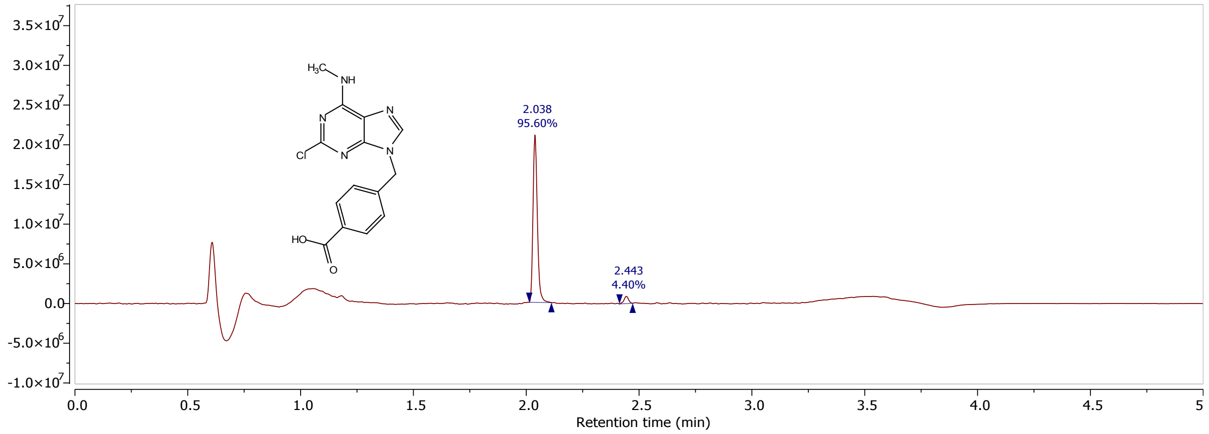


## (4-((2,6-dichloro-9H-purin-9-yl)methyl)phenyl)methanol 30

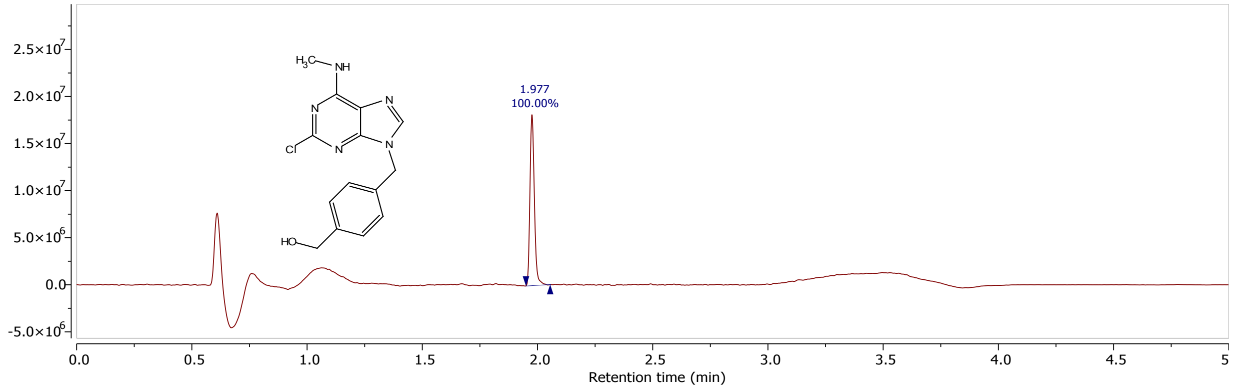

**N-(4-chloro-2-((2-chloro-6-(methylamino)-9H-purin-9-yl) methyl)phenyl) methanesulfonamide 31**

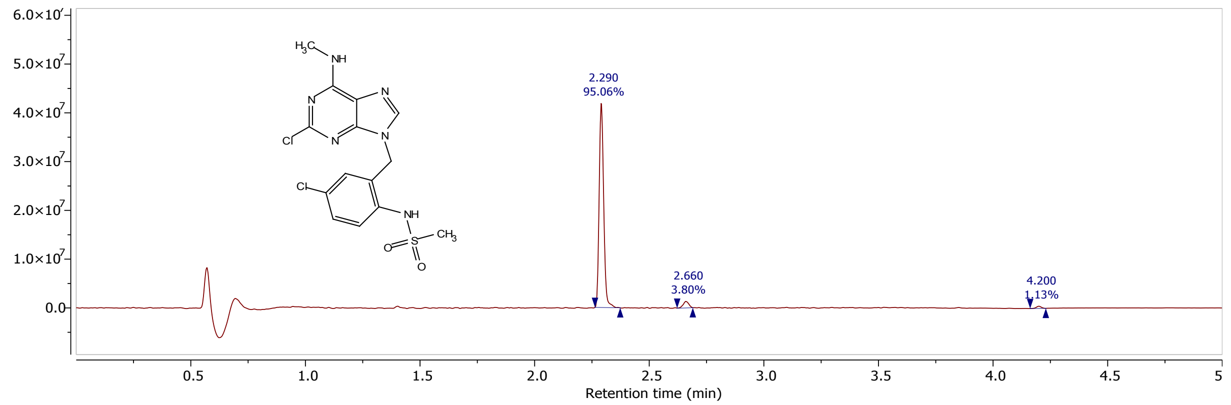


***3*-chloro-5-((2-chloro-6-(methylamino)-9H-purin-9-yl)methyl)benzoic acid 32**

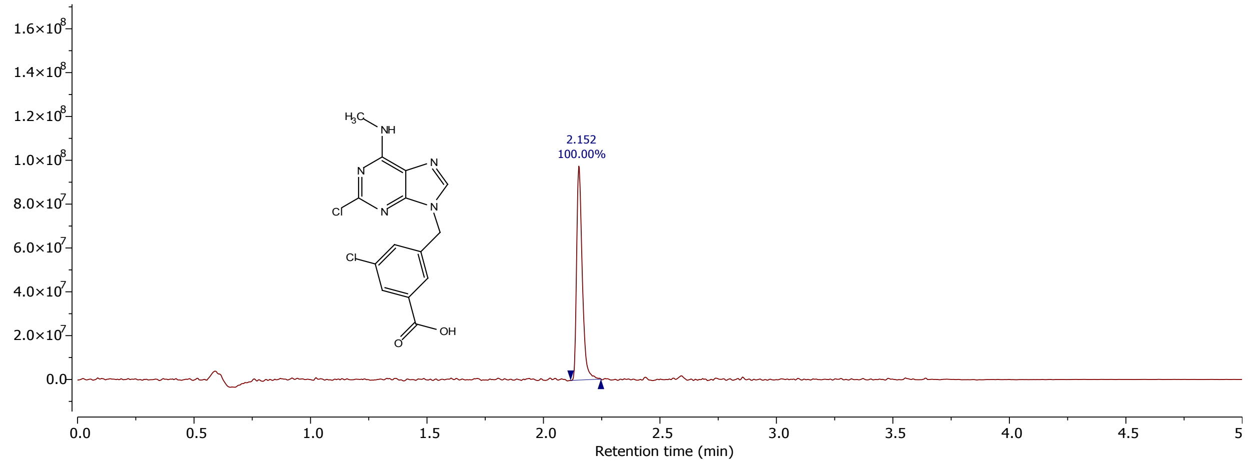


**methyl 3-chloro-5-((2-chloro-6-(methylamino)-9H-purin-9-yl)methyl)benzoate 33**

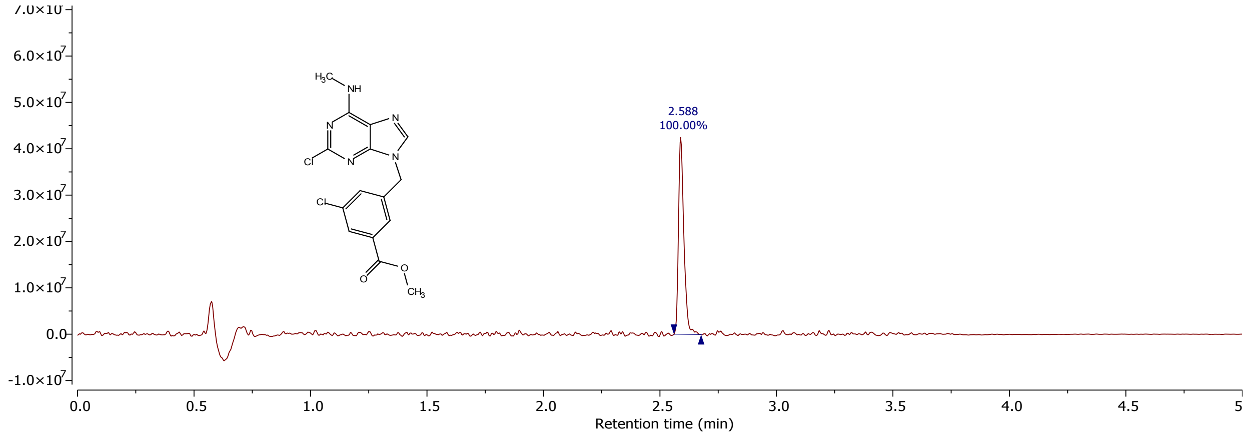

## 2-chloro-9-(3-chloro-4-methoxybenzyl)-N-methyl-9H-purin-6-amine 34

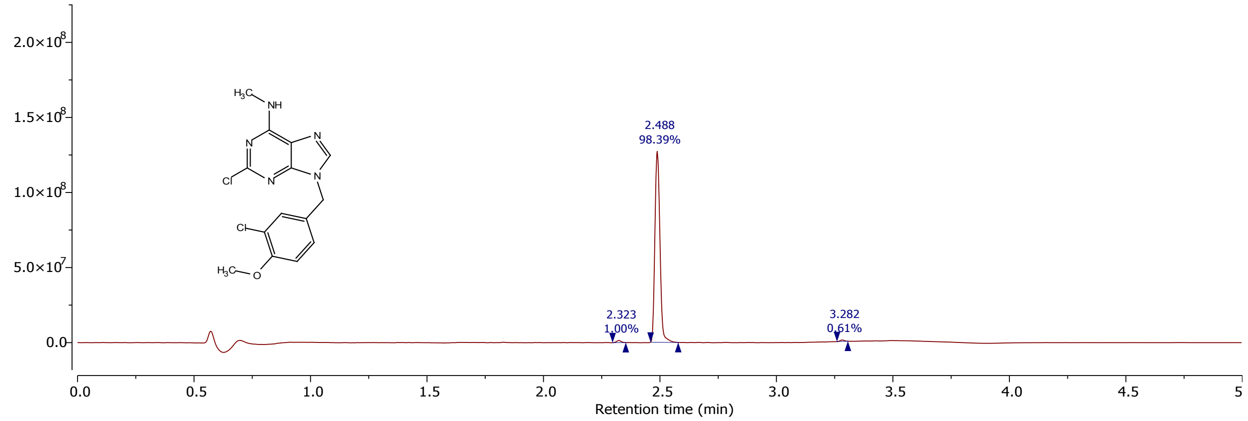


## 2-chloro-9-(3,5-dichloro-4-methoxybenzyl)-N-methyl-9H-purin-6-amine 35

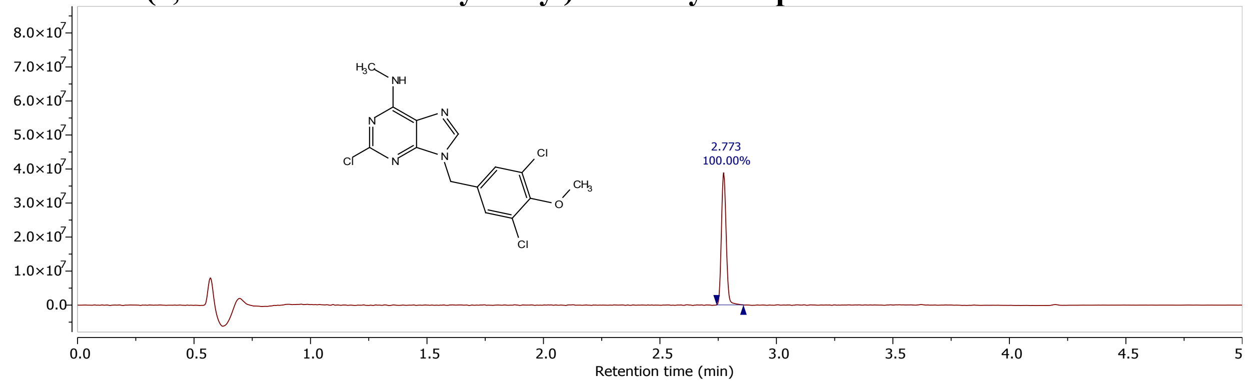


## 9-(3-chlorobenzyl)-2-fluoro-N-methyl-9H-purin-6-amine 36

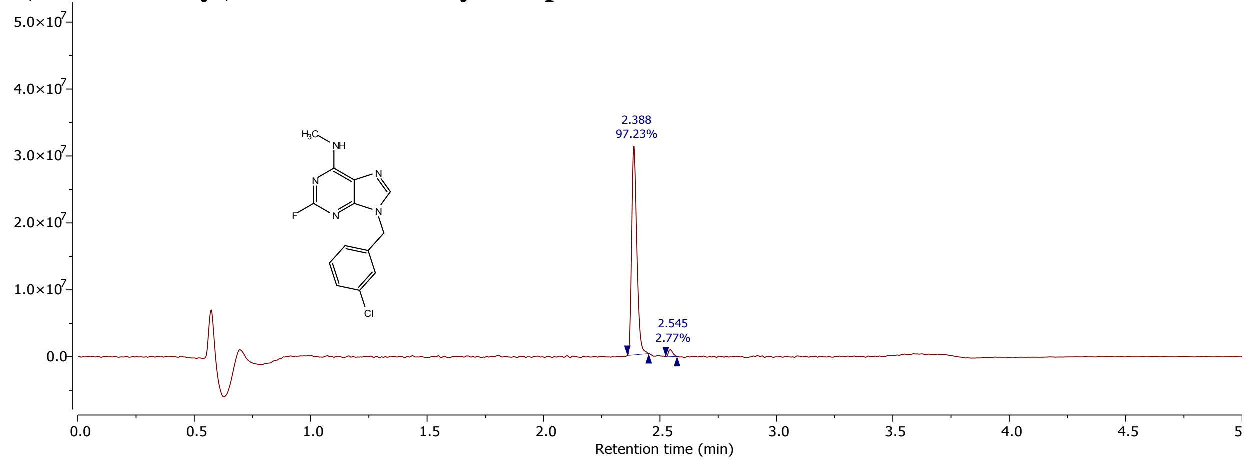

## 2-chloro-9-(3-chlorobenzyl)-N-cyclopropyl-9H-purin-6-amine 37

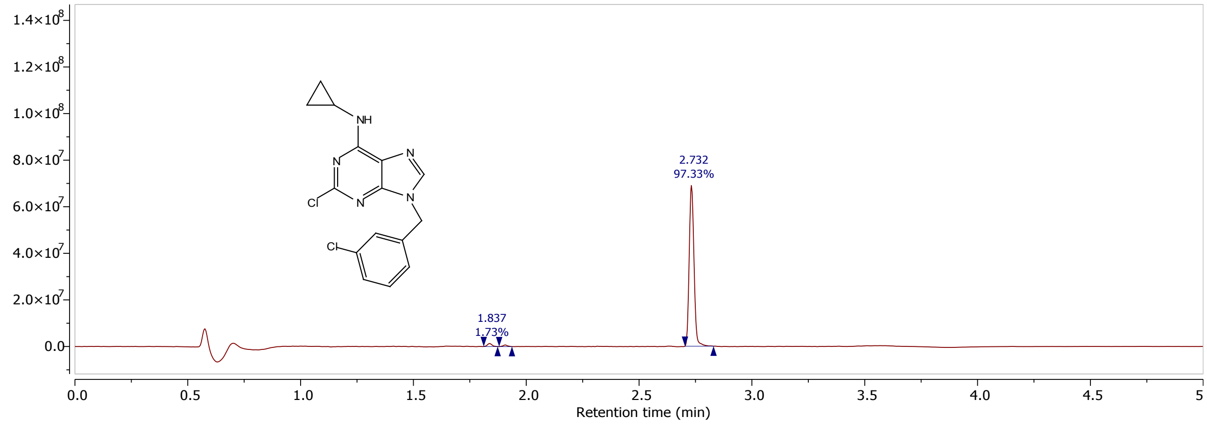


## 2,6-dichloro-9-(3-chlorobenzyl)-9H-purine 38

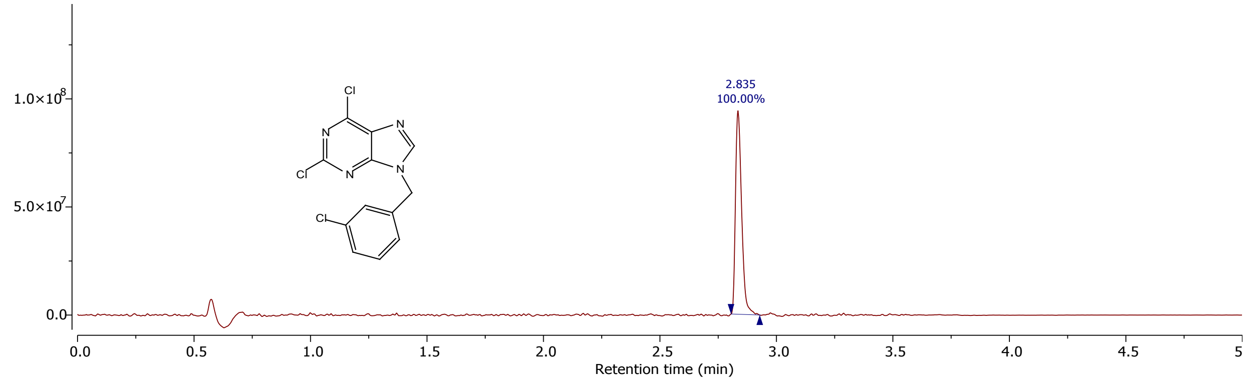


## 6-chloro-9-(3-chlorobenzyl)-N-methyl-9H-purin-2-amine 39

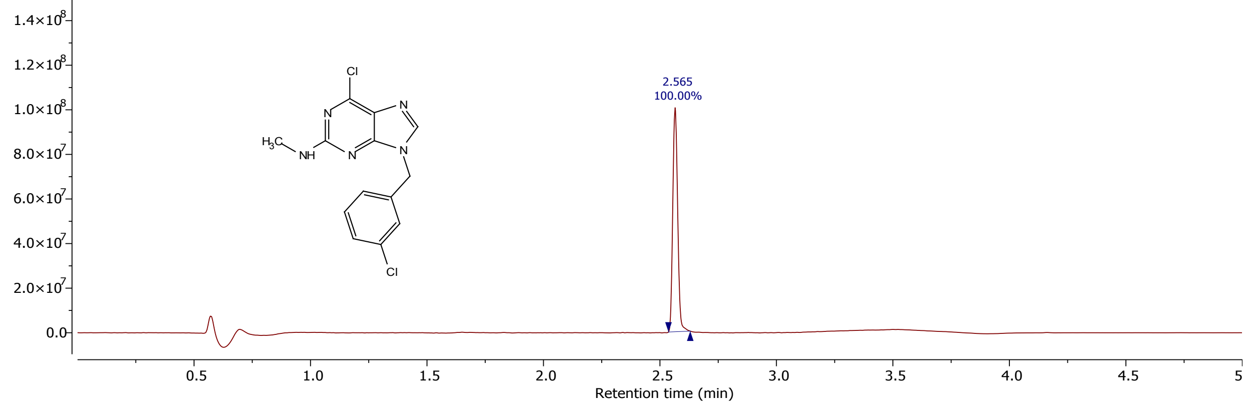

## 5-chloro-3-(3-chlorobenzyl)-N-methyl-2H-pyrazolo[4,3-d]pyrimidin-7-amine 40

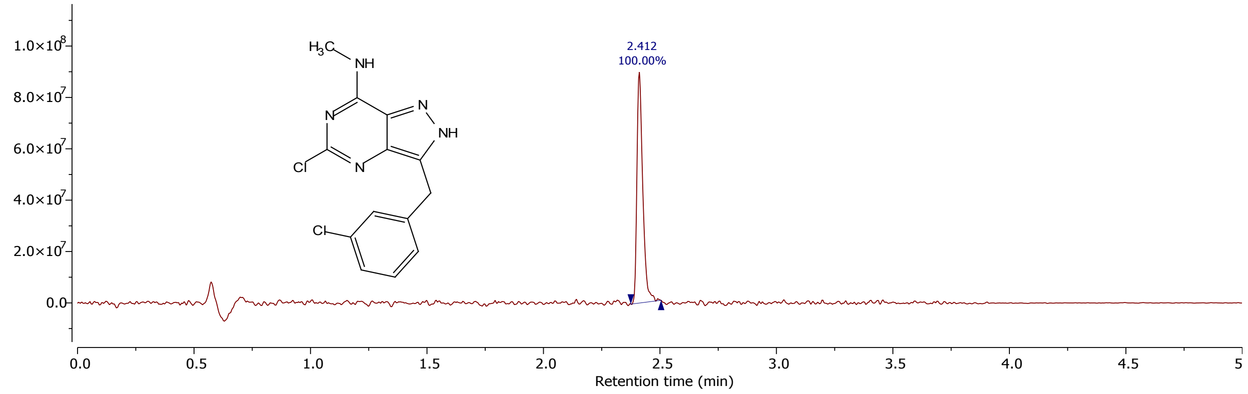

Supplement: Supplementary file 1 — jm4c00599_si_001.pdf [file jm4c00599_si_001.pdf]
